# A point-of-care ultrasound education curriculum for pediatric critical care medicine

**DOI:** 10.1186/s13089-022-00290-6

**Published:** 2022-10-31

**Authors:** Vidit Bhargava, Bereketeab Haileselassie, Samuel Rosenblatt, Mark Baker, Kevin Kuo, Erik Su

**Affiliations:** 1 Department of Pediatrics, Division of Critical Care Medicine, University of Alabama, 1600 7th Avenue S CPPI Suite 102, Birmingham, AL 35233 USA; 2 Department of Chemical and Systems Biology, Stanford University School of Medicine, Stanford, CA USA; 3 Department of Anesthesiology and Critical Care Medicine, Children’s Hospital of Philadelphia, Philadelphia, PA USA; 4 Department of Pediatrics, Division of Pediatric Emergency Medicine, University of Alabama, Birmingham, AL USA; 5 Department of Pediatrics, Division of Critical Care Medicine, Stanford University School of Medicine, Palo Alto, CA USA; 6 Department of Pediatrics, Division of Critical Care Medicine, McGovern School of Medicine, Baylor College of Medicine at Texas Children’s Hospital, Houston, TX USA

**Keywords:** Echocardiography, Procedures, Pediatric Intensive Care Unit, Simulation, Respiratory failure, Shock

## Abstract

**Background:**

Diagnostic and procedural point-of-care ultrasound (POCUS) change patient management with the potential to improve outcomes. Pediatric critical care medicine trainees have limited access to education and training opportunities in diagnostic POCUS in the pediatric ICU. A dearth of published pediatric ICU curricular resources restricts these educational opportunities.

**Methods:**

A 7-week longitudinal curriculum including lectures, practical skills sessions, and knowledge assessment covering core modules including (1) machine operation, (2) vascular access, (3) non-vascular procedures, (4) cardiac imaging, (5) hemodynamic assessment, (6) pulmonary imaging, and (7) abdominal imaging, was disseminated to pediatric critical care trainees and faculty at a single tertiary care pediatric hospital.

**Results:**

The knowledge of trainees and participating faculty in procedural and diagnostic POCUS improved after implementing the curriculum. Pre-test scores mean and standard deviation (59.30% ± 14.15%) improved significantly (75.60% ± 9.43%) for all learners (*p* < 0.001). The overall self-reported comfort in diagnostic and procedural ultrasound improved for all learners. 100% of the learners reported utilizing diagnostic POCUS in their clinical practice four months after disseminating the curriculum.

**Discussion:**

We describe a single center’s approach to POCUS education with improvement in knowledge, self-reported comfort, and attitudes towards procedural and diagnostic POCUS. The curricular resources for adaptation in a similar educational context are provided.

**Supplementary Information:**

The online version contains supplementary material available at 10.1186/s13089-022-00290-6.

## Educational objectives

By the end of this activity, The learners of the curriculum will be able to

1. Explain fundamental ultrasound physics.
2. Describe evidence-based applications of point-of-care ultrasound in caring for children in the pediatric critical care unit.
3. Demonstrate the knowledge base and technical skills necessary to acquire ultrasound images in a patient.
4. Interpret ultrasound images in a pediatric critical care patient using the appropriate clinical context

**Supplementary Information:**

The online version contains supplementary material available at 10.1186/s13089-022-00290-6.

## Background

Point-of-care ultrasound (POCUS) is progressively gaining acceptance in the practice of pediatric critical care medicine [1]. It has become a standard of care for vascular access procedures in addition to applications in thoracentesis and paracentesis due to improved safety and efficiency in some of these procedures [2]. POCUS as a diagnostic tool is also promising given its potential for improving diagnostic accuracy and workflow efficiency [3]. Compared to traditional ultrasound imaging, POCUS simplifies processes by having clinicians caring for critically ill children operate and interpret ultrasound imaging in real time, allowing for serial exams and limiting interobserver variability [4]. POCUS also improves the overall diagnostic accuracy, decrease the time to intervention, and thus leads to changes in patient management with the potential to impact outcomes [5].

Despite its utility and many advantages, opportunities for diagnostic POCUS training are limited for pediatric intensive care physicians [6]. While the first programs for teaching POCUS within pediatric critical care have been established recently, they notably occur at a few large children’s hospitals [7, 8]. Additional opportunities for POCUS education include national training courses; however, these courses are offered infrequently, particularly for pediatrics. Each of these factors impedes rapid POCUS dissemination within pediatric critical care. Thus, most pediatric critical care trainees and practitioners have not been trained in POCUS.

Ideally, given the clear potential to improve diagnostic ability and patient care, all current pediatric critical care trainees would have training in POCUS that equips each learner with the knowledge and skills needed to use POCUS to care for their patients. In January 2020, the Joint Commission endorsed a statement from the Emergency Care Research Institute (ECRI) citing POCUS as a potential hazard to patients for reasons related to training and skill verification, oversight of use, and recordkeeping and accountability mechanisms for clinical use [9]. This highlights the need for programmatic development and education as well as training and certification standards necessary to implement POCUS safely [10].

Published educational recommendations and curricula for teaching POCUS in the PICU are sparse [3, 11, 12]. Education in diagnostic applications as a part of complete POCUS education is a necessary and natural progression of the field. Education specific to pediatric critical care medicine is indicated so that providers can conceptualize its use in the specialty. Its implementation may require attention to institutional resource availability and patient care needs. This study's objective was to develop an institutional ultrasound curriculum with clear objectives and outcome assessment in the pediatric ICU of a tertiary referral children’s hospital. A secondary objective was a description of the curriculum for consideration to be used as a guide for other institutions considering implementing POCUS education. This curriculum targets pediatric critical care trainees and faculty with little or no prior training in diagnostic and/or procedural POCUS.

## Methods

We utilized Kern’s model of curriculum development as a conceptual framework for our study [13]. This includes six interrelated steps: problem identification, needs assessment, goals and objectives, educational strategies, implementation, and evaluation and feedback.

### Target learners

The target learners for this curriculum in POCUS are all pediatric critical care fellowship trainees at the University of Alabama (UAB) School of Medicine. These learners have completed an ACGME accredited pediatric residency training and underwent fellowship instruction in using POCUS for central venous catheter insertion. Thus, the learners had a baseline understanding and familiarity with the equipment and general techniques needed for ultrasound imaging of vasculature. As part of our needs assessment, a pre-course survey was distributed to all learners before implementing the curriculum to understand their specific needs (Additional file 1). The 5-question pre-course survey included demographics, prior POCUS learning, and the comfort level in diagnostic and procedural POCUS. The results of this survey are discussed in the results section.

### The learning environment and health education context

The learning environment for POCUS at the study institution benefits from several factors. The adult and pediatric emergency medicine programs have an existing curriculum led by collaborating faculty with vital interests in ultrasound education. The pediatric intensive care unit has equipment capable of diagnostic thoracic and abdominal imaging and procedural applications. The institution is a level I trauma center with dedicated pediatric critical care, burn, and cardiac critical care units. The pediatric intensive care unit has more than 1,500 patient admissions per year, cares for medical and surgical patients, and provides clinicians ample opportunity to apply POCUS.

At the institutional level, interested parties formed a coalition of pediatric critical care and pediatric emergency medicine faculty to address POCUS activities. The consultation was also obtained from national experts (B.H., E.S., K.A, Y.D.) for both curricular design and implementation. The pediatric intensive care unit can be very busy, and allowing time for dedicated instruction, directly observed practice, and reviewing images is a major limiting factor. This applies to both faculty and fellows. With seven fellows simultaneously providing patient care in diverse locations throughout the health system, including the PICU, the pediatric cardiothoracic intensive care unit, adult trauma/burn, palliative care, and anesthesia, there are significant logistical challenges in scheduling additional time for instruction and practice. The same applies to the schedules of a limited number of faculty members who can serve as instructors. Time for dedicated POCUS education, observation, and image review was coordinated in the PICU environment for faculty and fellows. All learners included in the study completed a pre-course knowledge assessment before attending these didactics (Additional file 2).

Goals and objectives were created based upon general and targeted needs assessments.

*Goal* Pediatric critical care fellows will develop the knowledge and skills to use POCUS in caring for pediatric intensive care unit patients.

*Objectives* By the end of the curriculum, the learner will be able to demonstrate the following knowledge and psychomotor skill objectives:

- *1.* *Machine operation* (Additional file 3)
  - Describe the basics of ultrasound physics, including wave characteristics, wave reflection and absorption, and understanding and recognition of common ultrasound artifacts.
  - Demonstrate, as assessed by faculty through at least one direct observation while scanning a live model, choosing an appropriate ultrasound probe, and setting up the machine.
  - Demonstrate, as assessed by faculty through at least one direct observation while scanning a live model, image optimization for respective applications by adjusting gain, depth, frequency, and other relevant ultrasound settings.
  - Demonstrate, as assessed by faculty through at least one direct observation while scanning a live model, accountability for imaging data by saving relevant images and videos and appropriately documenting their interpretation of the saved images and videos.
- *2. Procedural ultrasound*
  - *2a. Vascular access* (Additional file 4)
    - Describe the long-axis (longitudinal, or in-plane) and short-axis (transverse, or out-of-plane) techniques of obtaining vascular access.
    - Describe the advantages and pitfalls of using the short technique over the long technique.
    - As demonstrated in simulation practice models, obtain vascular access in venous and arterial structures using long- or short-axis technique.
- *2b. Non-vascular procedures* (Additional file 5)
  - Describe the use of POCUS in performing drainage procedures of the peritoneum and pleura.
  - Recognize landmarks necessary to visualize for thoracentesis, paracentesis, and US-guided lumbar puncture.
- *3. Diagnostic ultrasound*
  - *3a. Cardiac imaging* (Additional file 6)
    - Describe at least five views (parasternal long, parasternal short, subcostal long-axis, apical four chamber and subcostal IVC views) for focused cardiac exams.
    - Demonstrate the choice of the appropriate probe, preset, image acquisition, identification of anatomical structures, and optimization of the image using depth, gain and probe movement
- *3b. Hemodynamic assessment* (Additional file 7)
  - Identify hemodynamically significant pericardial effusion.
  - Identify severely depressed LV systolic function using qualitative and quantitative measures [E-Point Septal Separation (EPSS) and Fractional Shortening (FS)].
  - Describe the effect of respiratory variation on stroke volume [Aortic Flow Variability (AFV) or Velocity Time Integral (VTi)] as a surrogate for intravascular volume status
  - Describe pitfalls and limitations of qualitative and quantitative measures, M mode, and Doppler techniques used in the hemodynamic assessment.
- *3c. Pulmonary imaging* (Additional file 8)
  - Describe a focused pulmonary exam using pleural ultrasound.
  - Identify findings consistent with the recognition of pneumothorax. pleural effusion, and consolidation.
  - Describe the pitfalls and limitations of these techniques in pulmonary imaging.
- *3d. Abdominal diagnostic imaging* (Additional file 9)
  - Describe four views necessary to identify free fluid using the FAST exam and two urinary bladder views to perform a bladder volume assessment.
  - Describe pitfalls and limitations of focused ultrasound techniques in abdominal imaging compared to abdominal computed tomography scans

With these goals and objectives in mind, we pursued a 7-week longitudinal series that included seven core modules covering the abovementioned objectives. These included: (1) machine operation, (2) vascular access, (3) non-vascular procedures, (4) cardiac Imaging, (5) hemodynamic assessment, (6) pulmonary imaging, and (7) abdominal imaging. We delivered one module per week, and each module included a 45-min lecture and a one-hour hands-on session. All fellows had protected time to attend live or recorded classes and completed a pre-course assessment for each core module before attending these didactics (Additional file 2). The lectures were delivered by PICU faculty (V.B.), a guest faculty (E.S.), and an emergency medicine faculty (M.B.). The hands-on sessions were facilitated by PICU (V.B.) and emergency medicine faculty (M.B.). The pre-course assessment was modeled after the online emergency ultrasound test created by the emergency ultrasound section of the American College of Emergency Physicians (ACEP) [14].

Practical skill sessions were performed soon after the didactic sessions. During the practical session, each fellow was required to obtain images and identify anatomical structures satisfactorily after a demonstration by the instructor. Three fellows accompanied each instructor during practical sessions, and all fellows completed these sessions. For the practical session, the clinical team selected patients and discussed the patient’s history and safety for the performance of an ultrasound exam with the ultrasound faculty before the teaching session. If there were no contraindications to the patient’s involvement, then the faculty approached the patient’s family for verbal consent for education.

### Learner assessment

A post-course assessment and survey were conducted 4 months after completion of the curriculum (Additional file 2). The post-course assessment included 30 multiple choice questions and evaluated knowledge but not psychomotor skills. Both the pre- and post-course assessments were modeled after the online emergency ultrasound test created by the emergency ultrasound section of ACEP and included still images and video clips [14]. The assessments were not separately validated before use. The post-course survey had questions assessing the learner’s comfort in their procedural and diagnostic ultrasound skills since taking the course on a 7-point Likert scale and the percentage of learners integrating diagnostic POCUS in their clinical practice (Additional file 10)**.**

## Results

We evaluated the impact of our curriculum through pre–post knowledge assessments, practice surveys, and direct observations to assess reaction, learning, and behavior results. A targeted needs assessment was completed before planning and disseminating the course. Seven critical care fellows (Two 1st year trainees, three 2nd year trainees, and two 3rd year trainees) and three faculty completed the survey and participated in the course. One faculty member had participated in the 2-day course offered by the Society of Critical Care Medicine (SCCM) and used POCUS clinically in their practice. The other two faculty had also attended the 2-day ultrasound course offered by SCCM and had incidental practice. Four fellows were exposed to ultrasound and had no prior experience with diagnostic ultrasound. Six learners expressed comfort in being able to operate the ultrasound machine. All learners were interested in applying diagnostic and procedural ultrasound to their practice after taking the course.

**Fig. 1:**
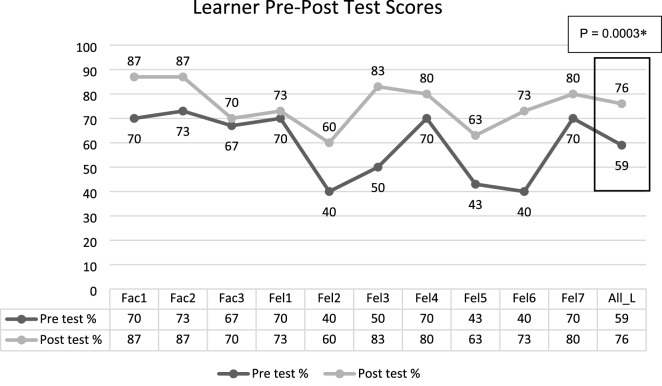
Learner pre- and post-course test scores for individual learners, including faculty and fellows. The mean of post-test scores for all learners improved significantly after the curriculum (p = 0.0003). *Fac* faculty, *Fel* fellow*, All_L* all learners

All learners participated in all educational sessions. The learners completed a knowledge assessment before taking the course and a post-course knowledge assessment afterward. Figure 1 illustrates the results from the pre- and post-course assessments. All learners demonstrated an improvement in scores after taking the course. Assessment scores improved even for experienced faculty learners. Pre-test scores mean and standard deviation was 59.30% ± 14.15% for all learners. Post-test scores improved to a mean and standard deviation of 75.60% ± 9.43% for all learners (p < 0.001). A post-course survey (Likert scale 1–7) was performed four months after the curriculum to assess the leaner’s reaction to the curriculum. In this survey, two faculty learners reported feeling extremely comfortable with procedural and diagnostic ultrasound, two fellows reported feeling moderately comfortable, and the rest of the five fellows and one faculty learner reported feeling slightly comfortable. None of the learners reported being uncomfortable with their procedural ultrasound skills after taking the course. The median score from the Likert scale was 5, with a range of 5–7. The second question sought to assess the behavior change and understand the percentage of learners utilizing diagnostic ultrasound in their clinical practice using yes or no choices. 100% of the learners reported utilizing diagnostic ultrasound in their clinical practice.

## Discussion

This manuscript describes the development and implementation of a longitudinal evidence-based POCUS curriculum for pediatric intensive care providers at a single center. This curriculum was designed as an introductory course for learners without prior ultrasound training. A targeted needs assessment suggested a lack of prior training and educational opportunities for critical care fellows in diagnostic and procedural POCUS. There was heterogeneity in prior experience and comfort, but all learners intended to integrate diagnostic and procedural ultrasound in patient care. The curriculum allowed the learners in a busy environment to participate and engage in this educational opportunity. The learners’ knowledge and self-reported comfort with POCUS increased after the course.

This curriculum attempts to address the dearth of resources in pediatric critical care POCUS education using a single center’s experience and provides context and resources in which the implementation of this curriculum was successful. With the absence of pre-existing infrastructure, the pooling of resources at the authors’ institution through partnerships with adult and pediatric emergency medicine POCUS programs and leveraging non-institutional resources such as expert speakers from other institutions facilitated the dissemination of content. The PICU leadership and the divisions of radiology and cardiology acknowledged the benefit of POCUS education to critical care trainees and supported the curriculum. Two ultrasound machines equipped for diagnostic and procedural POCUS applications were available to the PICU providers at all times (Venue, General Electric Company, Waukesha, WI) The lectures and hands-on sessions were strategically scheduled after the transition of overnight patient care, and the trainees were provided protected educational time by faculty and advanced practice providers shouldering patient care responsibilities. In rare cases, when a trainee could not attend the scheduled academic session, the smaller size of the fellowship program permitted one-on-one time for catch-up. Hands-on sessions were supervised by critical care (V.B.) and emergency medicine (M.B.) faculty with formal training in POCUS through completing emergency ultrasound fellowships. Recordkeeping for quality assurance and credentials maintenance was done by manually archiving images and documenting using password-protected databases. A 7-week longitudinal curriculum was selected over shorter courses due to information retention concerns demonstrated in previous studies [15, 16]. A dedicated time was provided for the dissemination of this curriculum by the PICU leadership, which was different from the pre-existing educational conferences. This allowed prioritization of the ultrasound education without taking away time and focus from other educational opportunities for the trainees.

This curriculum differs from the previously published literature and the educational context in several ways. Conlon et al. published their experience implementing the POCUS curriculum in a pediatric critical care unit at a large academic center [7]. It included a 16-h introductory course with didactic and hands-on training provided by multidisciplinary educators. Thirteen critical care faculties and three trainees completed their initial course. Two faculty members met the requirements for credentialing in all core competencies, and two completed the requirement in hemodynamic POCUS by completing at least 25 acceptable exams after taking the course. They also demonstrated changes in patient care with the implementation of their curriculum. However, several factors may preclude the translation of this experience to other centers. Their POCUS program benefitted from the presence of six critical care faculty trained in POCUS and multiple other faculty in pediatric and adult emergency medicine and support from radiology and cardiology. A pre-existing pathway for credentialing also provided a clear path and structure to the curriculum. These resources and infrastructure may not be present at many other centers. Good et al. described their experience implementing POCUS education in a pediatric critical care unit for pediatric residents. They utilized asynchronous learning, where the residents self-completed three educational modules, followed by weekly one-hour supervised hands-on sessions. The residents were provided education during their month-long rotation in the PICU. Six residents completed the curriculum and demonstrated a significant improvement in test scores (55–90%) compared to historical cohorts. Educational resources were also published and made available for implementation at other centers. However, the scope of this curriculum was geared towards pediatric residents as opposed to pediatric critical care medicine specialists. Several high-quality, free open access resources are available online for POCUS education. These resources though not primarily geared towards pediatric critical care, can be collated, and utilized with some modifications to the content. A list of such educational resources is presented in (Additional file 11). In contrast, this curriculum provides a comprehensive resource that can serve as a framework for POCUS education for pediatric critical care trainees. The resource incorporates evidence-based literature and clinical scenarios that are relevant to the pediatric critical care practice. This curriculum can be time and resource-saving for educators that may not have time and resources available to collate resources from multiple sources or create their own.

Several factors hinder the adoption of POCUS education in pediatric critical care training programs [17]. In the results of an extensive national survey, 83% (43/52) of the respondents agreed that POCUS education should be a core component of pediatric critical care training. However, only 67% (35/52) of the programs provided trainee education in diagnostic POCUS. Other components of POCUS education, such as credentialing, documentation, image storage, and quality assurance, were present in less than 25% of the programs. The larger programs were more likely to have these components in place. Lack of trained ultrasound faculty and oversight were common barriers to implementing POCUS education [17]. Brant et al. evaluated a longitudinal POCUS curriculum for pediatric residents [18] and reported a 61–90% improvement in learner comfort with the utilization of POCUS in their cohort. However, the adoption of POCUS into clinical practice was limited in their study. More than 90% of the learners performed less than 5 POCUS exams in the three months after the dissemination of the curriculum. They cited similar reasons for the limited adaption in their cohort of learners. Only 3 out of 91 faculty (pediatric emergency medicine and pediatricians) were credentialed using POCUS. The smaller number of critical care trainees allowed us to implement this curriculum despite the limited availability of trained faculty. We pooled resources from multiple divisions to supervise and oversee the trainees. However, the feasibility of this practice in the long term and as the number of learners increases is unknown. Other institutions will likely have to develop creative solutions to address these barriers locally.

Competency in POCUS is utilizing provider knowledge, skills, and attitudes in patient care. It is broadly sub-structured into four domains: understanding the indications for performing an exam, acquiring appropriate images both in terms of suitability for the indication and quality, interpreting the exam, and finally integrating the exam in patient care [14]. The perfect tool for the assessment of learner competency is lacking. Nonetheless, tools such as written examination, image review, objective structured clinical examination (OSCE), standardized direct observation tool (SDOT), etc., are available at the discretion of the educators [19]. Utilizing these resources is time and resource intensive. This curriculum used written examination and direct observations to evaluate the success of the curriculum in achieving defined objectives. The learners took the written test before and at the end of the curriculum. All learners improved their individual test scores. The overall post-participation score improved significantly from a baseline of 59–75%. This is comparable to the improvement in the knowledge (from 60 to 80%) of a cohort of internal medicine residents training in thoracic ultrasonography [20]. Our post-interventions scores are slightly lower than a group of adult critical care trainees receiving a similar longitudinal curriculum. In this cohort, the post-interventions scores improved from 71 to 89% [21]. Direct observations were made during the hands-on session, whereby in-time feedback was provided to the learners to improve their image acquisition. Specifically, feedback was provided regarding the choice of the probe, image optimization using gain and depth, acquisition and optimization of views, and assessment.

Further, asynchronous feedback was provided to the learners through biweekly image review sessions. This curriculum did not assess the learners' integration of new skills into clinical practice. The number of studies performed or the change in patient management based on these studies was not evaluated by the curriculum and represented a limitation. OSCE and SDOT were not performed for competency assessment as part of this curriculum due to the lack of time and resources.

### Limitations to generalizability

This curriculum was implemented in a small cohort of pediatric critical care trainees and faculty and was subject to institutional biases. This may have contributed to the successful implementation of the curriculum as it allowed effective utilization of the limited resources and personnel. Educational program implementation is widely dependent on institutional factors, and additional work, optimally in a multi-institutional consensus fashion, is essential for determining common elements necessary for ultrasound education in pediatric critical care medicine as a specialty. A few factors can limit the applicability of this curriculum to other settings. First, this curriculum is based on the latest evidence compiled by the ESPNIC group and other well-curated reviews for patients admitted to pediatric critical care [3, 12]. While the principles and practices of POCUS remain the same, its applicability based on the setting is quite variable. This curriculum was delivered over seven weeks. This may not be feasible in specific programs and may need to be delivered over shorter or longer periods. The dissemination benefitted from the simultaneous availability of critical care trainees. However, this may not be replicable as it is at a different institution and may require adaptation based on local practices. Asynchronous learning through recorded lectures or dissemination of resources in this curriculum can be an alternative when limited by the simultaneous availability of critical care trainees. A lack of faculty with interest or training in POCUS can be a barrier to implementing this curriculum. In such cases, the training programs can tap on expertise outside the division, including emergency medicine, cardiology, or radiology or even their adult counterparts that may have already established their own training programs. The training programs can also seek help outside their institution to bring in experience and expertise that may not be available locally. Ultrasound machine availability is also a potential limitation for implementation at some centers that would require resource allocation.

## Conclusions

A 7-week longitudinal POCUS education curriculum implemented at a tertiary referral PICU may improve self-reported knowledge and implementation of POCUS in pediatric critical care clinicians. Future development in POCUS education wound likely benefit from investigations of its implementation in additional pediatric critical care medicine settings with ongoing skill assessment and evaluation of its impact on patient care and outcomes.

## Supplementary Information

**Additional file 1.** Needs assessment for learners of the point-of-care ultrasound curriculum

**Additional file 2.** Pre-course knowledge assessment of the learners

**Additional file 3.** The basics of machine operation, image acquisition and optimization and saving an image

**Additional file 4.** Ultrasound-guided vascular access procedures

**Additional file 5.** Ultrasound-guided non-vascular access and advanced procedures

**Additional file 6.** Focused echocardiographic examination

**Additional file 7.** Hemodynamic assessment of a critically ill patient using point-of-care ultrasound

**Additional file 8.** Focused thoracic imaging in a critically ill patient

**Additional file 9.** Focused assessment using sonography in trauma

**Additional file 10.** Post-course survey to assess the impact of the curriculum on the learners

**Additional file 11.** High-quality, free open access resources for point-of-care ultrasound education

## Data Availability

The datasets used and analyzed during the current study are available from the corresponding author on reasonable request.

## Abbreviations

POCUS: Point-of-care ultrasound
ACGME: American Council of Graduate Medical Education
EPSS: E-point septal separation
FS: Fractional shortening
VTi: Velocity time integral
AFV: Aortic flow variability
FAST: Focused assessment with sonography in trauma
ACEP: American College of Emergency Physicians
SCCM: Society of Critical Care Medicine
OSCE: Objective structural clinical examination
SDOT: Standardized direct observation assessment tool
