# Supplementary material for: A point-of-care ultrasound education curriculum for pediatric critical care medicine
Source: Ultrasound J. 2022 Oct 31;14:44. doi: 10.1186/s13089-022-00290-6 (PMC9622960; doi:10.1186/s13089-022-00290-6)
Supplement: Supplementary file 1 — Additional file 1. Needs assessment for learners of the point-of-care ultrasound curriculum [file 13089_2022_290_MOESM1_ESM.docx]

Q1 Please write your initials and year of birth. (ex: JD1987 for John Doe year of birth 1987)

- ________________________________________________

Q2 What is your current diagnostic ultrasound experience?

On the job experience

No formal experience

Extensive experience through clinical practice and external/internal coursework

Q3 How comfortable do you feel in your diagnostic point of care ultrasound skills?

Extremely comfortable

Moderately comfortable

Slightly comfortable

Neither comfortable nor uncomfortable

Slightly uncomfortable

Moderately uncomfortable

Extremely uncomfortable

Q4 What is your current procedural ultrasound experience?

On the job experience

No formal experience

Extensive experience through clinical practice and external/internal coursework

Q5 How comfortable do you feel in your procedural point of care ultrasound skills?

Extremely comfortable

Moderately comfortable

Slightly comfortable

Neither comfortable nor uncomfortable

Slightly uncomfortable

Moderately uncomfortable

Extremely uncomfortable

Q6 Do you know how to operate your current point of care ultrasound machine? (save & delete images/clips, review older studies, save patient data)

Yes

No
