# Supplementary material for: A point-of-care ultrasound education curriculum for pediatric critical care medicine
Source: Ultrasound J. 2022 Oct 31;14:44. doi: 10.1186/s13089-022-00290-6 (PMC9622960; doi:10.1186/s13089-022-00290-6)
Supplement: Supplementary file 2 — Additional file 2. Pre-course knowledge assessment of the learners [file 13089_2022_290_MOESM2_ESM.docx]

SECTION 1 – GEN INFO:

Q1 Please write your initials and year of birth. (ex: JD1987 for John Doe year of birth 1987)

- ________________________________________________

Q2 What is your current diagnostic ultrasound experience?

On the job experience

No formal experience

Extensive experience through clinical practice and external/internal coursework

Q3 How comfortable do you feel in your diagnostic point of care ultrasound skills?

Extremely comfortable

Moderately comfortable

Slightly comfortable

Neither comfortable nor uncomfortable

Slightly uncomfortable

Moderately uncomfortable

Extremely uncomfortable

Q4 What is your current procedural ultrasound experience?

On the job experience

No formal experience

Extensive experience through clinical practice and external/internal coursework

Q5 How comfortable do you feel in your procedural point of care ultrasound skills?

Extremely comfortable

Moderately comfortable

Slightly comfortable

Neither comfortable nor uncomfortable

Slightly uncomfortable

Moderately uncomfortable

Extremely uncomfortable

Q6 Do you know how to operate your current point of care ultrasound machine? (save & delete images/clips, review older studies, save patient data)

Yes

No

SECTION 2: KNOBOLOGY & US PHYSICS

Q7 What is the frequency of ultrasound waves used in modern day diagnostic ultrasound?

< 20 Hz

20 Hz - < 1 MHz

1 MHz - 25 MHz

______________

Q8 An object that appears bright on the ultrasound screen is known as?

Anechoic

Hyperechoic

Isoechoic

None of the above

Q9 The ultrasound waves lose energy as they pass through the tissue. Keeping this in mind which of the following is true?

The high frequency probe (linear probe) loses more energy and travel a shorter distance.

The high frequency probe (linear probe) loses less energy and travel a longer distance.

The low frequency probe (phased array) loses less energy and travel a shorter distance.

The distance traveled by the probe is irrespective of the frequency of sound emitted by the probe.

Q10 While using color doppler which of the following conventions is correct?


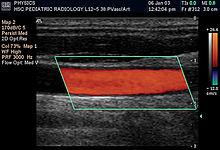


Blue color is always venous and red color is always arterial flow

Blue is blood moving away from the probe and red is blood flow towards the probe

The color depends on the velocity of flow

Regurgitant jets are red in color and forward flow is blue in color

Q11 The image shown below demonstrates which two artefacts?


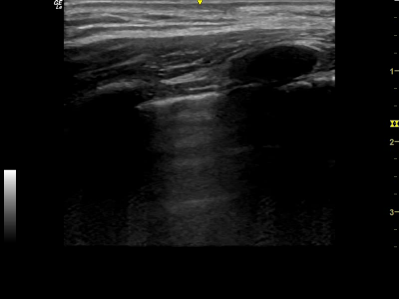


Posterior acoustic shadowing

Posterior acoustic enhancement

Reverberation artefact

Mirror image

SECTION 3: VASCULAR ACCESS

Q12 Which of the following explains the rationale for using ultrasound guidance in obtaining vascular access?

It decreases overall success and increases mean number of attempts per successful access

It increases the first stick and overall success associated with obtaining vascular access and decreases the mean number of attempts needed for successful access

Vascular access obtained with US guidance lasts longer

It increases the complications associated with central line placement

Q13 The following technique of obtaining vascular access is known as?


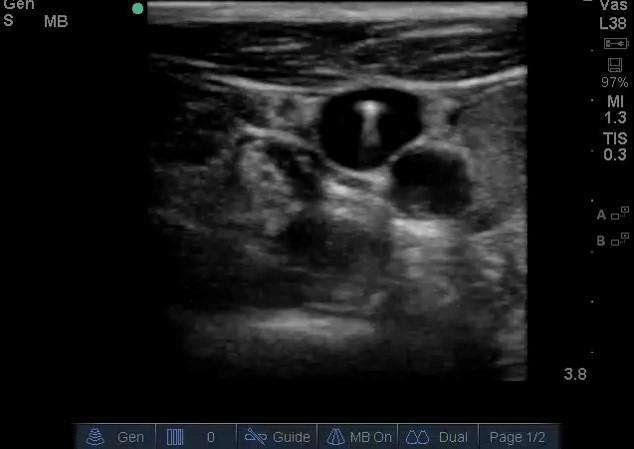


In plane approach

Short axis approach

Long axis approach

Oblique approach

Q14 Which of the following statements is true regarding in plane axis?

It is also knows as the short axis approach

It allows complete visualization of the needle

It is much more forgiving for a novice user and allows lateral side to side movements

Both A & B

Q15 The angle of insonation is the angle maintained between the ultrasound probe and the needle tip (other structures of interest) to obtain a clear image. This angle is typically maintained at:

30 degree
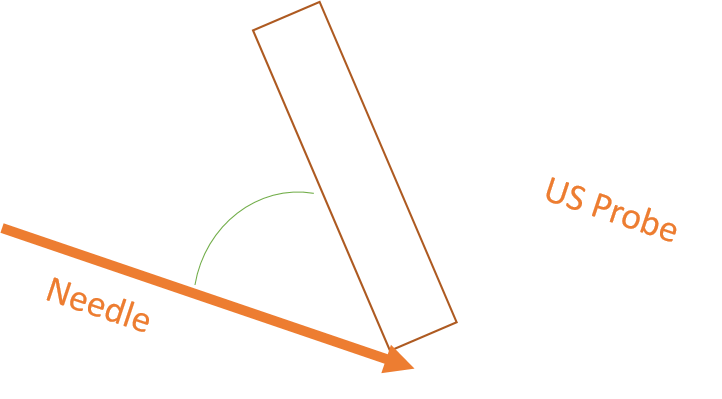


45 degree
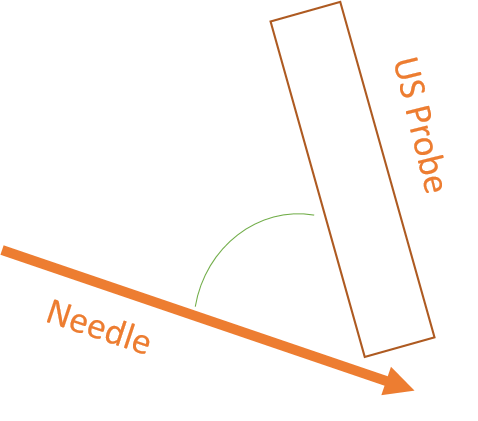


60 degree
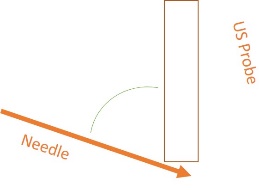


90 degree
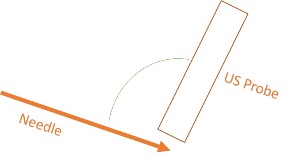


Q16 Which of the following methods can be used to confirm central line placement?

Confirmation of wire in the vessel confirmed on long & short axis

Confirmation of wire in the RA using a subxiphoid approach

Administration of agitated saline through the central line and visualizing in RA/RV

All of the above

None of the above

SECTION 4: ECHO

Q17 Identify the correct FATE view and the position of the probe: (still image only)

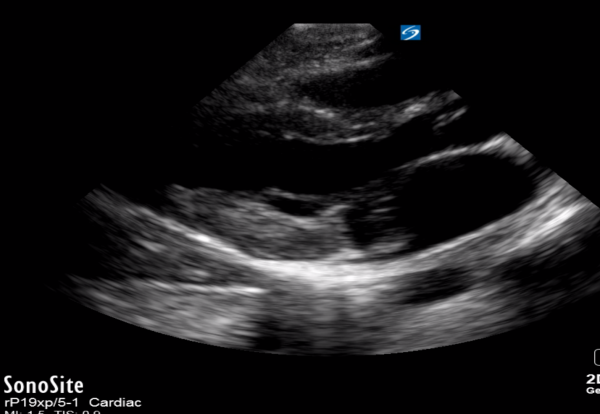


Parasternal long axis; probe placed 2nd - 3rd ICS with probe marker towards patient's right shoulder

Parasternal short axis; probe placed 2nd - 3rd ICS with probe marker towards patient's right shoulder

Parasternal long axis; probe placed 2nd - 3rd ICS with probe marker towards patient's left shoulder

Parasternal short axis; probe placed 2nd - 3rd ICS with probe marker towards patient's left shoulder

Q18 Identify the correct FATE view and the highlighted structure: (still image only)


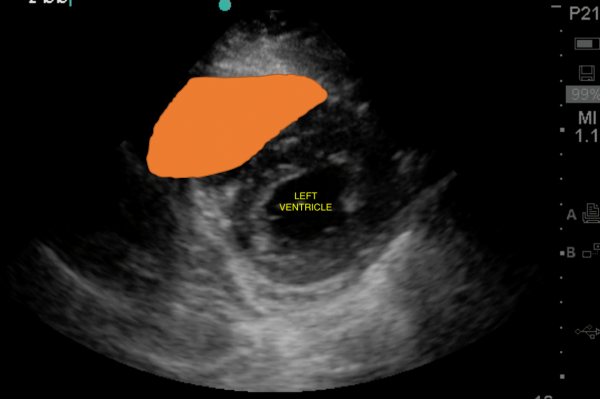


Parasternal long - Right ventricle

Parasternal short - Right ventricle

Apical 2 chamber - Right ventricle

None of the above

Q19 Identify the FATE view and the correct interpretation: (Please use the YouTube link)

<https://youtu.be/u0Itaajbk6k>

Parasternal long; decreased contractility

Apical 4 chamber; hyperdynamic function

Parasternal short; normal function

Parasternal long; hyperdynamic function

Q20 Identify the correct FATE view and the diagnosis: (Please use the YouTube link)

<https://youtu.be/X3uX3pdX5pA>

Parasternal short; decreased contractility

Parasternal short; RV dilation/hypertrophy

Parasternal long; decreased contractility

Apical 2 chamber: decreased contractility

Q21 Identify the correct FATE view and the diagnosis: (Please use the YouTube link)

<https://youtu.be/FIn0nVDv_wk>

Subxiphoid view: Normal

Subxiphoid view: pericardial effusion and RV scalloping

Apical 4 chamber view: Pericardial effusion and RV scalloping

Apical 4 chamber view: Normal

SECTION 5: HEMODYNAMIC ASSESSMENT

Q22 Static and dynamic measures of volume assessment may be used to predict volume responsiveness in a patient. IVC diameter and collapsibility represent which of the two metrics?

Static measure of volume assessment

Dynamic measure of volume assessment

None of the above

Q23 Which of the following clips will predict the need for on going fluid resuscitation in case of shock/hypotension in a spontaneously breathing septic patient without evidence of myocardial dysfunction?

(1) <https://youtu.be/xa44xd7K2r4>

(2) <https://youtu.be/qOrQcqSSM8Y>

Q24 Which of the following ultrasonographic measures has consistently demonstrated benefit in predicting volume responsiveness?

IVC collapsibility

IVC/Ao ratio

Aortic flow variability

IVC compressibility index

Q25 The following images represent which modality of volume assessment?


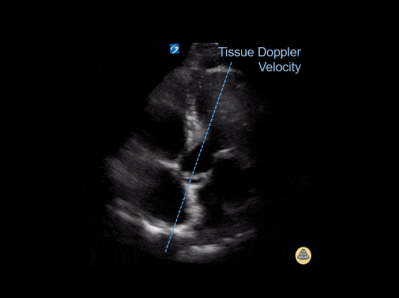


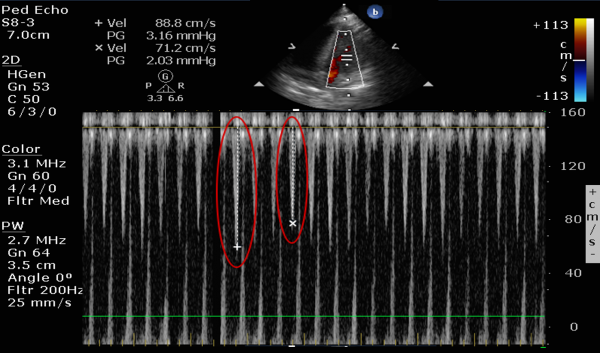


Pulse pressure variability

Aortic flow - Peak velocity variability

M mode - IVC compressibility

VTI - Velocity time integral

Q26 The use of IVC collapsibility as an assessment tool for volume status may not be useful in:

Patient's on positive pressure ventilatio

Patient with right sided heart failure

Patient with intrabdominal hypertension

All of the above

SECTION 6: LUNGS

Q27 The lungs are air filled structures. Air is a strong reflector of ultrasound waves. The appearance of air on ultrasound is described as:

Anechoic

Hyperechoic

Incorrect statement. Air is a poor reflector of the ultrasound waves

None of the above

Q28 The following image demonstrates which artefact?


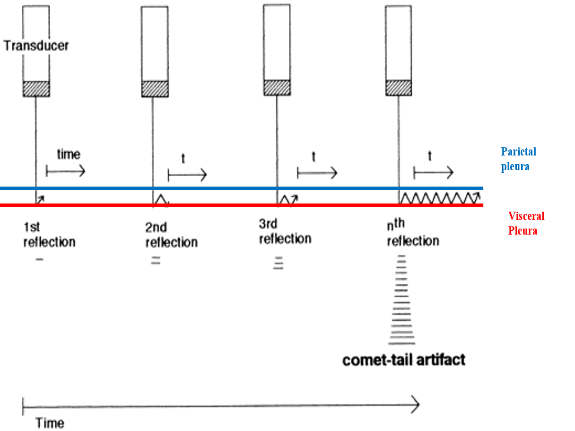


Reverberation artefact

Rarefaction artefact

Posterior acoustic shadowing

Side lobe artefact

Q29 The arrow in the image refers to which lung artefact?


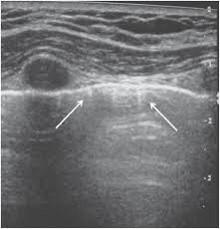


A lines

B lines

Z lines

D lines

Q30 The clip below demonstrates which finding on the lung ultrasound?

<https://youtu.be/n5EXZNJMgwA>

Pleural sliding

Absent pleural sliding

Lung point

Lung pulse

Q31 Which of the following ultrasonographic findings are seen in patients with pneumonia?

Lung hepatization

Air bronchogram

Subpleural consolidation

B lines

Shred sign

All of the above

Q32 Which of the following findings are seen in patients with fluid overload and pulmonary edema?

A lines > 2 present in multiple long zones +/- pleural sliding

Well defined B lines are present in multiple lung zones with absent pleural sliding

Confluent B lines +/- pleural sliding

Well defined B lines are present in multiple lung zones with normal pleural sliding

SECTION 7: FAST & ABDOMEN

Q33 FAST exam in pediatrics has:

High senstivity, low specificity

High sensitivity & specificity

Low sensitivity, high specificity

Low sensitivity and specificity

Q34 Which of the following sites is the most sensitive for the detection of free fluid?

Hepatorenal recess

Tip of the liver

Anterior to the bladder

Posterior to the bladder

Q35 The following image of the kidney depicts which of the following?


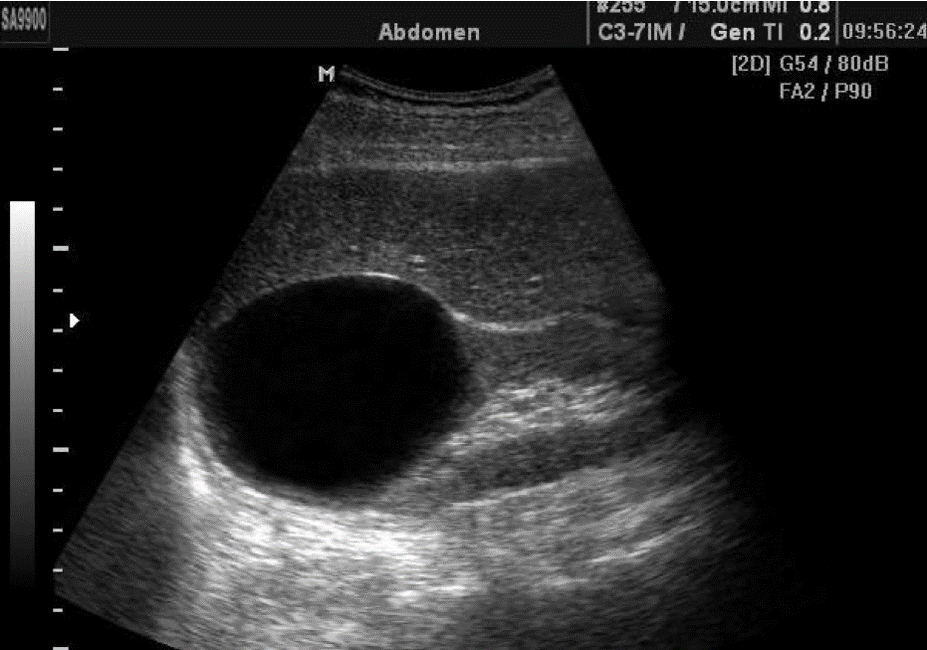


Mild hydropnephrosis

Moderate hydronephrosis

Severe hydronephrosis

Renal cyst

SECTION 8: ADVANCED PROCEDURES

Q36 Which of the following structures may get injured while performing paracentesis?

Epigastric vessels

Bladder

Loops of intestine

All of the above

Q37 Chest ultrasound can be helpful in detecting as little as ___ ml of pleural effusion:

less than 100 ml

100-200 ml

less than 20 ml

more than 200 ml and less than 500 ml

None of the above

Q38 The structure labeled A & B refer to:


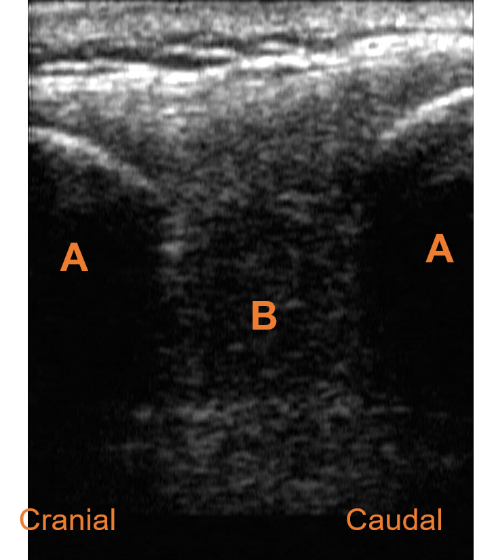


A- Lamina B - Spinous process

A- Interspinous ligament B - Supraspinous ligament

A- Spinous process B - Interspineous ligament

A- Foramen B - Spinal canal

Q39 Which of the following methods are not used routinely in FATE exam to assess contractility:

Qualitative estimation using estimation in multiple views

Fractional shortening (FSS)

End point septal separation (EPSS)

Simpson’s 2 disc method
