# Supplementary material for: A point-of-care ultrasound education curriculum for pediatric critical care medicine
Source: Ultrasound J. 2022 Oct 31;14:44. doi: 10.1186/s13089-022-00290-6 (PMC9622960; doi:10.1186/s13089-022-00290-6)
Supplement: Supplementary file 3 — Additional file 3. The basics of machine operation, image acquisition and optimization and saving an image [file 13089_2022_290_MOESM3_ESM.pptx]

## Slide 1
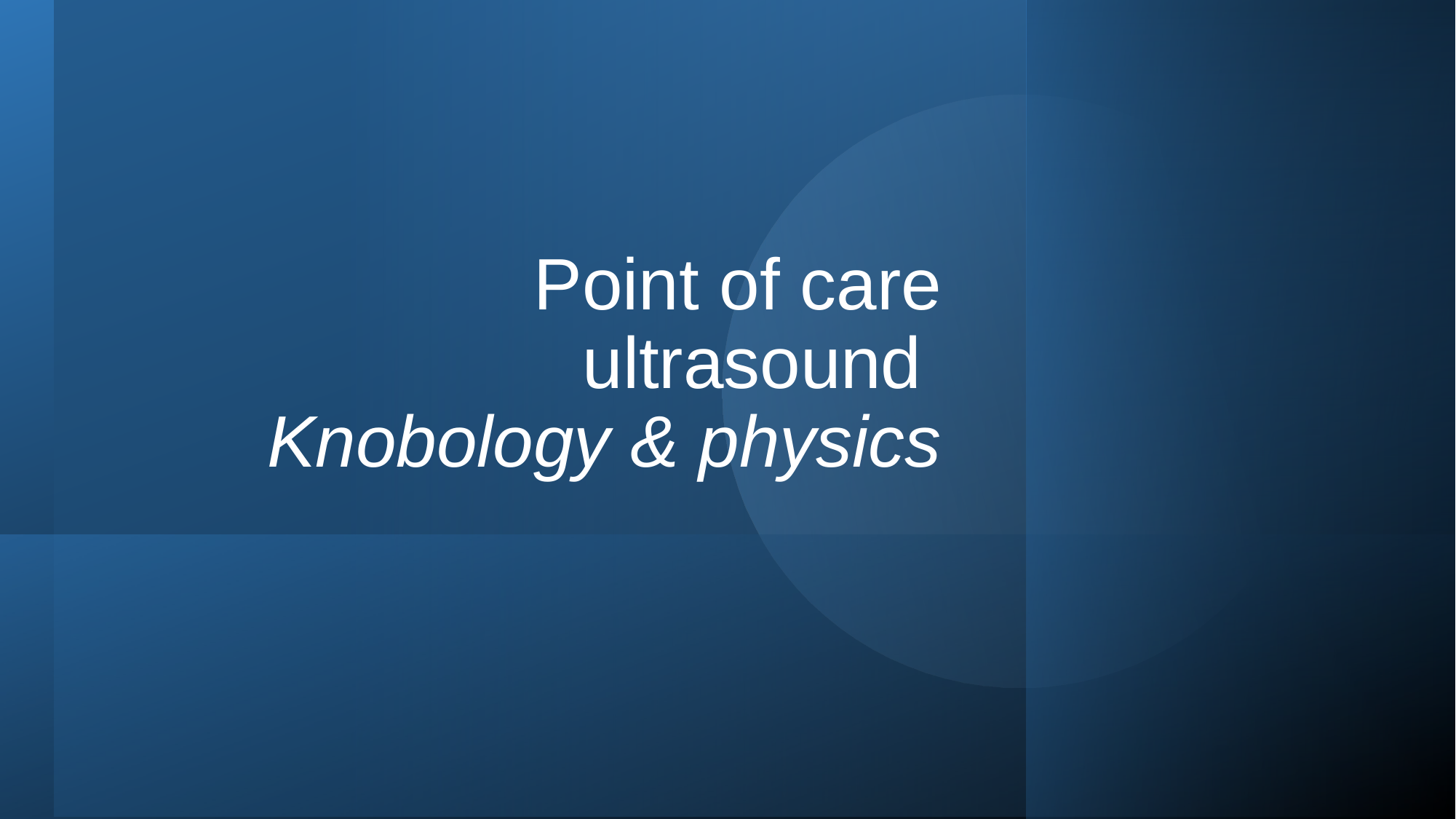

# Point of care ultrasound Knobology & physics

## Slide 2
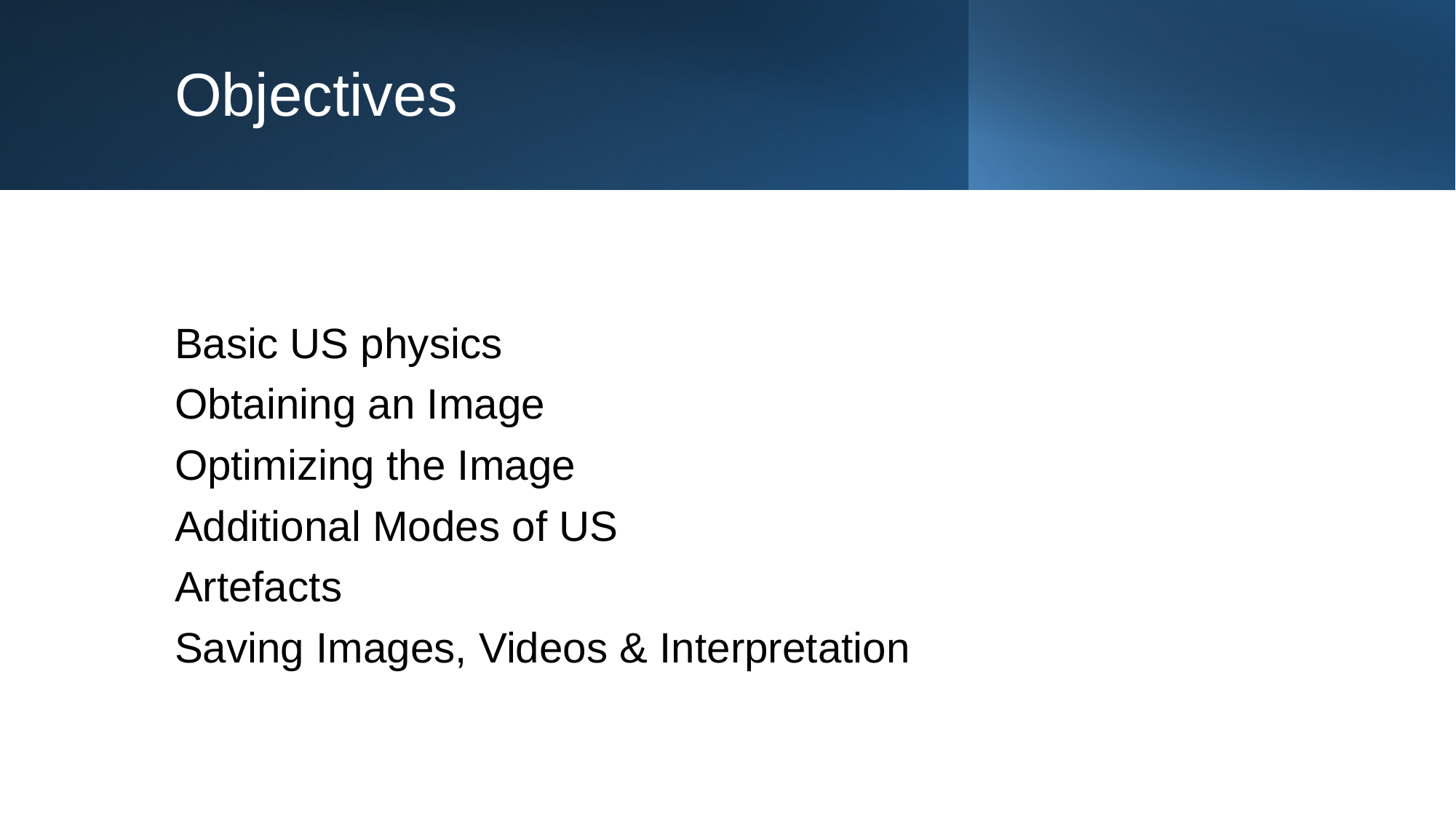

# Objectives
Basic US physics
Obtaining an Image
Optimizing the Image
Additional Modes of US
Artefacts
Saving Images, Videos & Interpretation

## Slide 3
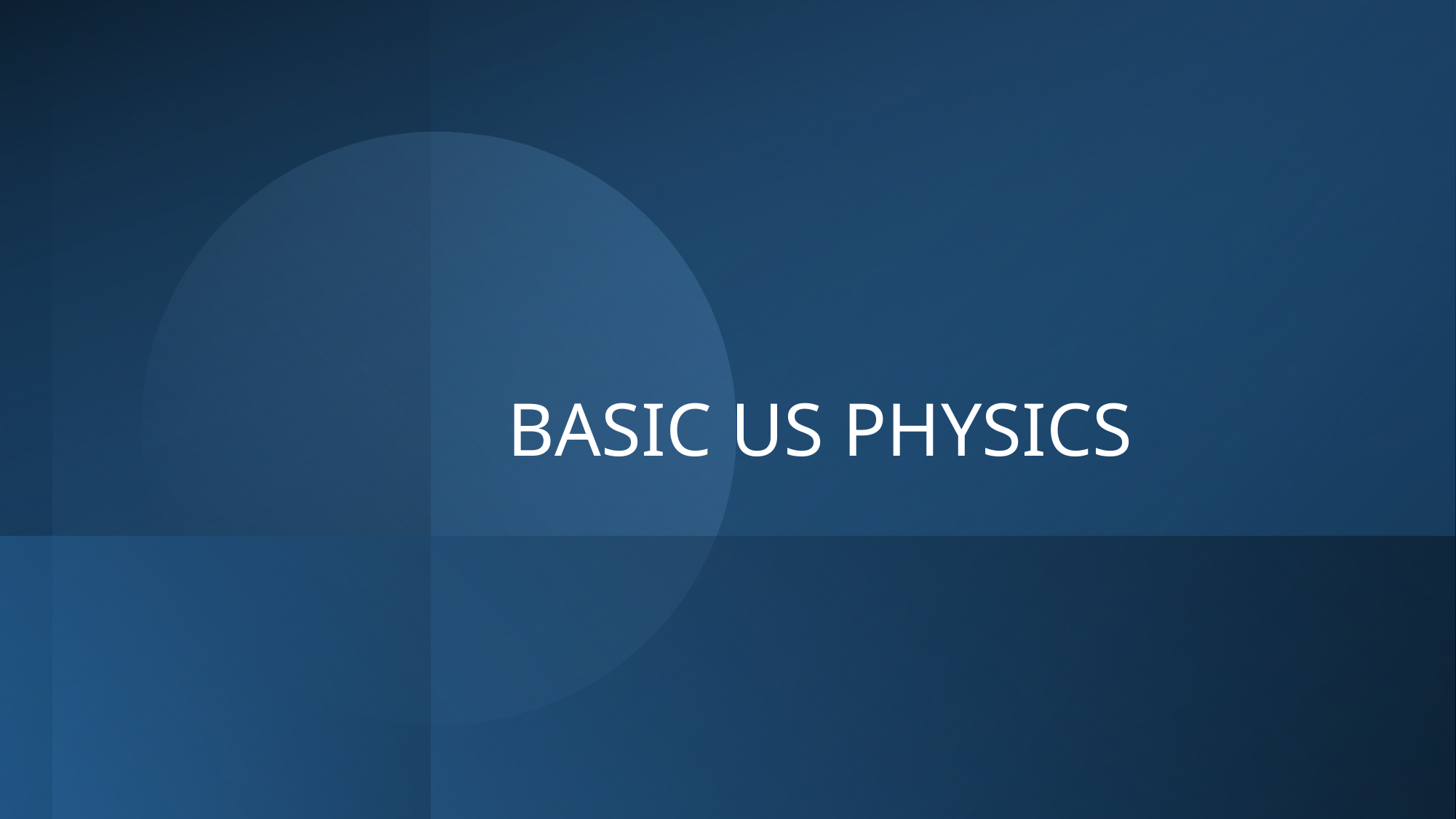

# BASIC US PHYSICS

## Slide 4
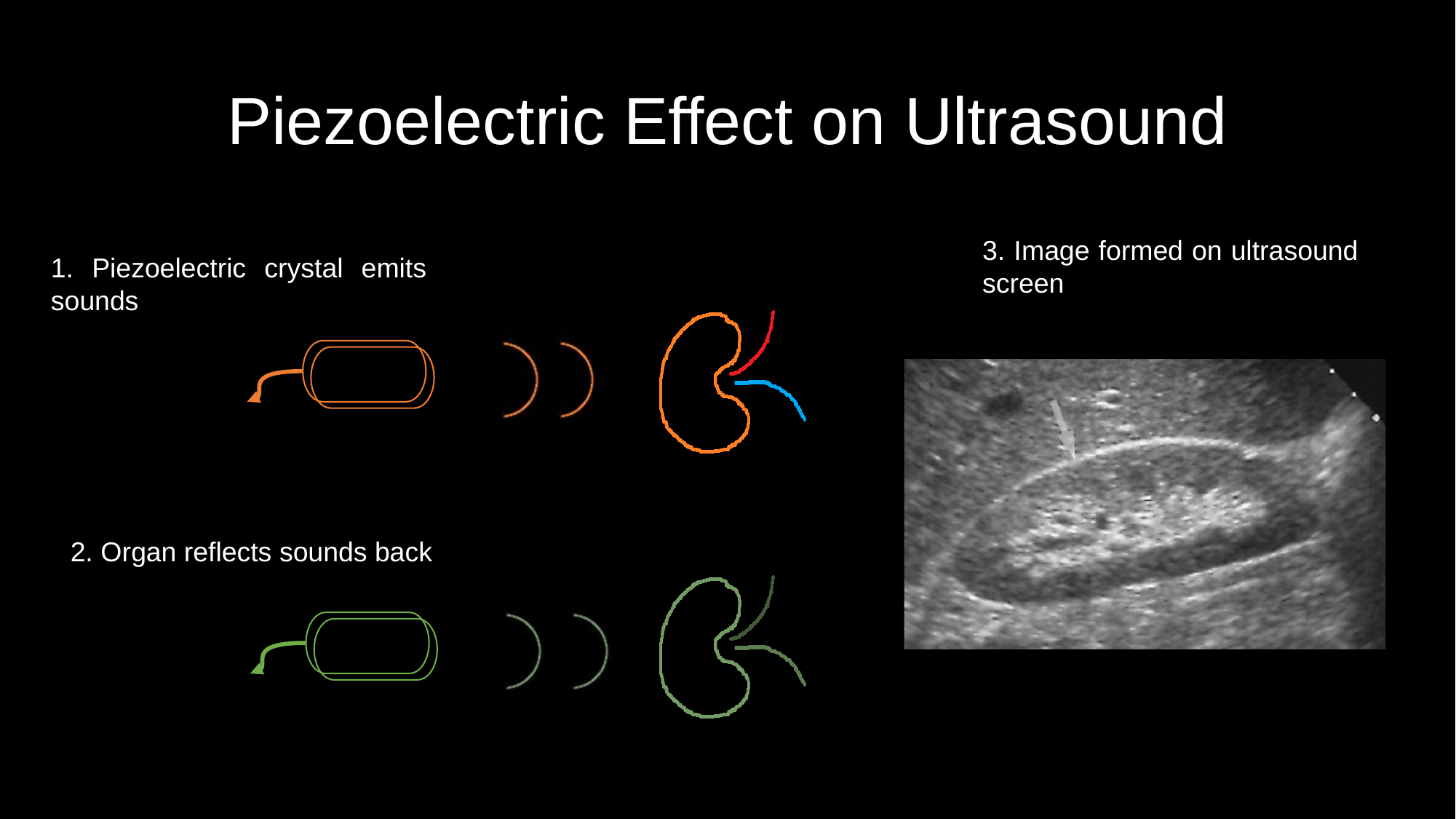

# Piezoelectric Effect on Ultrasound
3. Image formed on ultrasound screen
1. Piezoelectric crystal emits sounds
2. Organ reflects sounds back

## Slide 5
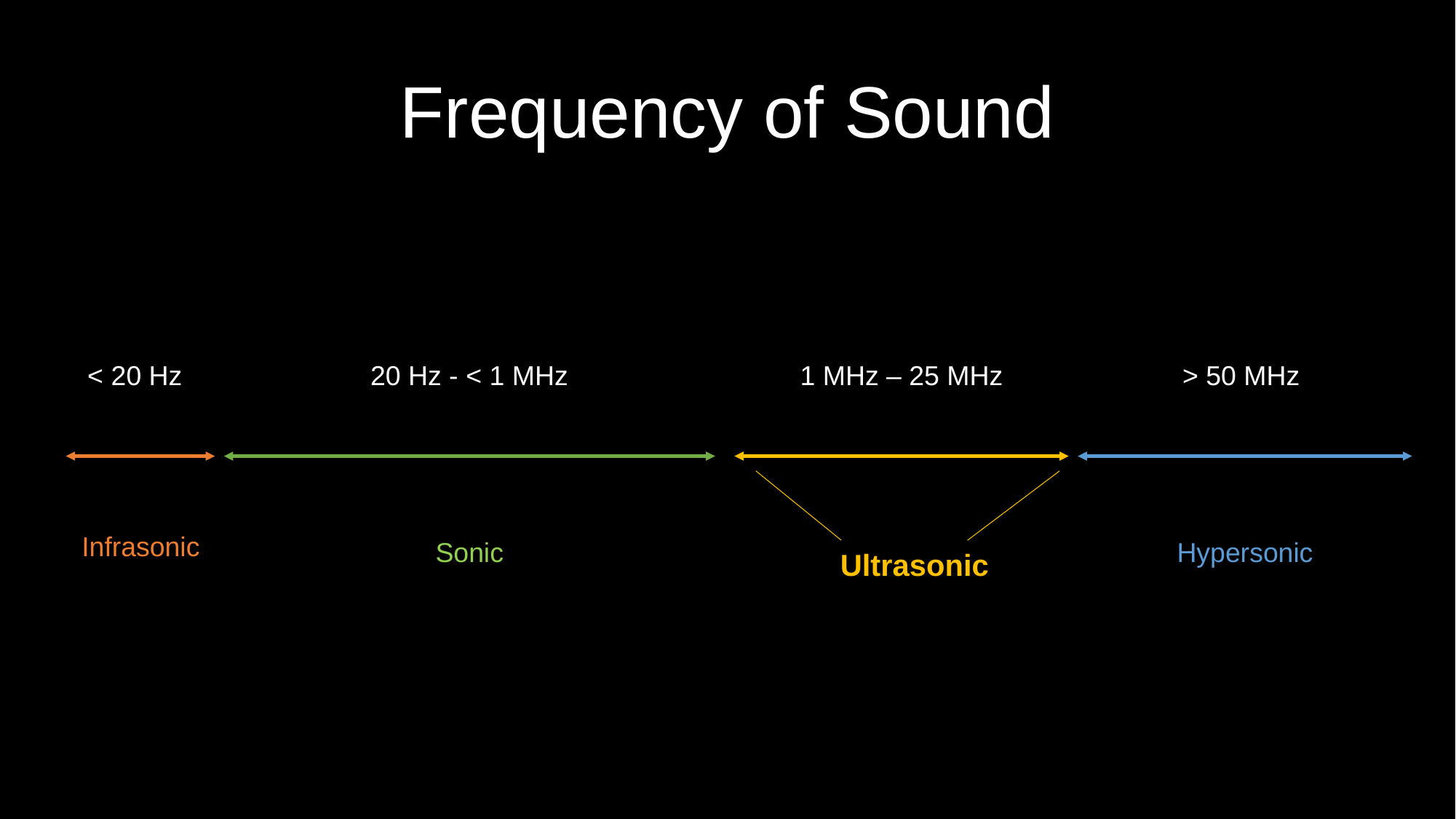

# Frequency of Sound
< 20 Hz
20 Hz - < 1 MHz
1 MHz – 25 MHz
> 50 MHz
Infrasonic
Sonic
Hypersonic
Ultrasonic

## Slide 6
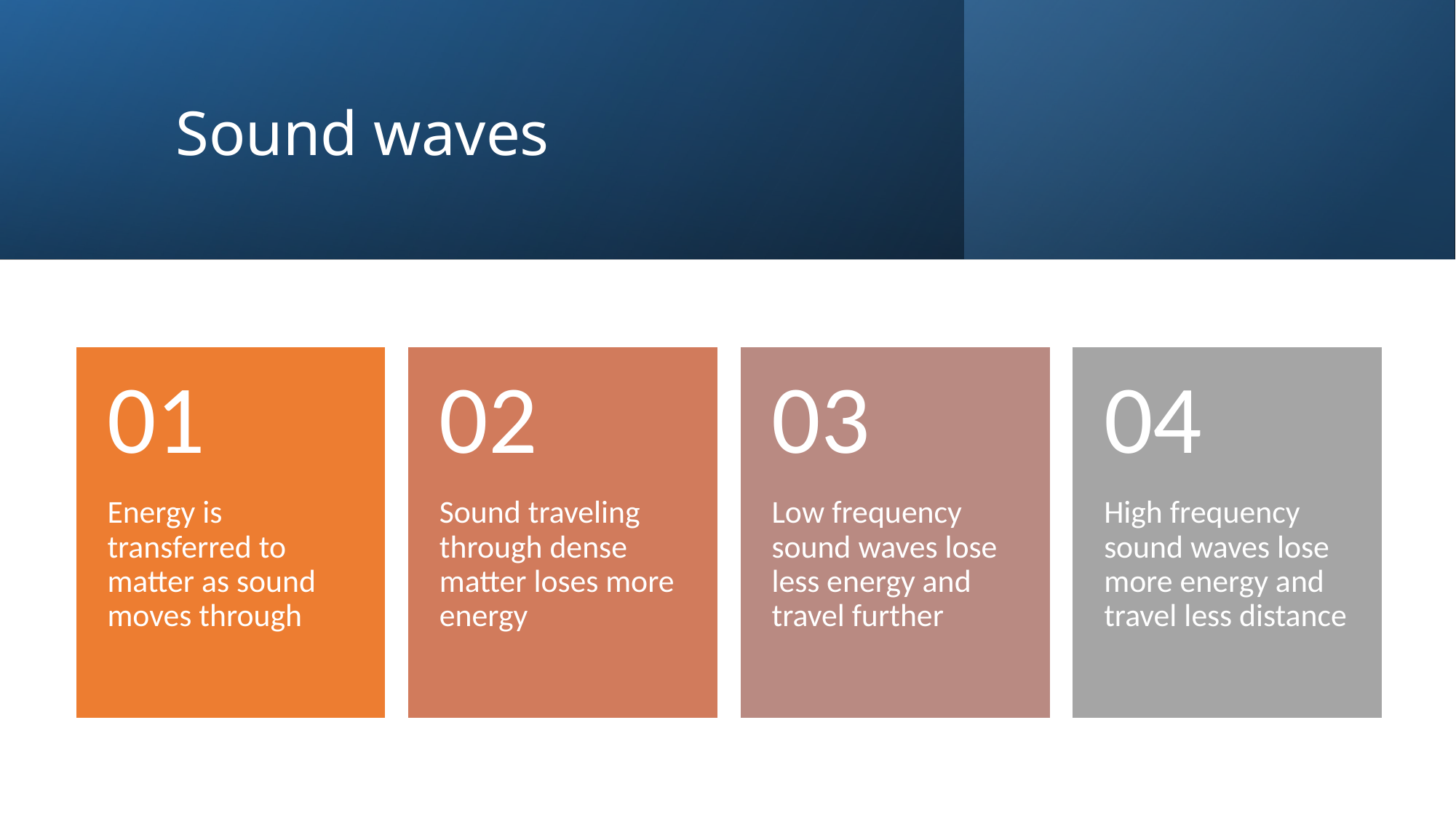

# Sound waves

## Slide 7
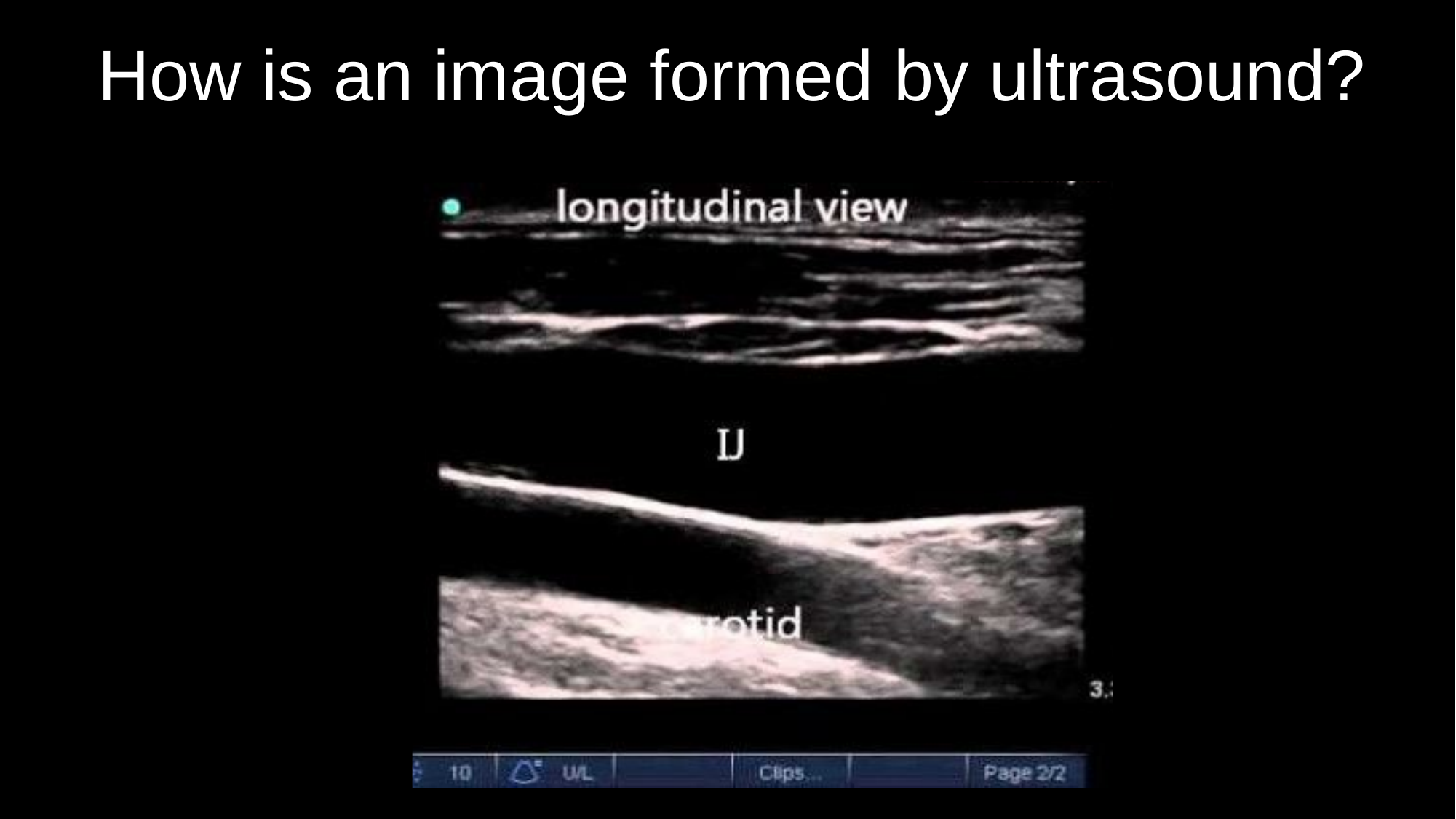

# How is an image formed by ultrasound?

## Slide 8
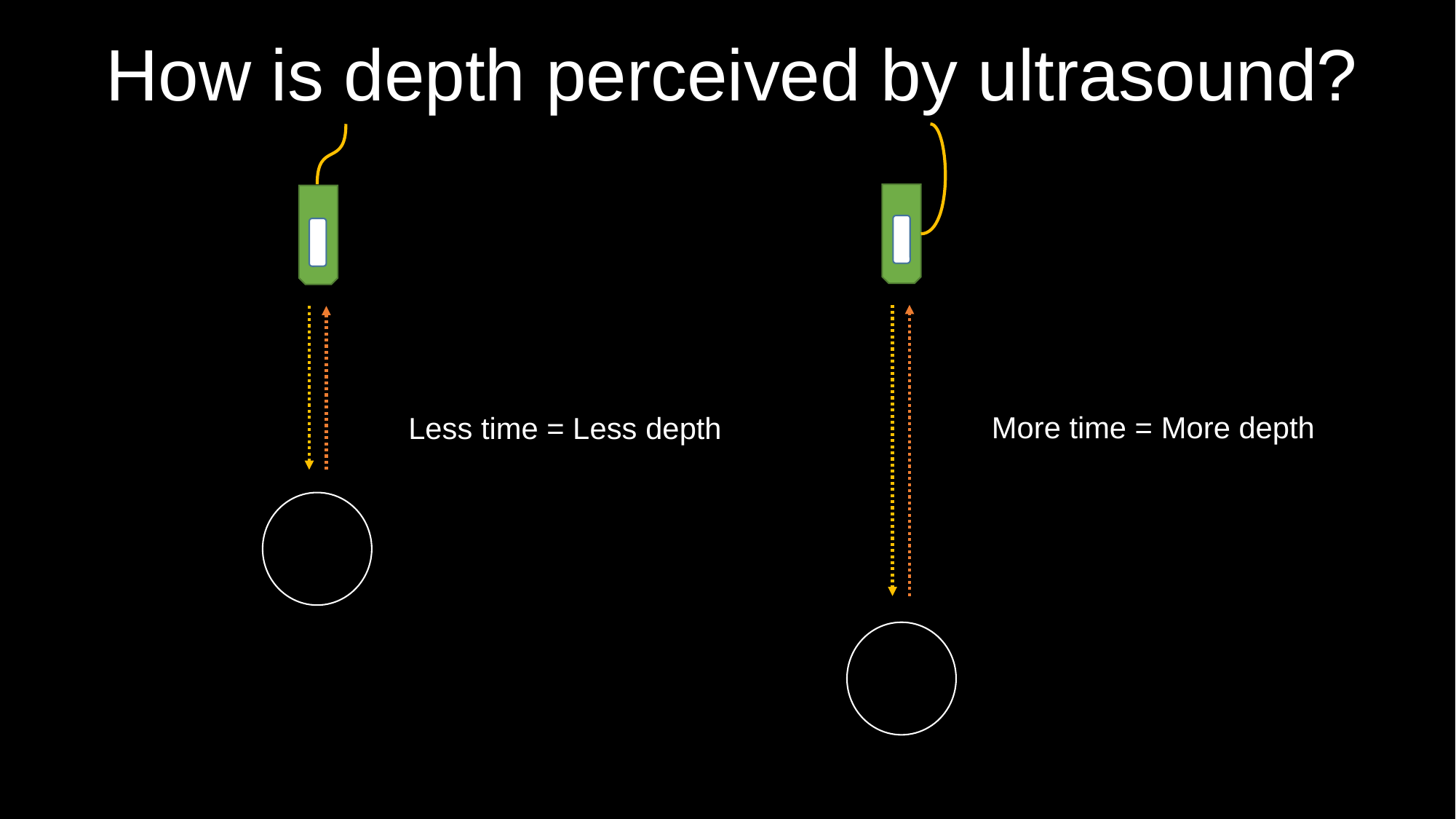

# How is depth perceived by ultrasound?
More time = More depth
Less time = Less depth

## Slide 9
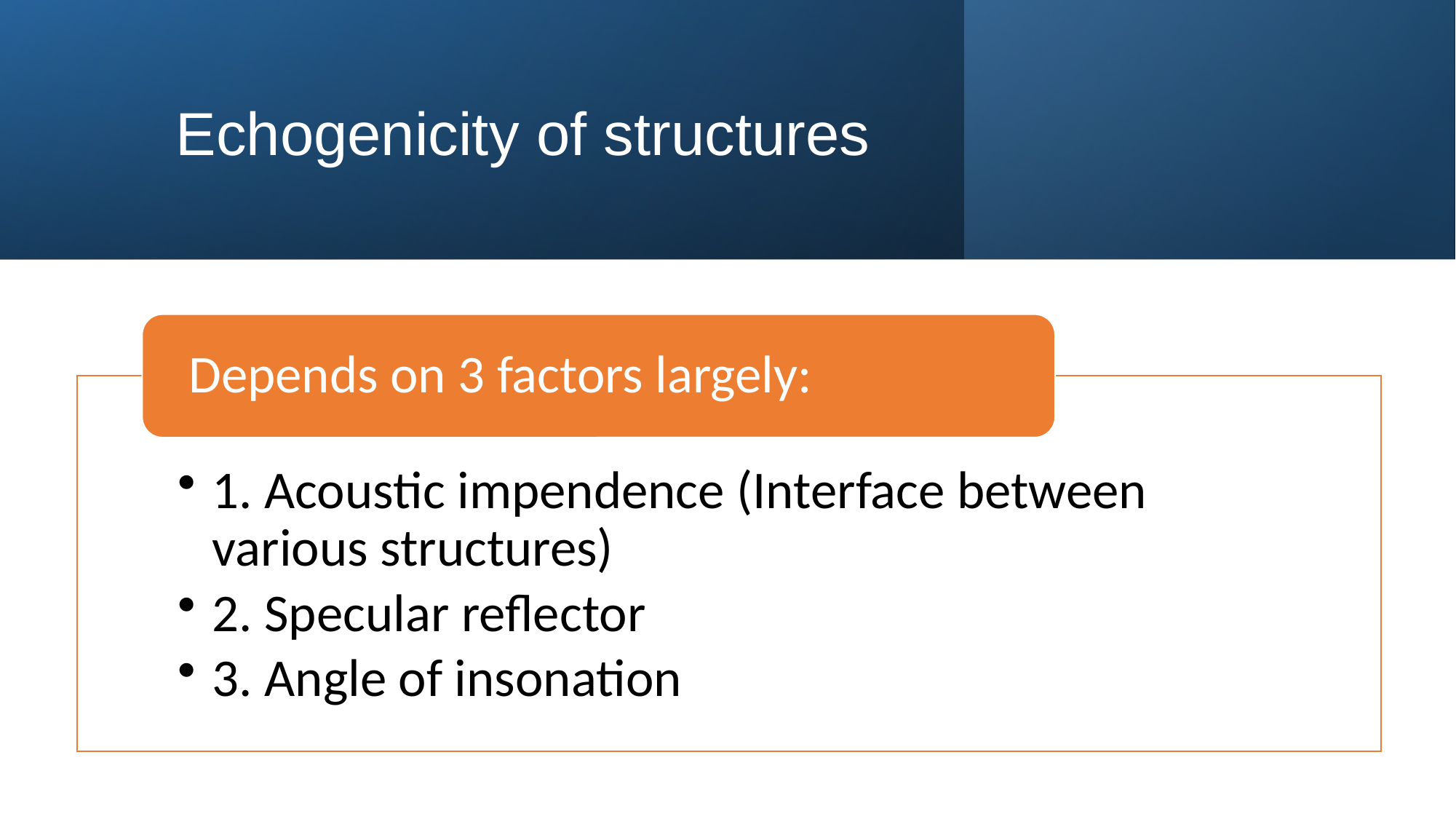

# Echogenicity of structures

## Slide 10
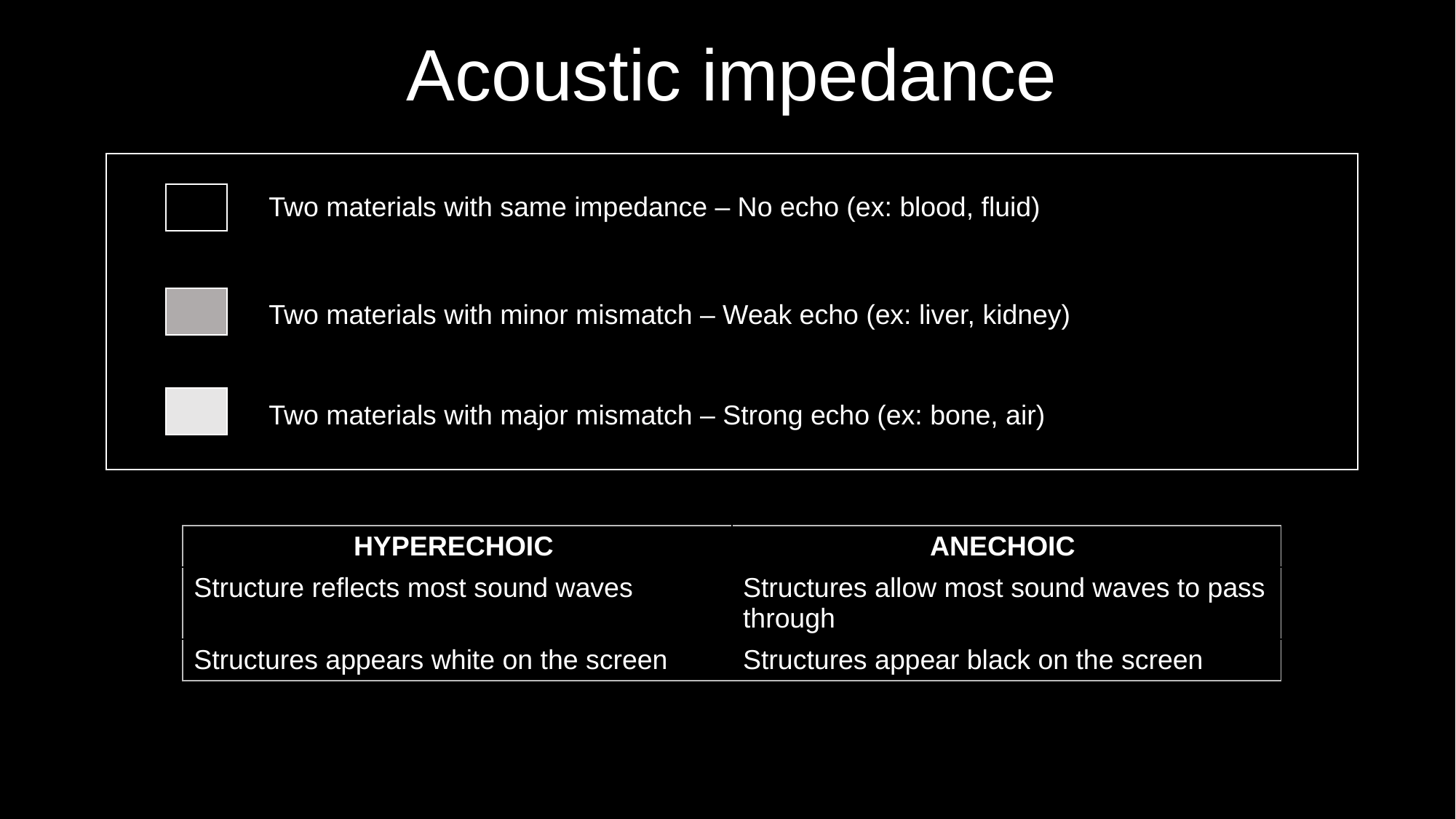

# Acoustic impedance
Two materials with same impedance – No echo (ex: blood, fluid)
Two materials with minor mismatch – Weak echo (ex: liver, kidney)
Two materials with major mismatch – Strong echo (ex: bone, air)
| HYPERECHOIC | ANECHOIC |
| --- | --- |
| Structure reflects most sound waves | Structures allow most sound waves to pass through |
| Structures appears white on the screen | Structures appear black on the screen |

## Slide 11
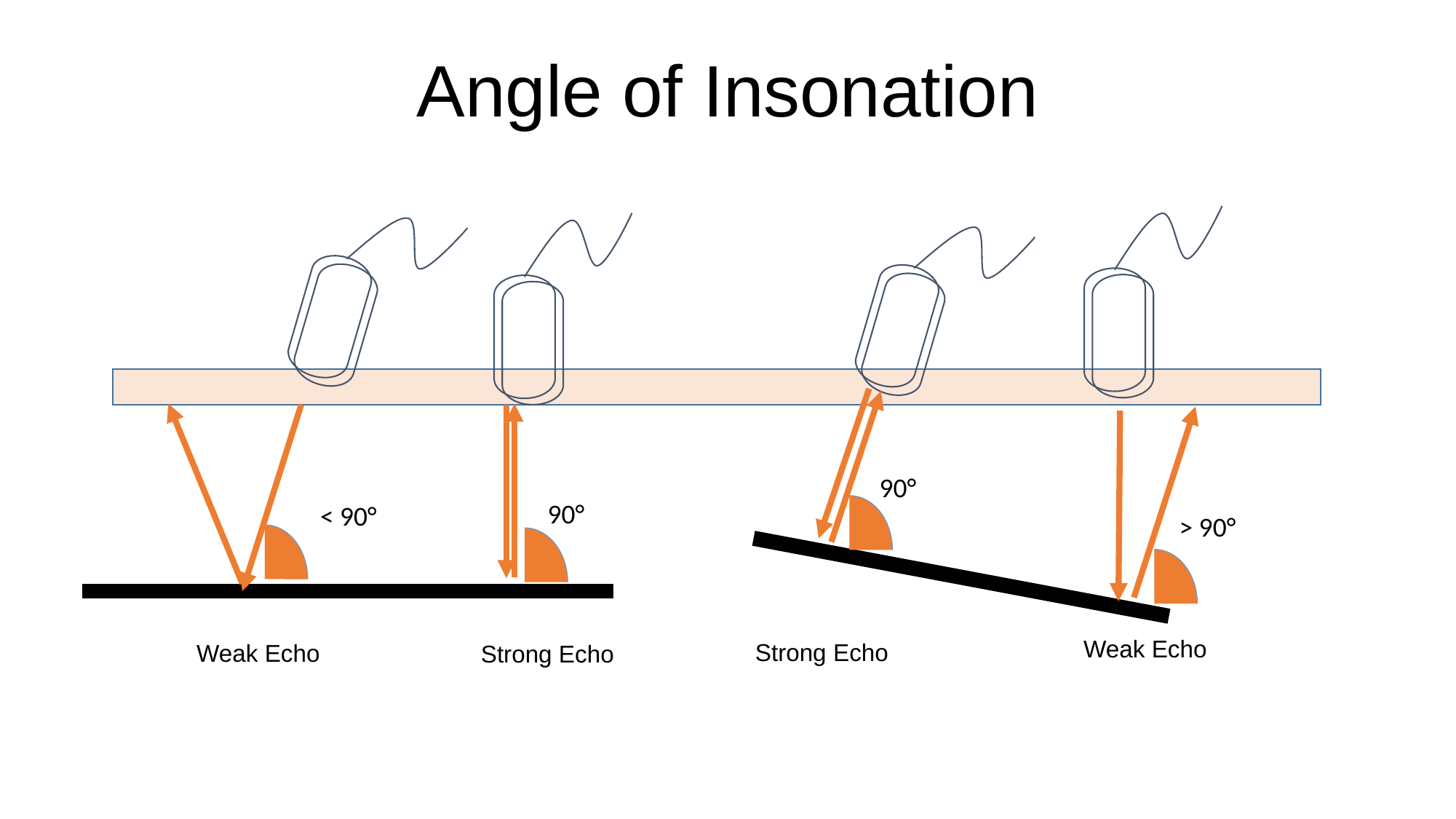

# Angle of Insonation
90°
90°
< 90°
> 90°
Weak Echo
Strong Echo
Weak Echo
Strong Echo

## Slide 12
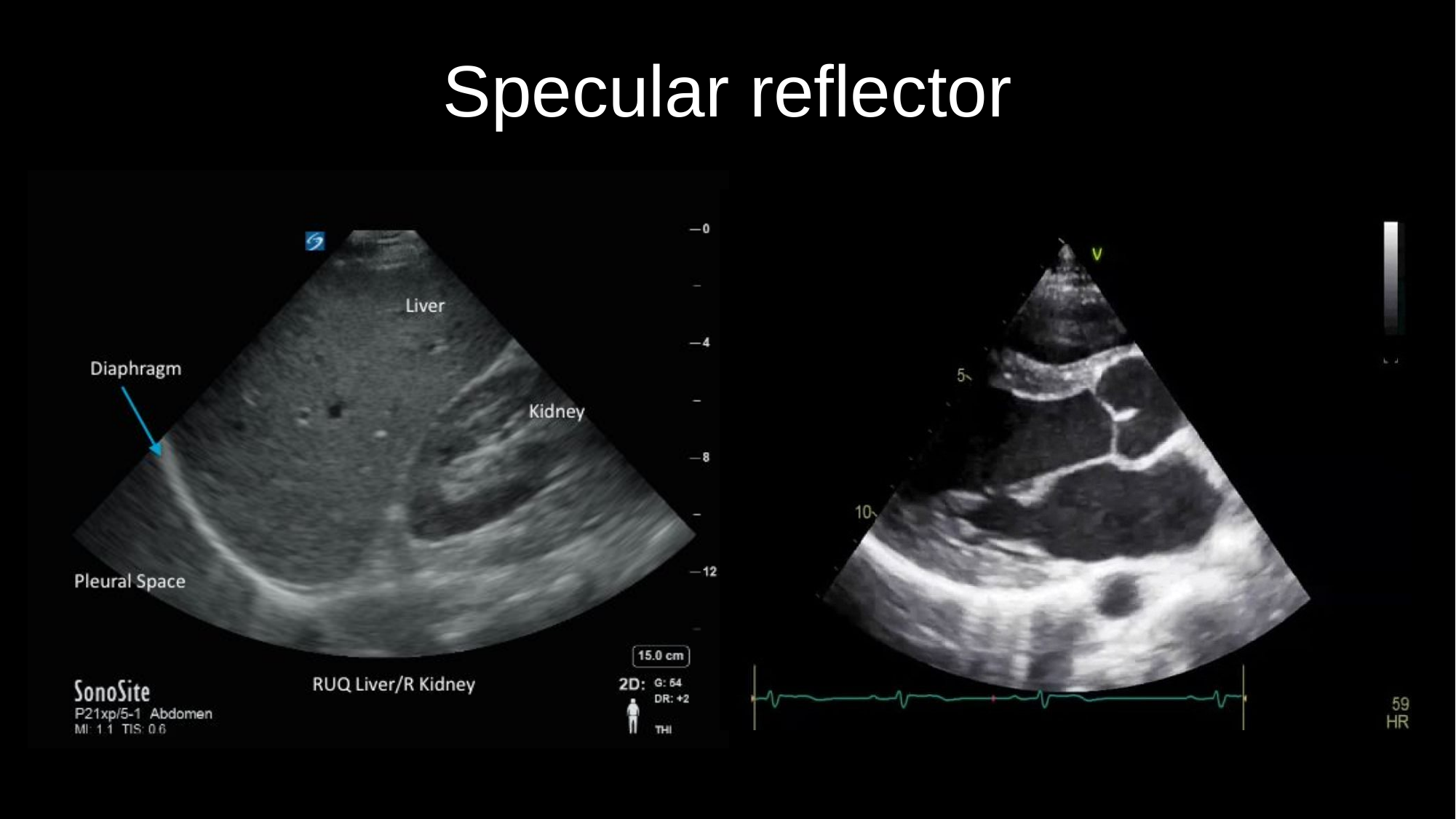

# Specular reflector

## Slide 13
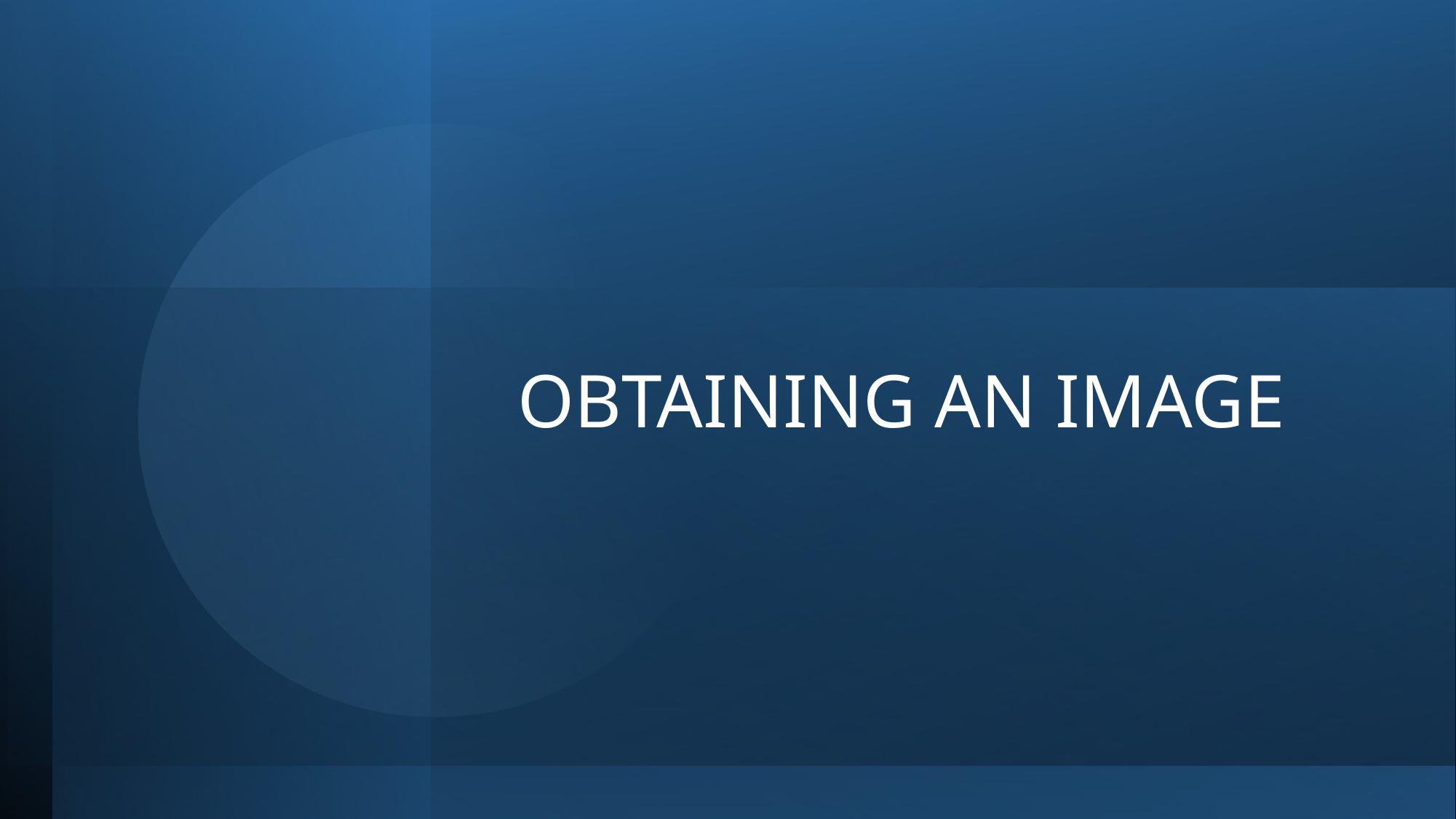

# OBTAINING AN IMAGE

## Slide 14
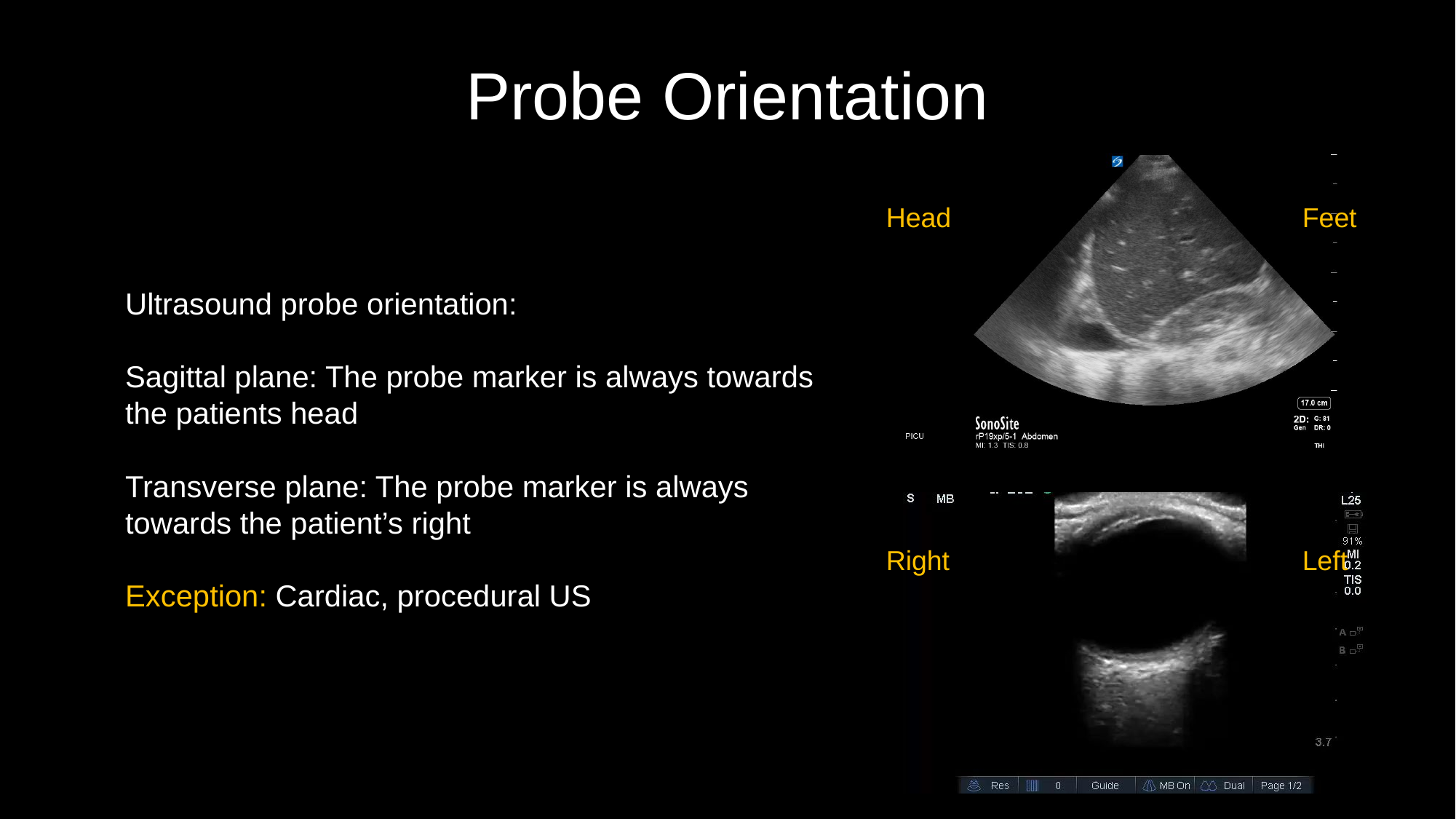

# Probe Orientation
Head
Feet
Ultrasound probe orientation:
Sagittal plane: The probe marker is always towards the patients head
Transverse plane: The probe marker is always towards the patient’s right
Exception: Cardiac, procedural US
Right
Left

## Slide 15
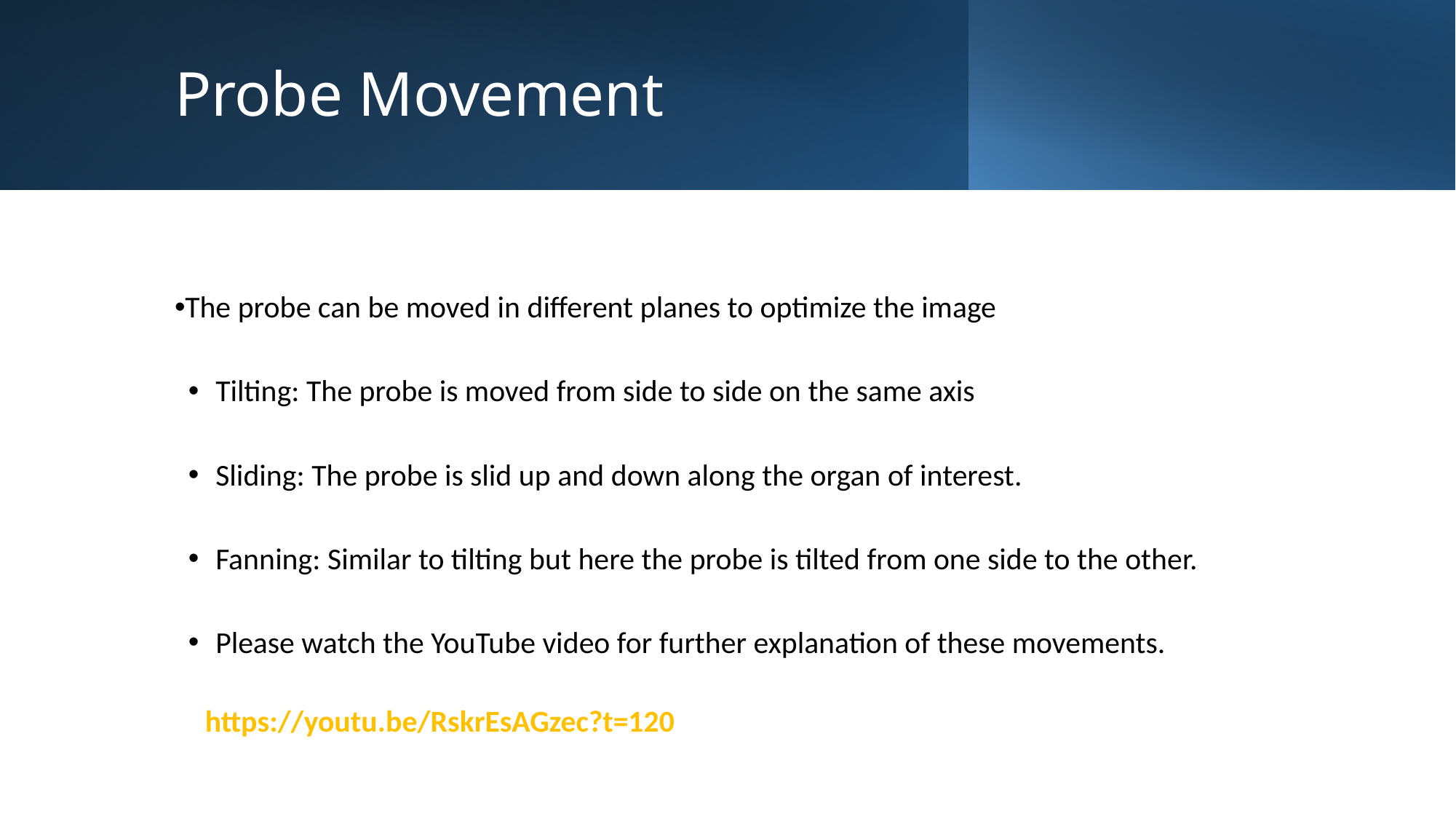

# Probe Movement
The probe can be moved in different planes to optimize the image
Tilting: The probe is moved from side to side on the same axis
Sliding: The probe is slid up and down along the organ of interest.
Fanning: Similar to tilting but here the probe is tilted from one side to the other.
Please watch the YouTube video for further explanation of these movements.
https://youtu.be/RskrEsAGzec?t=120

## Slide 16
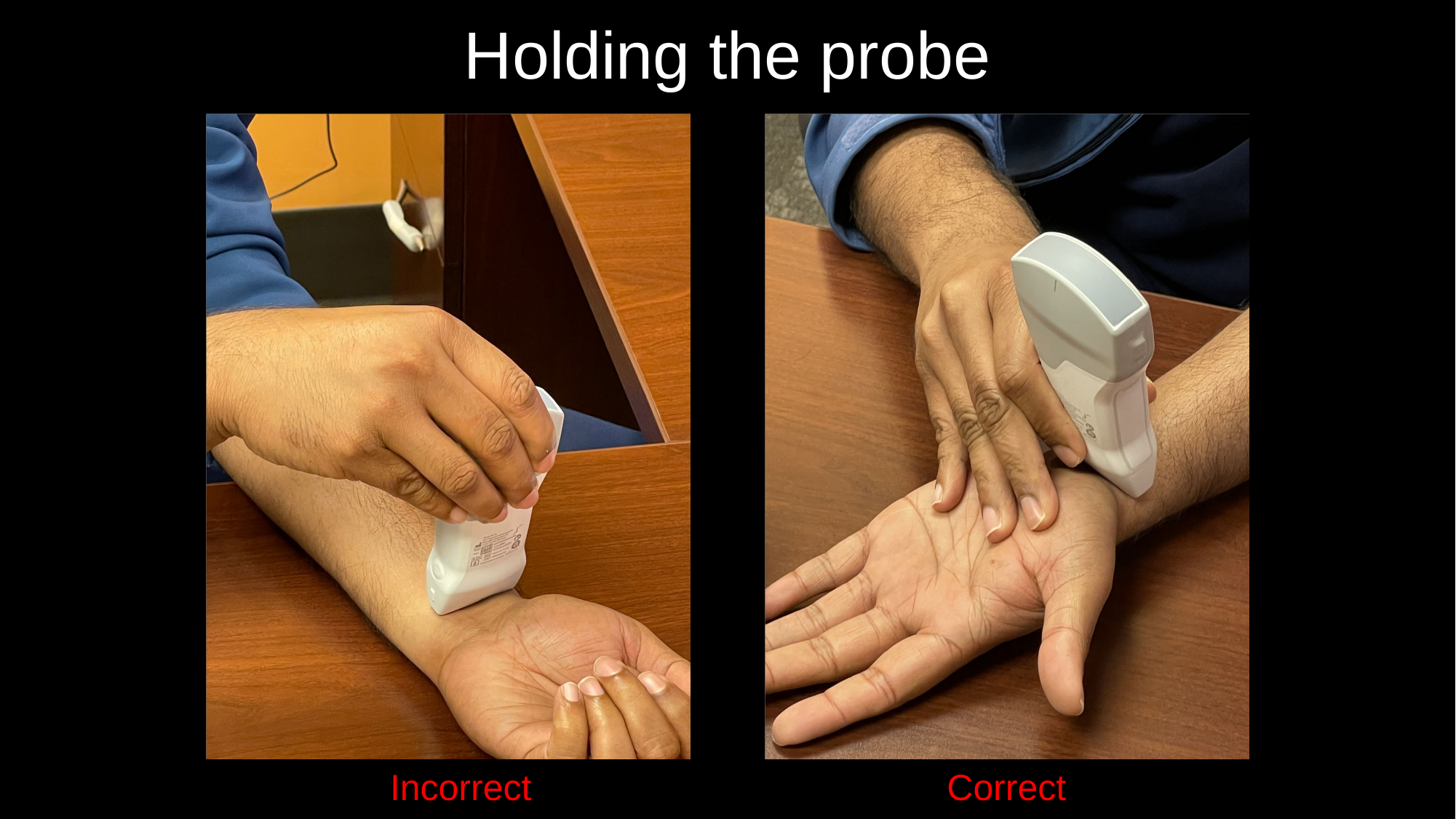

# Holding the probe
Correct
Incorrect

## Slide 17
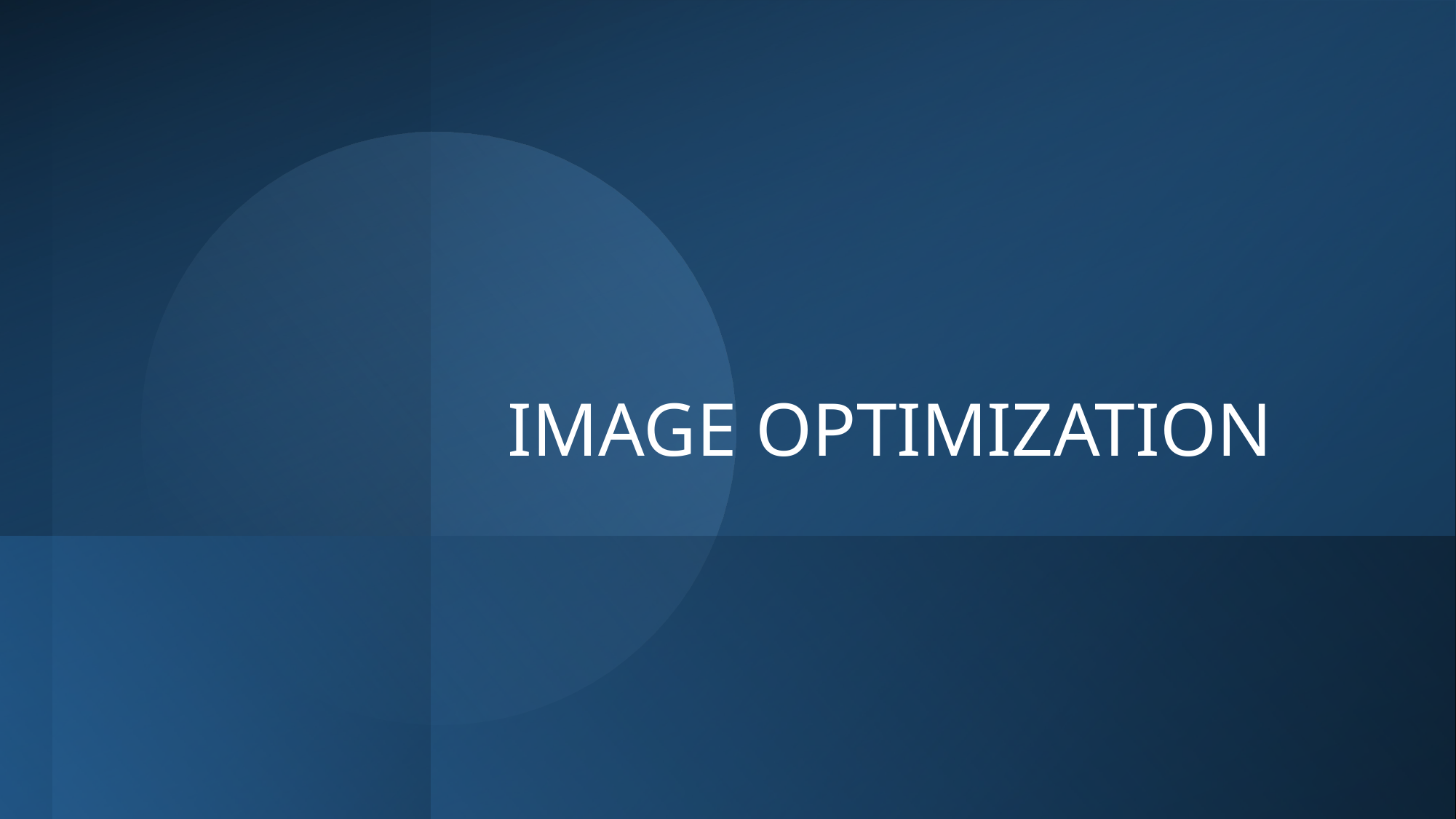

# IMAGE OPTIMIZATION

## Slide 18
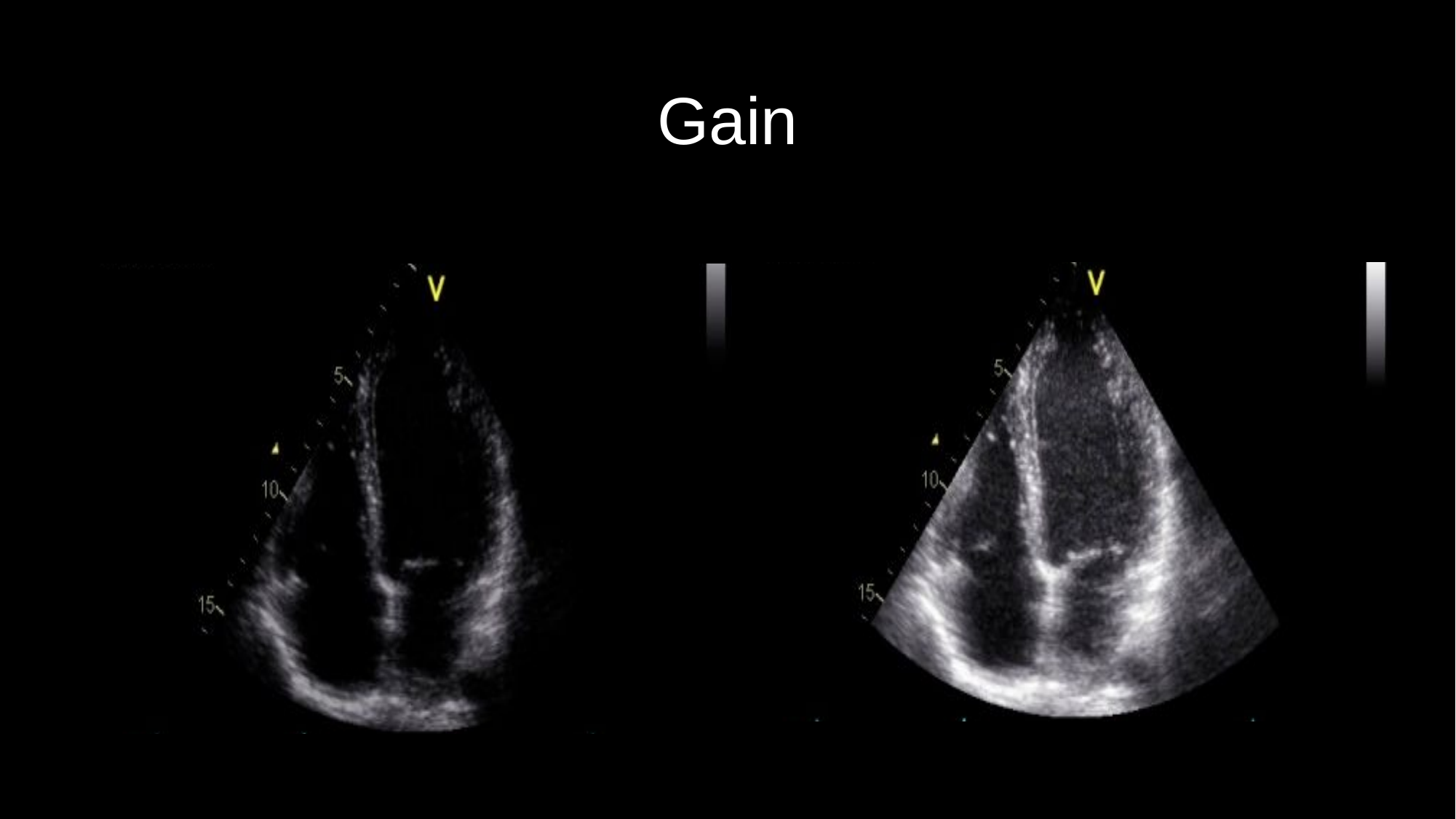

# Gain

## Slide 19
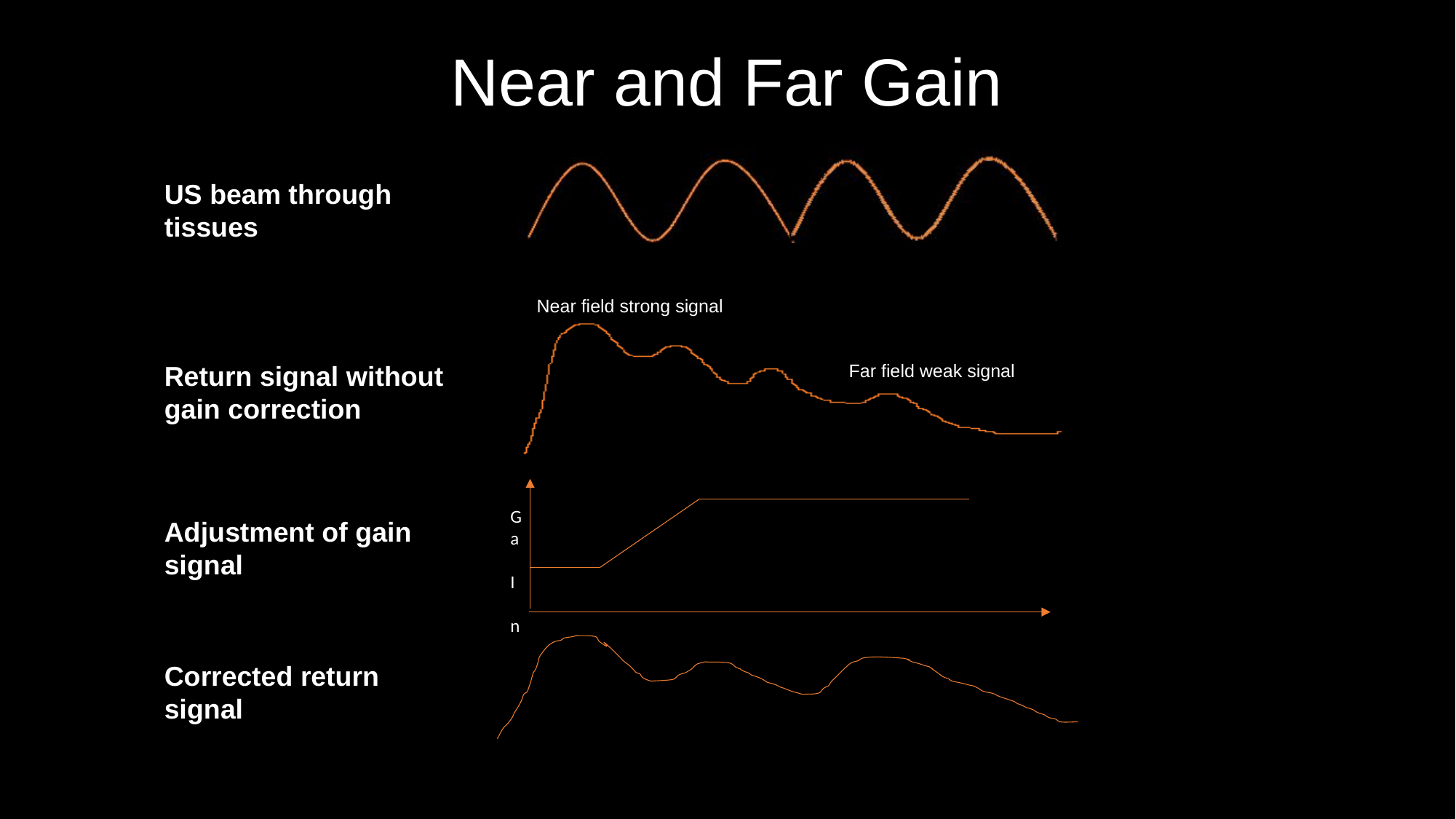

# Near and Far Gain
US beam through tissues
Near field strong signal
Far field weak signal
Return signal without gain correction
Ga I n
Adjustment of gain signal
Corrected return signal

## Slide 20
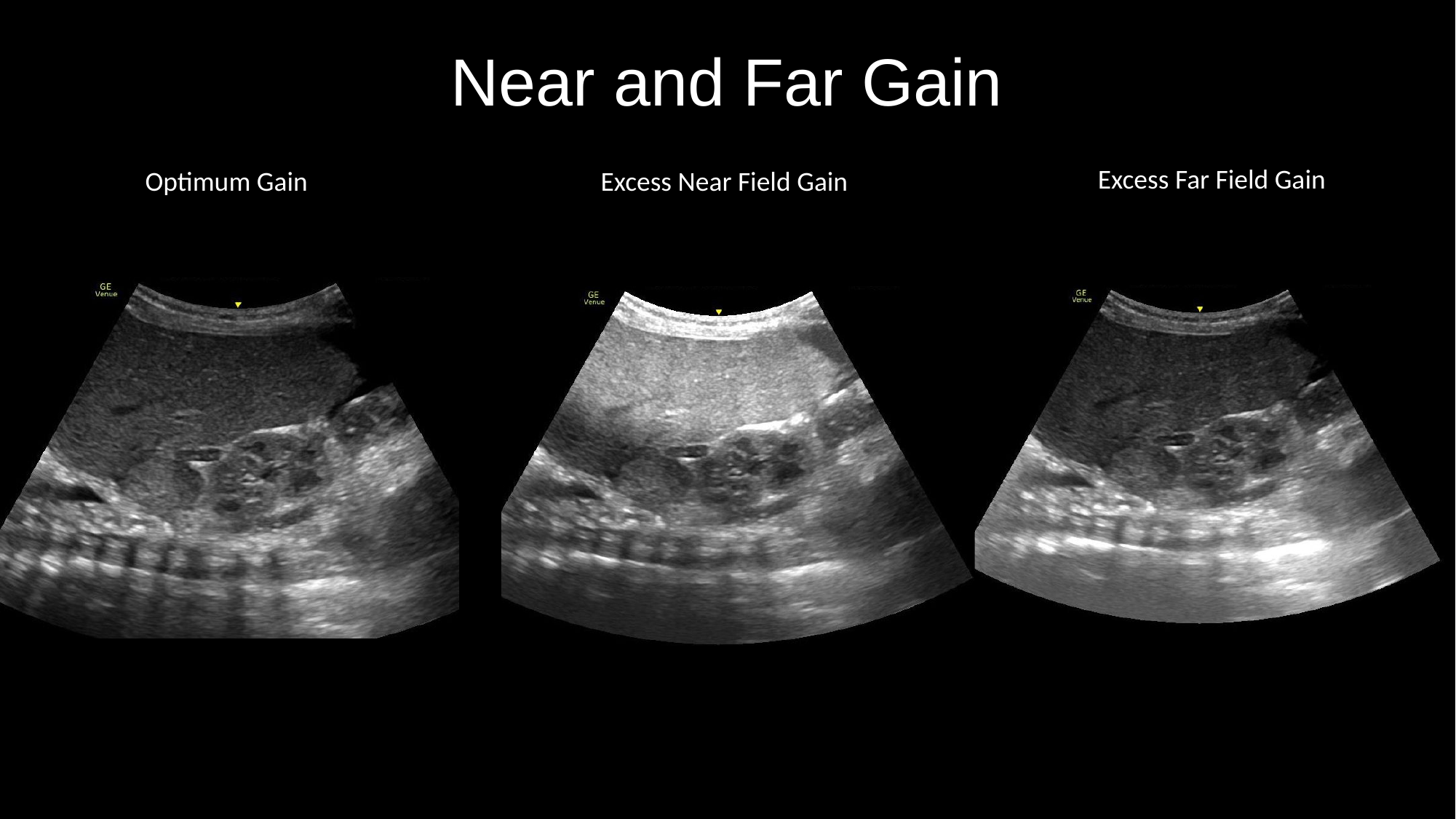

# Near and Far Gain
Excess Far Field Gain
Optimum Gain
Excess Near Field Gain

## Slide 21
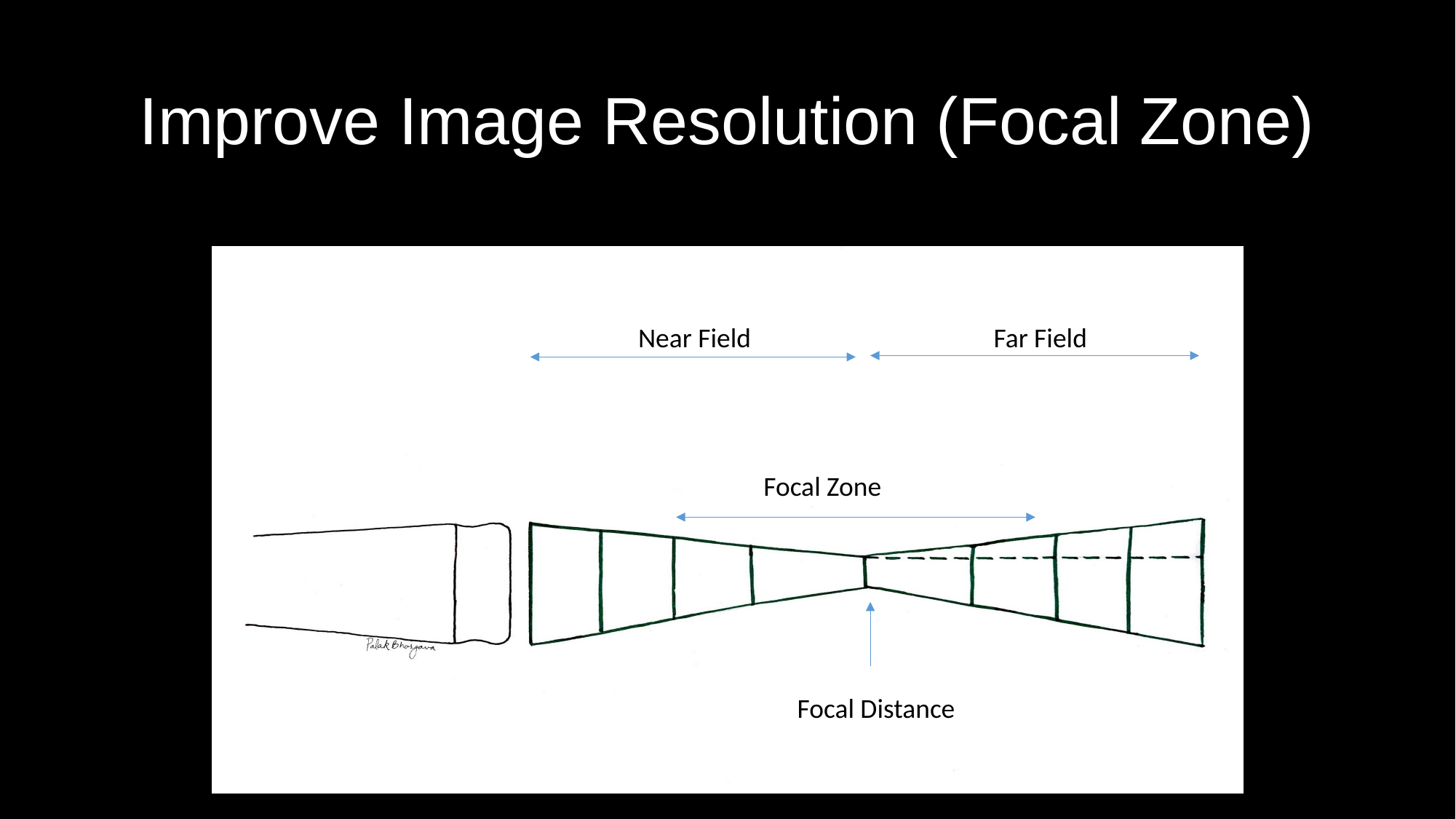

# Improve Image Resolution (Focal Zone)
Near Field
Far Field
Focal Zone
Focal Distance

## Slide 22
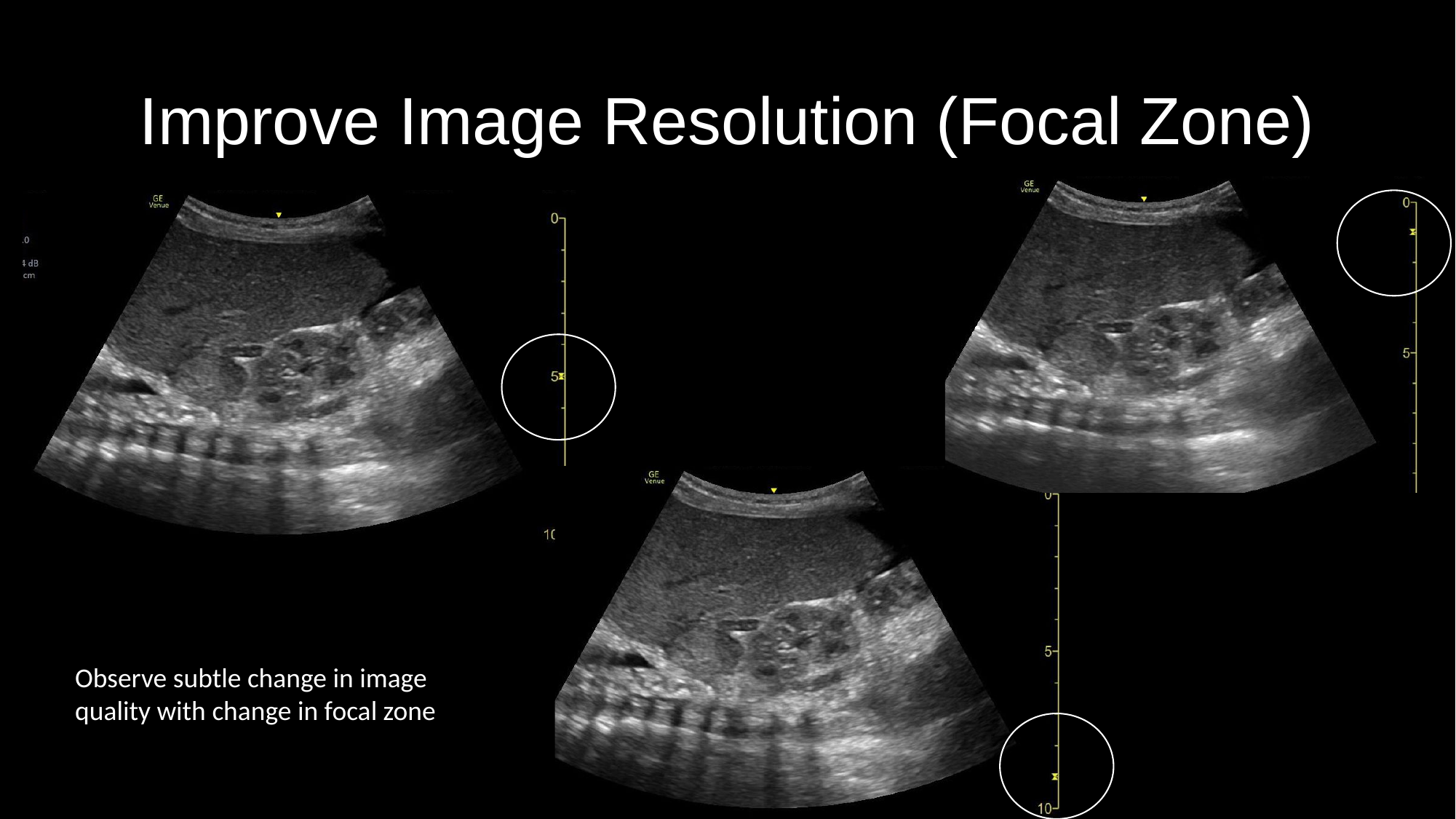

# Improve Image Resolution (Focal Zone)
Observe subtle change in image quality with change in focal zone

## Slide 23
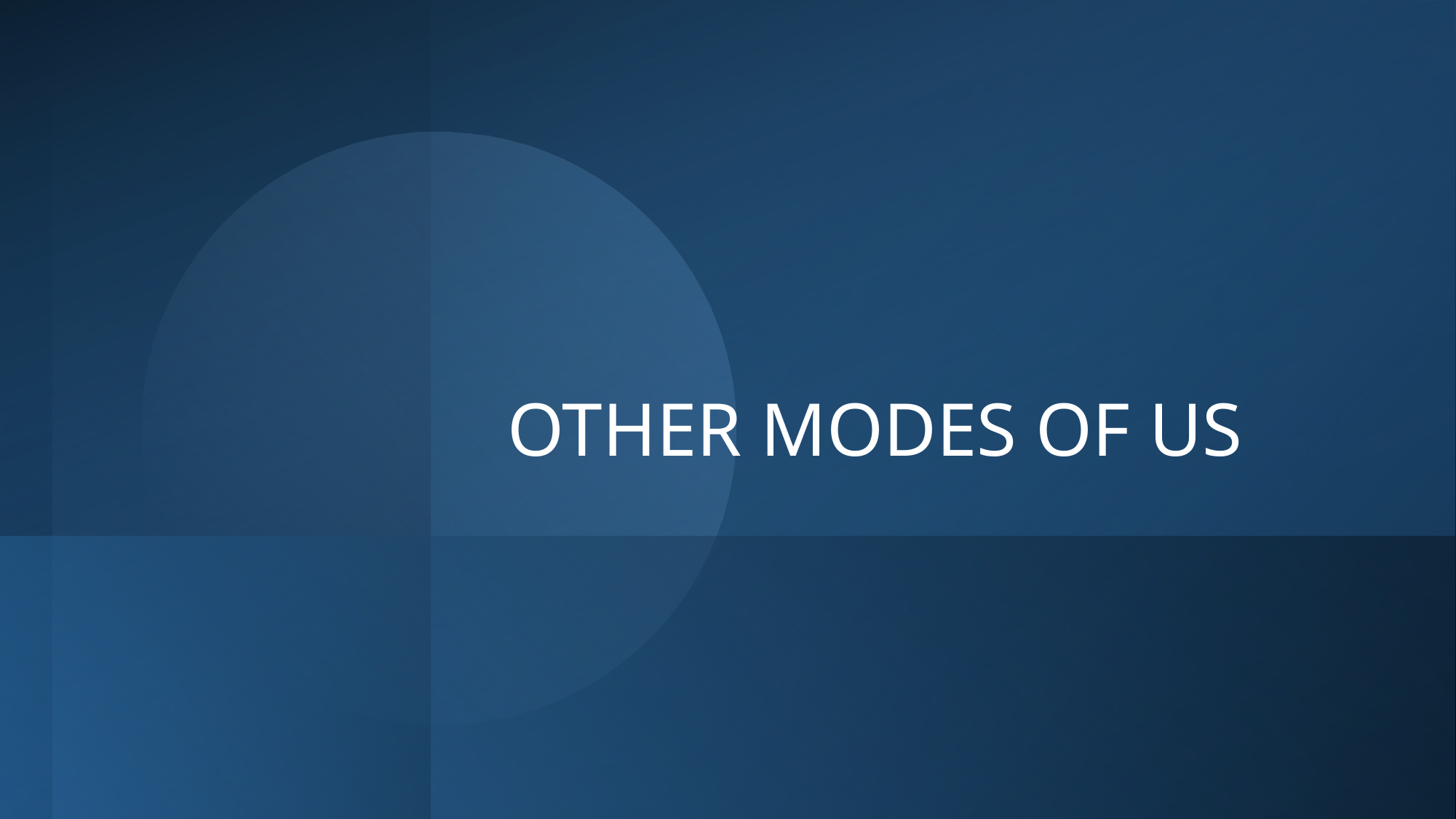

# OTHER MODES OF US

## Slide 24
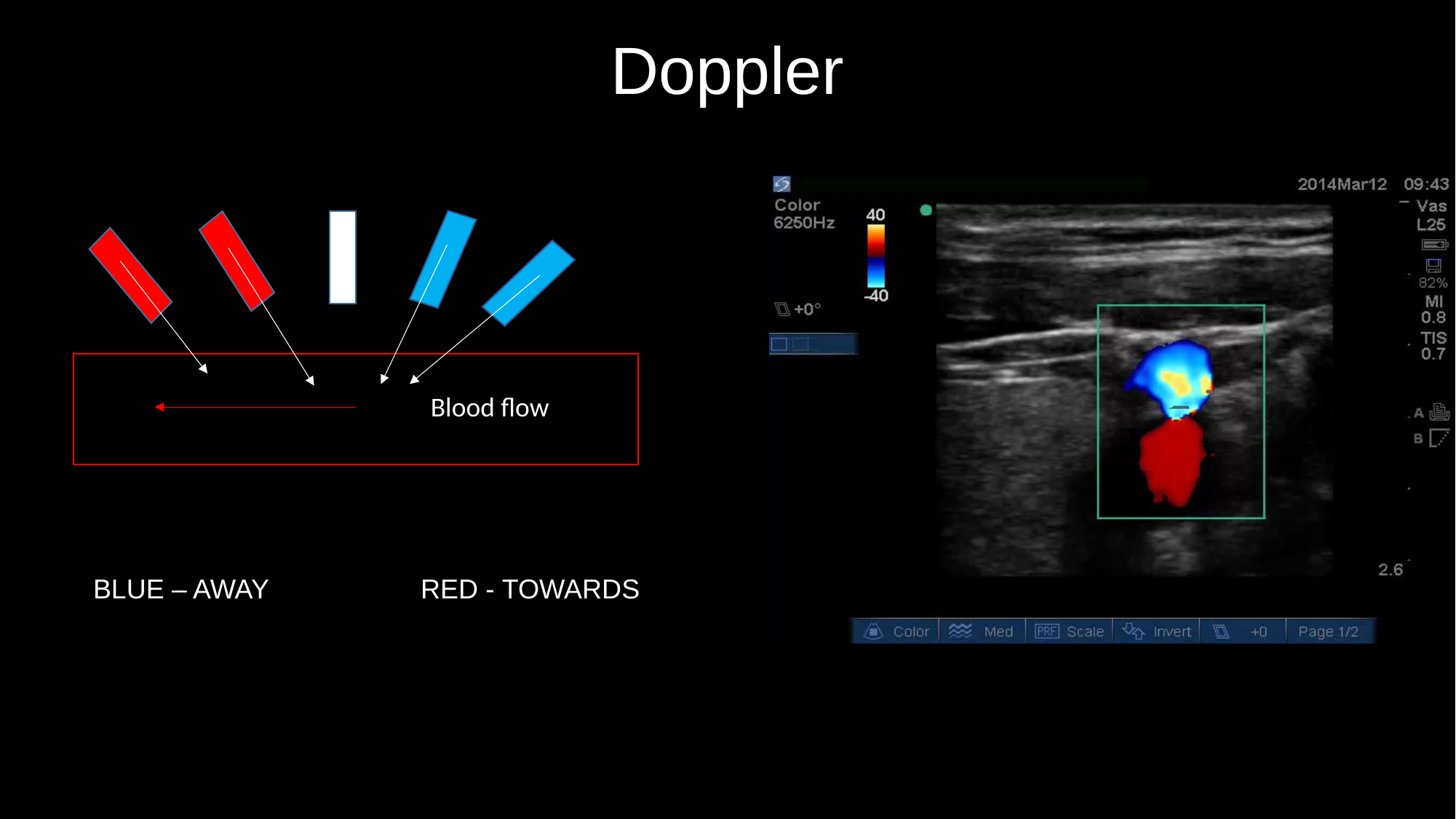

# Doppler
Blood flow
BLUE – AWAY 		RED - TOWARDS

## Slide 25
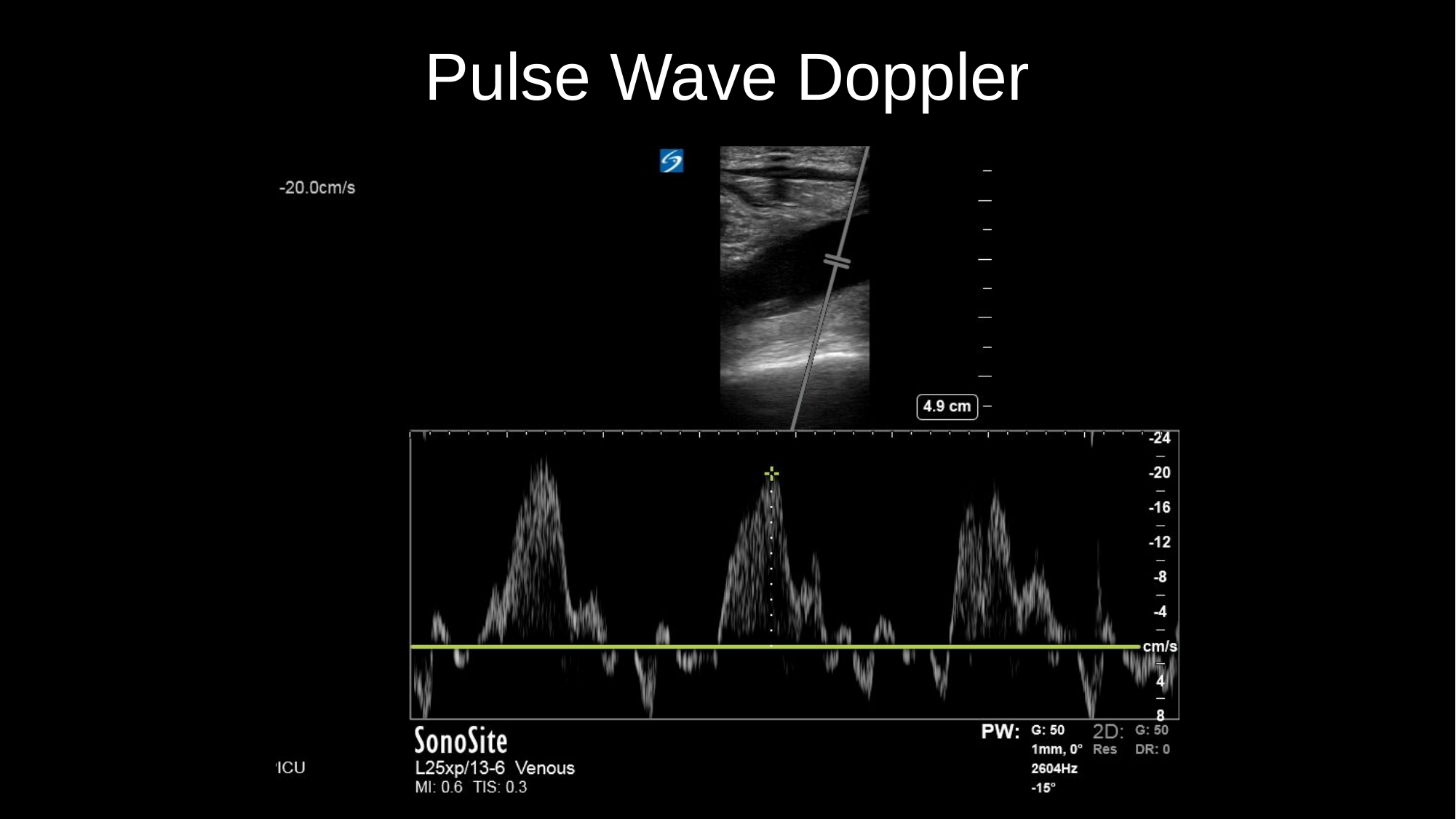

# Pulse Wave Doppler

## Slide 26
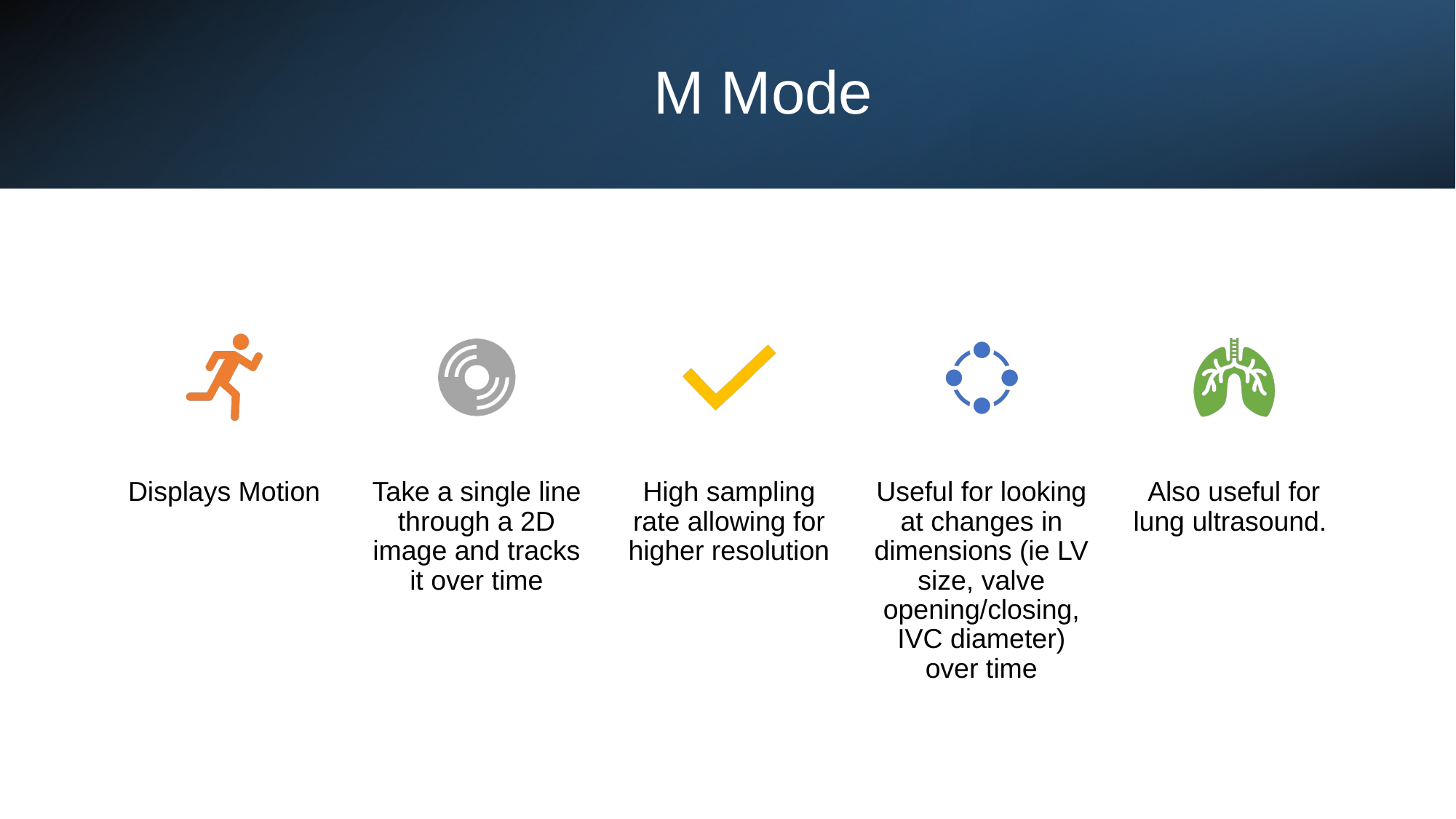

# M Mode

## Slide 27
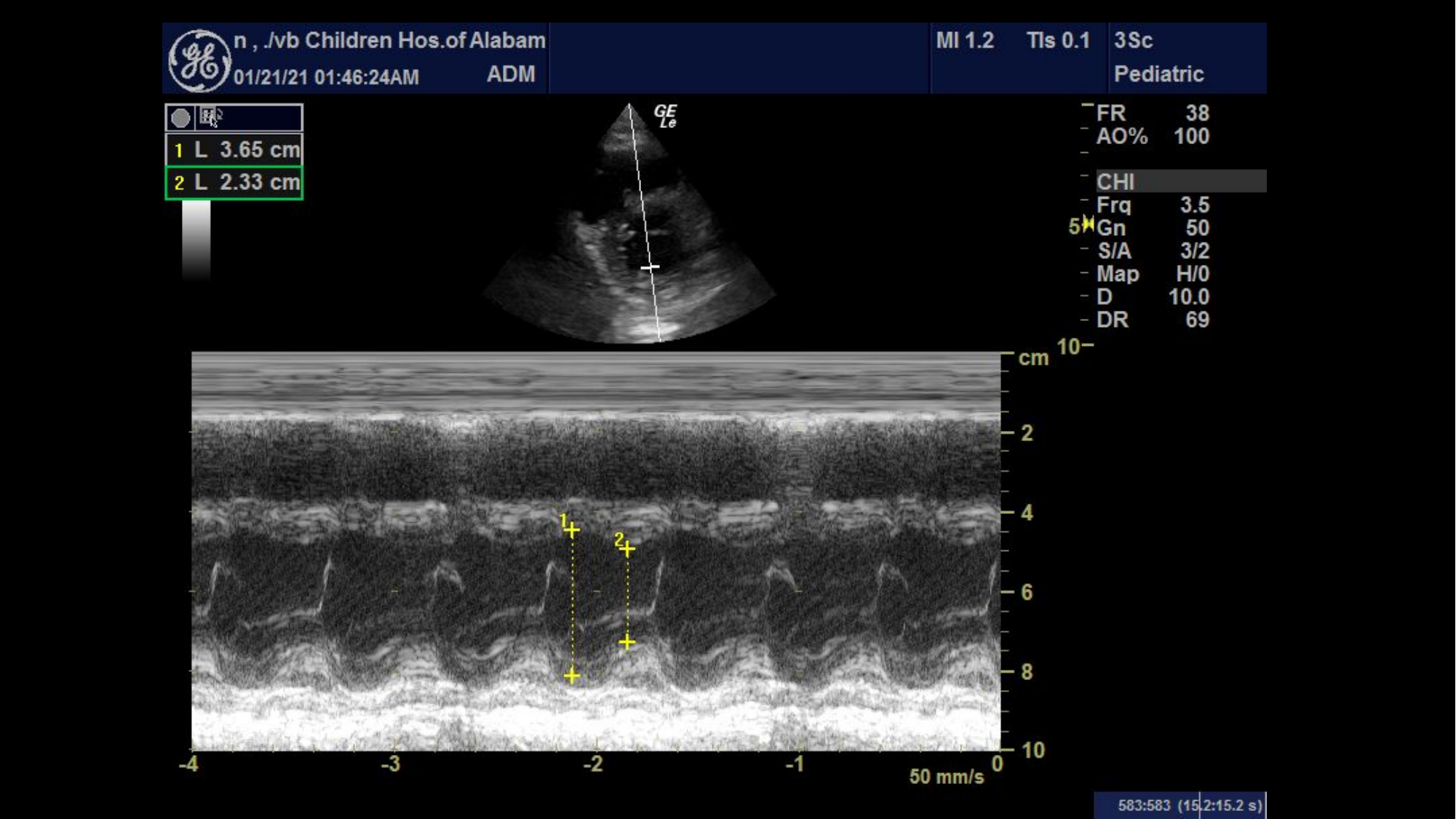

## Slide 28
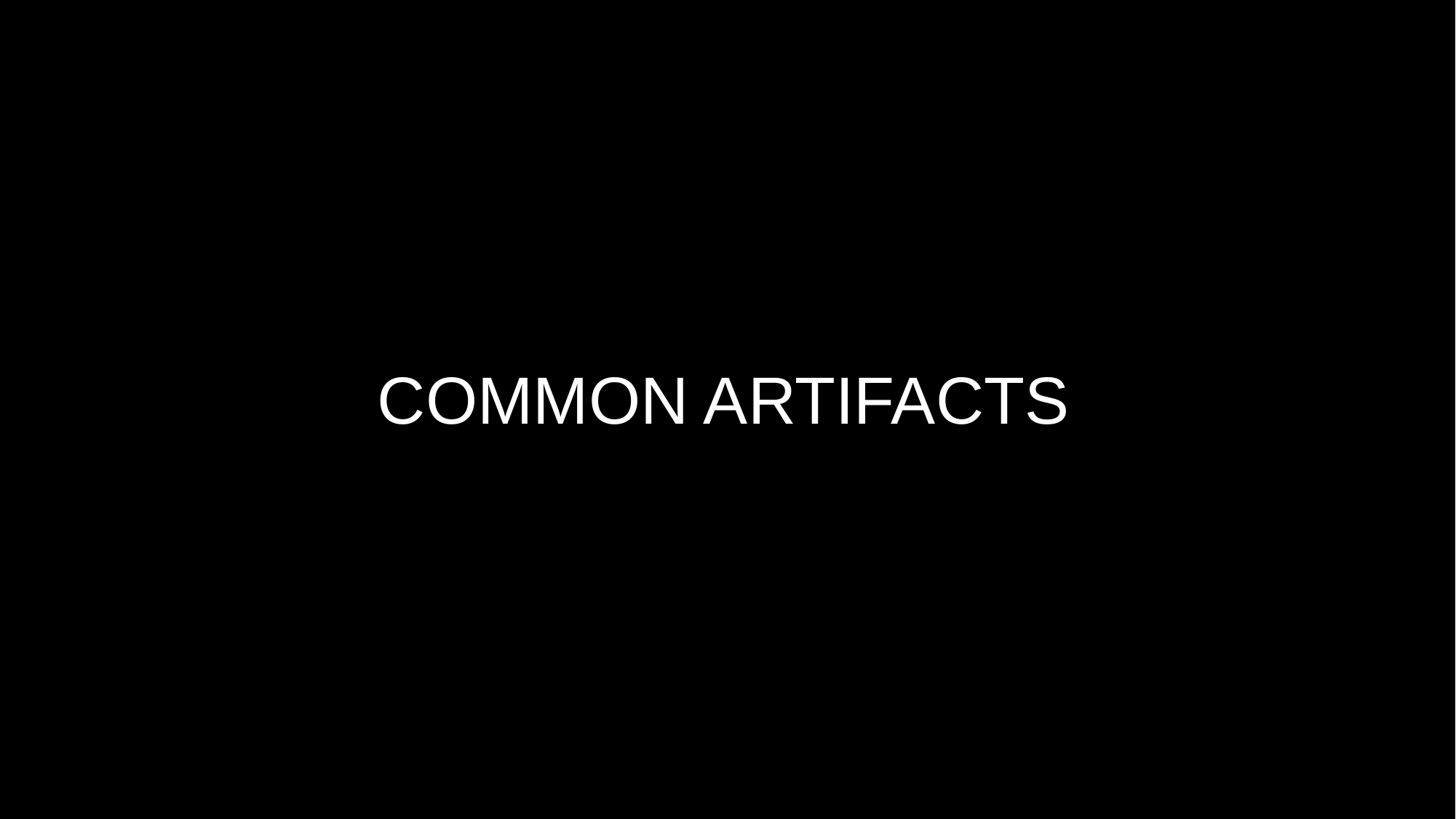

# COMMON ARTIFACTS

## Slide 29
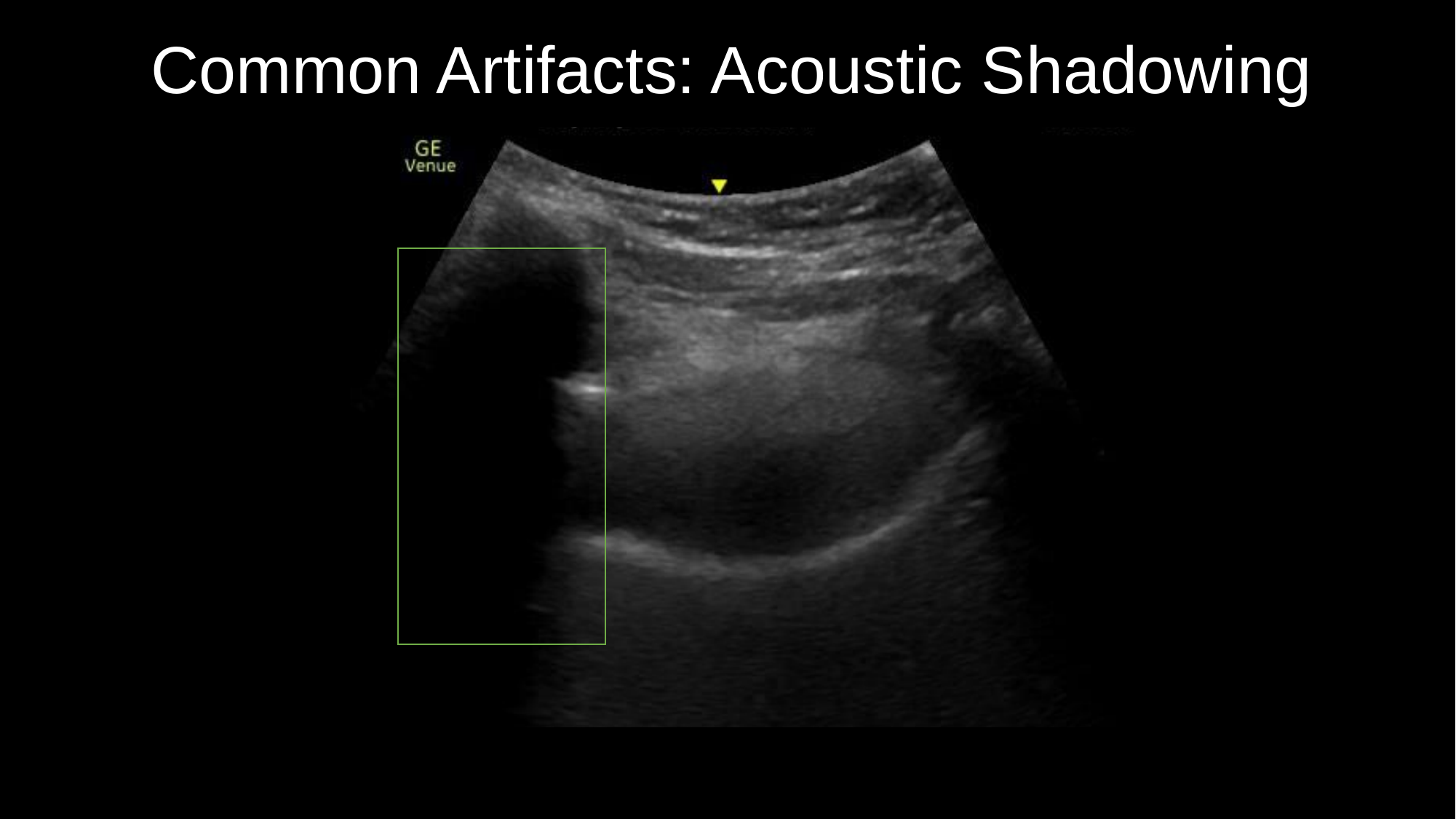

# Common Artifacts: Acoustic Shadowing

## Slide 30
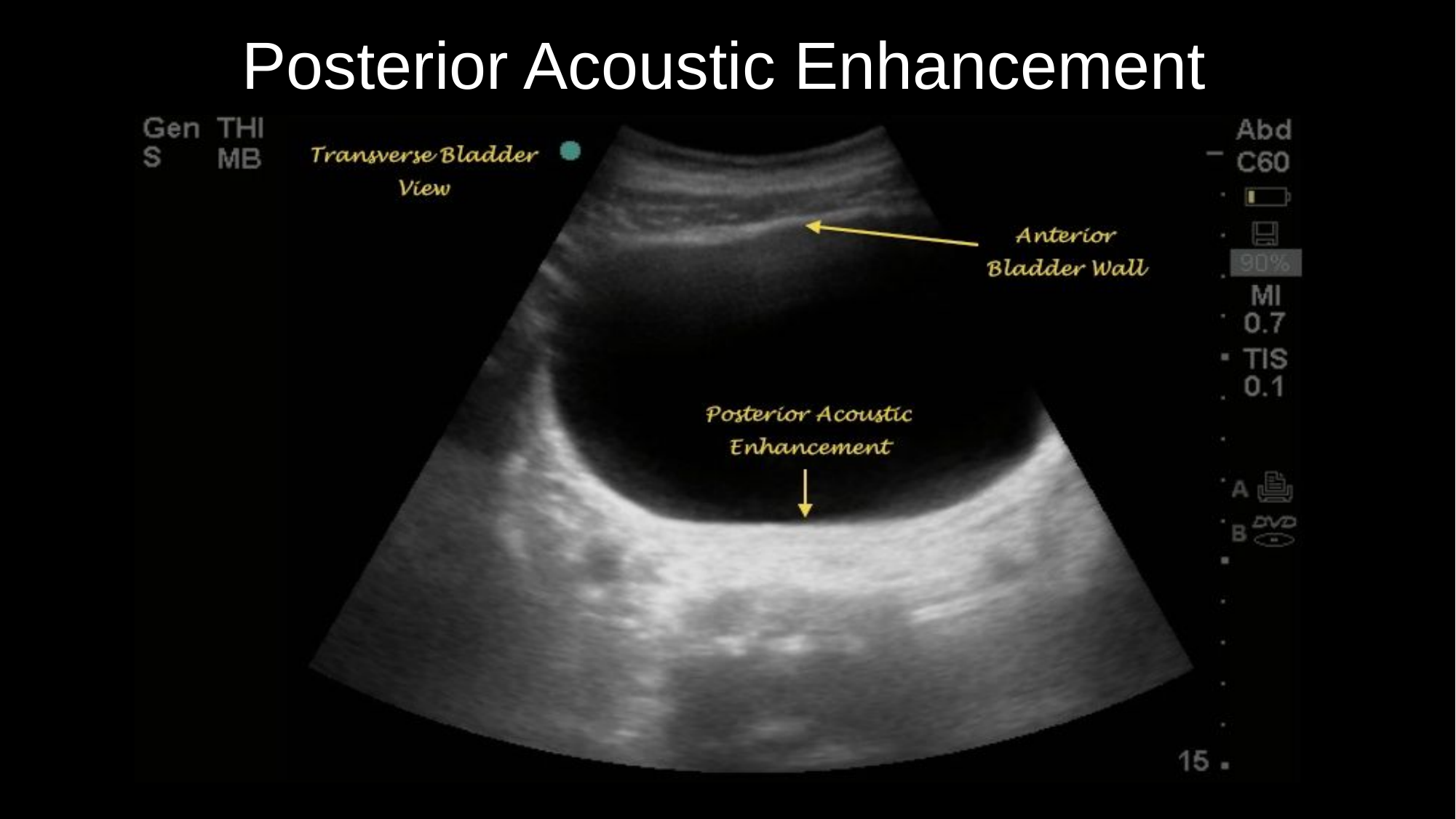

# Posterior Acoustic Enhancement

## Slide 31
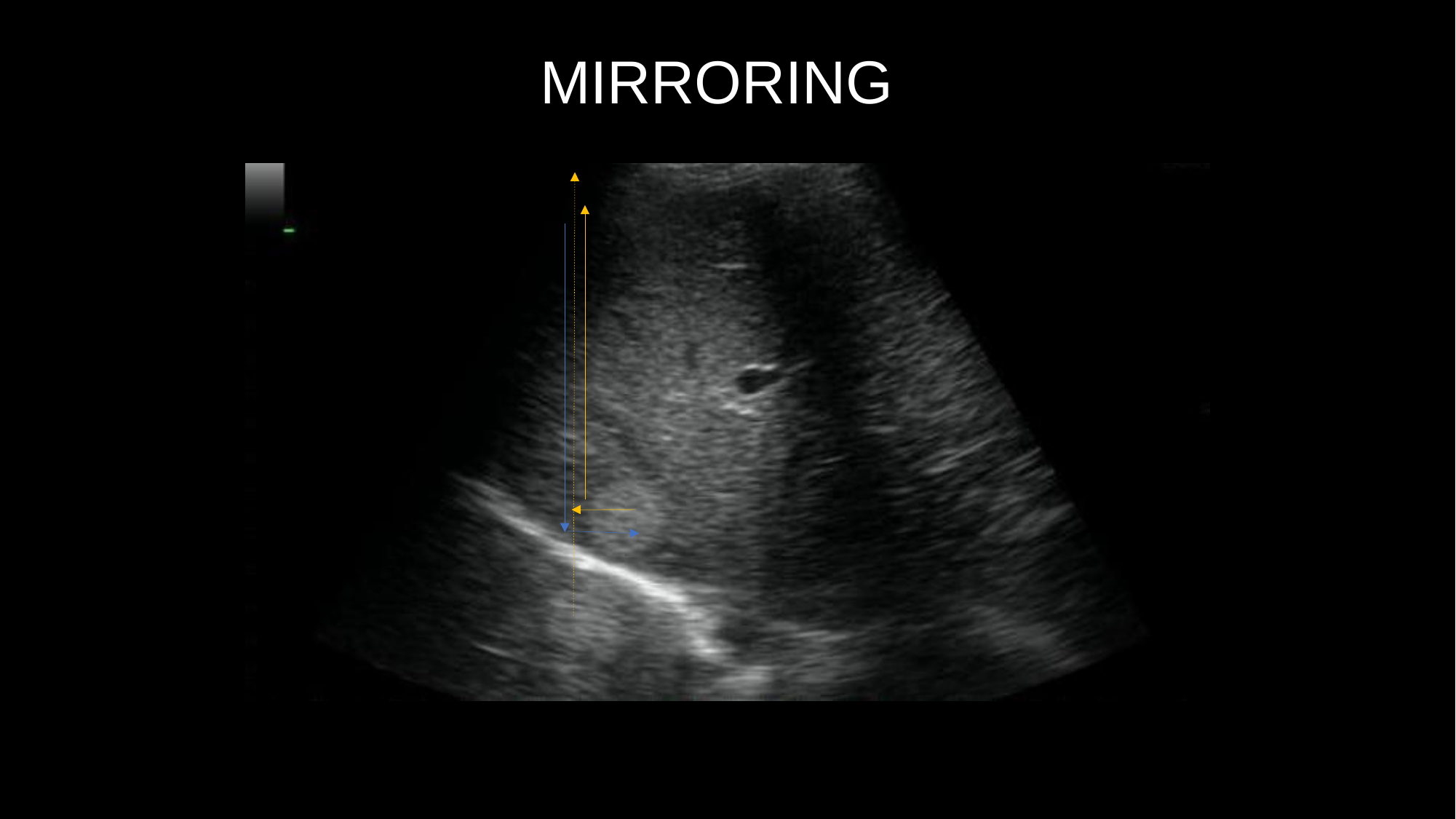

# MIRRORING

## Slide 32
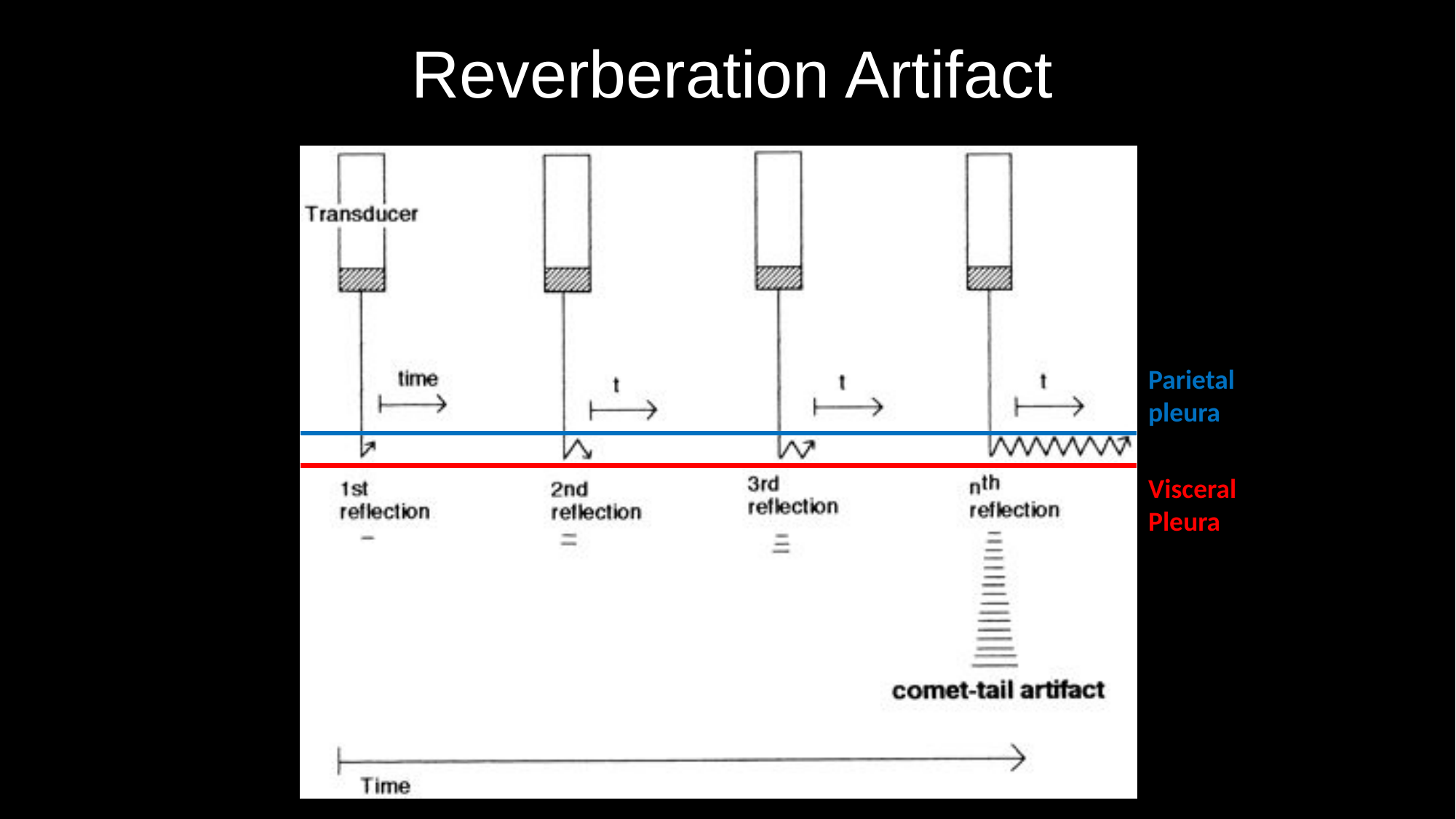

# Reverberation Artifact
Parietal pleura
Visceral Pleura

## Slide 33
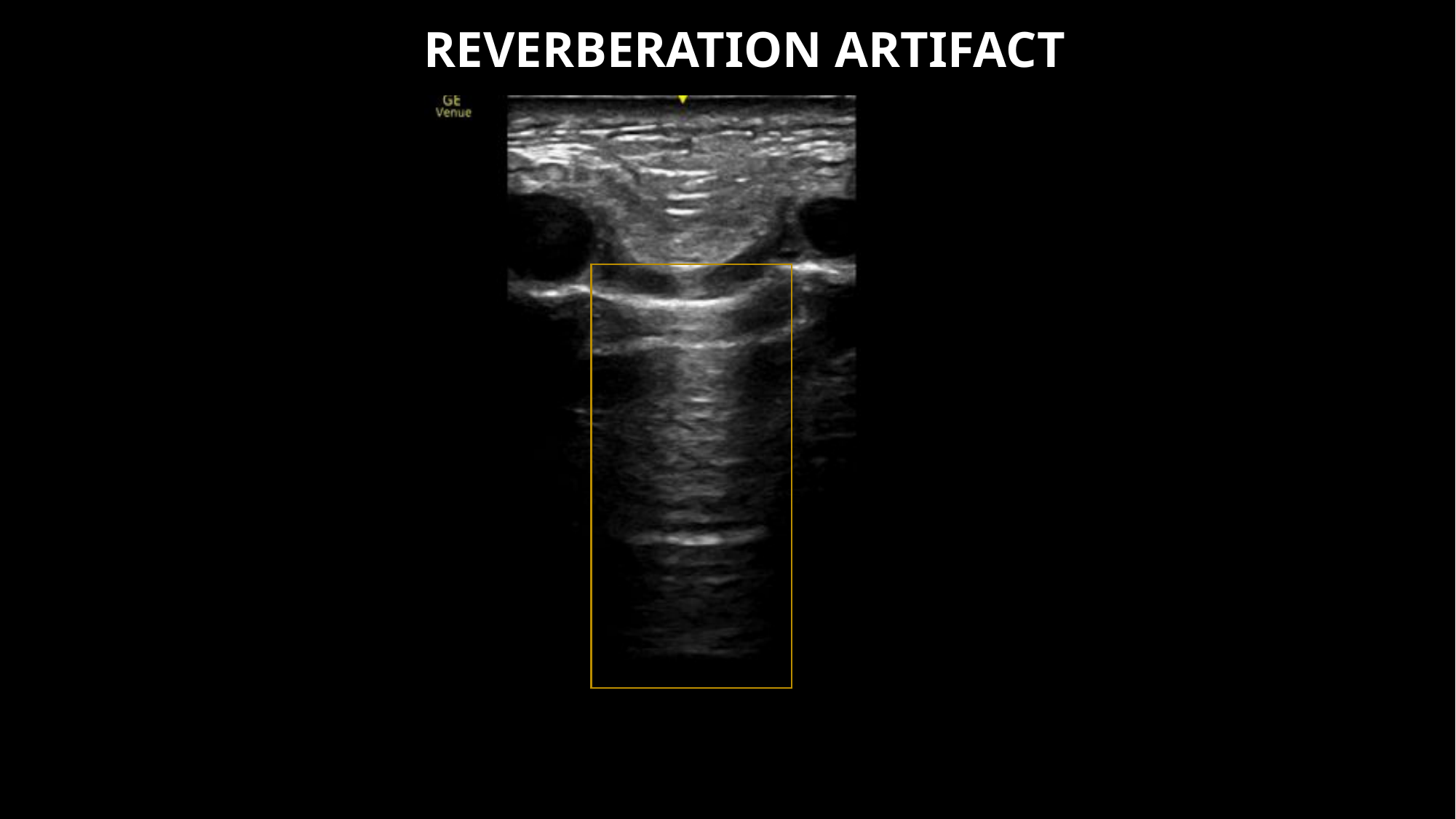

# REVERBERATION ARTIFACT

## Slide 34
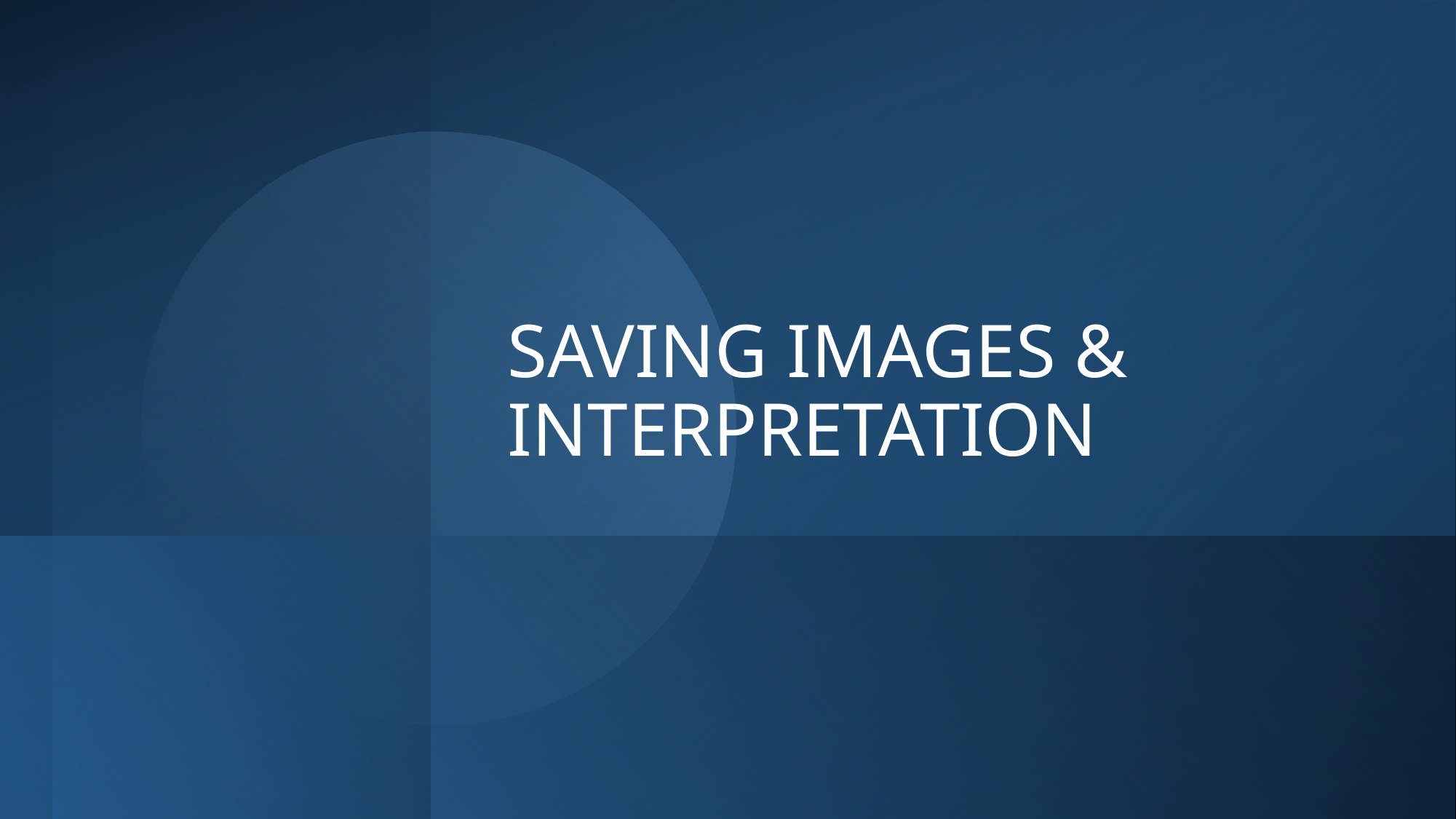

# SAVING IMAGES & INTERPRETATION

## Slide 35
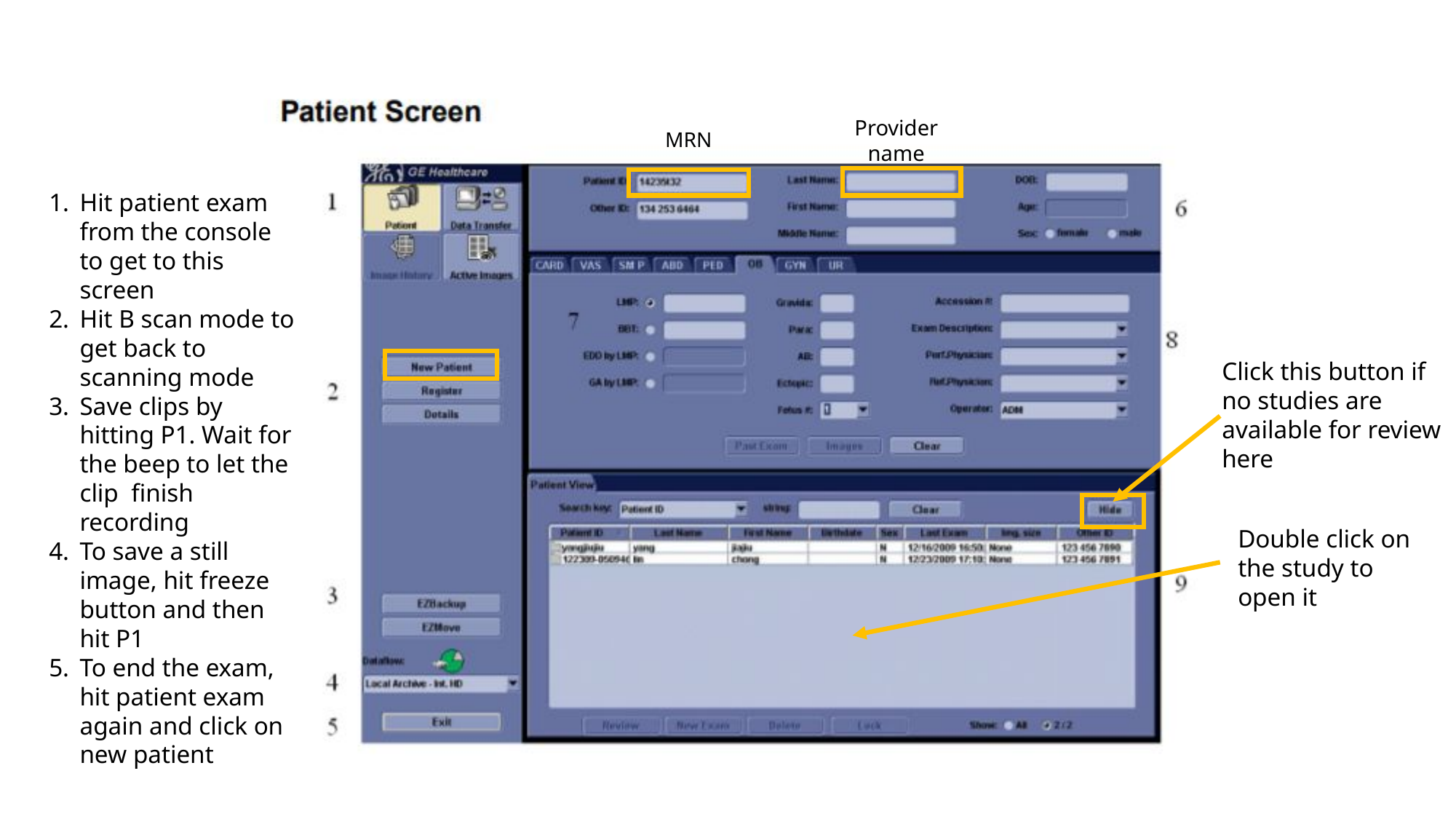

Provider name
MRN
Hit patient exam from the console to get to this screen
Hit B scan mode to get back to scanning mode
Save clips by hitting P1. Wait for the beep to let the clip finish recording
To save a still image, hit freeze button and then hit P1
To end the exam, hit patient exam again and click on new patient
Click this button if no studies are available for review here
Double click on the study to open it

## Slide 36
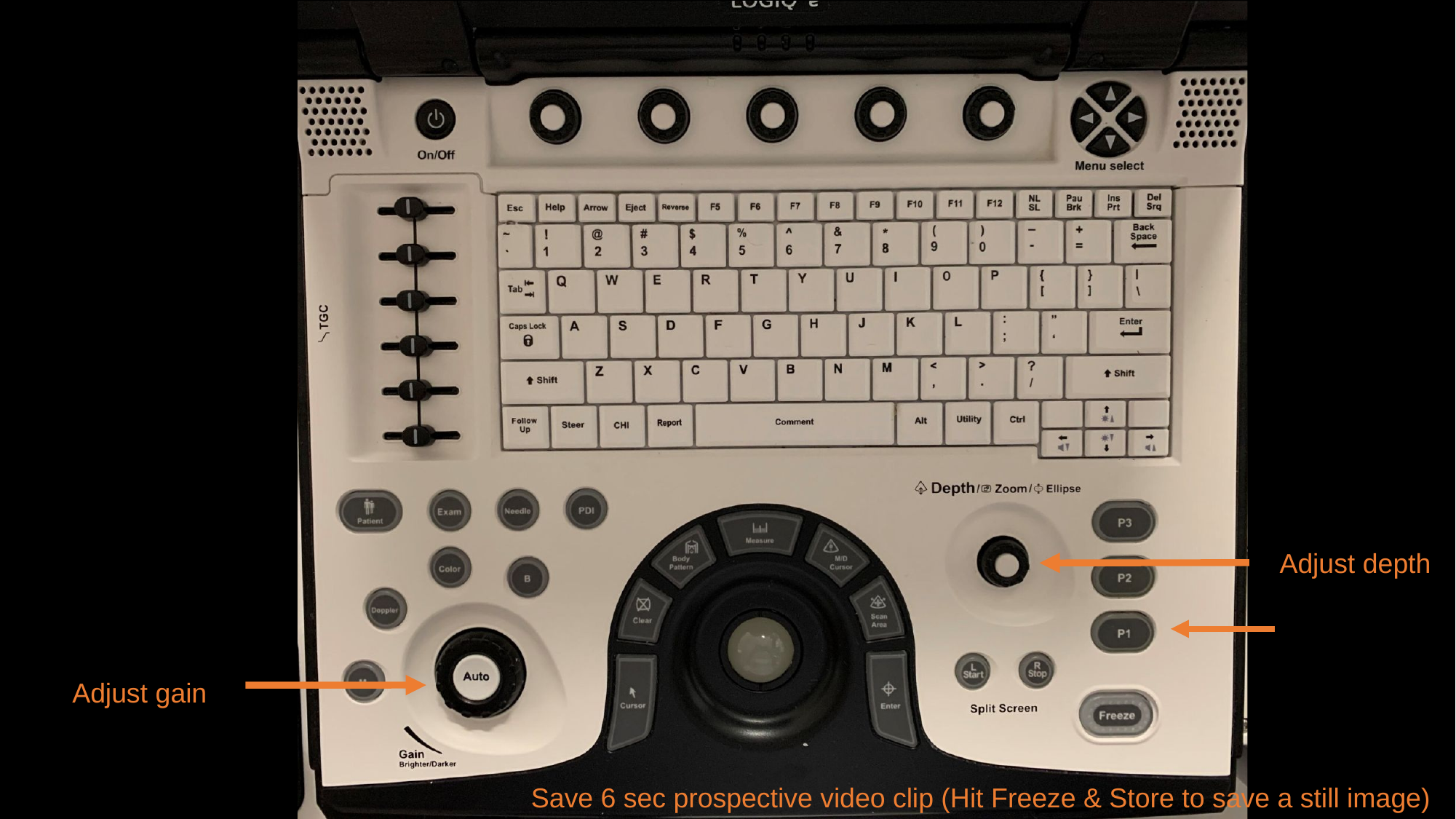

Adjust depth
Adjust gain
Save 6 sec prospective video clip (Hit Freeze & Store to save a still image)
