# Supplementary material for: A point-of-care ultrasound education curriculum for pediatric critical care medicine
Source: Ultrasound J. 2022 Oct 31;14:44. doi: 10.1186/s13089-022-00290-6 (PMC9622960; doi:10.1186/s13089-022-00290-6)
Supplement: Supplementary file 4 — Additional file 4. Ultrasound-guided vascular access procedures [file 13089_2022_290_MOESM4_ESM.pptx]

## Slide 1
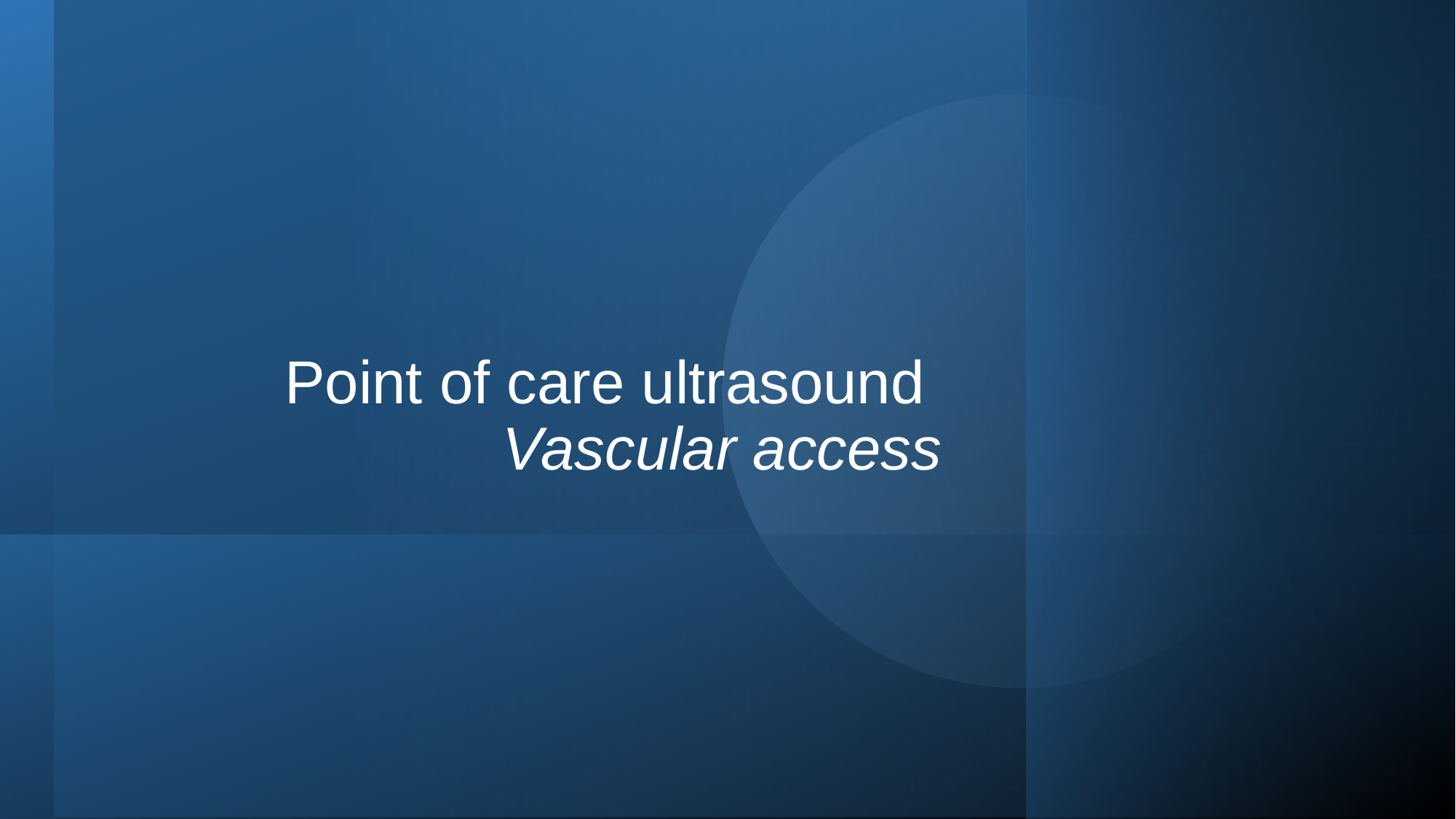

# Point of care ultrasound Vascular access

## Slide 2
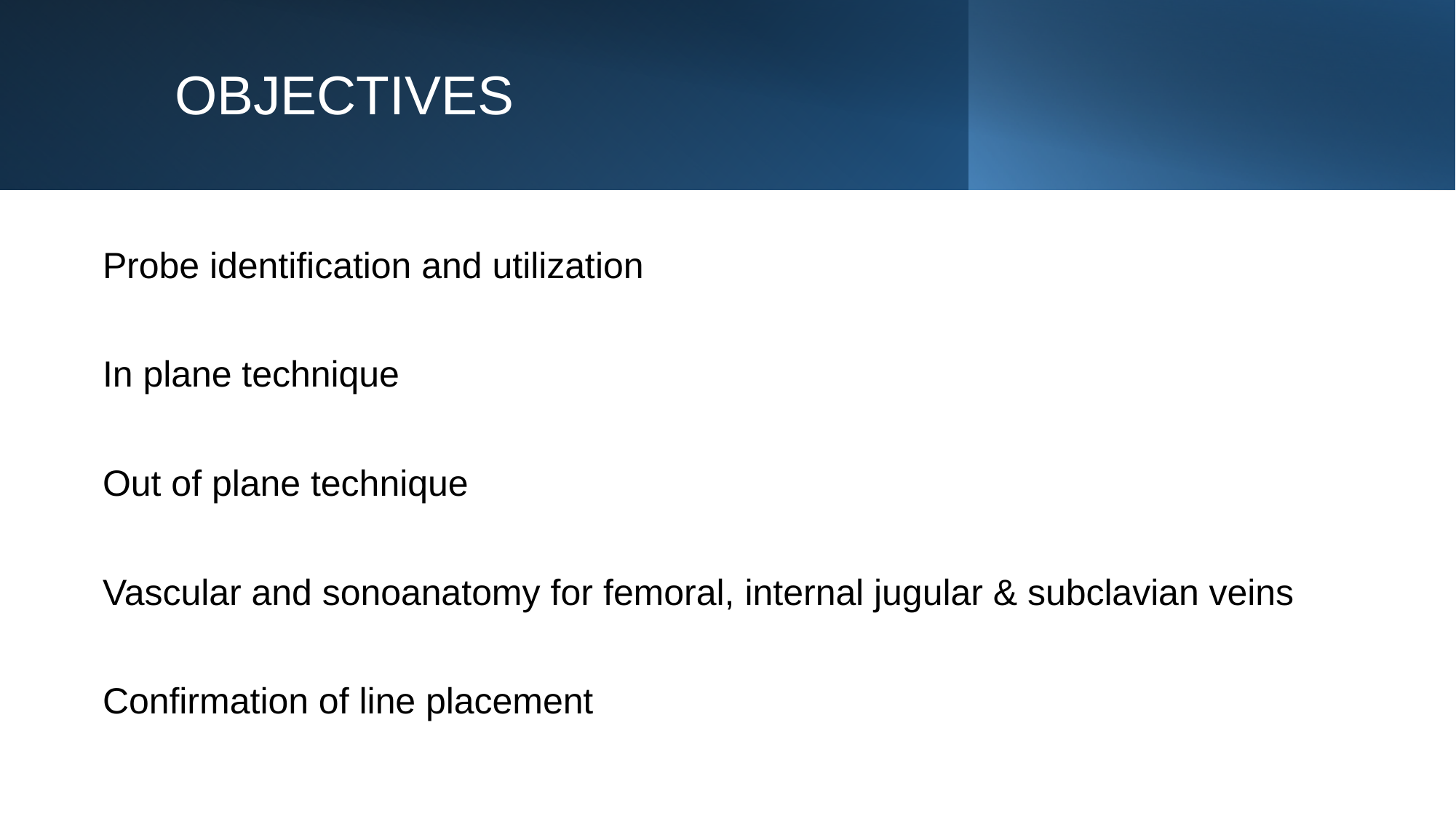

# OBJECTIVES
Probe identification and utilization
In plane technique
Out of plane technique
Vascular and sonoanatomy for femoral, internal jugular & subclavian veins
Confirmation of line placement

## Slide 3
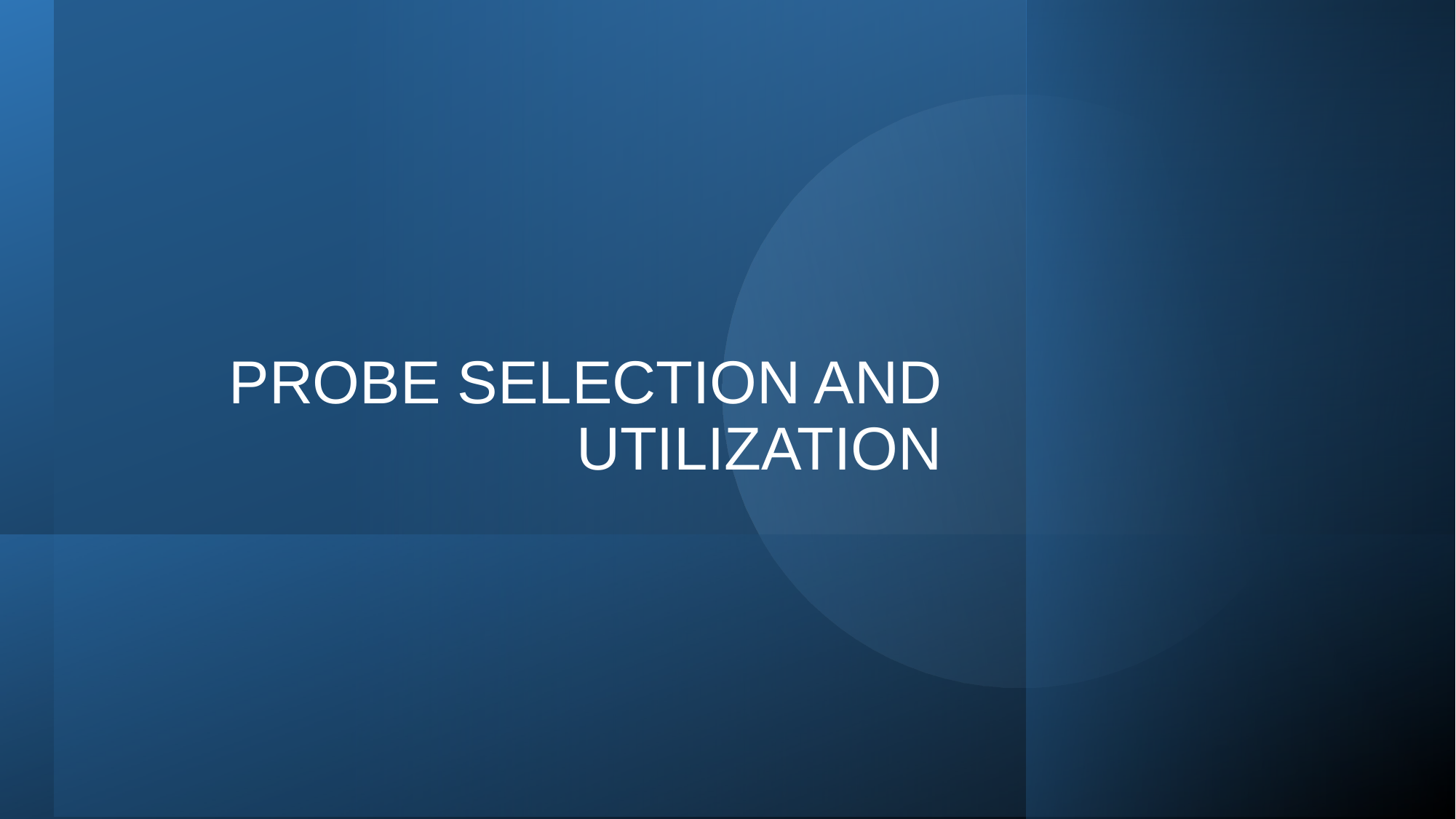

# PROBE SELECTION AND UTILIZATION

## Slide 4
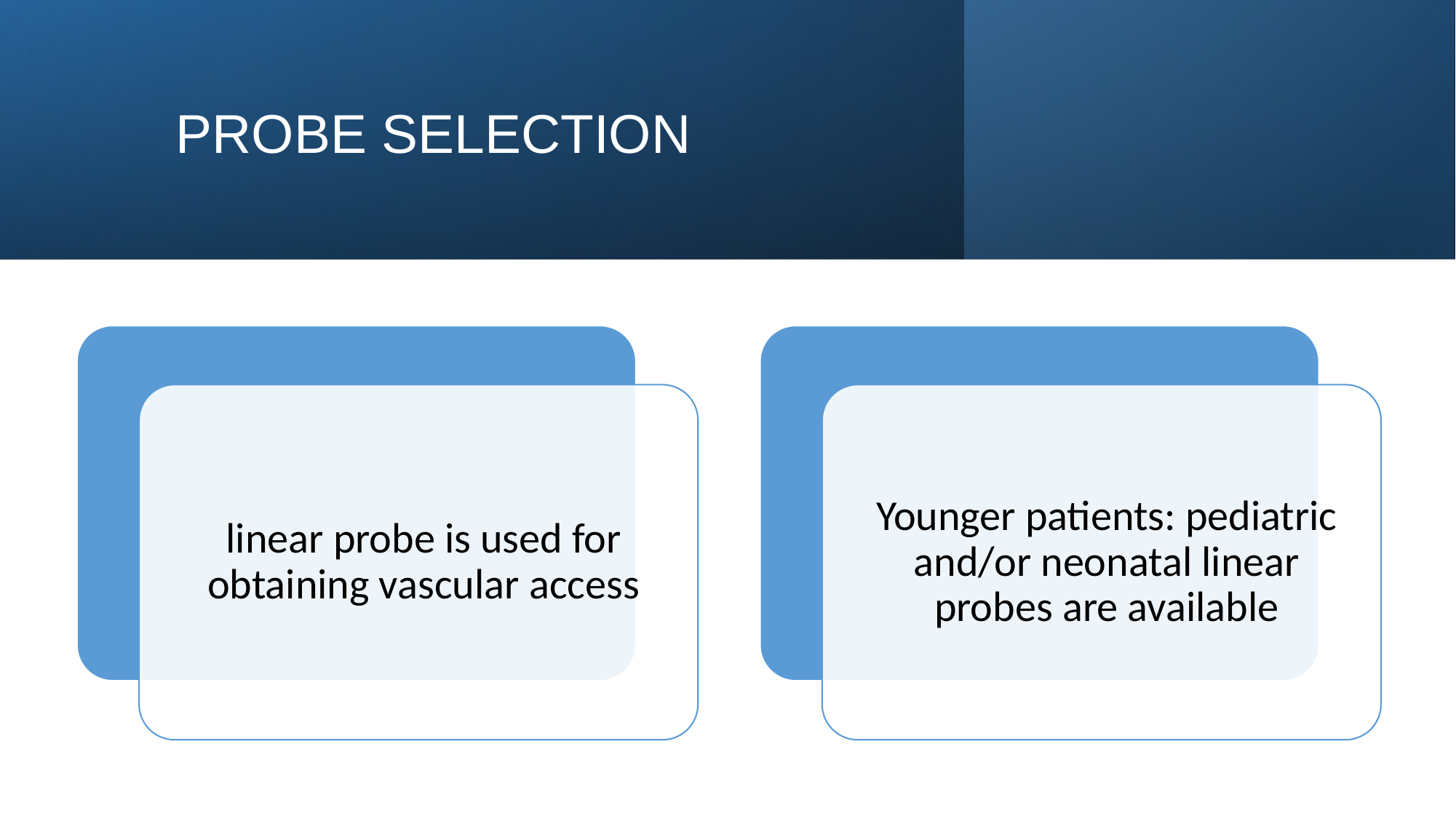

# PROBE SELECTION

## Slide 5
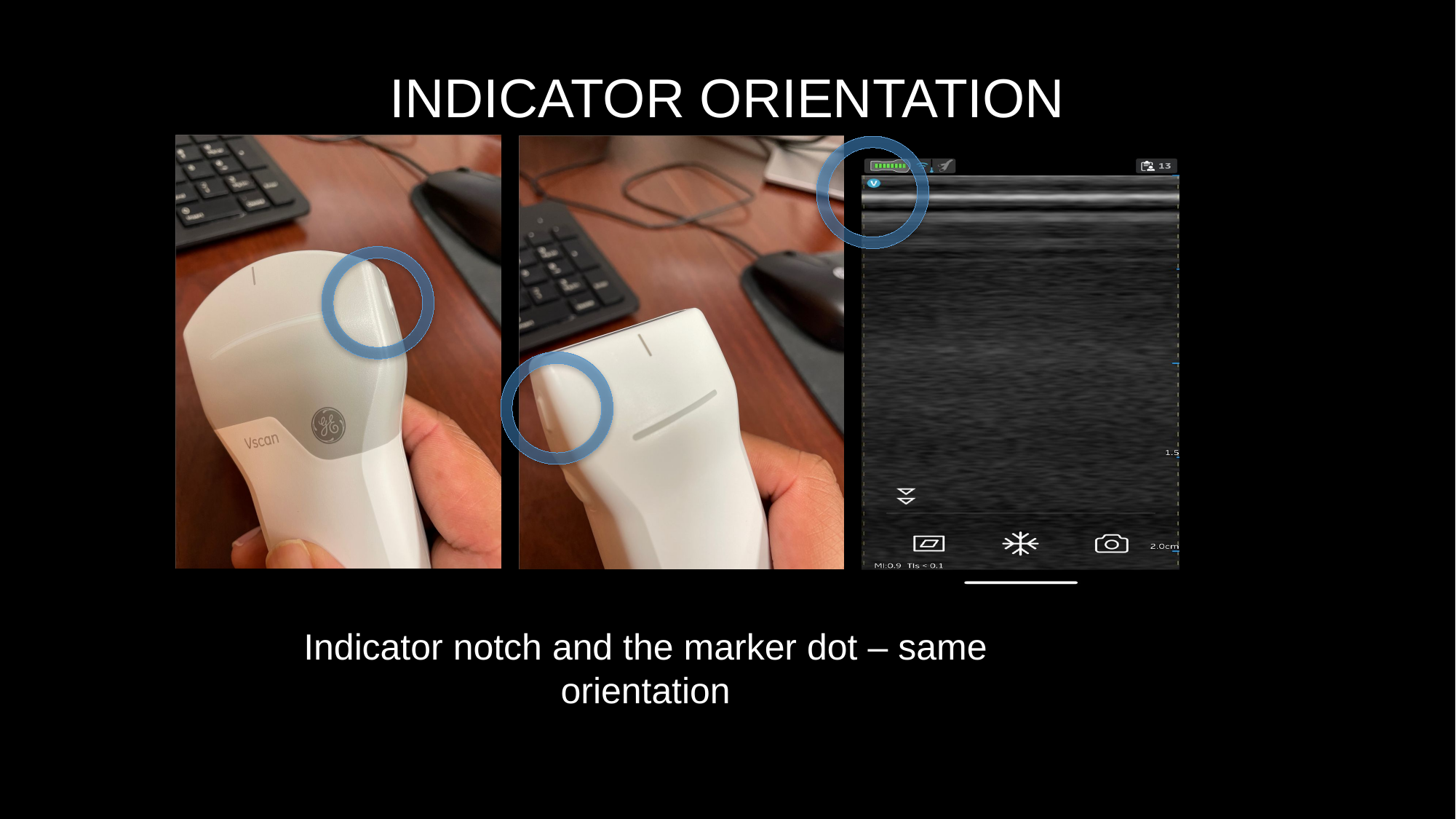

# INDICATOR ORIENTATION
Indicator notch and the marker dot – same orientation

## Slide 6
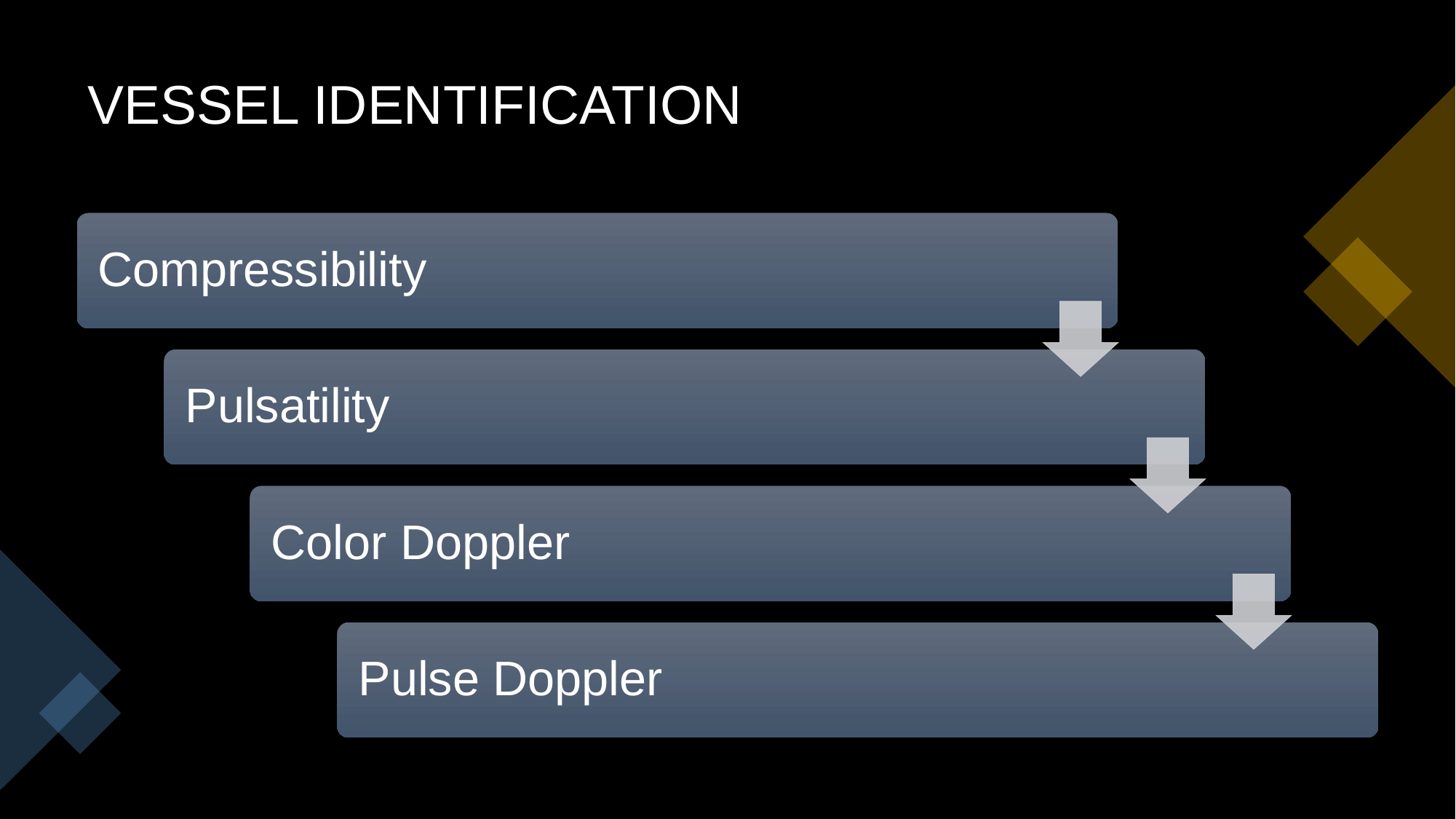

# VESSEL IDENTIFICATION

## Slide 7
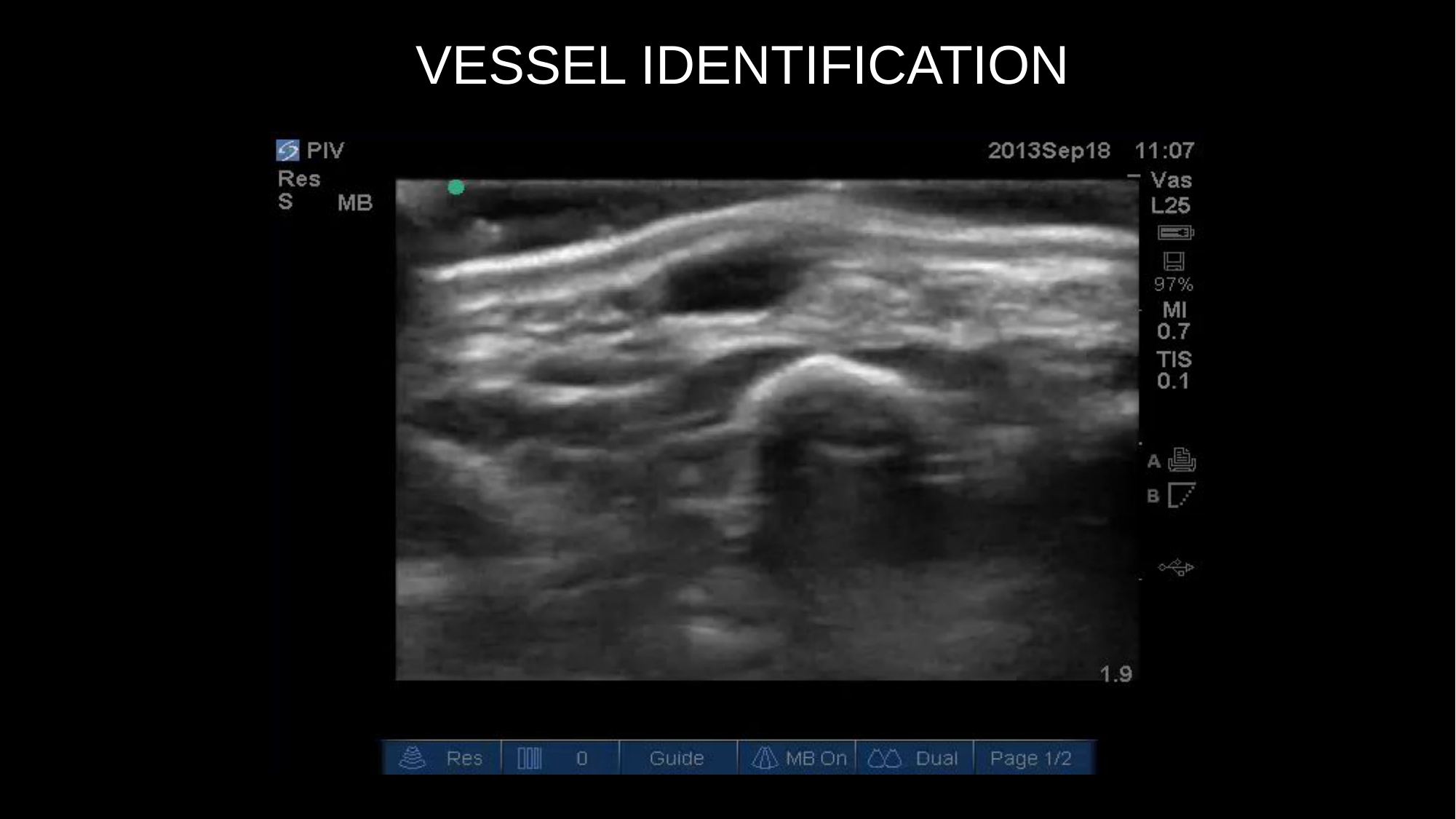

VESSEL IDENTIFICATION

## Slide 8
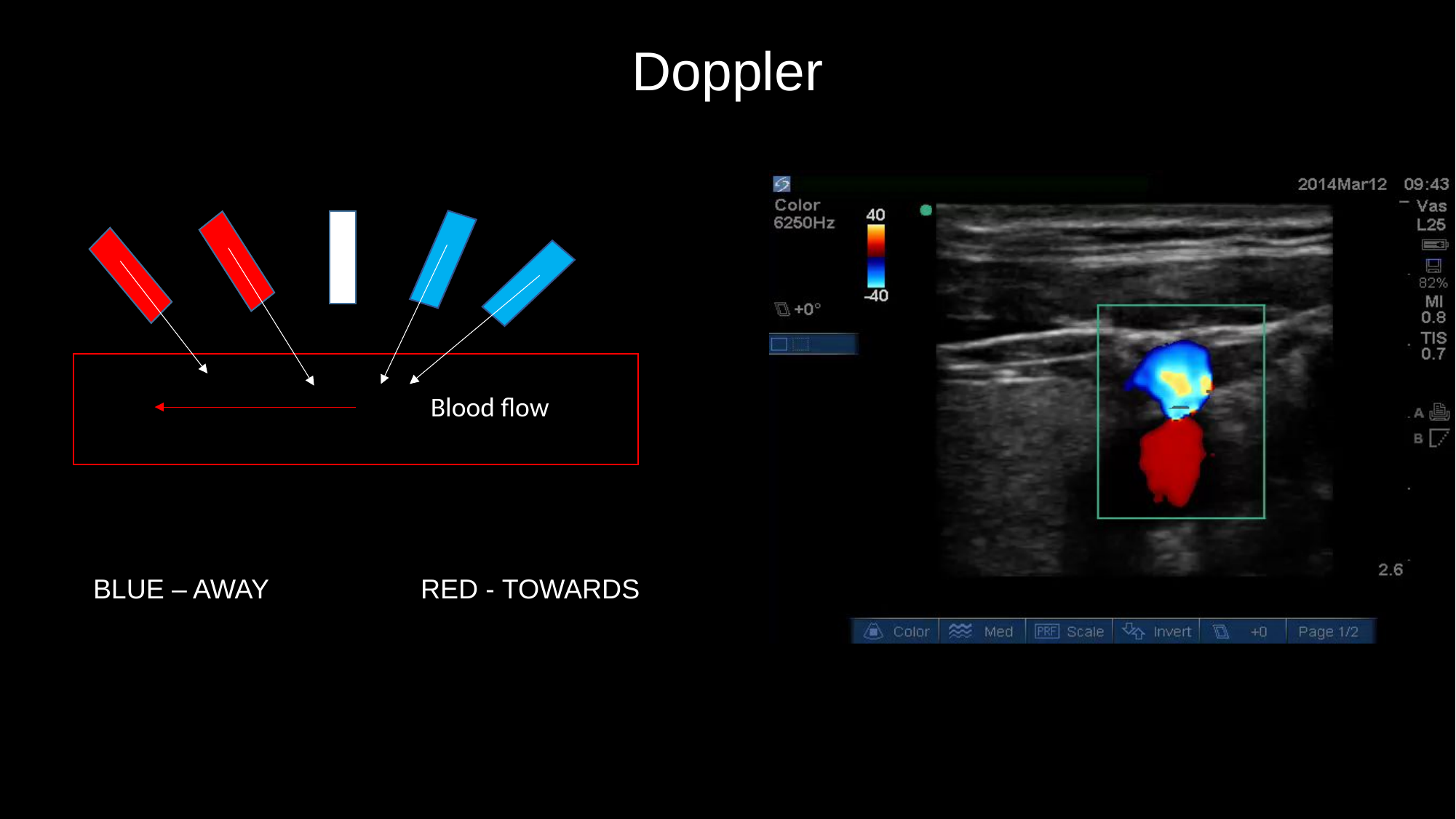

# Doppler
Blood flow
BLUE – AWAY 		RED - TOWARDS

## Slide 9
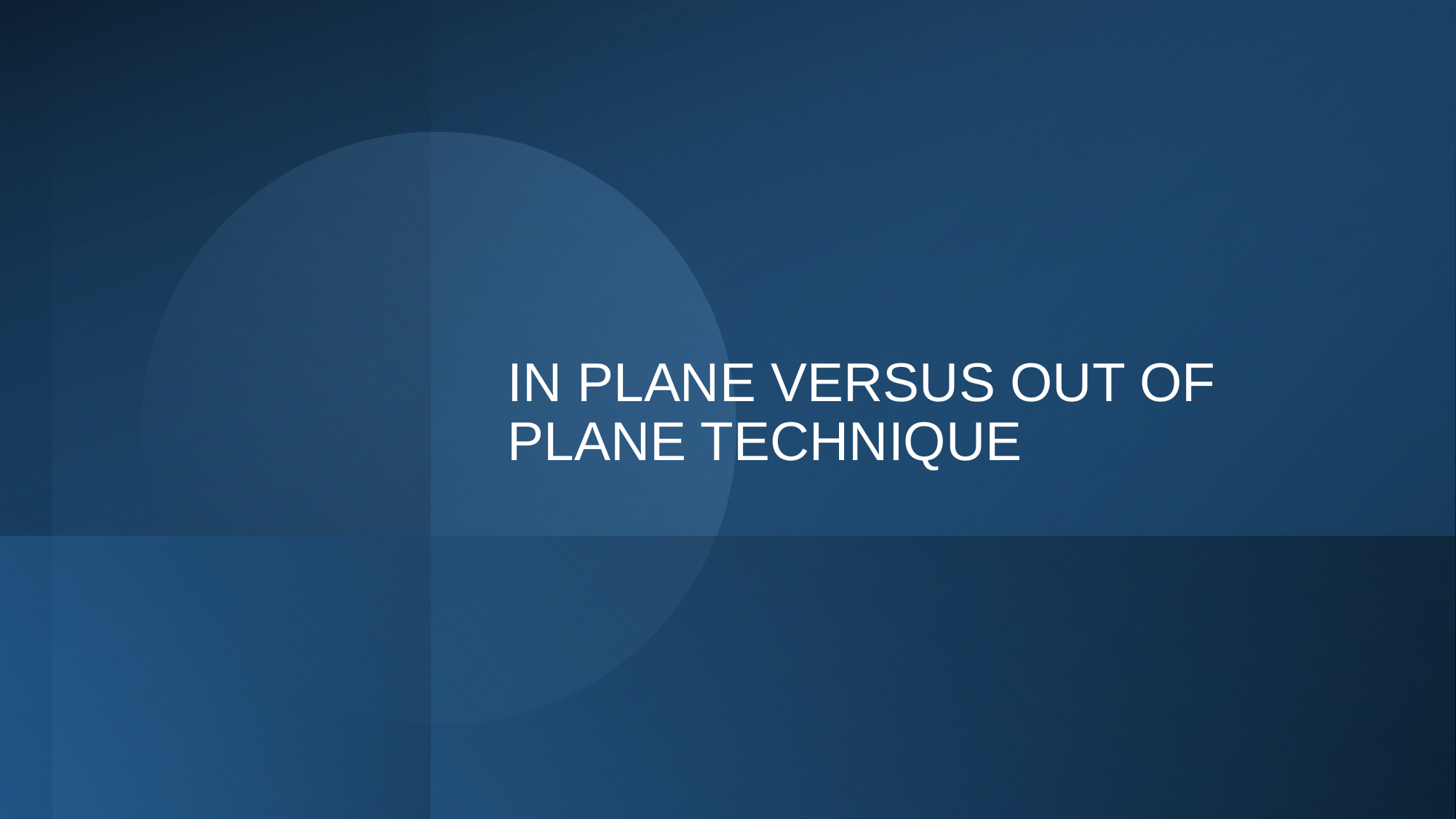

IN PLANE VERSUS OUT OF PLANE TECHNIQUE

## Slide 10
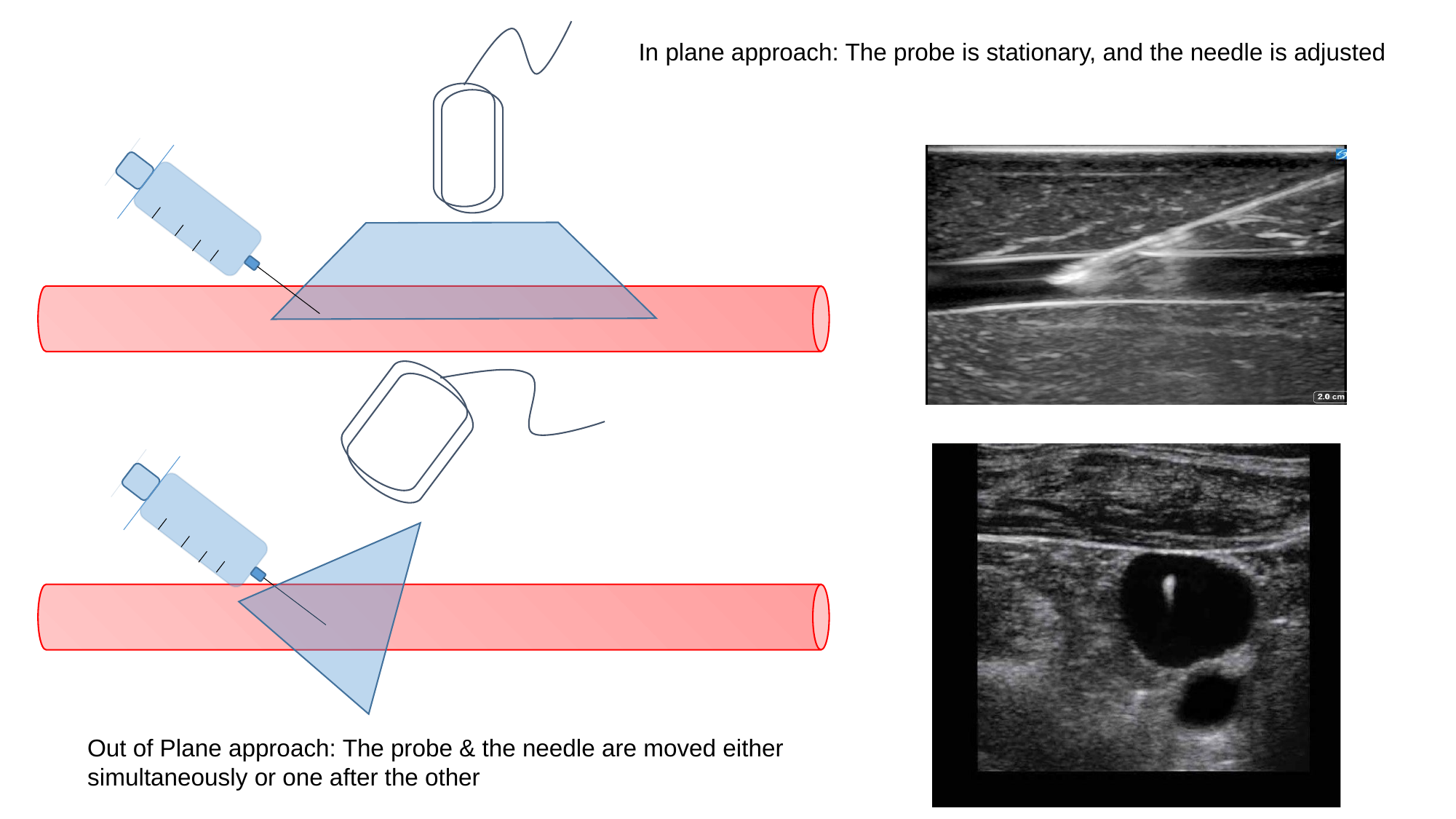

In plane approach: The probe is stationary, and the needle is adjusted
Out of Plane approach: The probe & the needle are moved either simultaneously or one after the other

## Slide 11
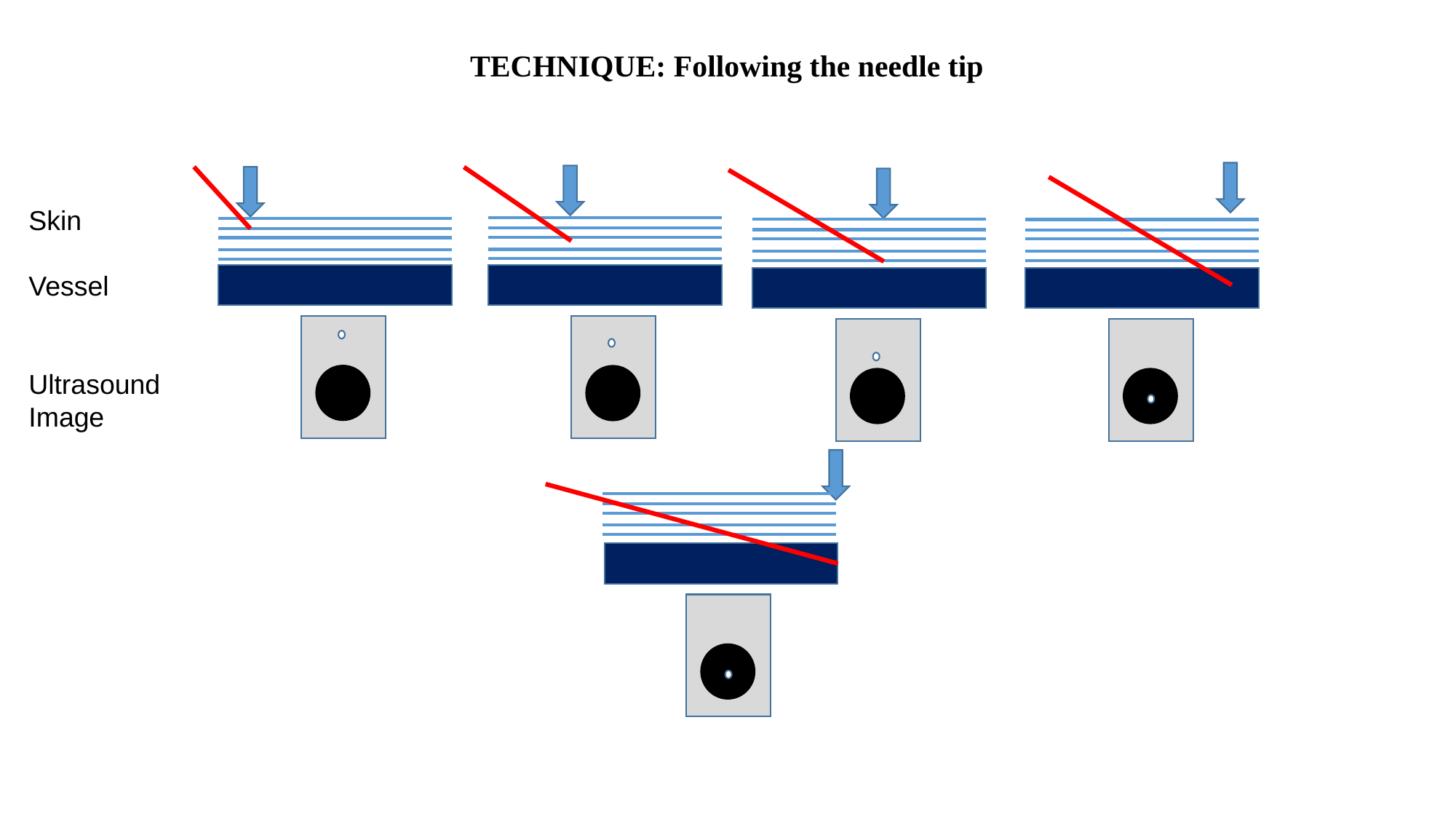

TECHNIQUE: Following the needle tip
Skin
Vessel
Ultrasound Image

## Slide 12
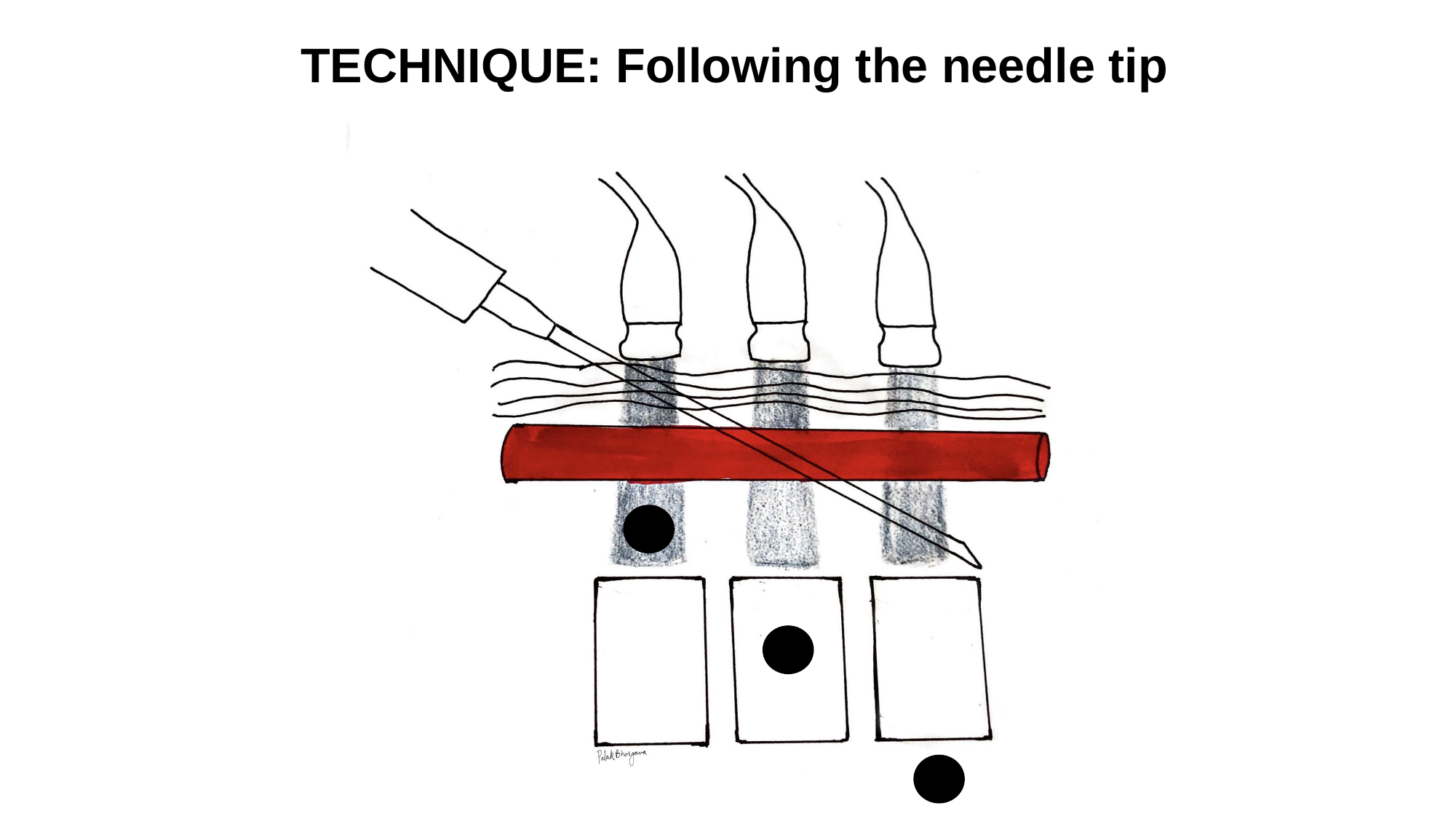

TECHNIQUE: Following the needle tip

## Slide 13
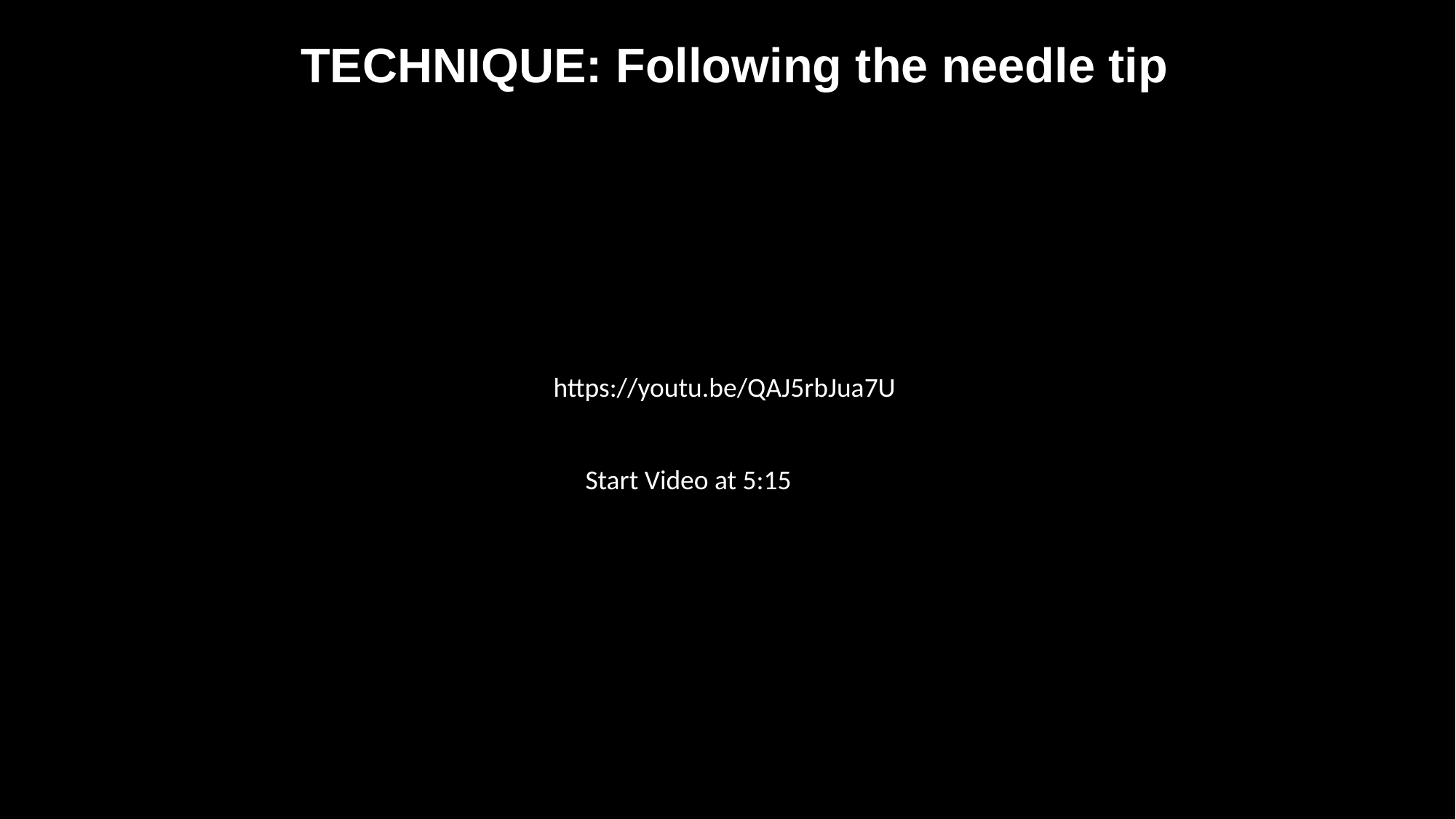

TECHNIQUE: Following the needle tip
https://youtu.be/QAJ5rbJua7U
Start Video at 5:15

## Slide 14
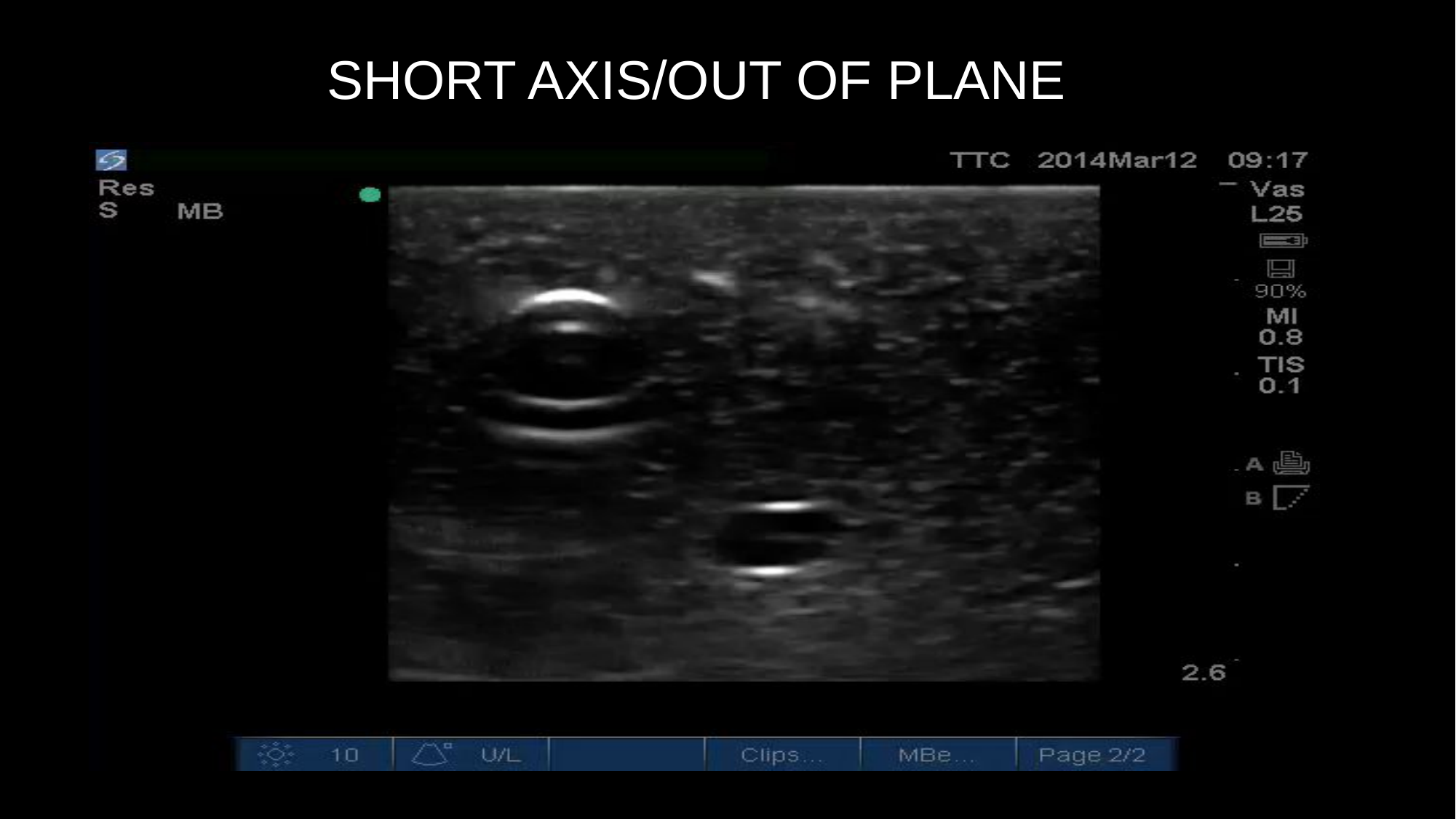

# SHORT AXIS/OUT OF PLANE

## Slide 15
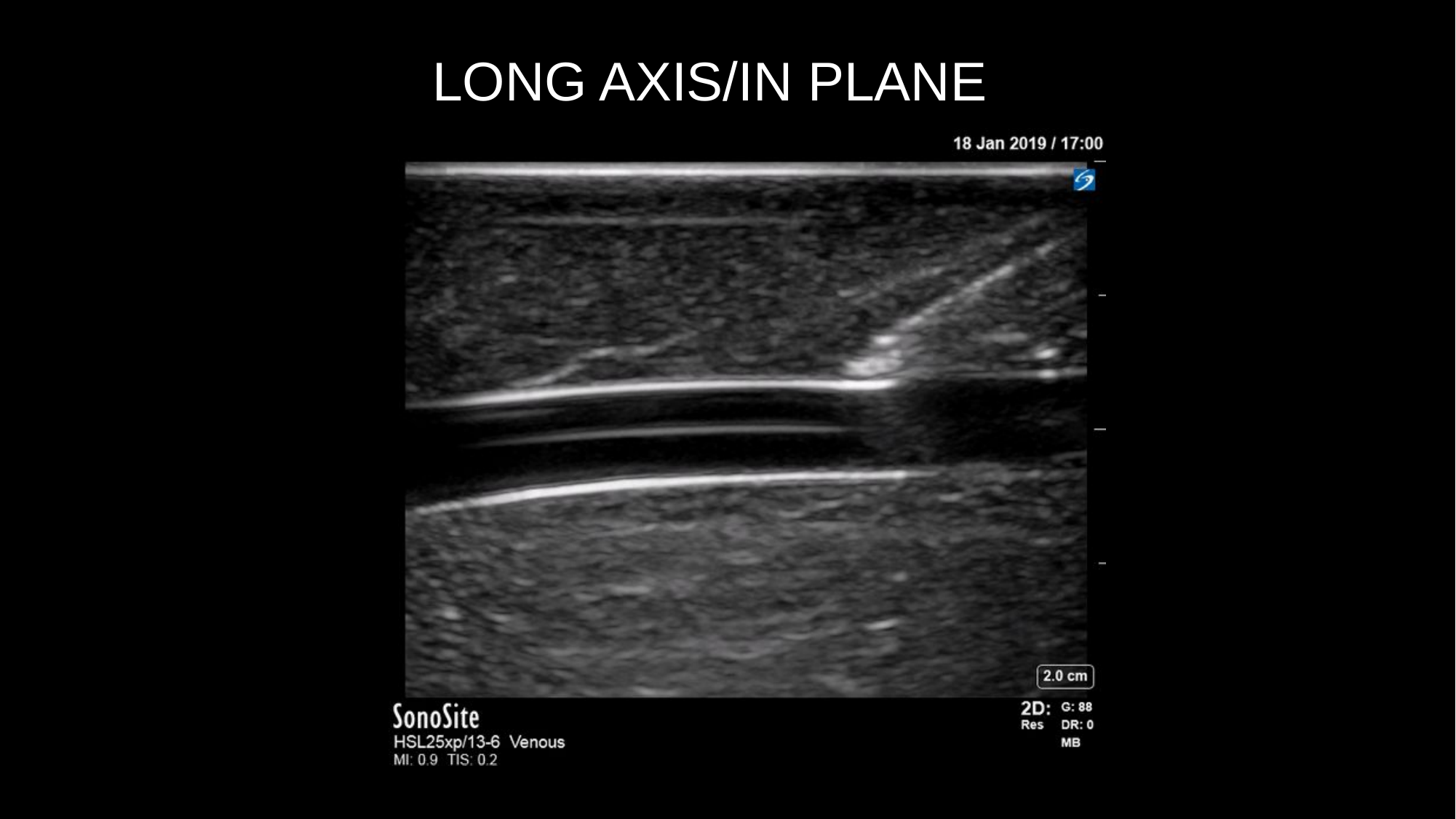

# LONG AXIS/IN PLANE

## Slide 16
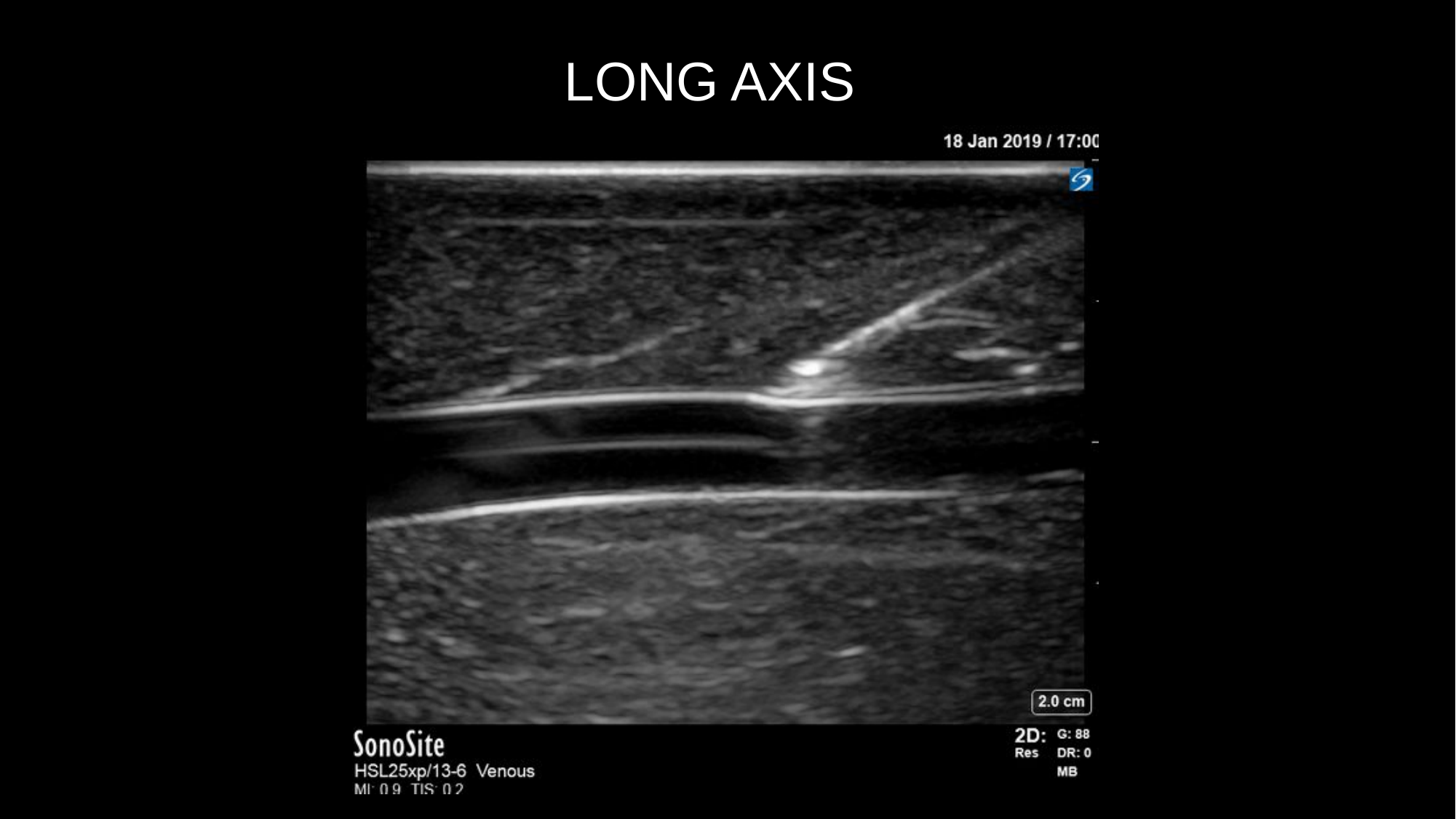

# Long Axis
LONG AXIS

## Slide 17
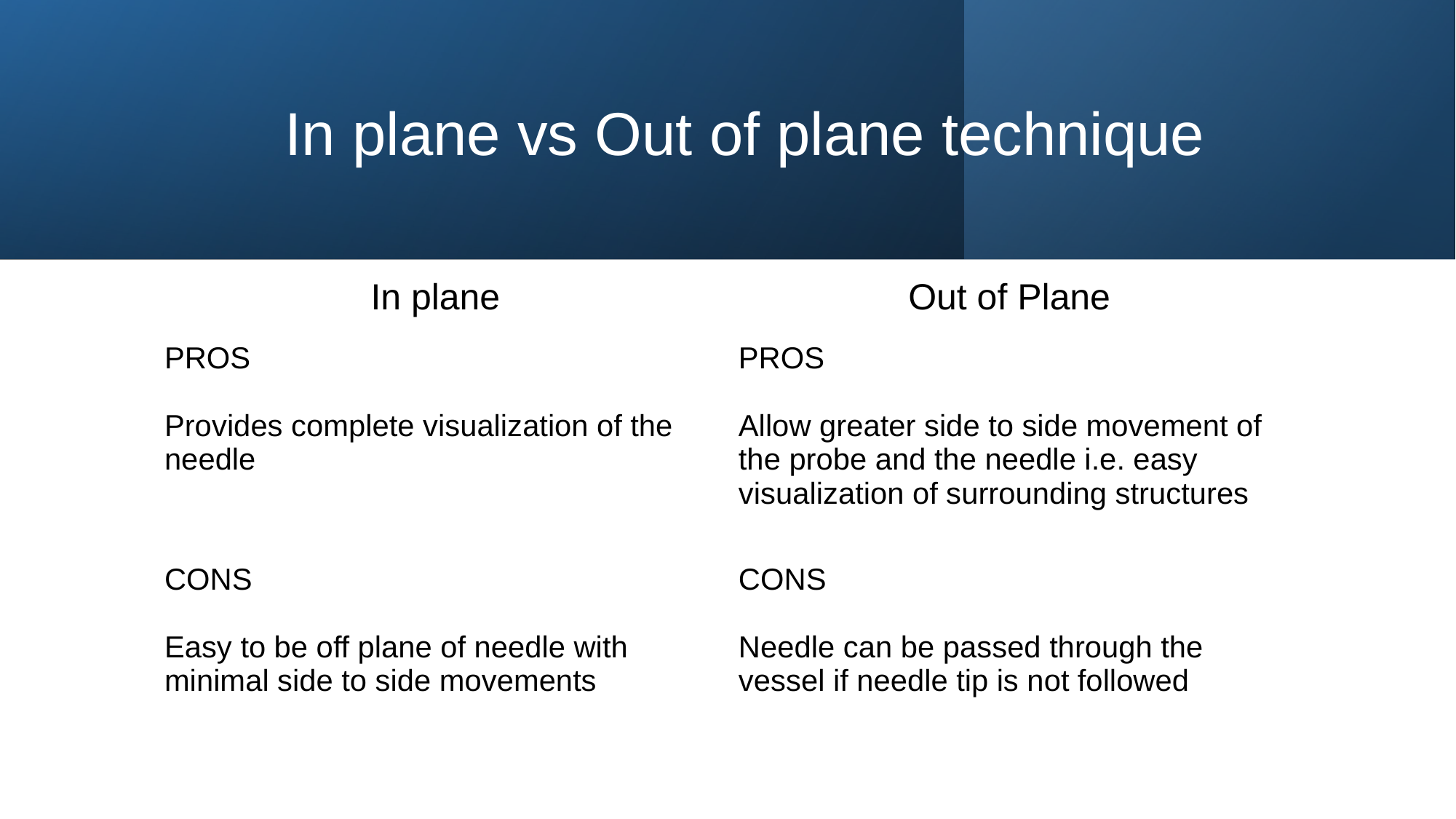

In plane vs Out of plane technique
| In plane | Out of Plane |
| --- | --- |
| PROS Provides complete visualization of the needle | PROS Allow greater side to side movement of the probe and the needle i.e. easy visualization of surrounding structures |
| CONS Easy to be off plane of needle with minimal side to side movements | CONS Needle can be passed through the vessel if needle tip is not followed |

## Slide 18
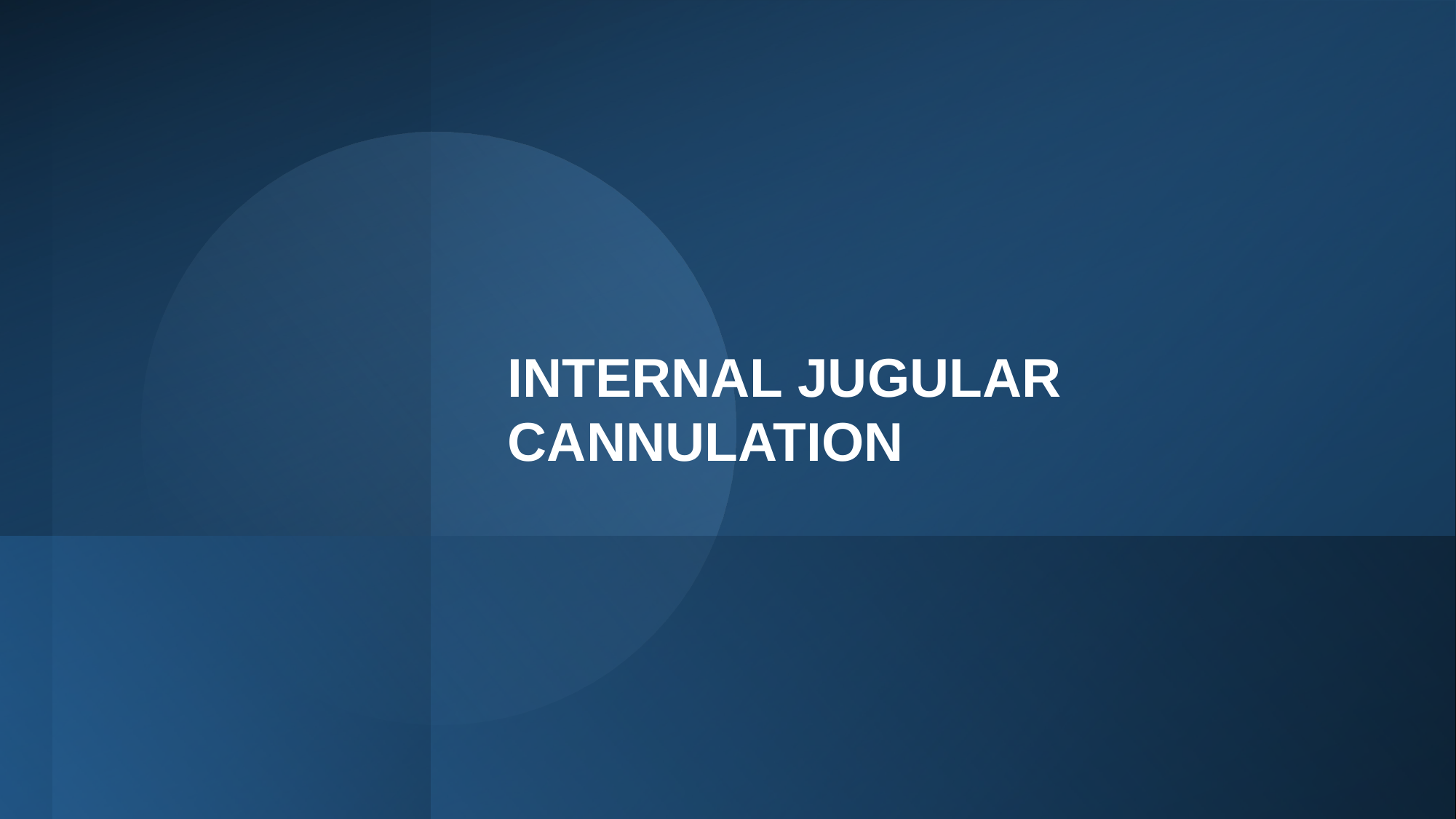

INTERNAL JUGULAR
CANNULATION

## Slide 19
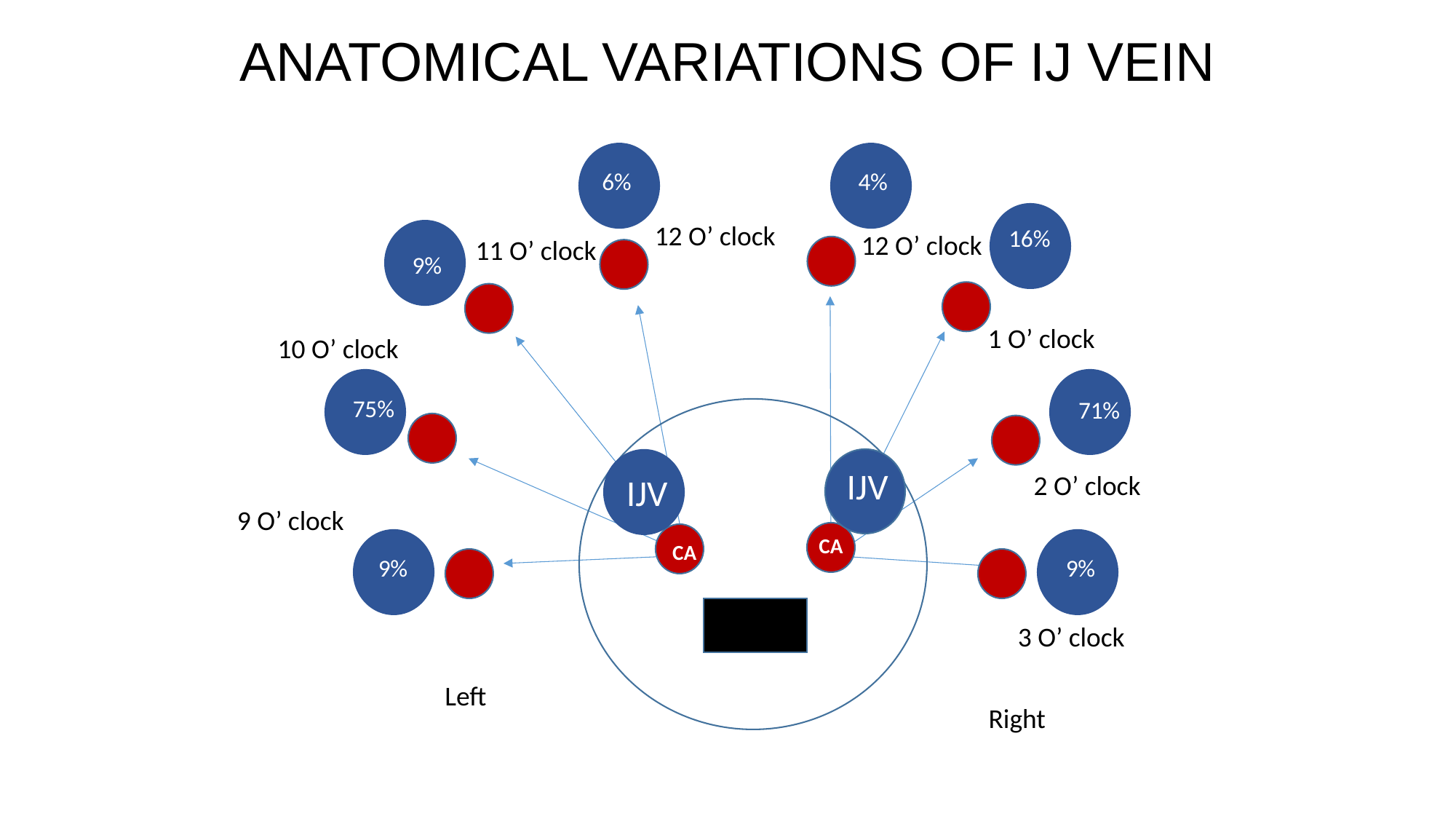

ANATOMICAL VARIATIONS OF IJ VEIN
6%
4%
12 O’ clock
16%
12 O’ clock
11 O’ clock
9%
1 O’ clock
10 O’ clock
75%
71%
IJV
2 O’ clock
IJV
9 O’ clock
CA
CA
9%
9%
3 O’ clock
Left
Right

## Slide 20
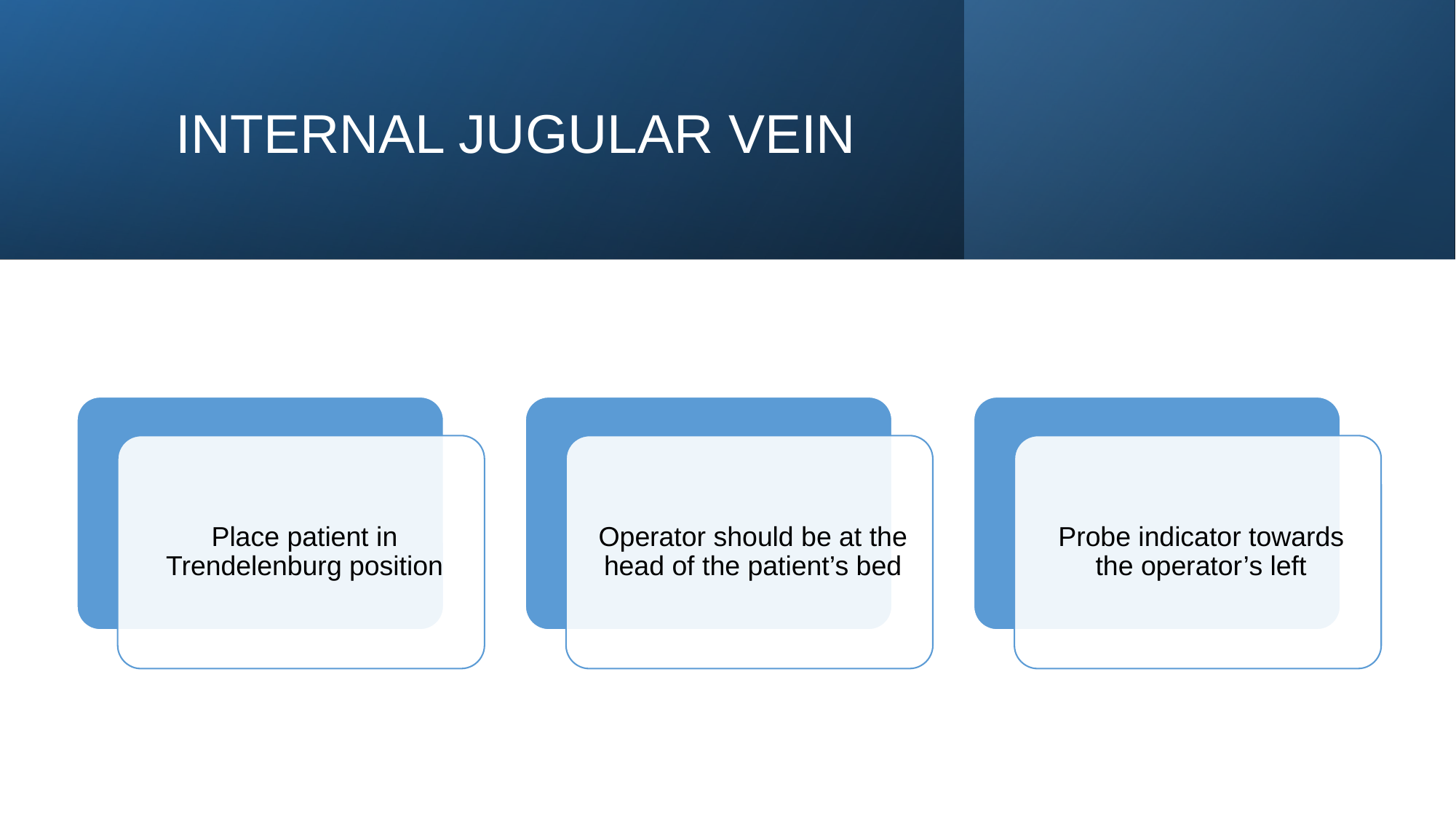

INTERNAL JUGULAR VEIN

## Slide 21
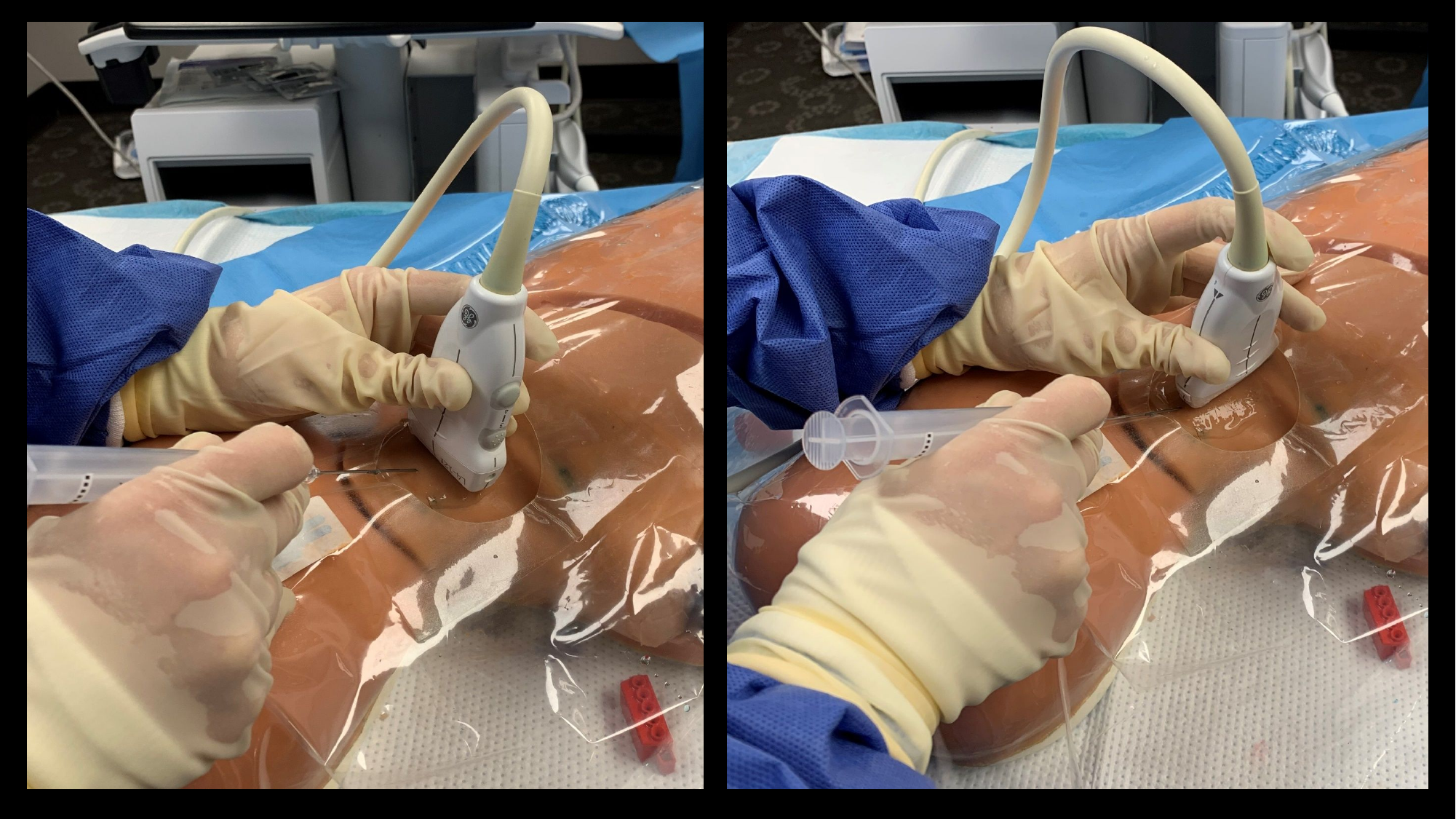

## Slide 22
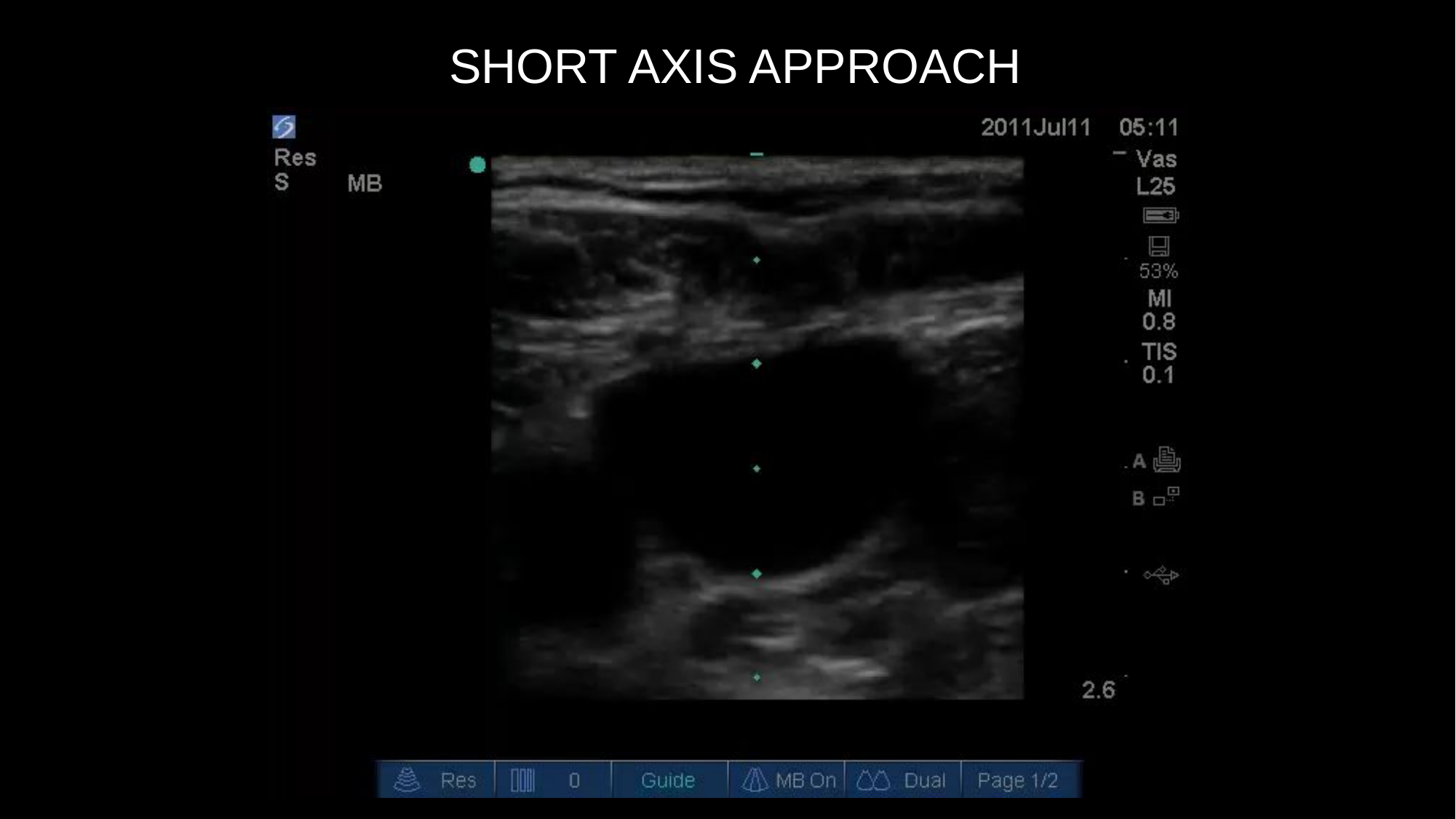

Short Axis Approach

## Slide 23
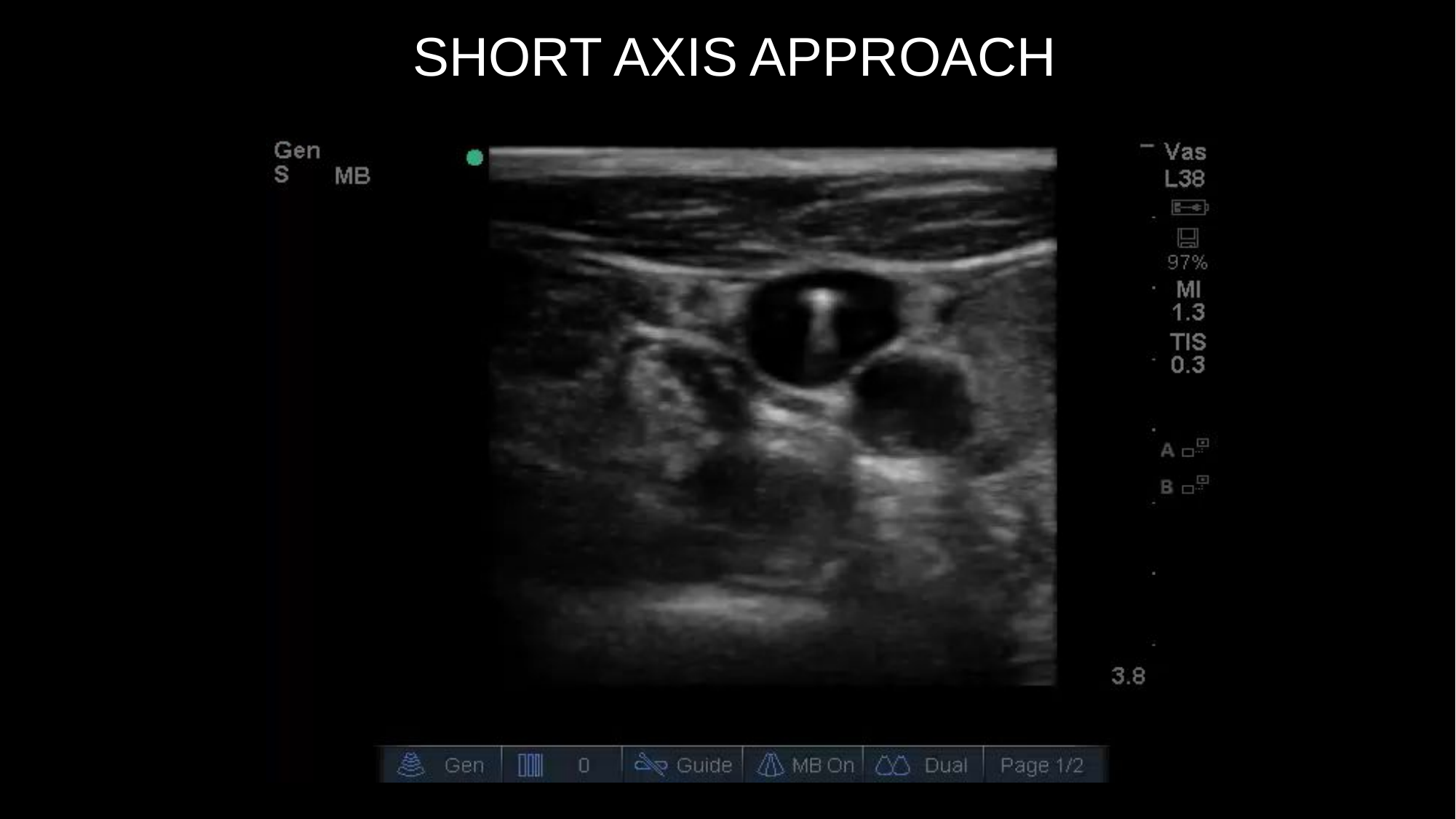

Short Axis Approach

## Slide 24
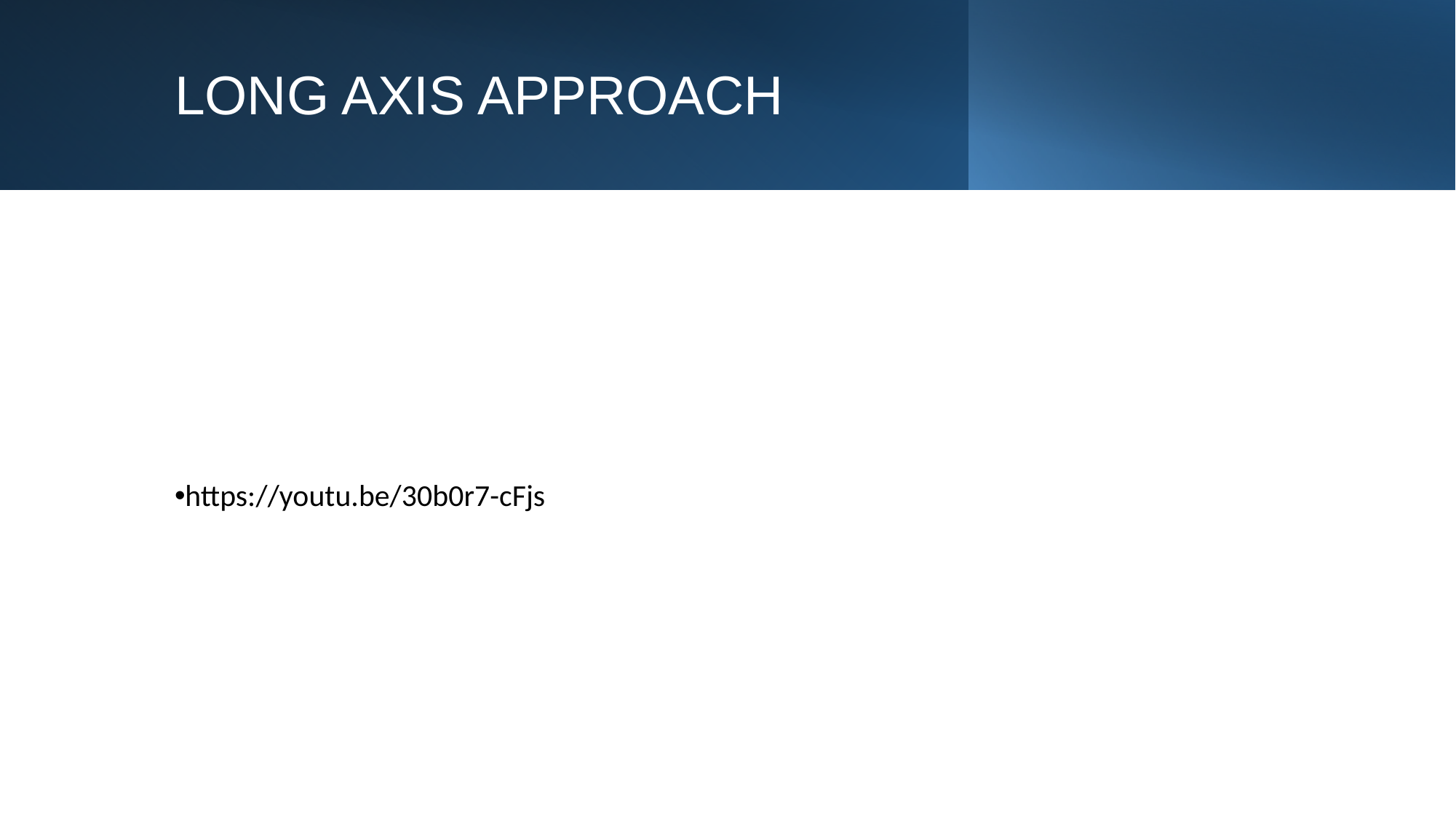

# LONG AXIS APPROACH
https://youtu.be/30b0r7-cFjs

## Slide 25
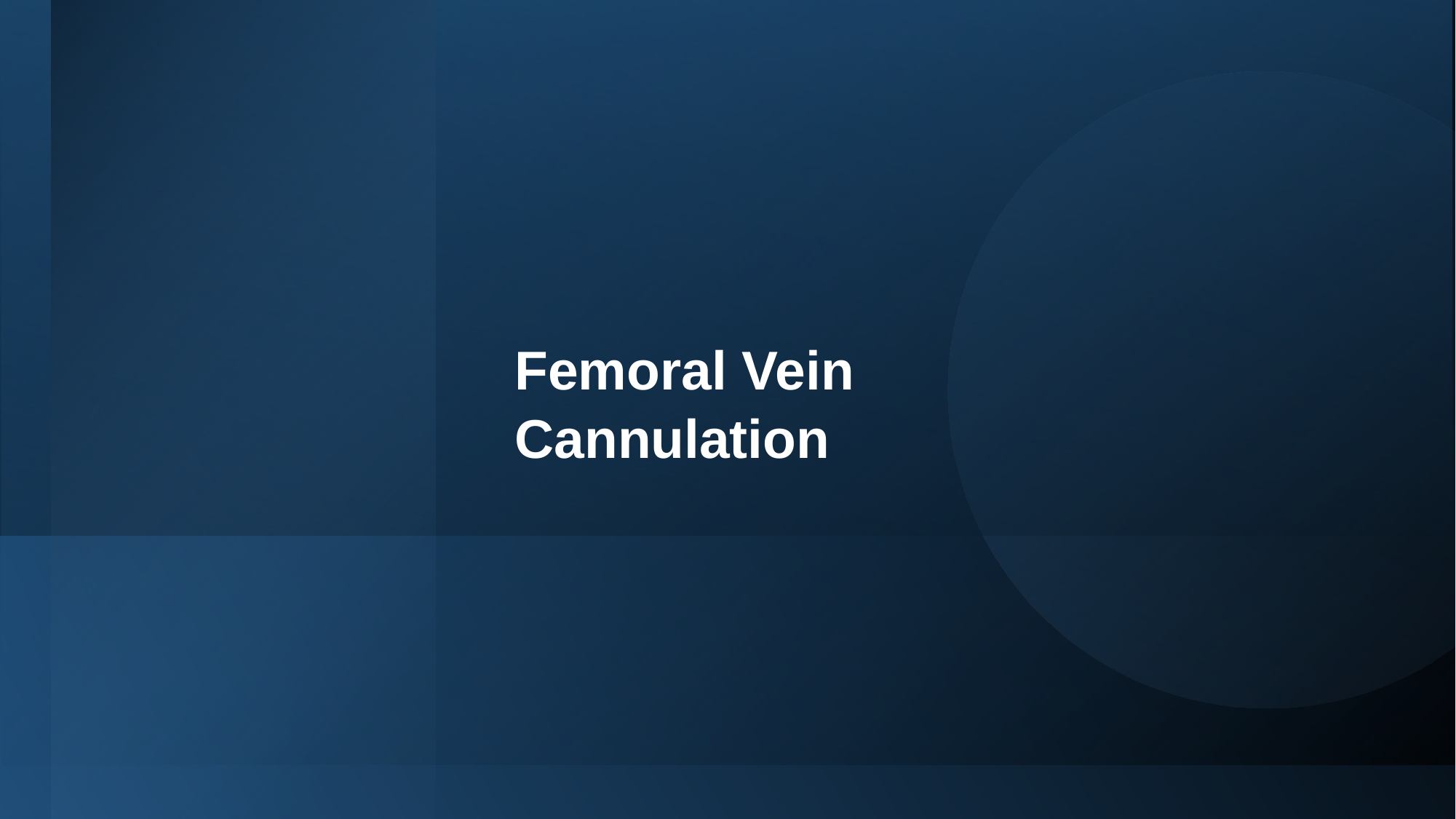

Femoral Vein
Cannulation

## Slide 26
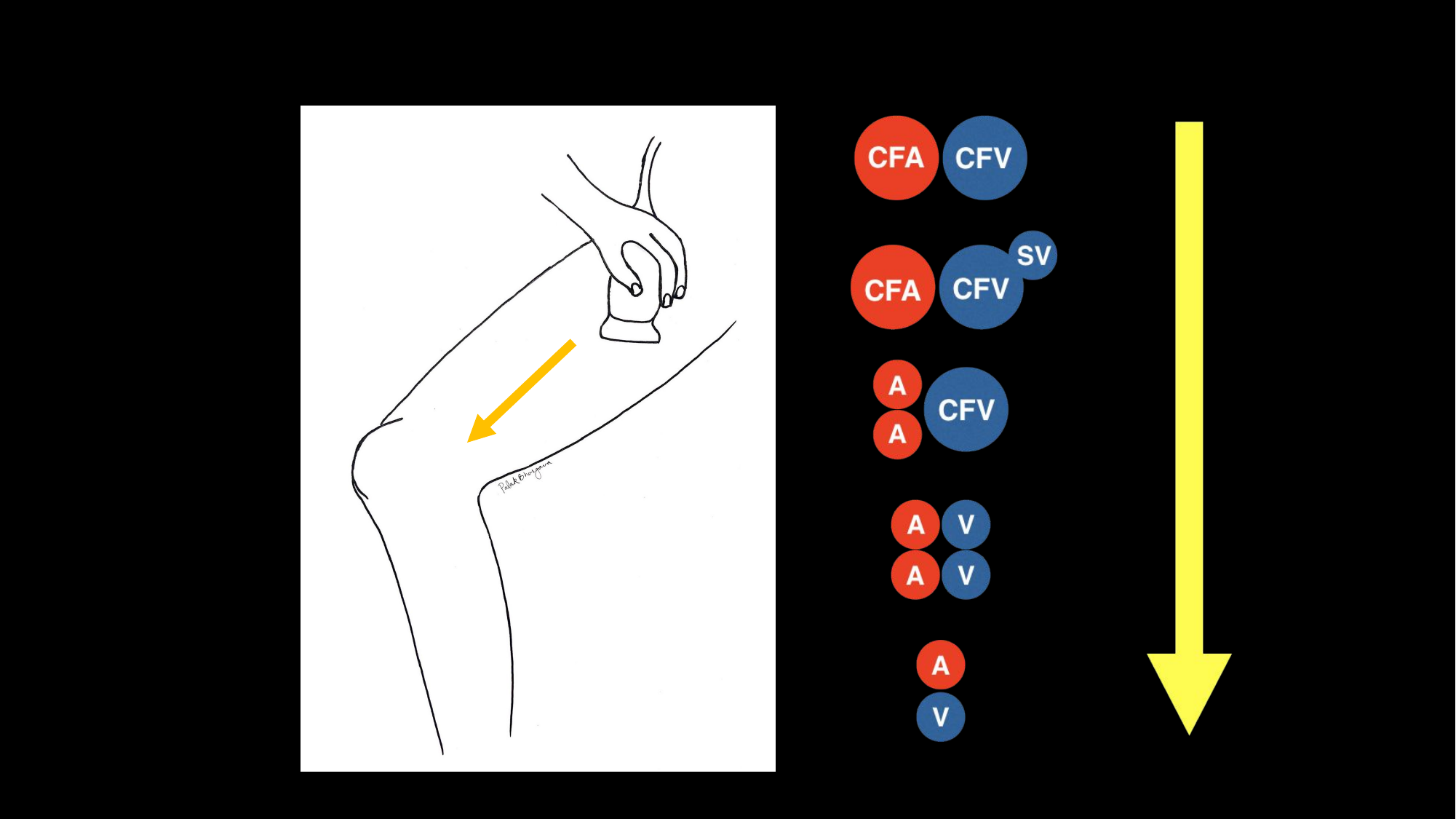

## Slide 27
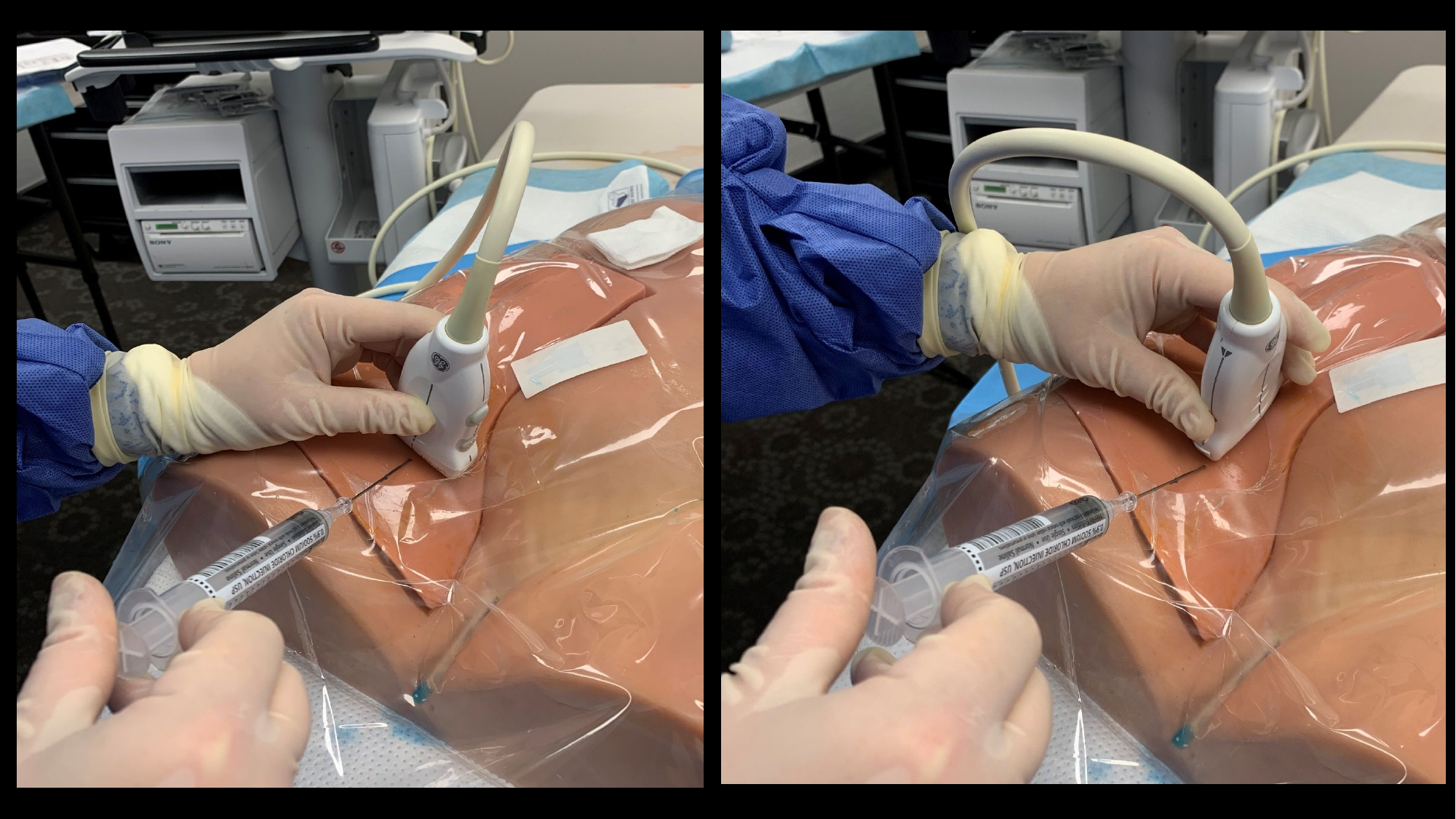

## Slide 28
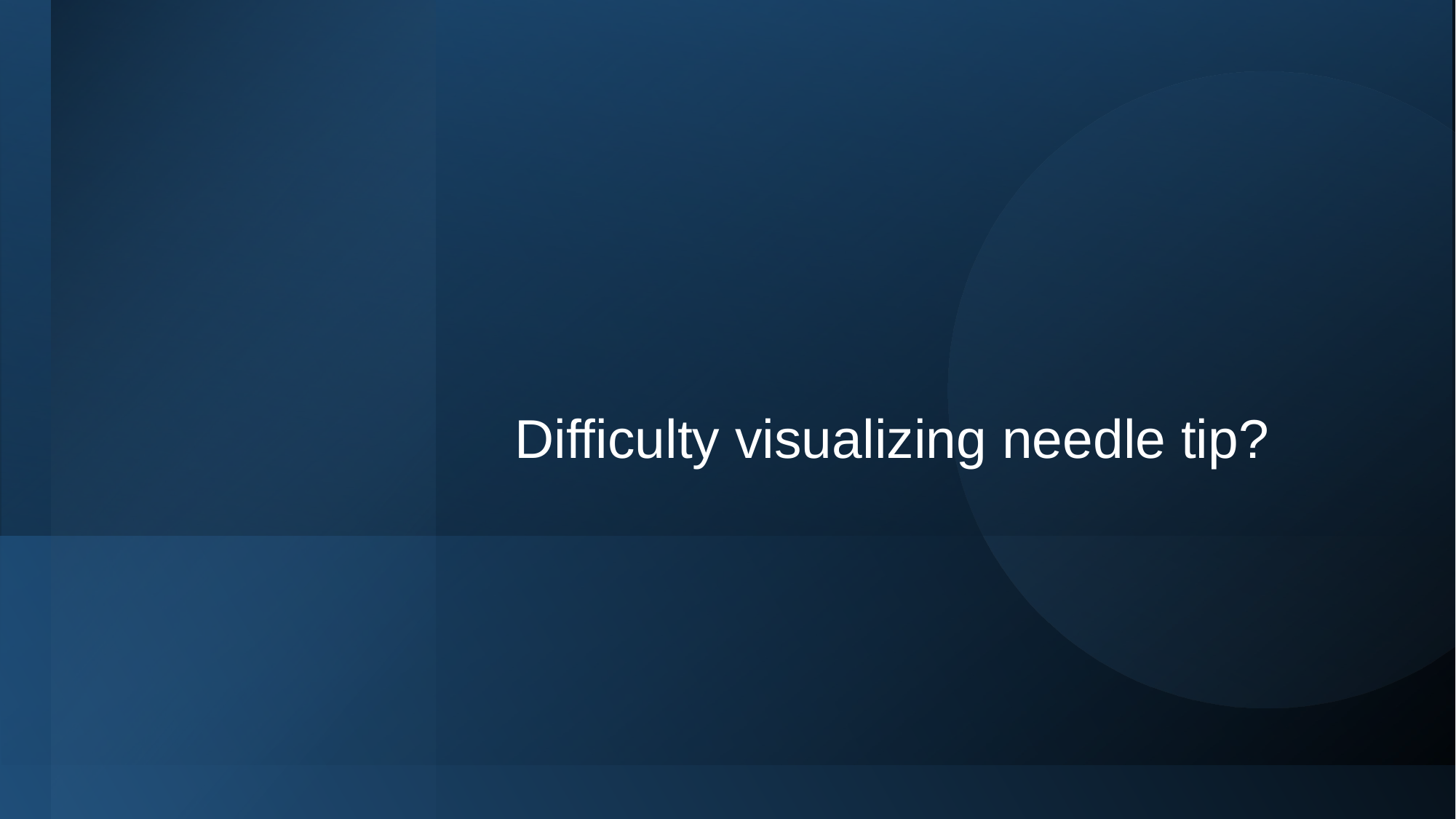

# Difficulty visualizing needle tip?

## Slide 29
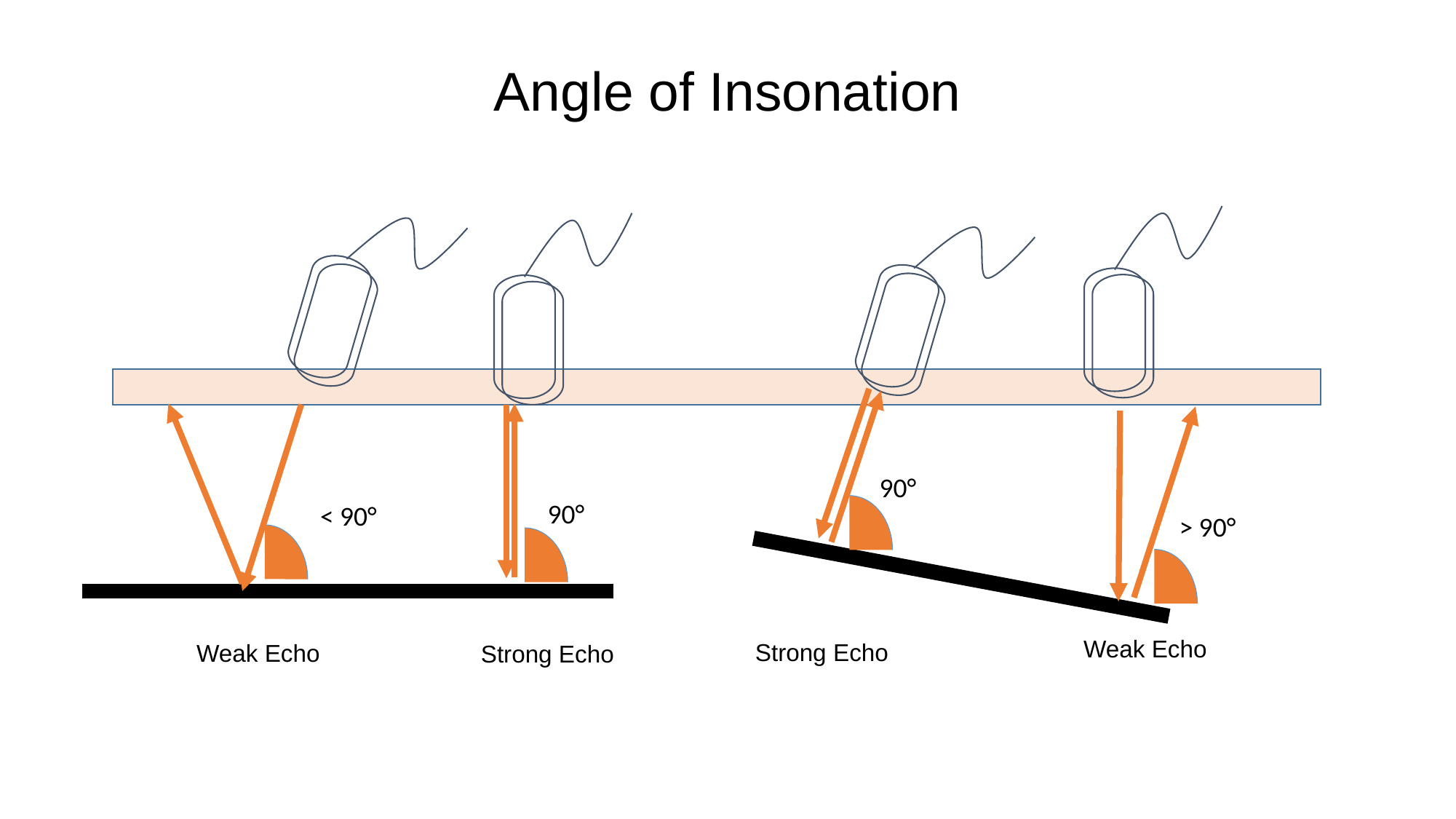

# Angle of Insonation
90°
90°
< 90°
> 90°
Weak Echo
Strong Echo
Weak Echo
Strong Echo

## Slide 30
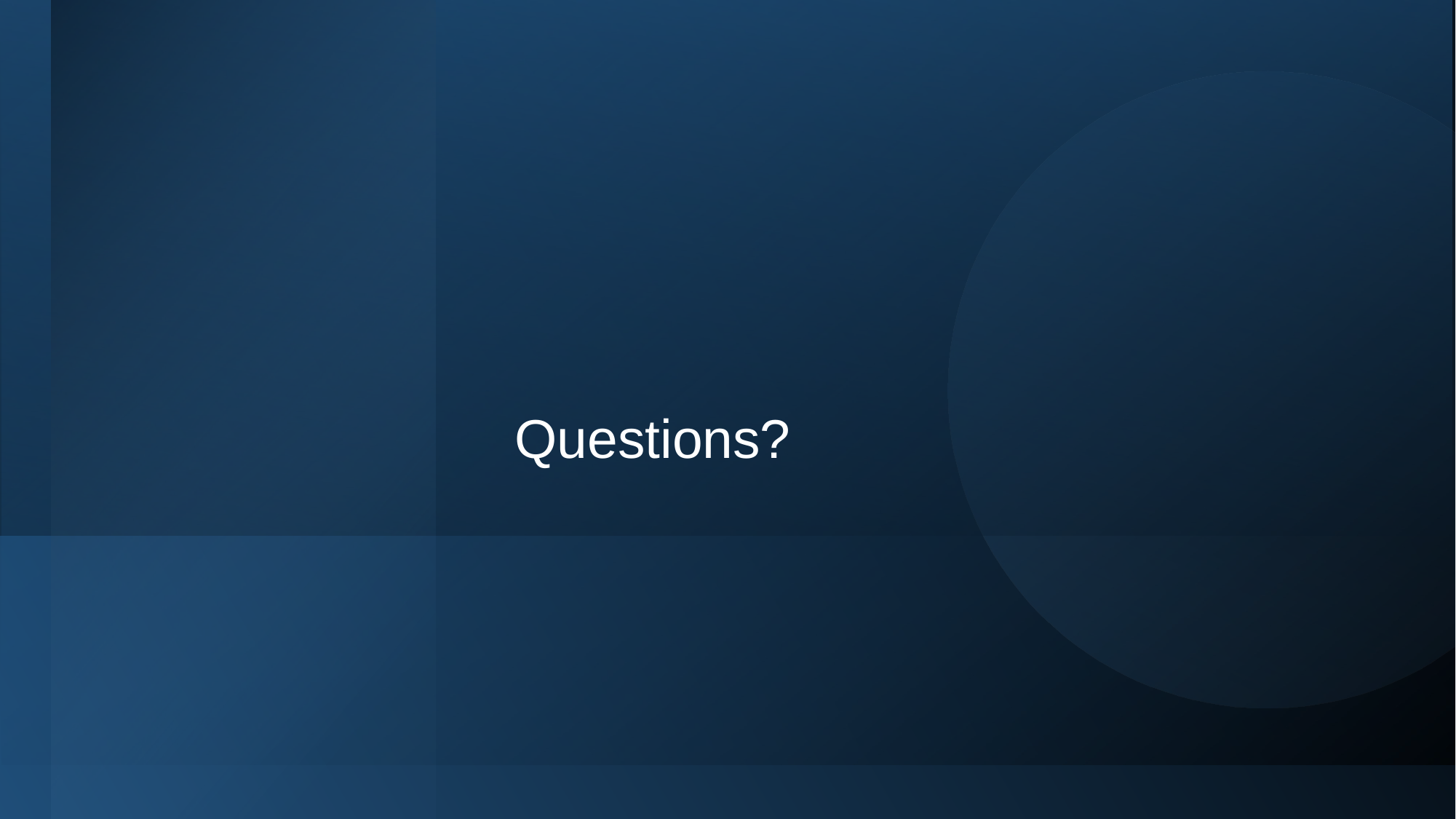

# Questions?
