# Supplementary material for: A point-of-care ultrasound education curriculum for pediatric critical care medicine
Source: Ultrasound J. 2022 Oct 31;14:44. doi: 10.1186/s13089-022-00290-6 (PMC9622960; doi:10.1186/s13089-022-00290-6)
Supplement: Supplementary file 5 — Additional file 5. Ultrasound-guided non-vascular access and advanced procedures [file 13089_2022_290_MOESM5_ESM.pptx]

## Slide 1
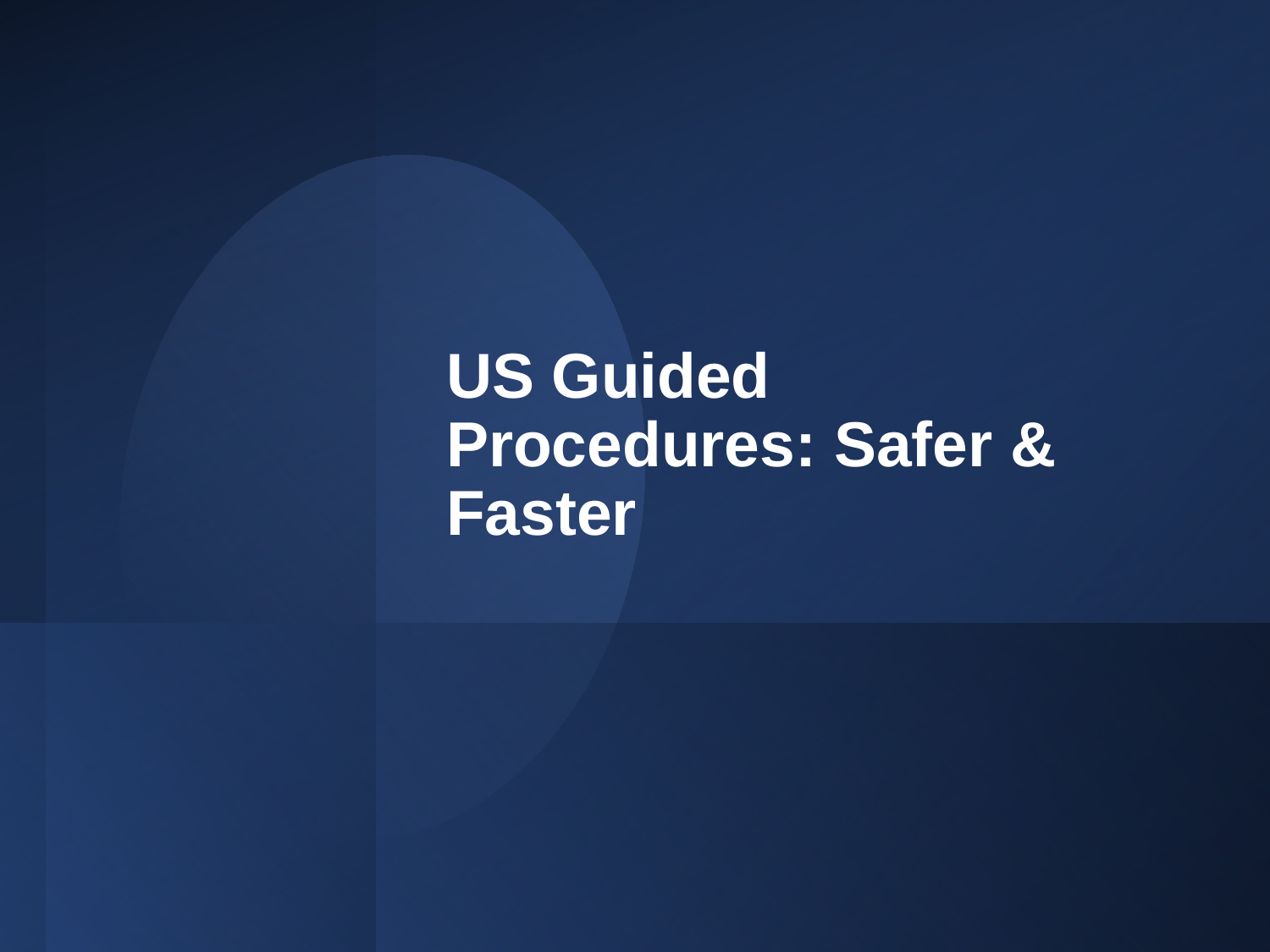

US Guided Procedures: Safer & Faster

## Slide 2
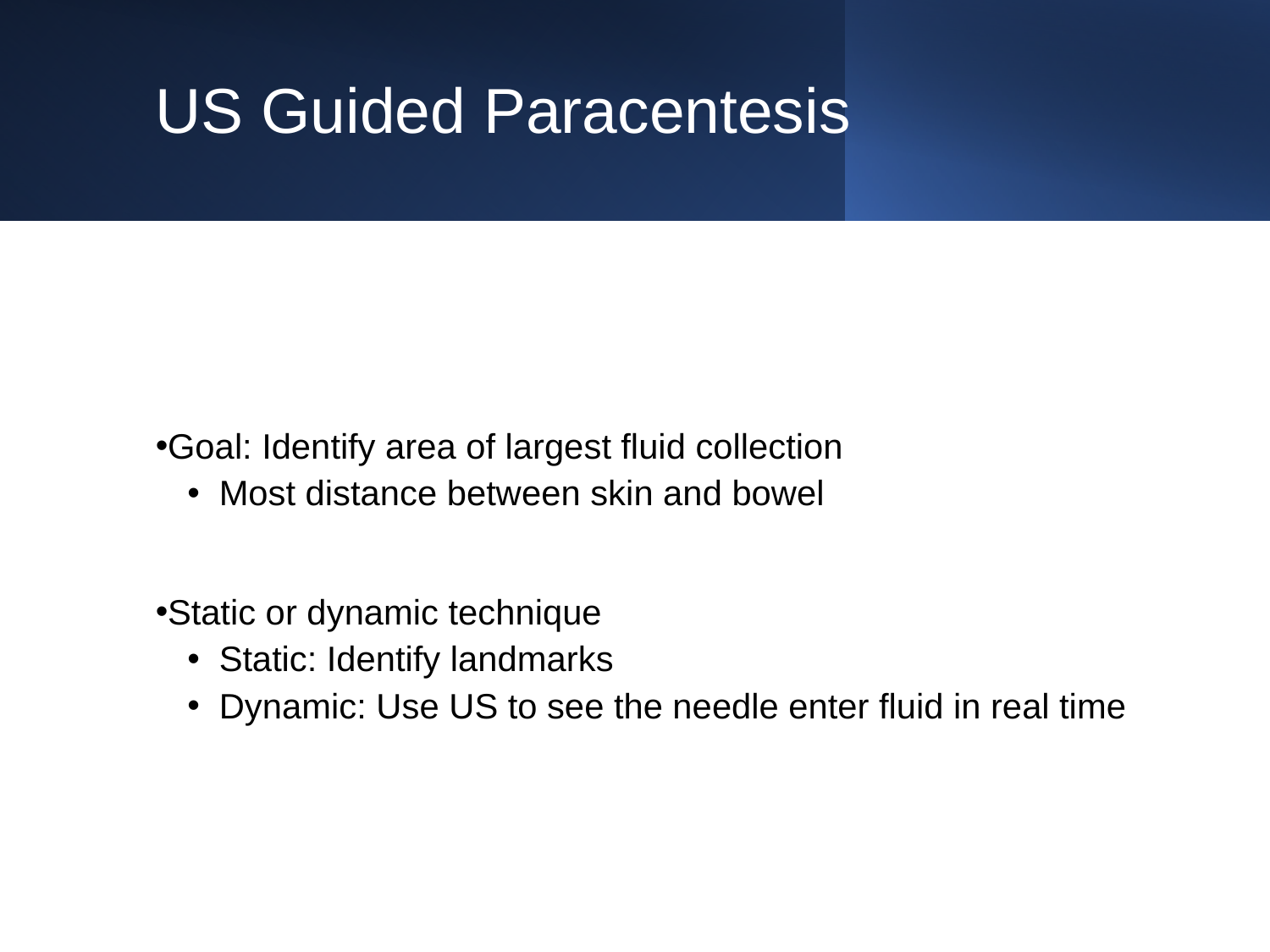

# US Guided Paracentesis
Goal: Identify area of largest fluid collection
Most distance between skin and bowel
Static or dynamic technique
Static: Identify landmarks
Dynamic: Use US to see the needle enter fluid in real time

## Slide 3
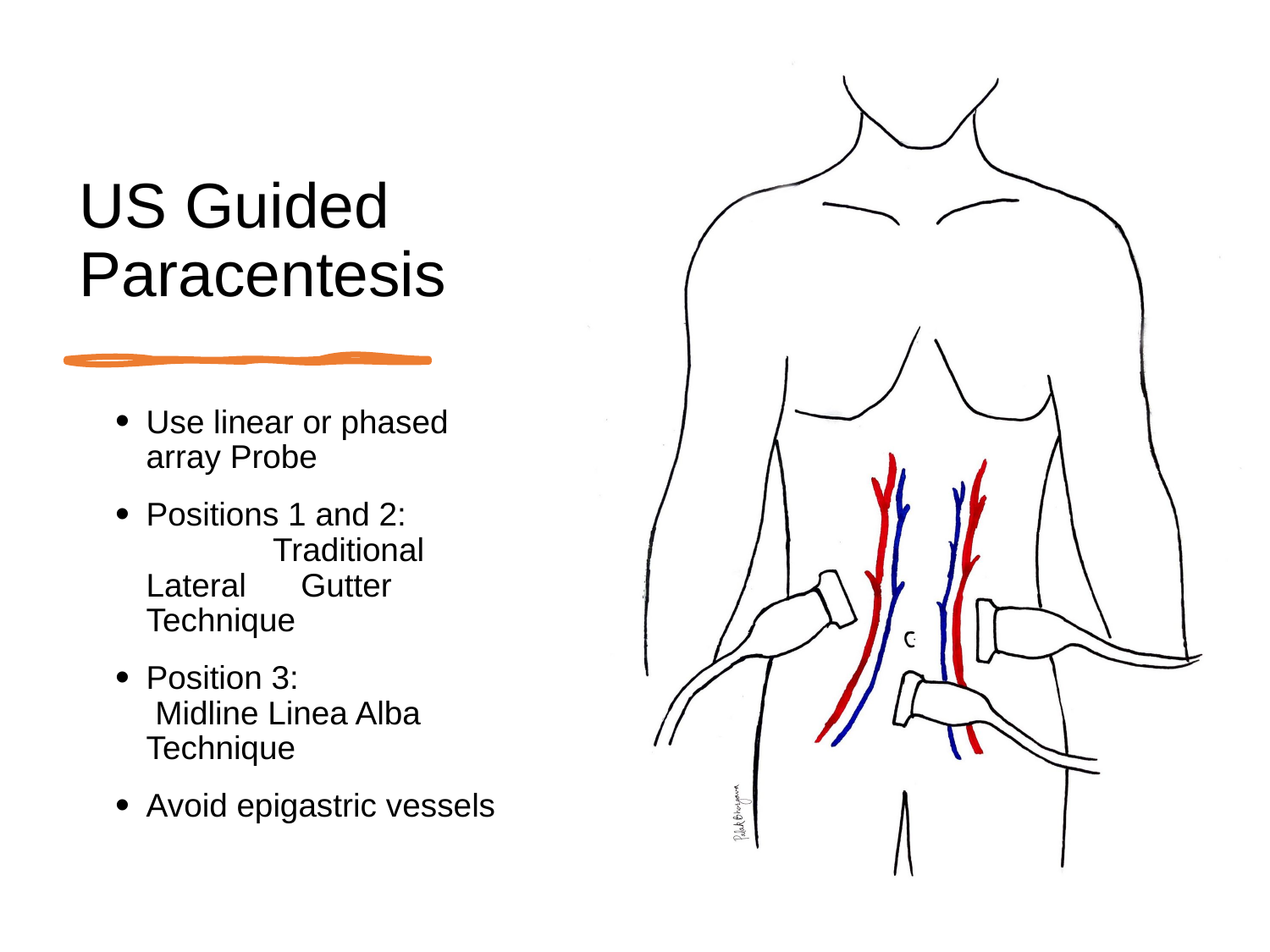

# US Guided Paracentesis
Use linear or phased array Probe
Positions 1 and 2: Traditional Lateral Gutter Technique
Position 3: Midline Linea Alba Technique
Avoid epigastric vessels

## Slide 4
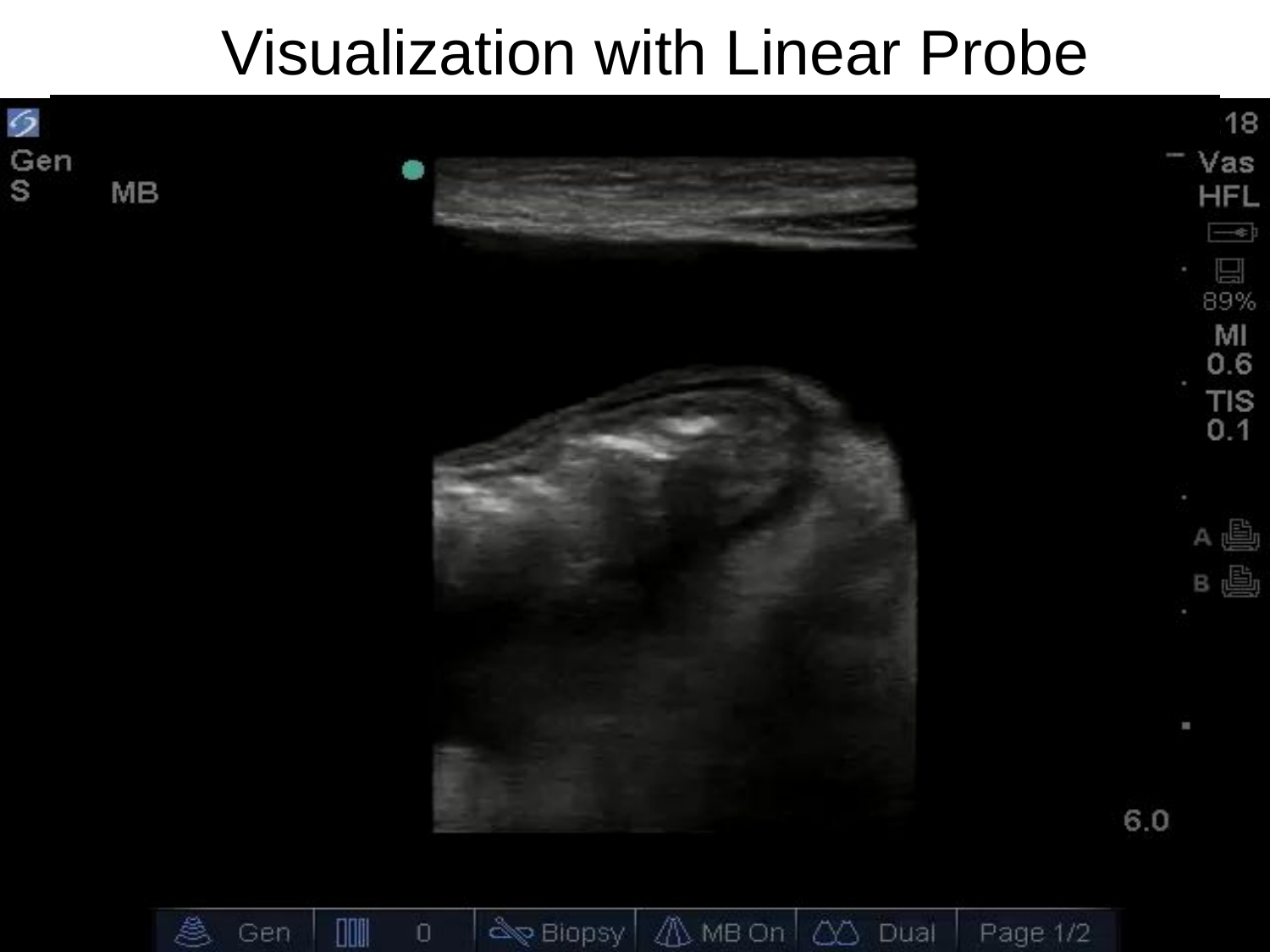

# Visualization with Linear Probe

## Slide 5
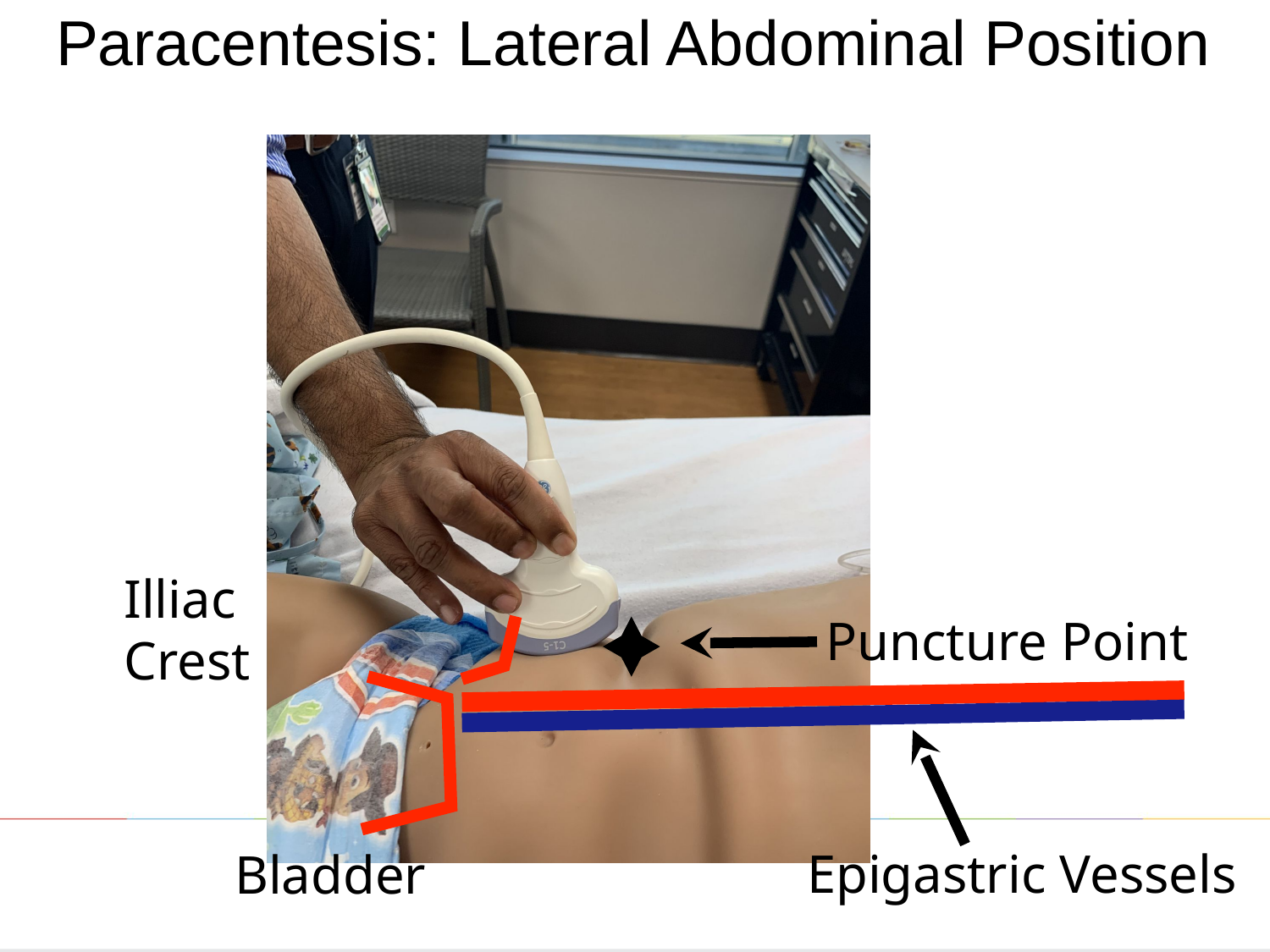

Paracentesis: Lateral Abdominal Position
Illiac
Crest
Puncture Point
Epigastric Vessels
Bladder

## Slide 6
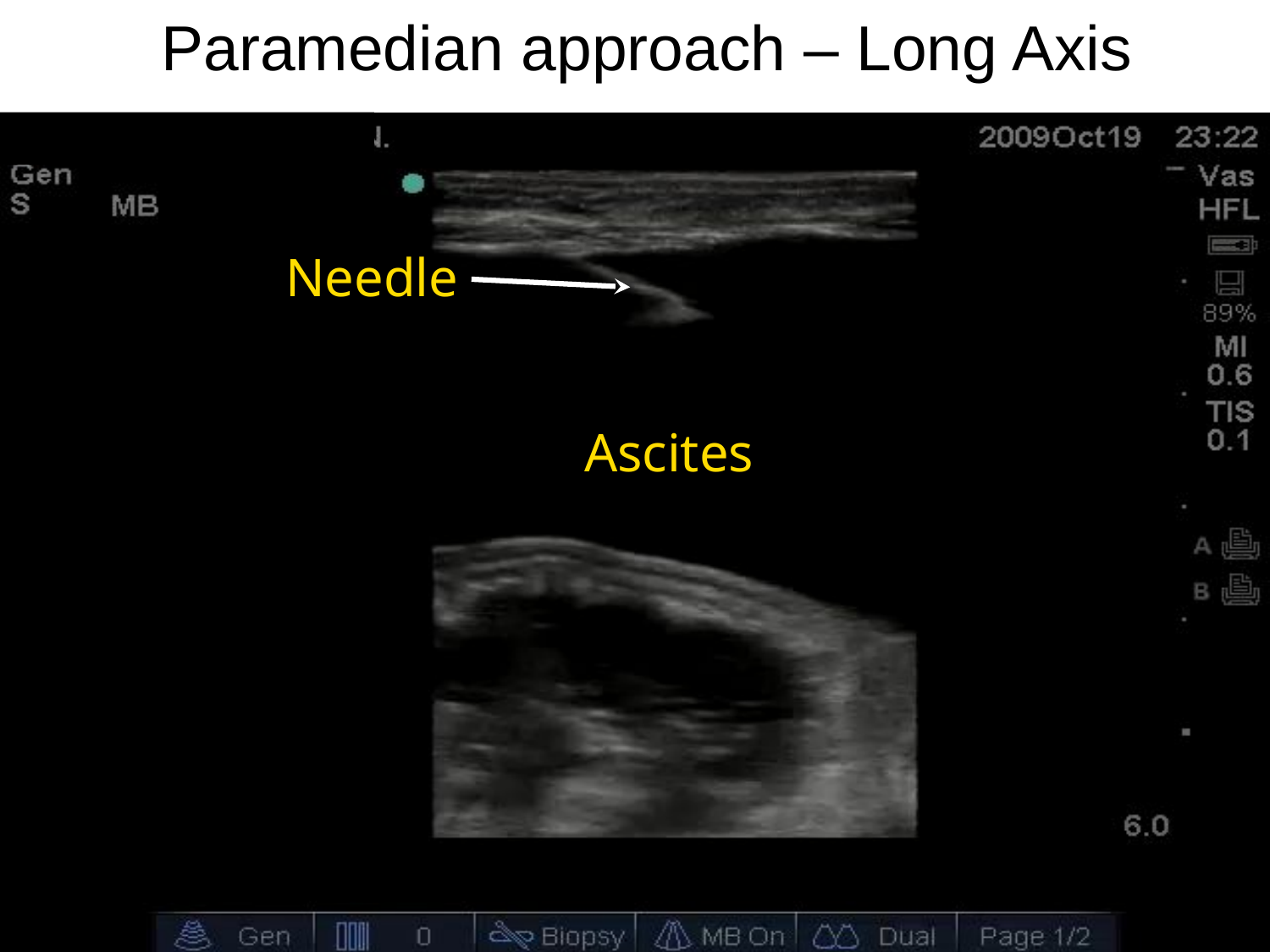

Paramedian approach – Long Axis
Needle
Ascites

## Slide 7
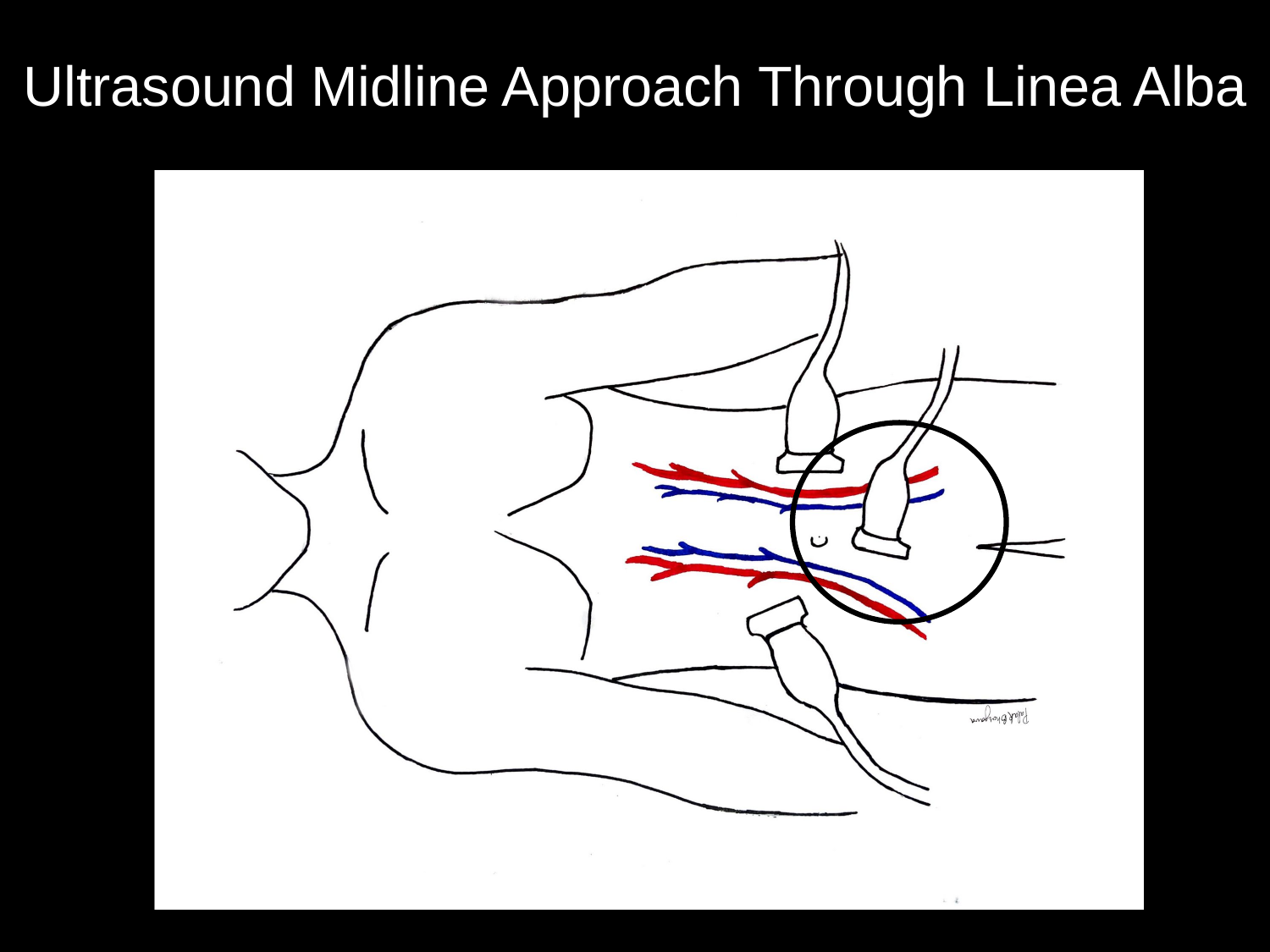

Ultrasound Midline Approach Through Linea Alba

## Slide 8
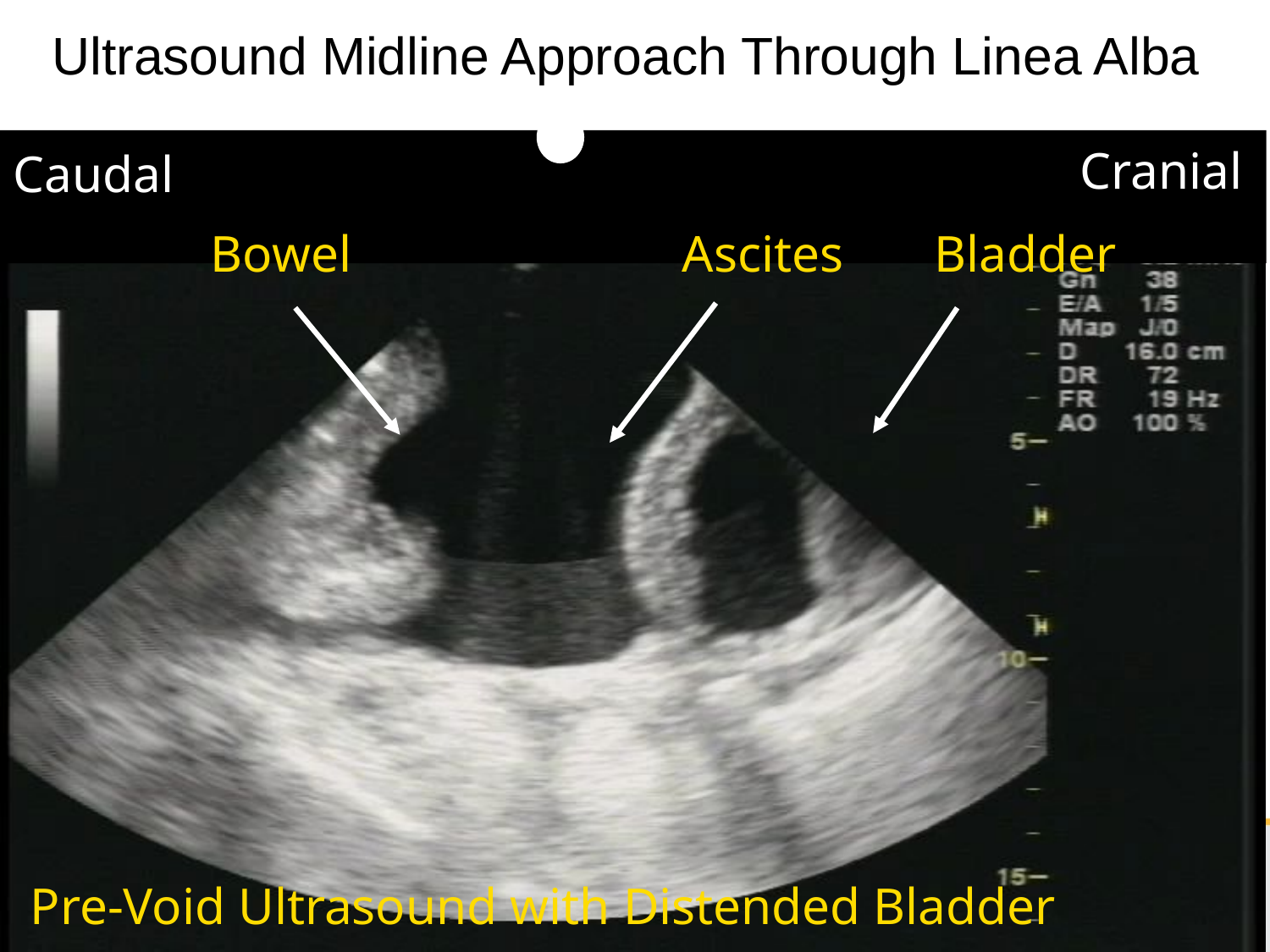

Ultrasound Midline Approach Through Linea Alba
Cranial
Caudal
Bowel
Ascites
Bladder
Pre-Void Ultrasound with Distended Bladder

## Slide 9
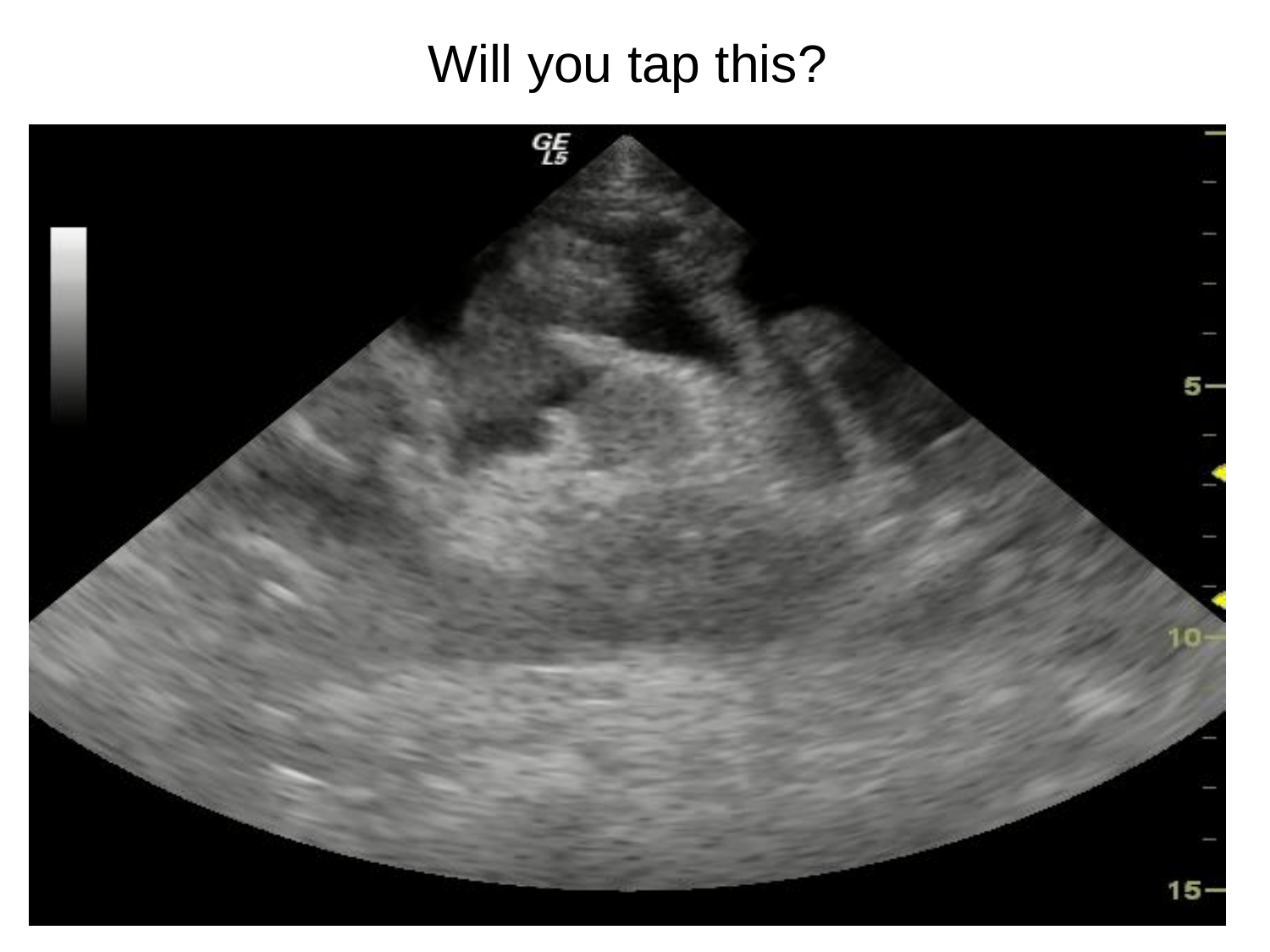

# Will you tap this?

## Slide 10
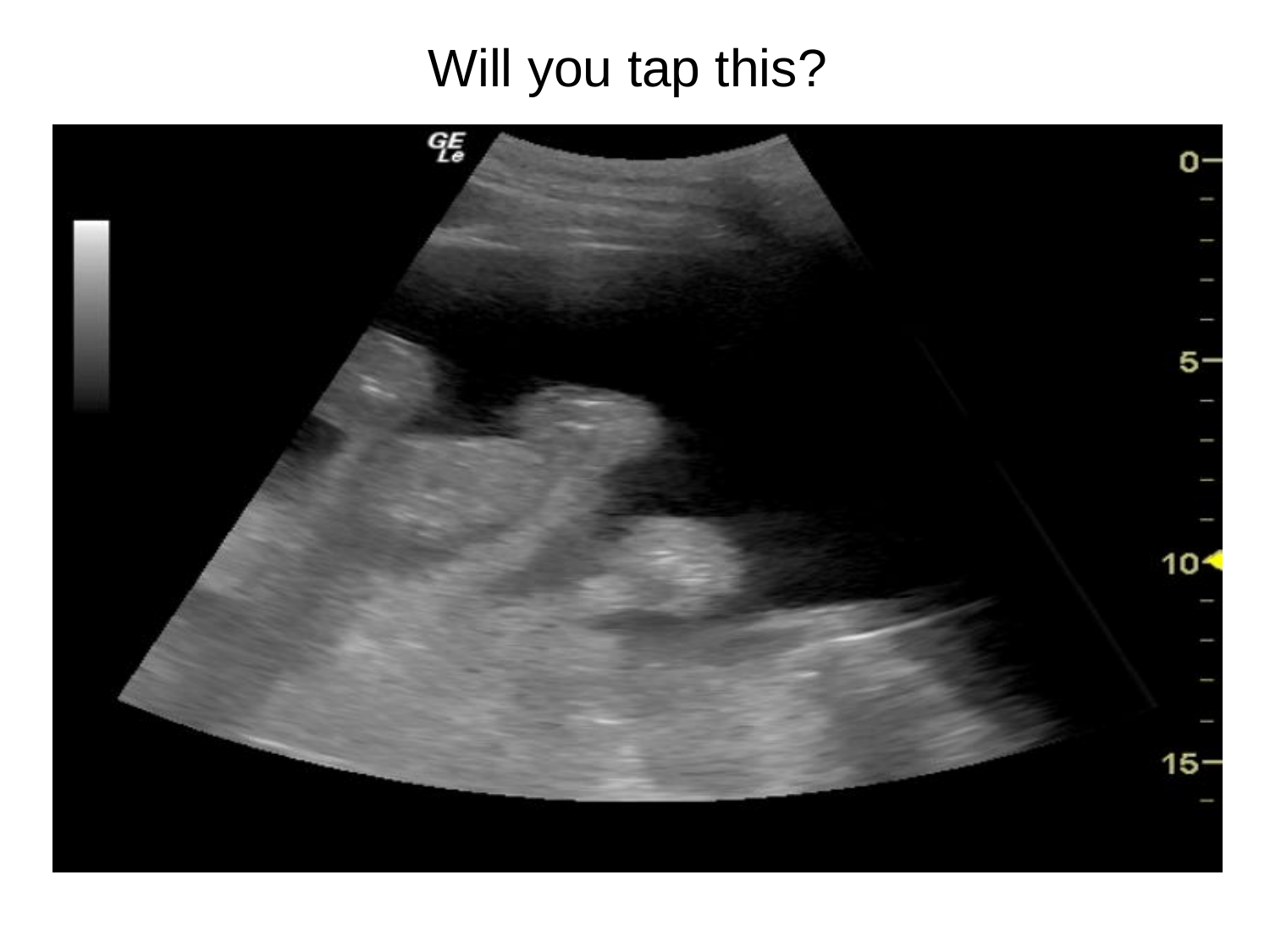

# Will you tap this?

## Slide 11
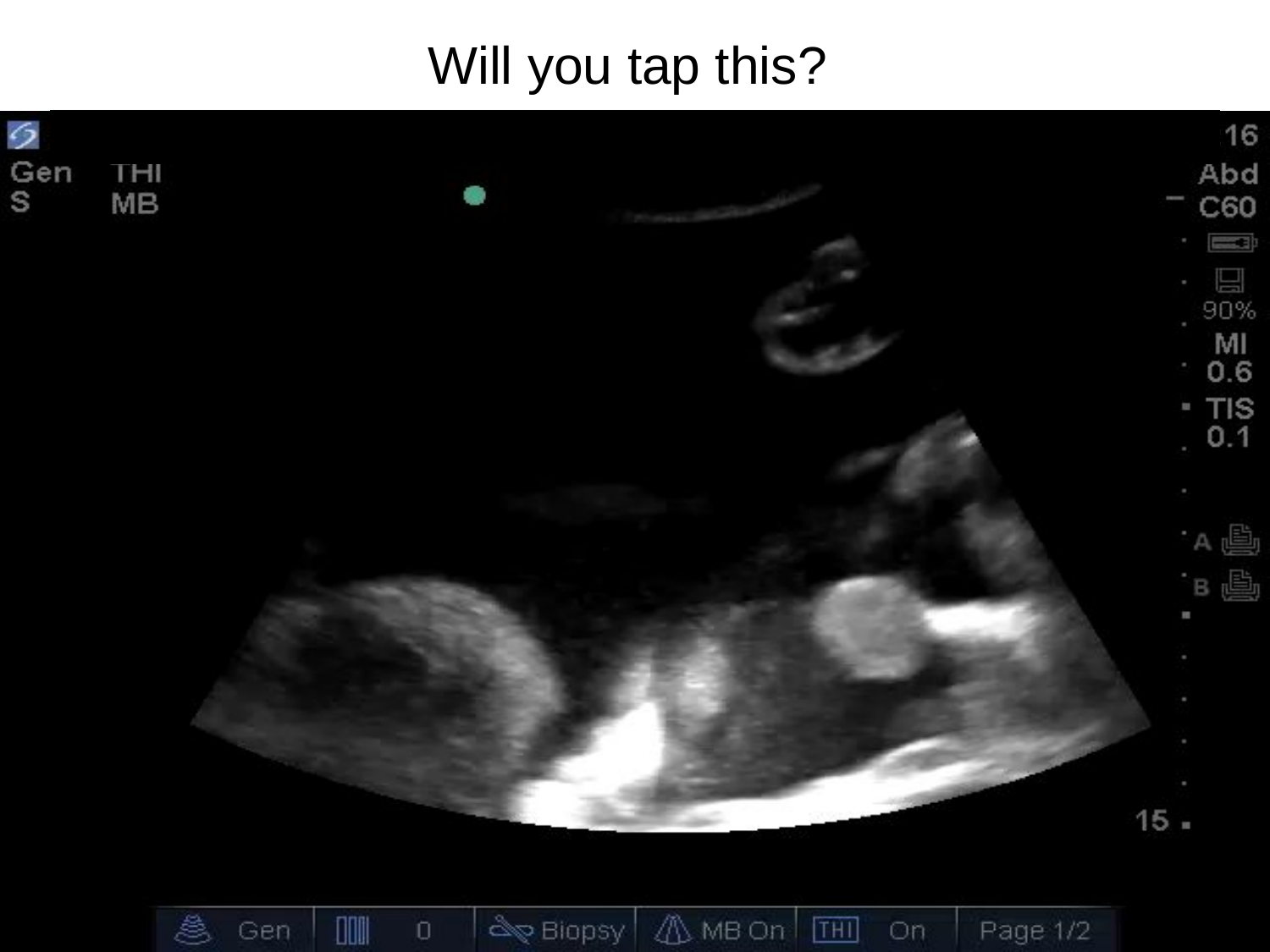

# Will you tap this?

## Slide 12
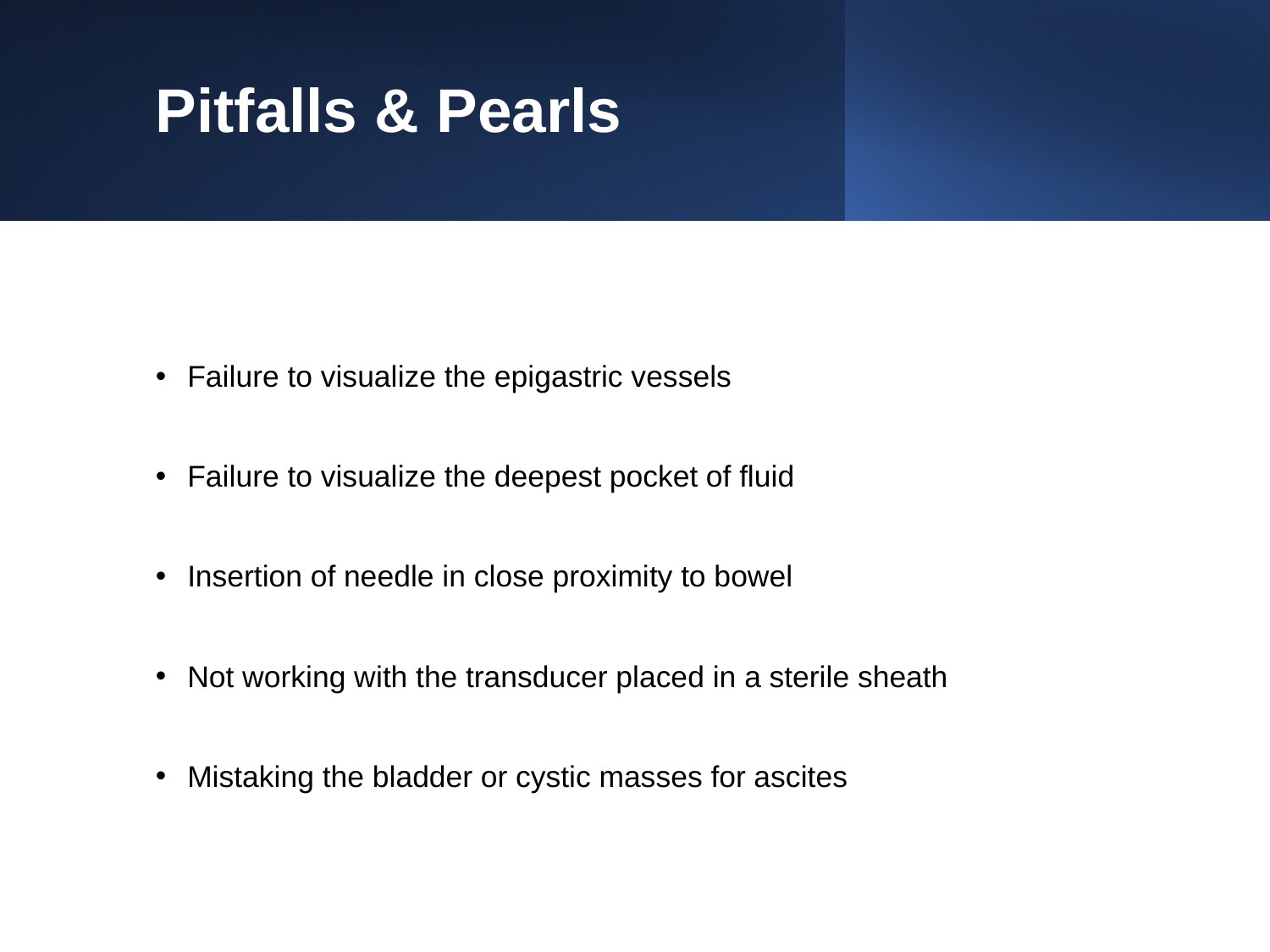

Pitfalls & Pearls
Failure to visualize the epigastric vessels
Failure to visualize the deepest pocket of fluid
Insertion of needle in close proximity to bowel
Not working with the transducer placed in a sterile sheath
Mistaking the bladder or cystic masses for ascites

## Slide 13
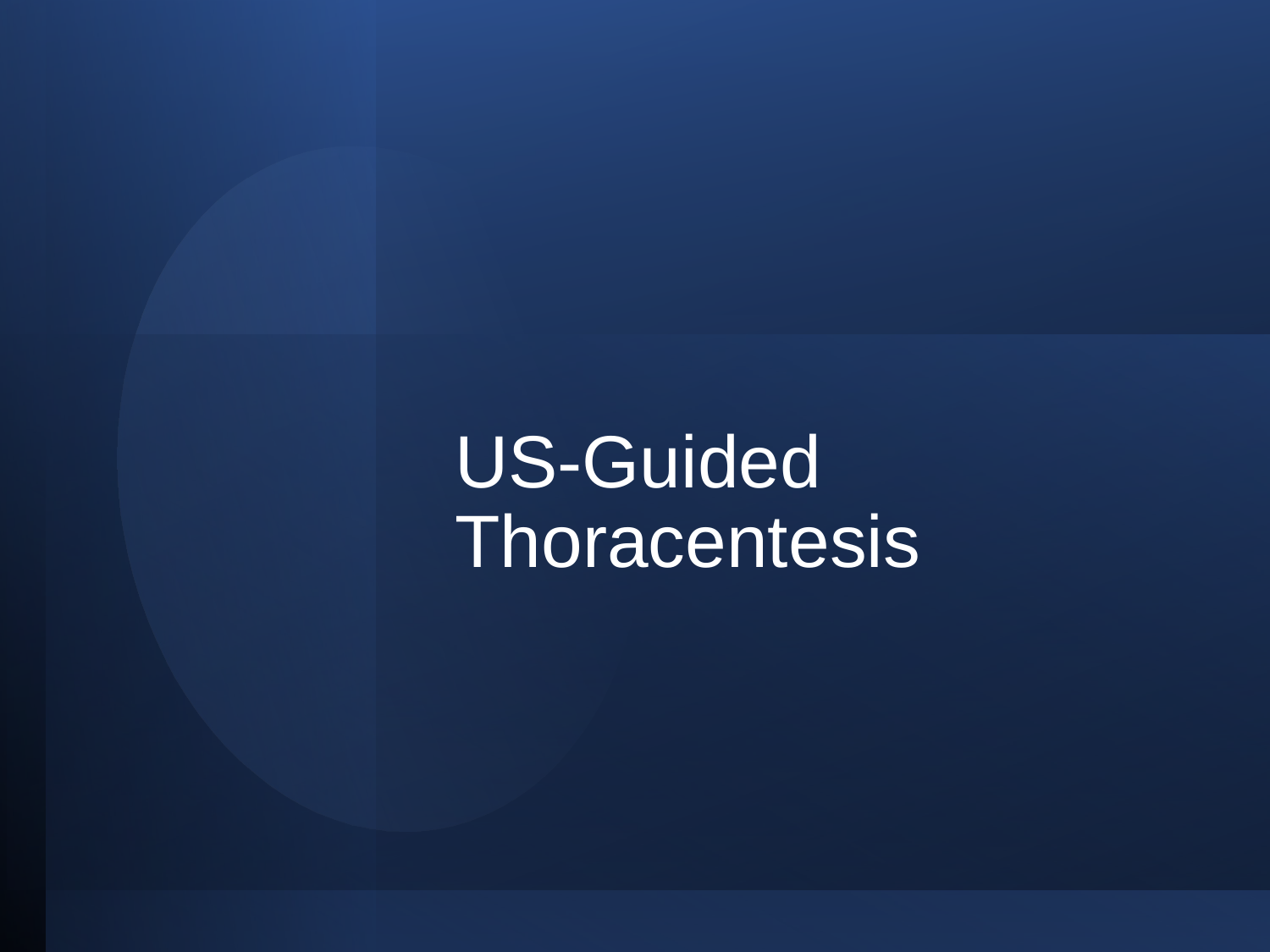

# US-Guided Thoracentesis

## Slide 14
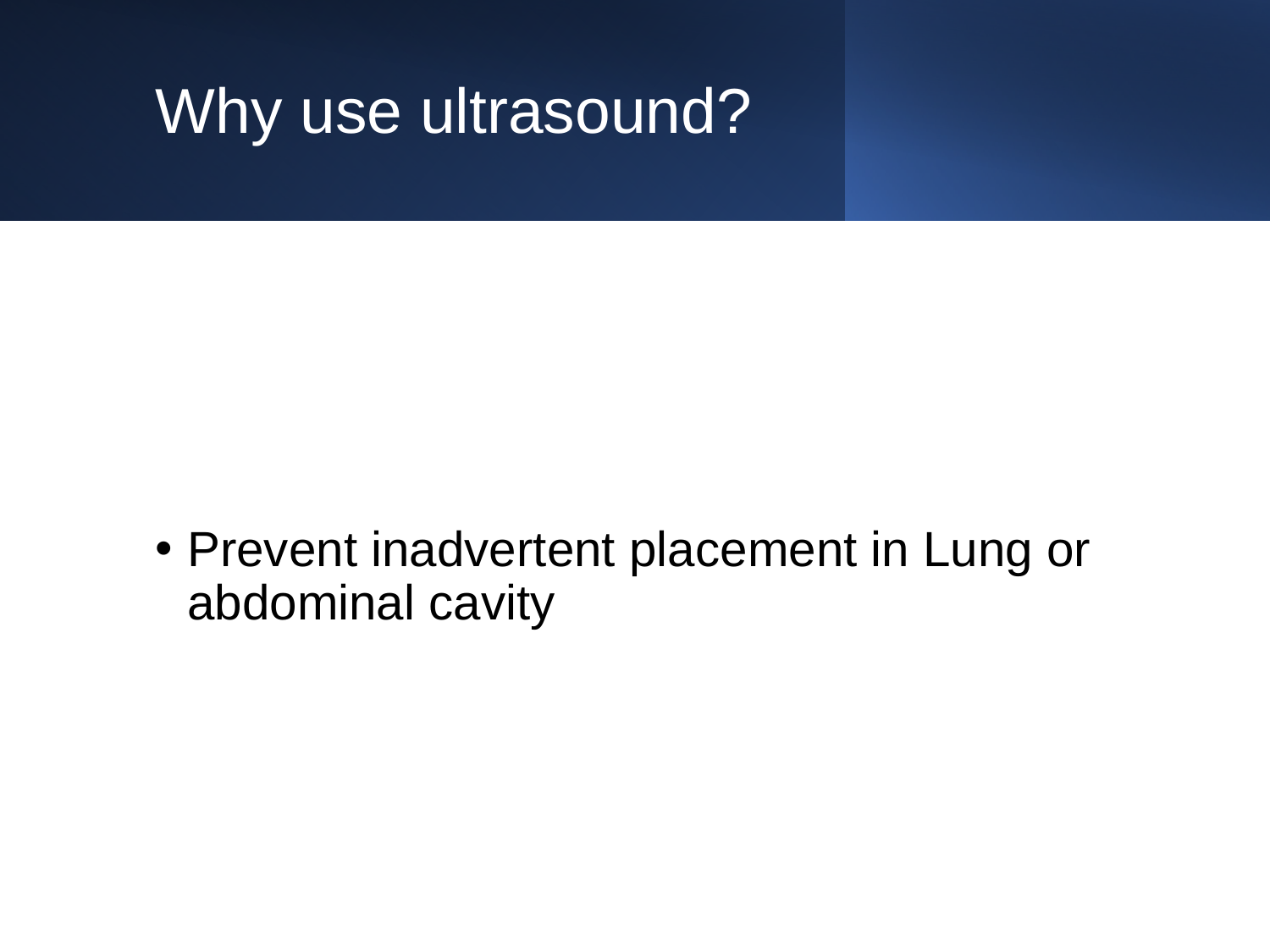

Why use ultrasound?
Prevent inadvertent placement in Lung or abdominal cavity

## Slide 15
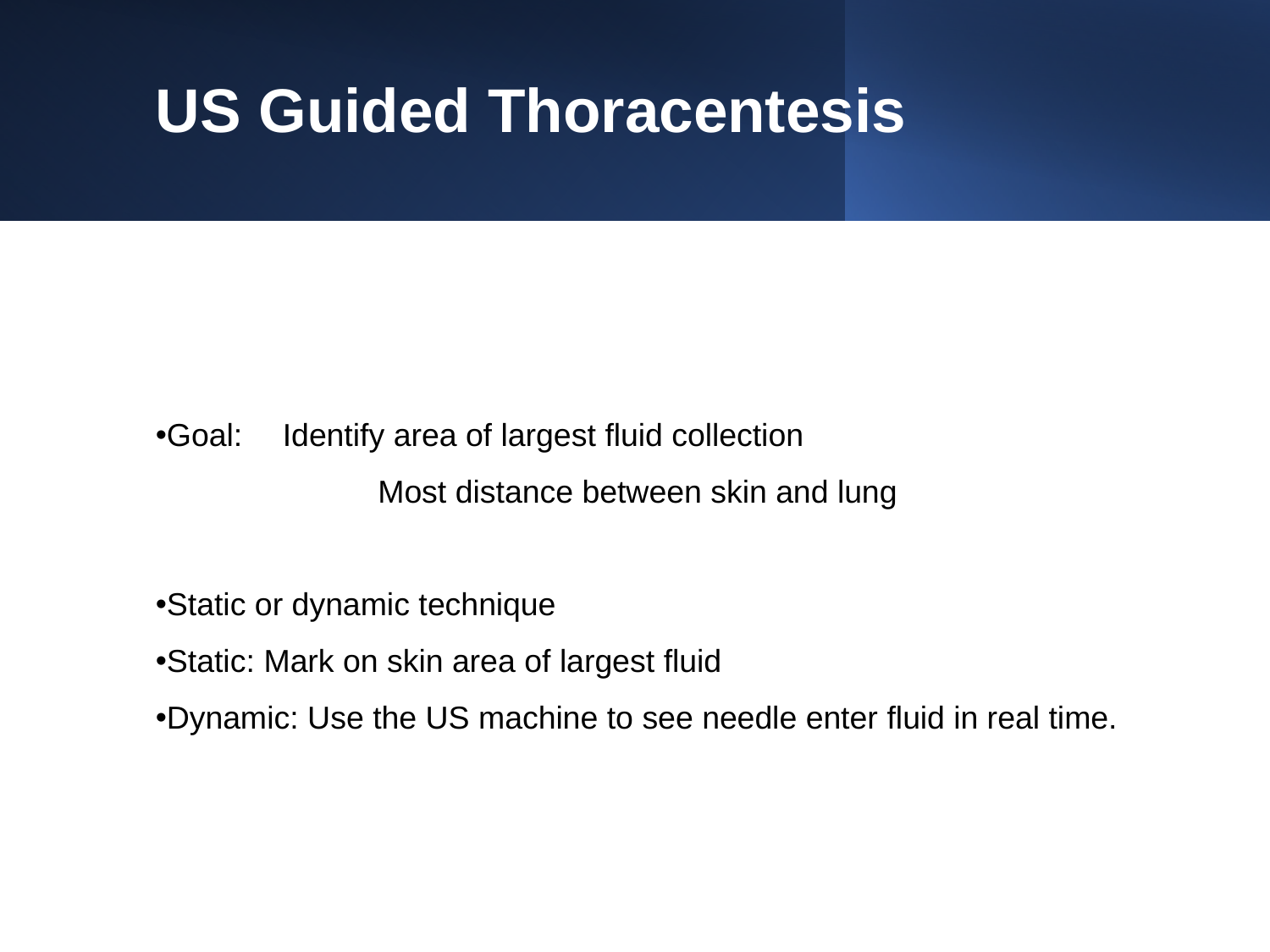

# US Guided Thoracentesis
Goal:	Identify area of largest fluid collection
	Most distance between skin and lung
Static or dynamic technique
Static: Mark on skin area of largest fluid
Dynamic: Use the US machine to see needle enter fluid in real time.

## Slide 16
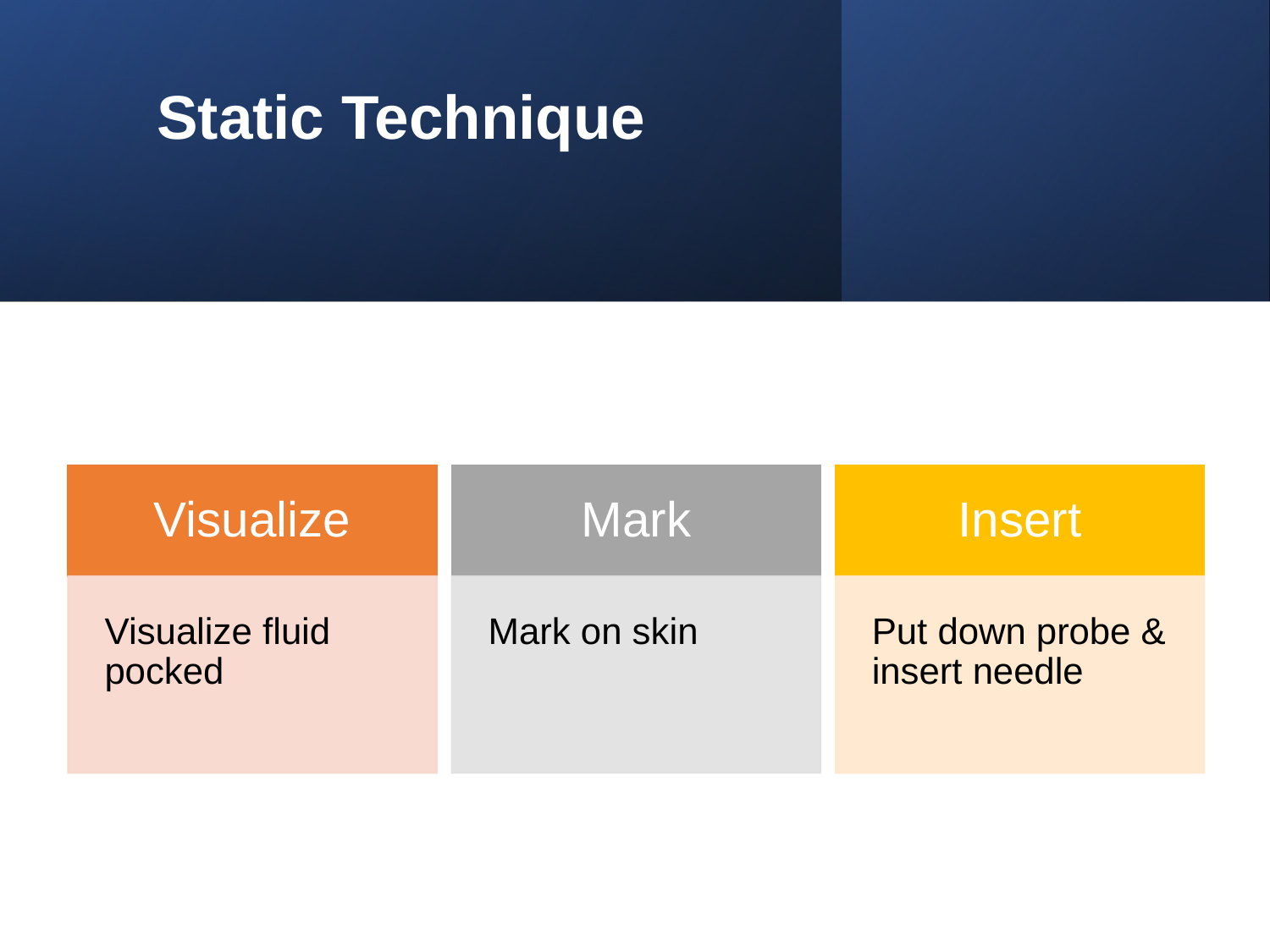

Static Technique

## Slide 17
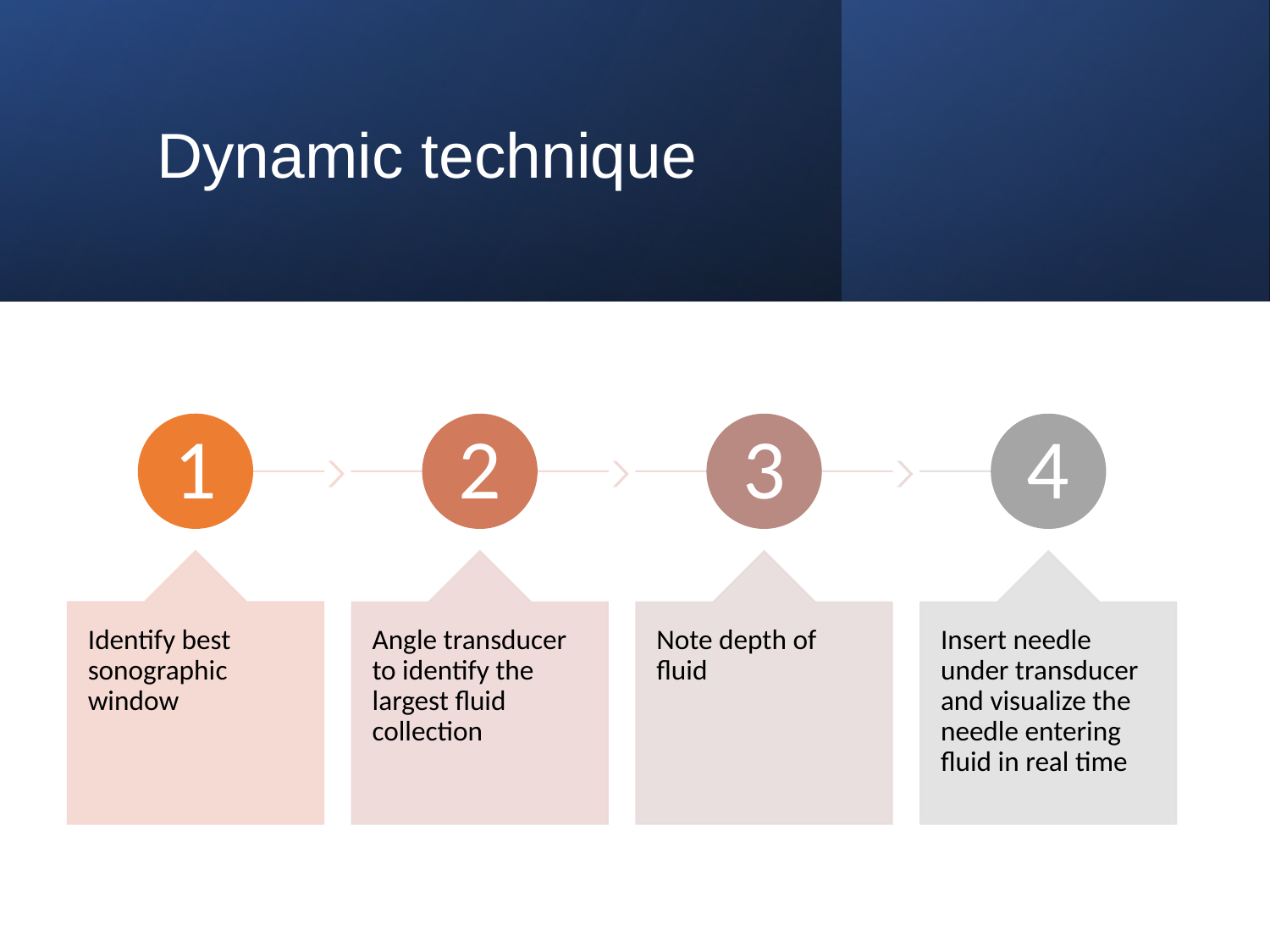

# Dynamic technique

## Slide 18
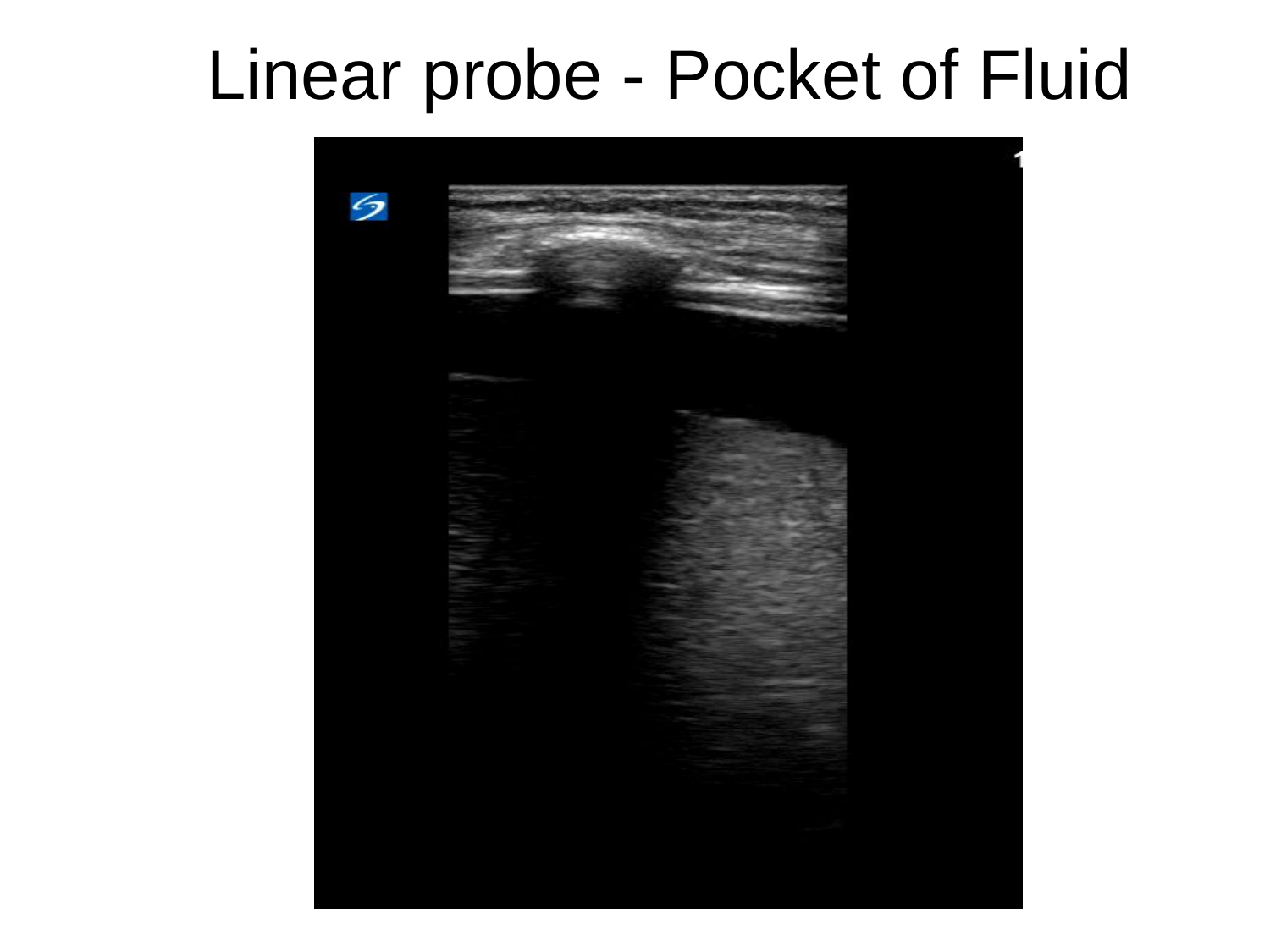

# Linear probe - Pocket of Fluid

## Slide 19
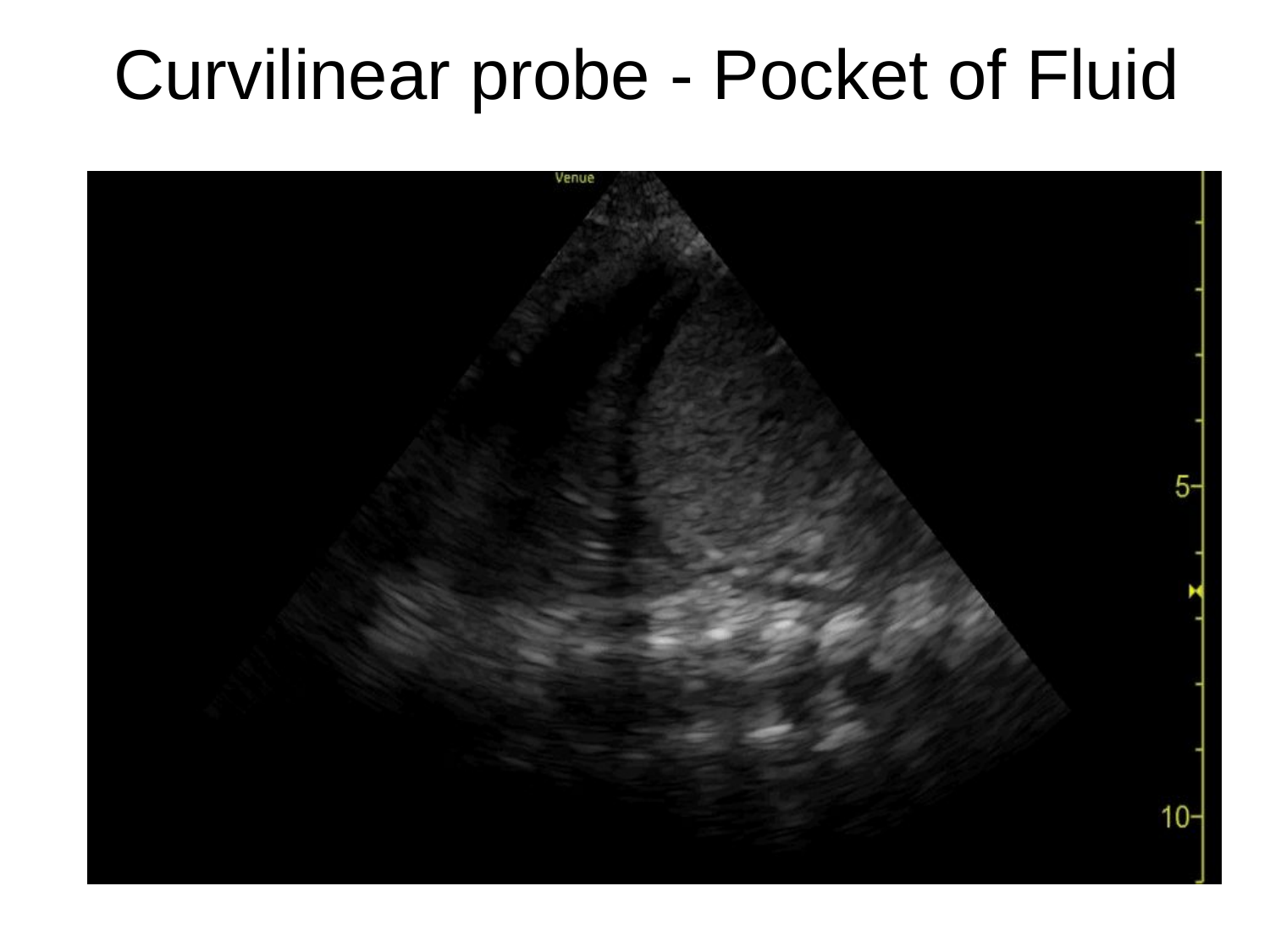

# Curvilinear probe - Pocket of Fluid

## Slide 20
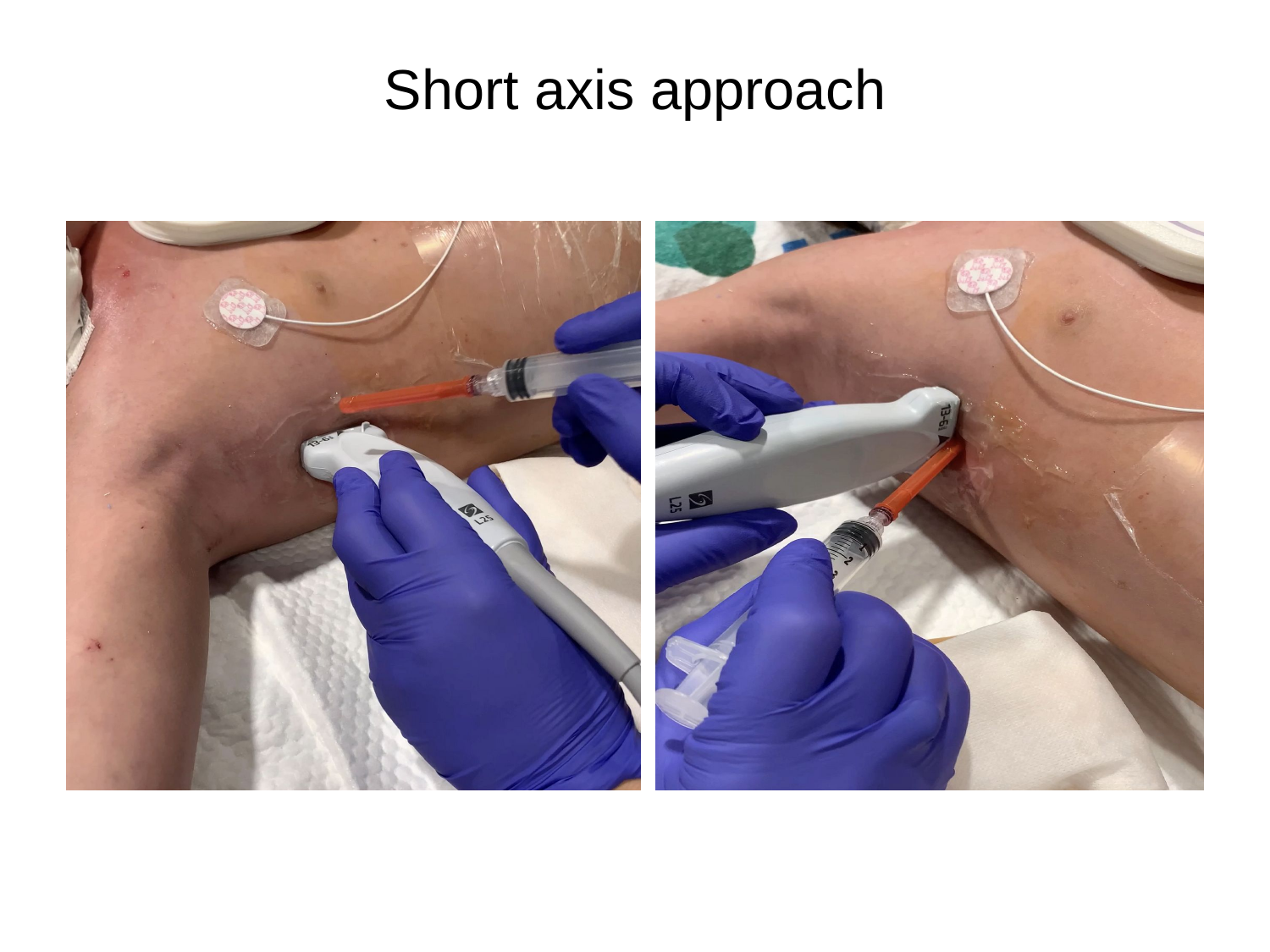

# Short axis approach

## Slide 21
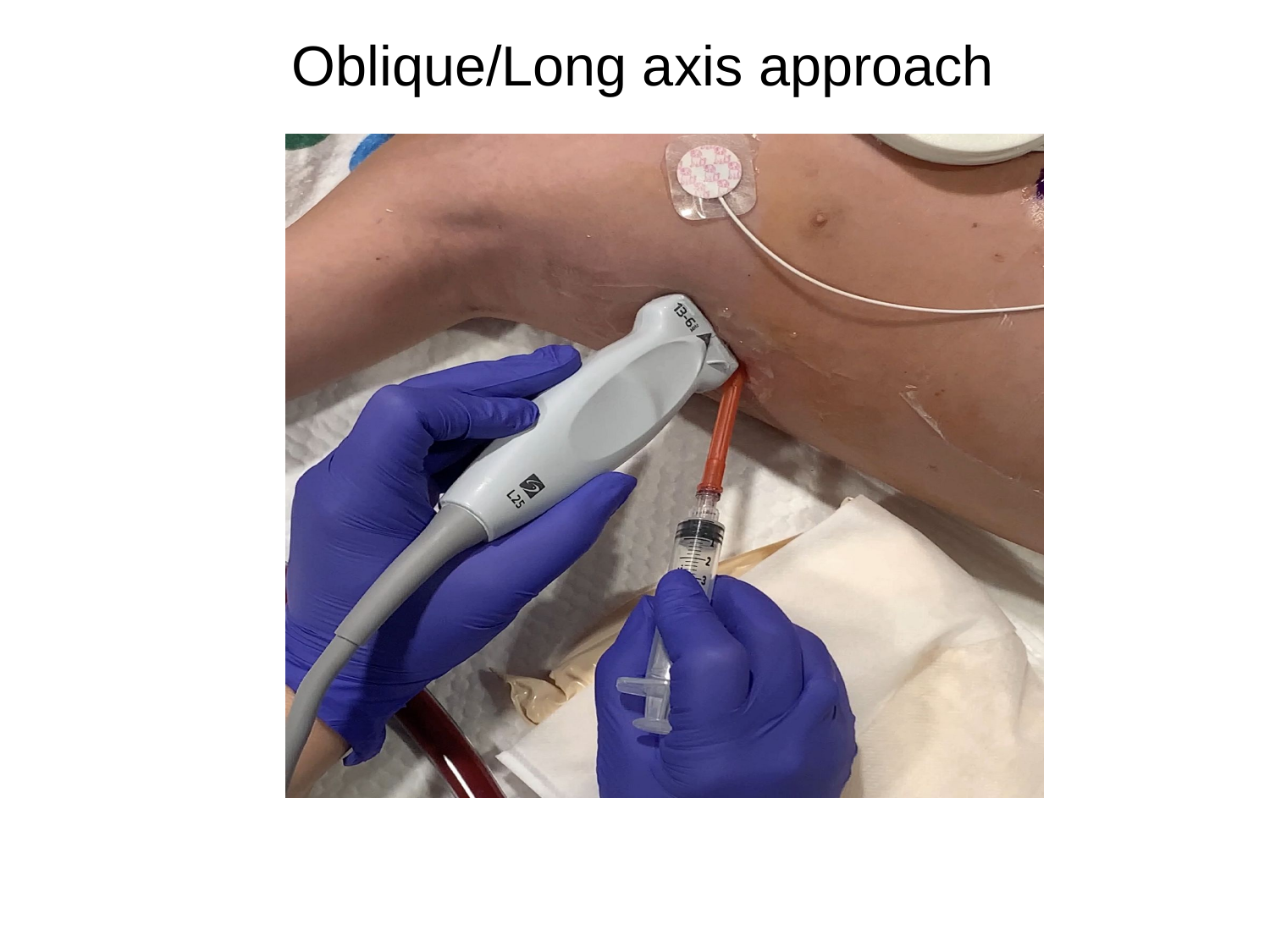

# Oblique/Long axis approach

## Slide 22
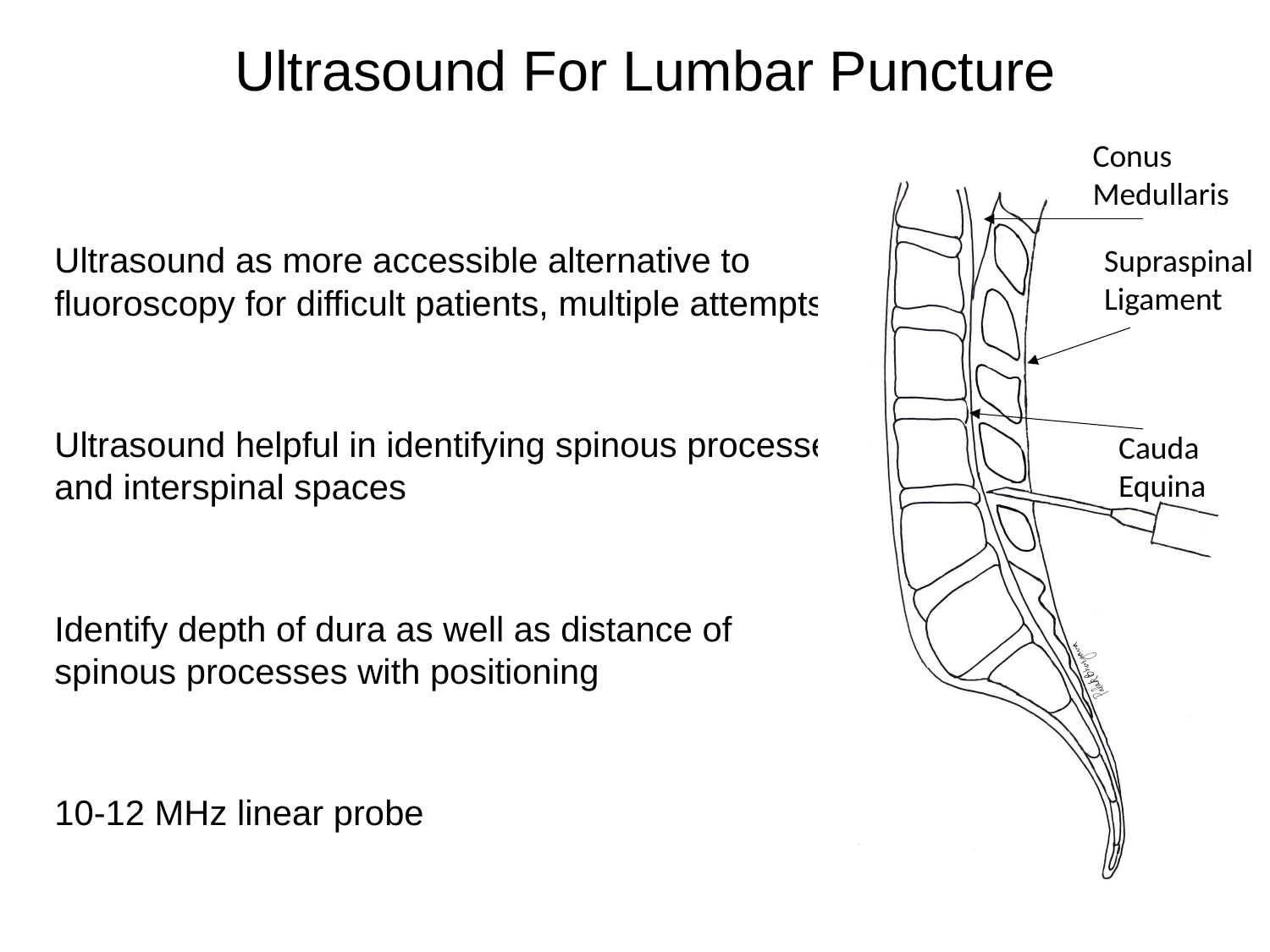

# Ultrasound For Lumbar Puncture
Conus Medullaris
Ultrasound as more accessible alternative to fluoroscopy for difficult patients, multiple attempts
Ultrasound helpful in identifying spinous processes and interspinal spaces
Identify depth of dura as well as distance of spinous processes with positioning
10-12 MHz linear probe
Supraspinal Ligament
Cauda Equina

## Slide 23
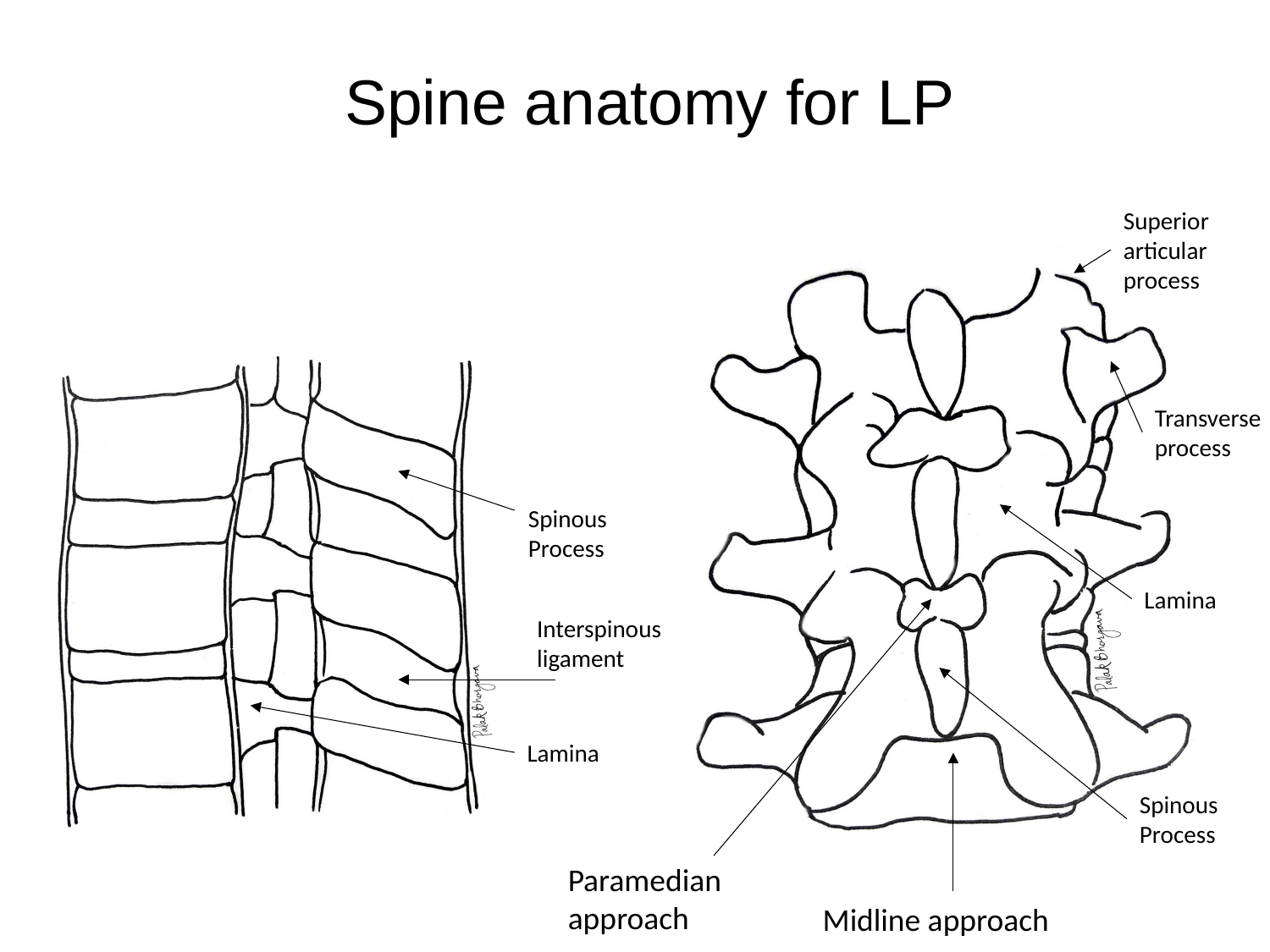

# Spine anatomy for LP
Superior articular process
Transverse process
Spinous Process
Lamina
Interspinous ligament
Lamina
Spinous Process
Paramedian approach
Midline approach

## Slide 24
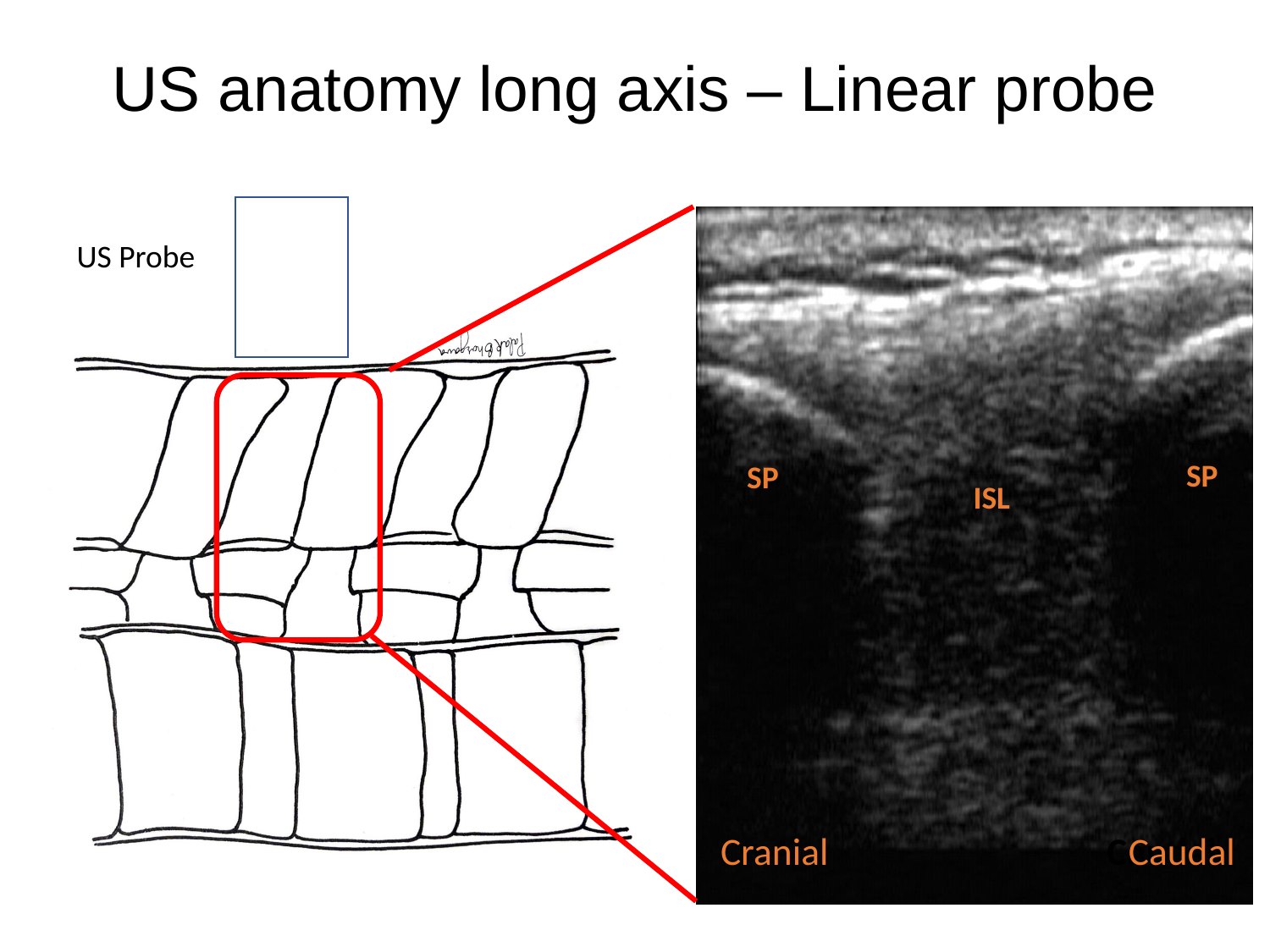

# US anatomy long axis – Linear probe
US Probe
SP
SP
ISL
Cranial
Caudal
Caudal

## Slide 25
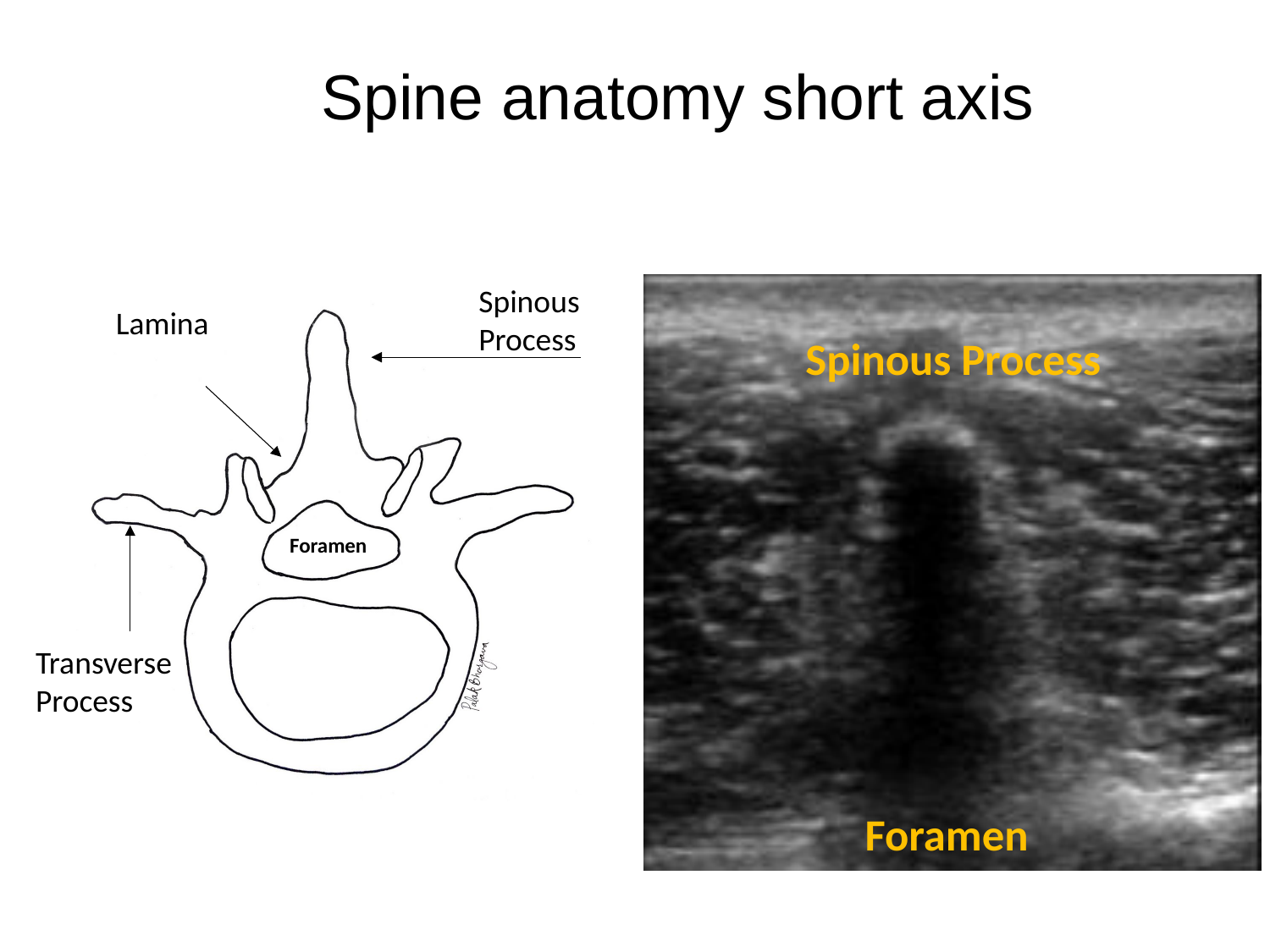

# Spine anatomy short axis
Spinous Process
Lamina
Spinous Process
Foramen
Transverse Process
Foramen

## Slide 26
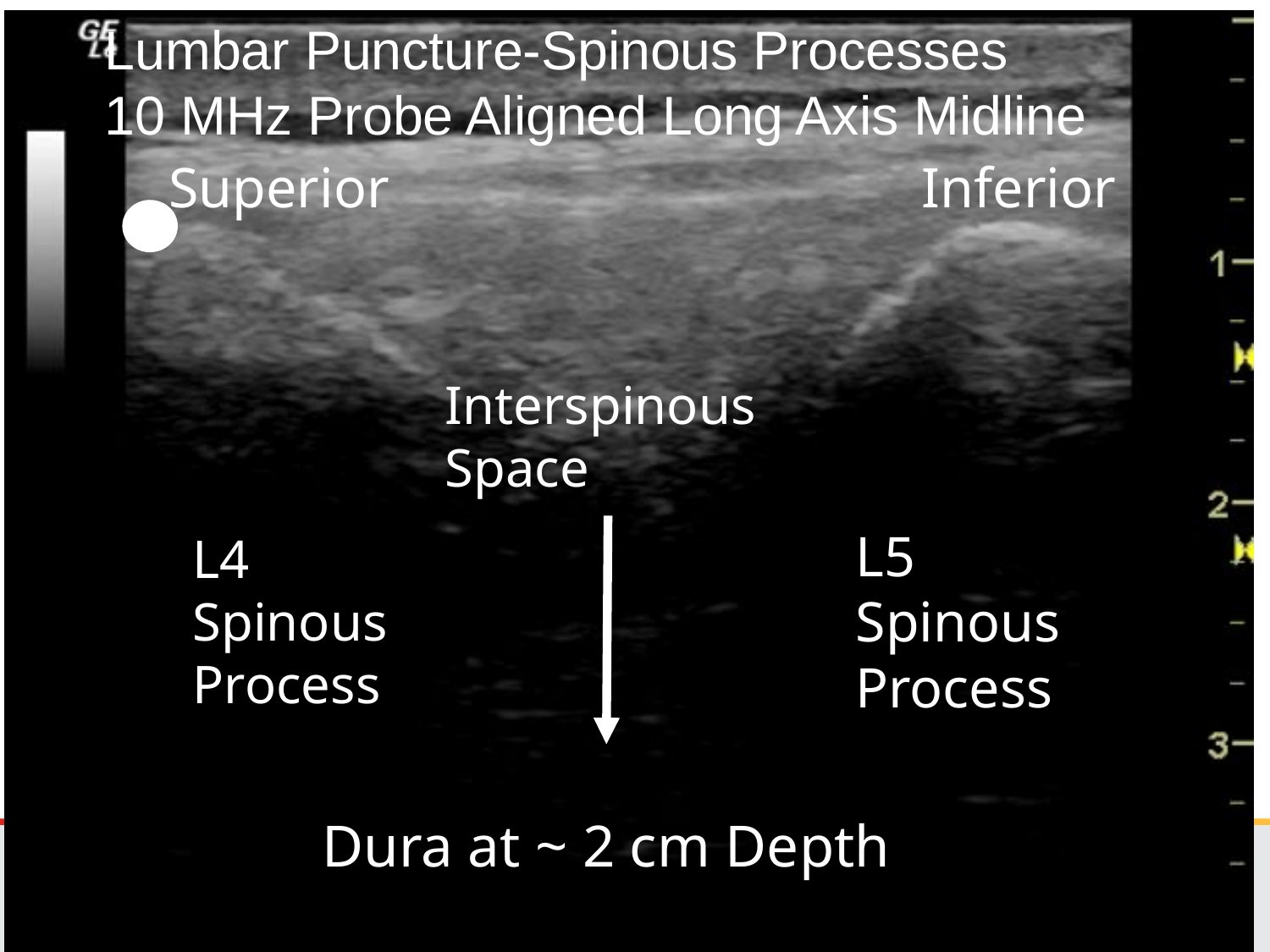

Lumbar Puncture-Spinous Processes
10 MHz Probe Aligned Long Axis Midline
Superior
Inferior
Interspinous Space
L5
Spinous
Process
L4
Spinous
Process
Dura at ~ 2 cm Depth

## Slide 27
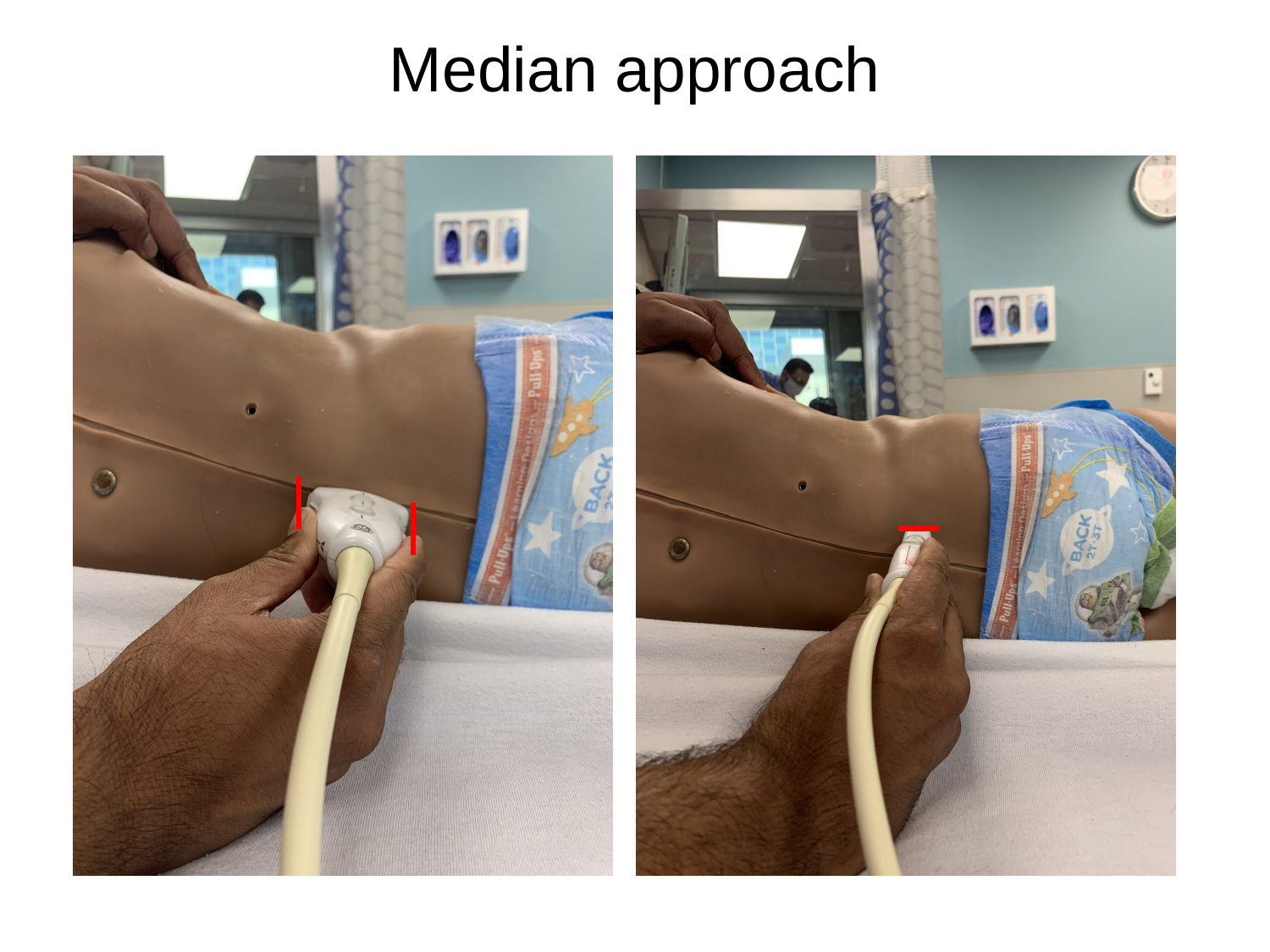

# Median approach

## Slide 28
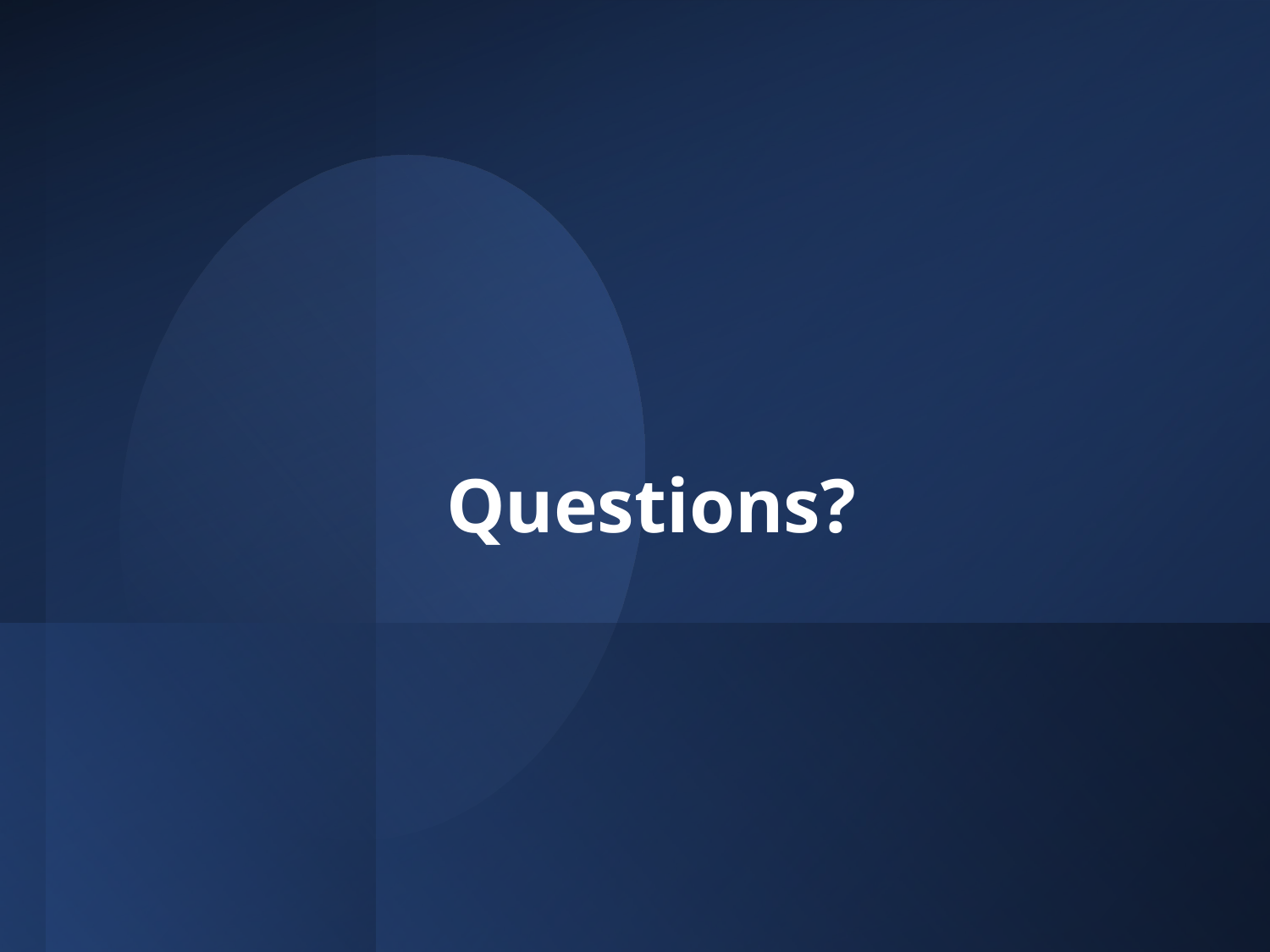

Questions?
