# Supplementary material for: A point-of-care ultrasound education curriculum for pediatric critical care medicine
Source: Ultrasound J. 2022 Oct 31;14:44. doi: 10.1186/s13089-022-00290-6 (PMC9622960; doi:10.1186/s13089-022-00290-6)
Supplement: Supplementary file 6 — Additional file 6. Focused echocardiographic examination [file 13089_2022_290_MOESM6_ESM.pptx]

## Slide 1
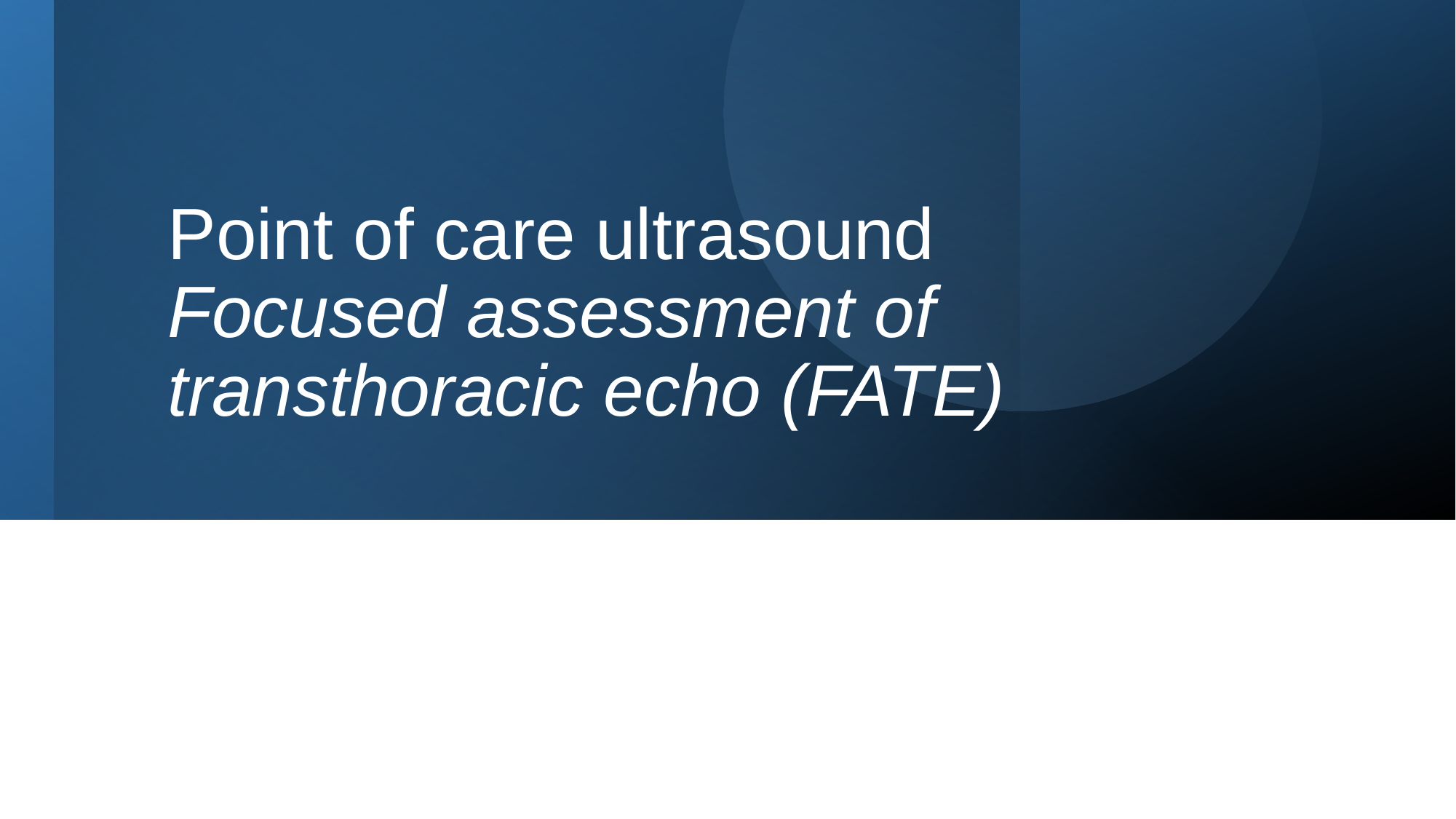

# Point of care ultrasound Focused assessment of transthoracic echo (FATE)

## Slide 2
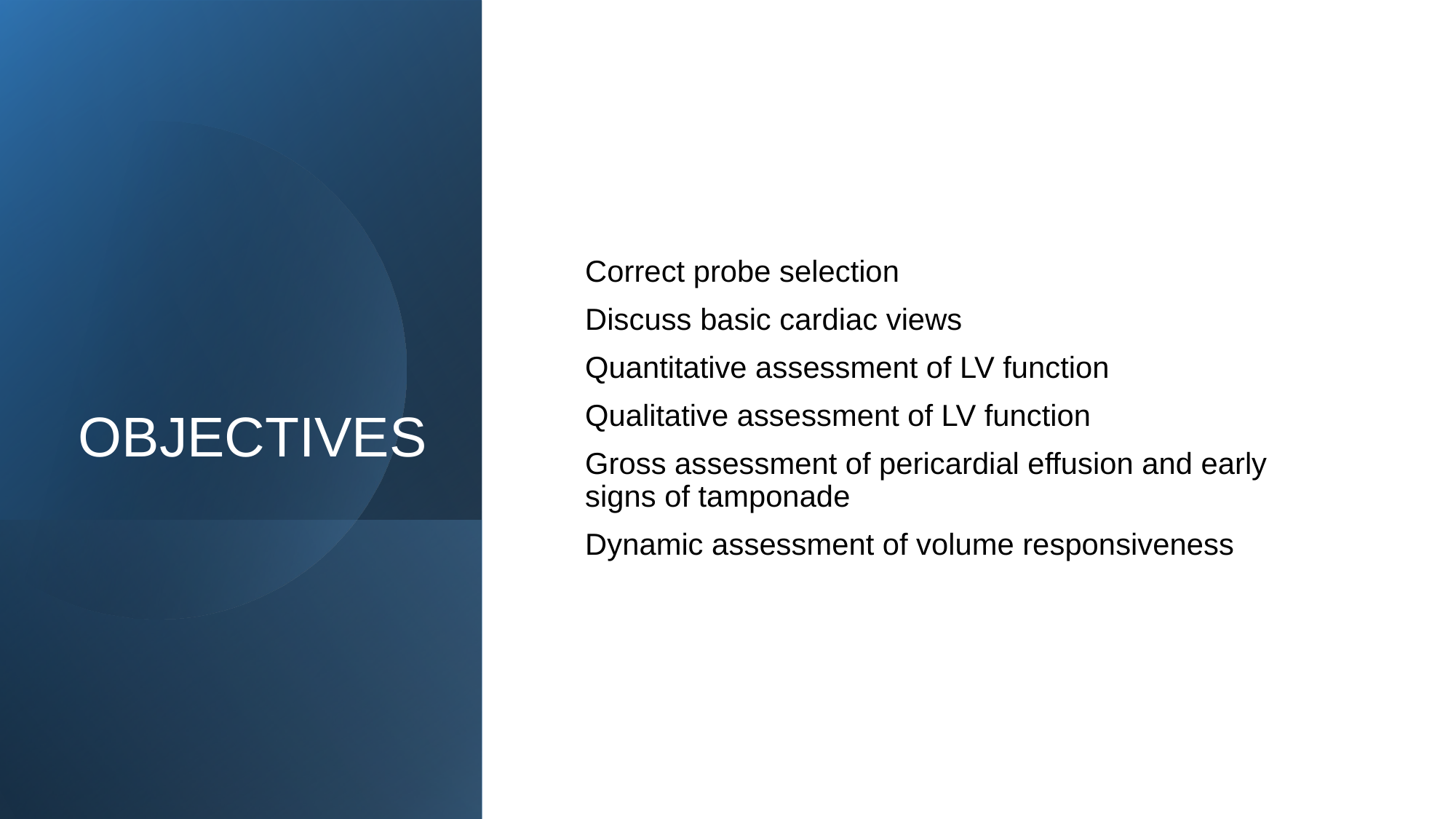

# OBJECTIVES
Correct probe selection
Discuss basic cardiac views
Quantitative assessment of LV function
Qualitative assessment of LV function
Gross assessment of pericardial effusion and early signs of tamponade
Dynamic assessment of volume responsiveness

## Slide 3
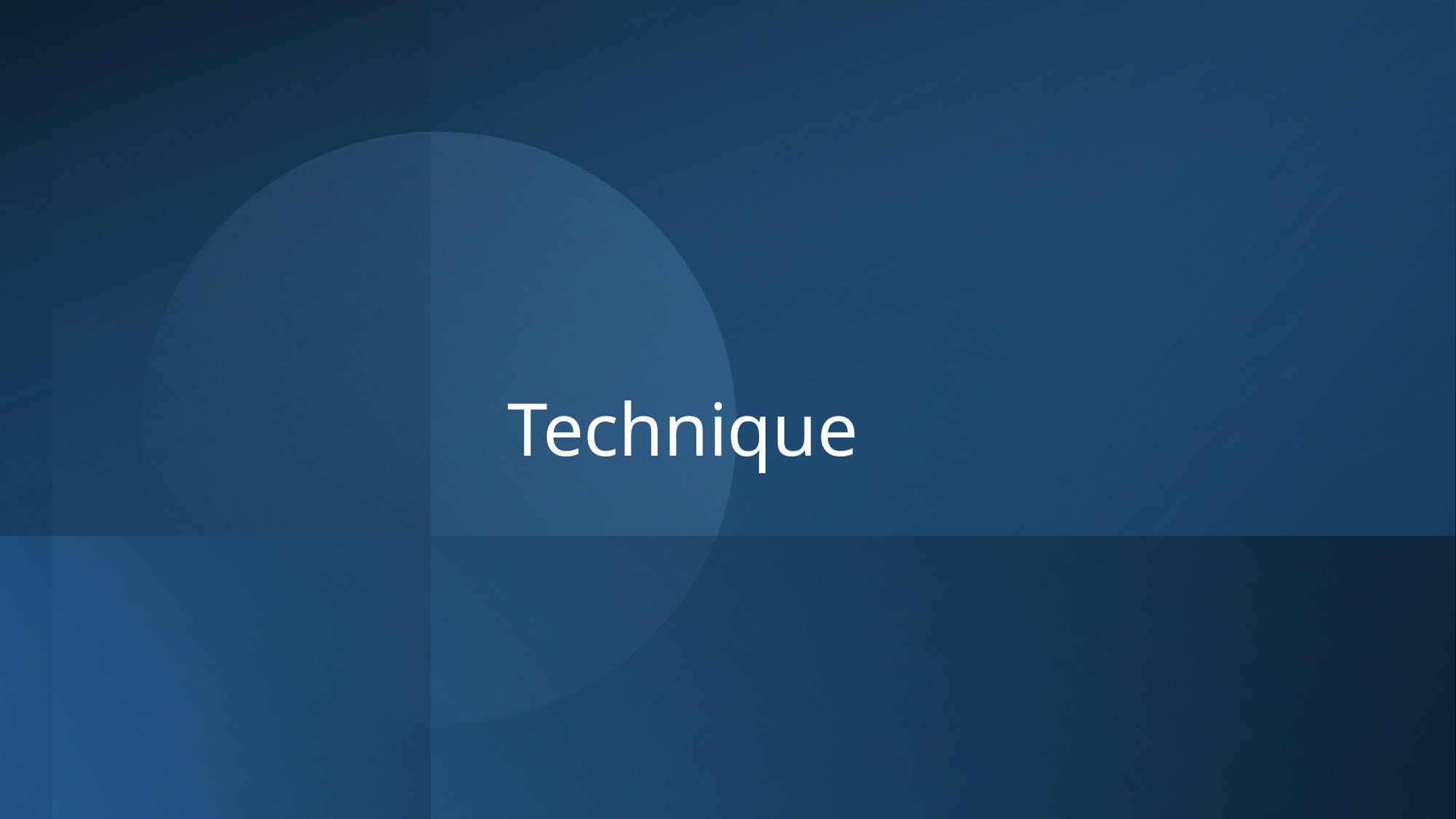

# Technique

## Slide 4
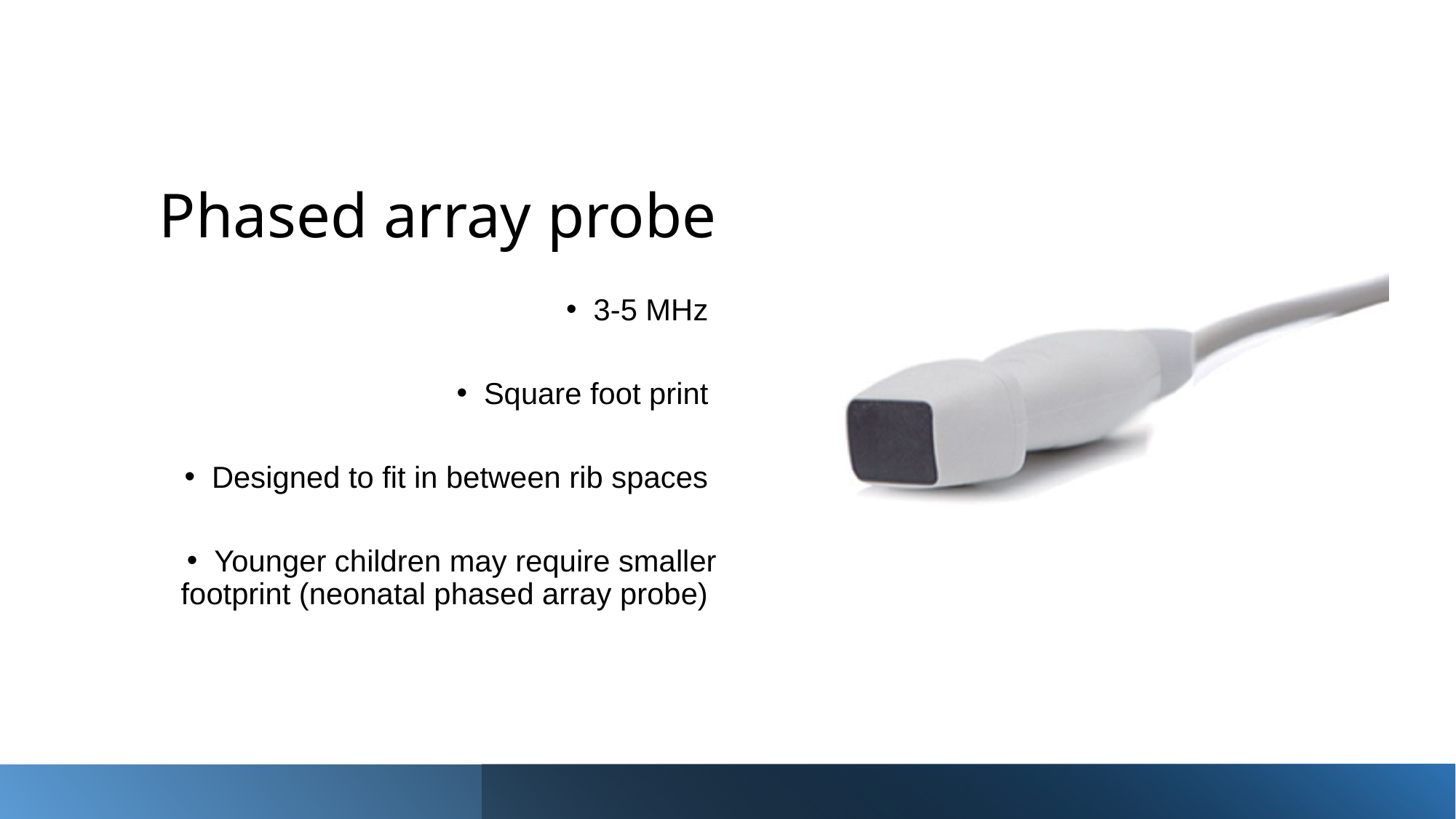

# Phased array probe
3-5 MHz
Square foot print
Designed to fit in between rib spaces
Younger children may require smaller footprint (neonatal phased array probe)

## Slide 5
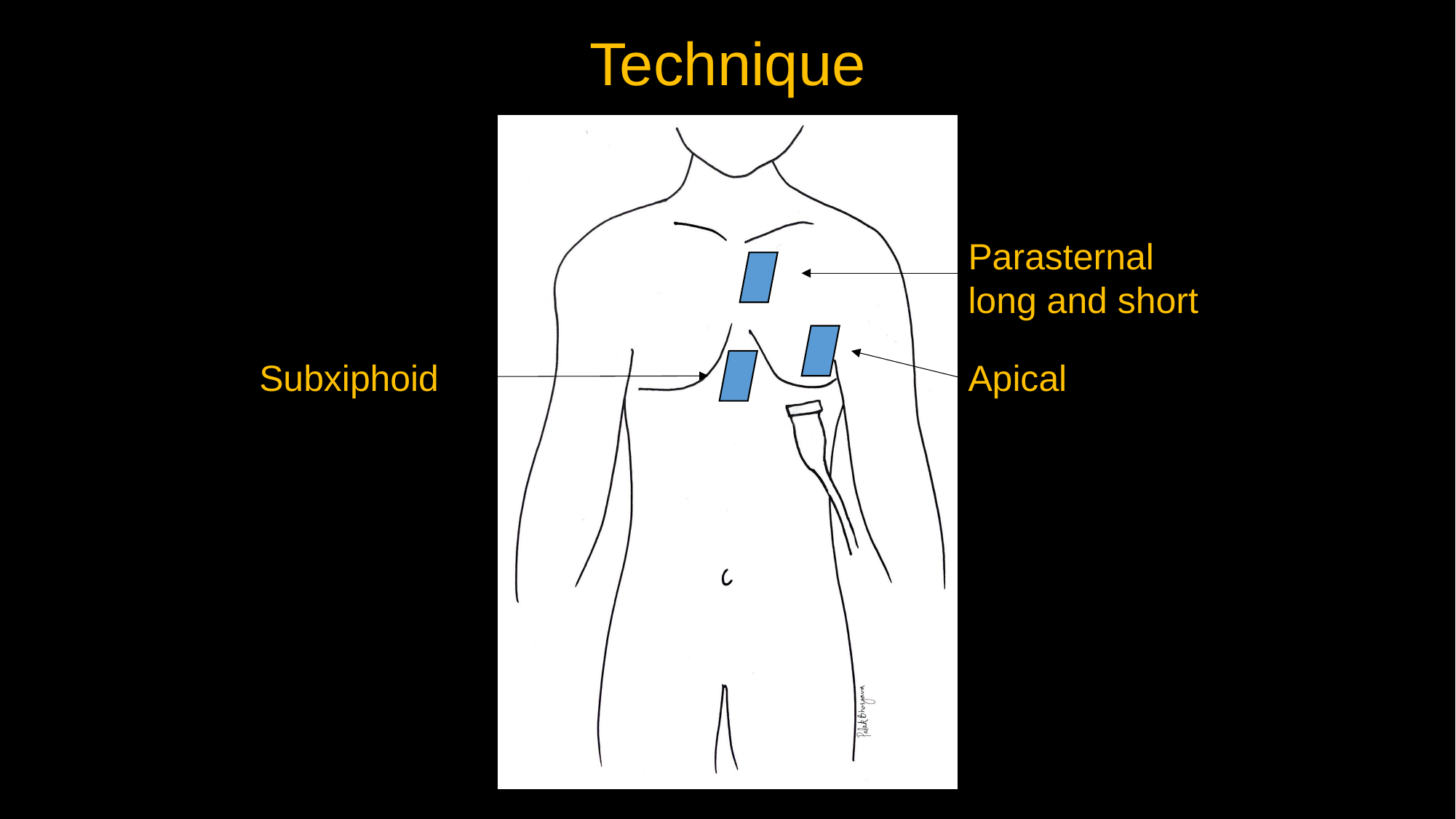

# Technique
Parasternal long and short
Subxiphoid
Apical

## Slide 6
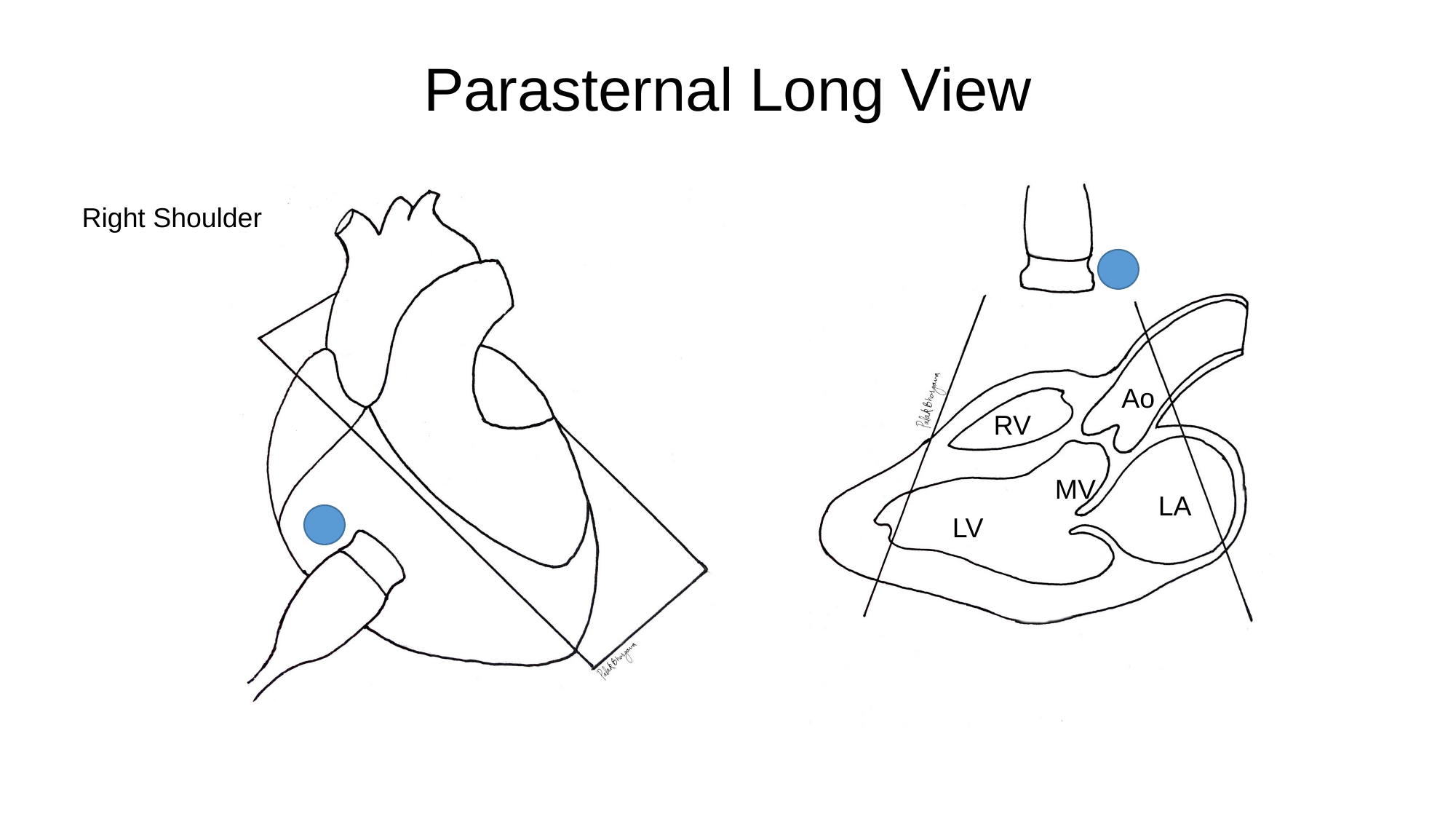

Parasternal Long View
Right Shoulder
Ao
RV
MV
LA
LV

## Slide 7
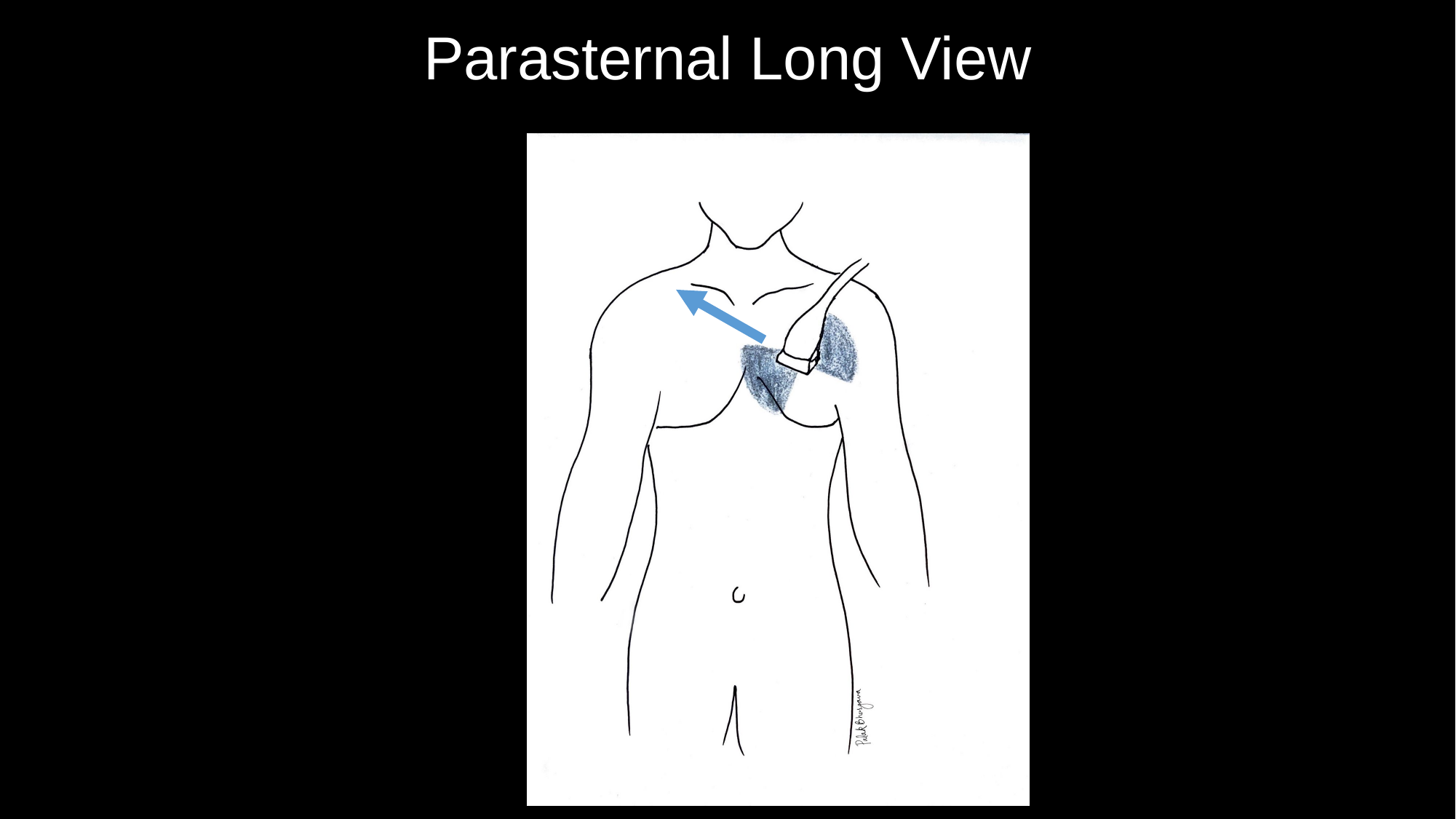

# Parasternal Long View

## Slide 8
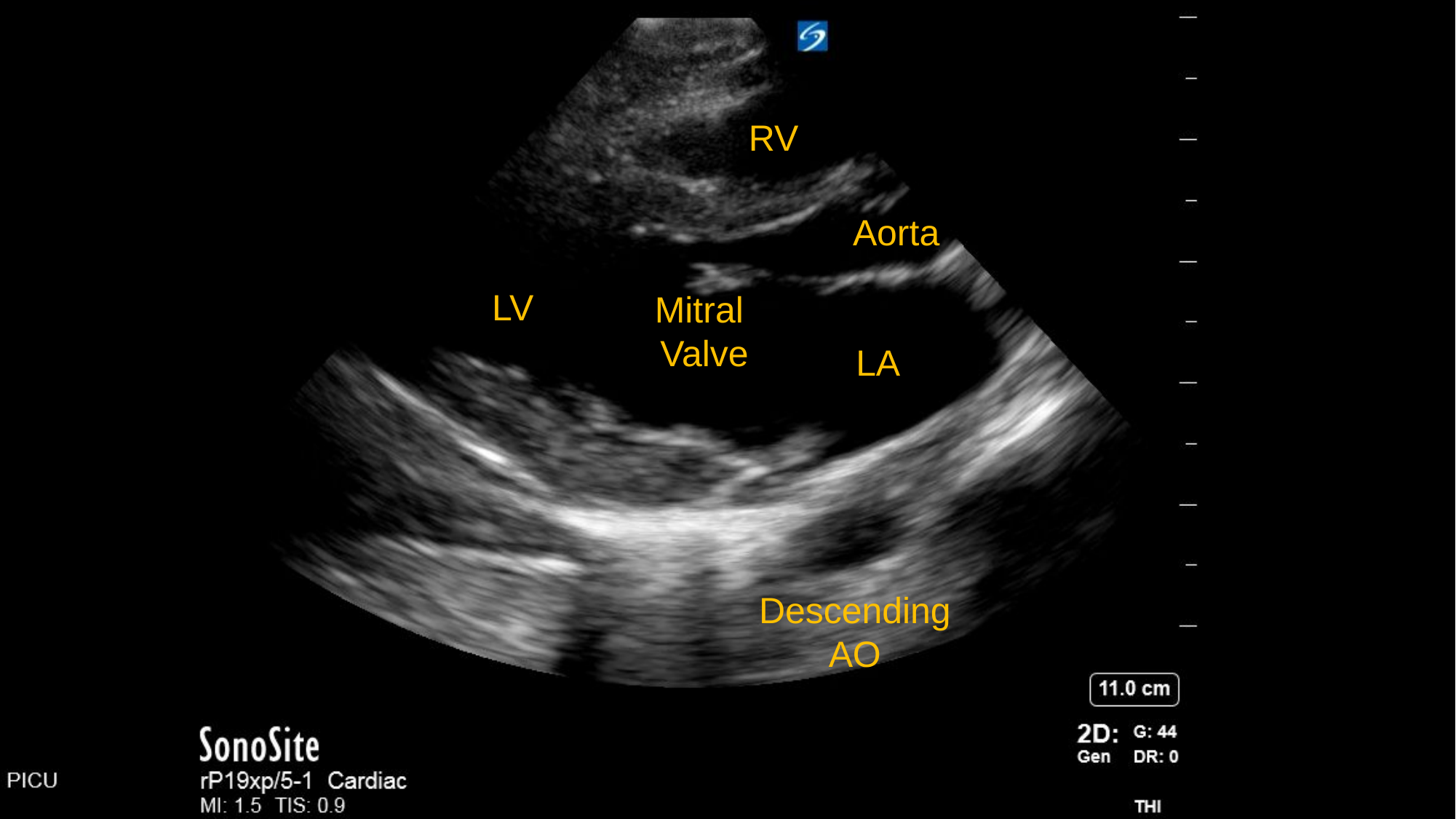

# Parasternal Long View
RV
Aorta
LV
Mitral
Valve
LA
Descending AO

## Slide 9
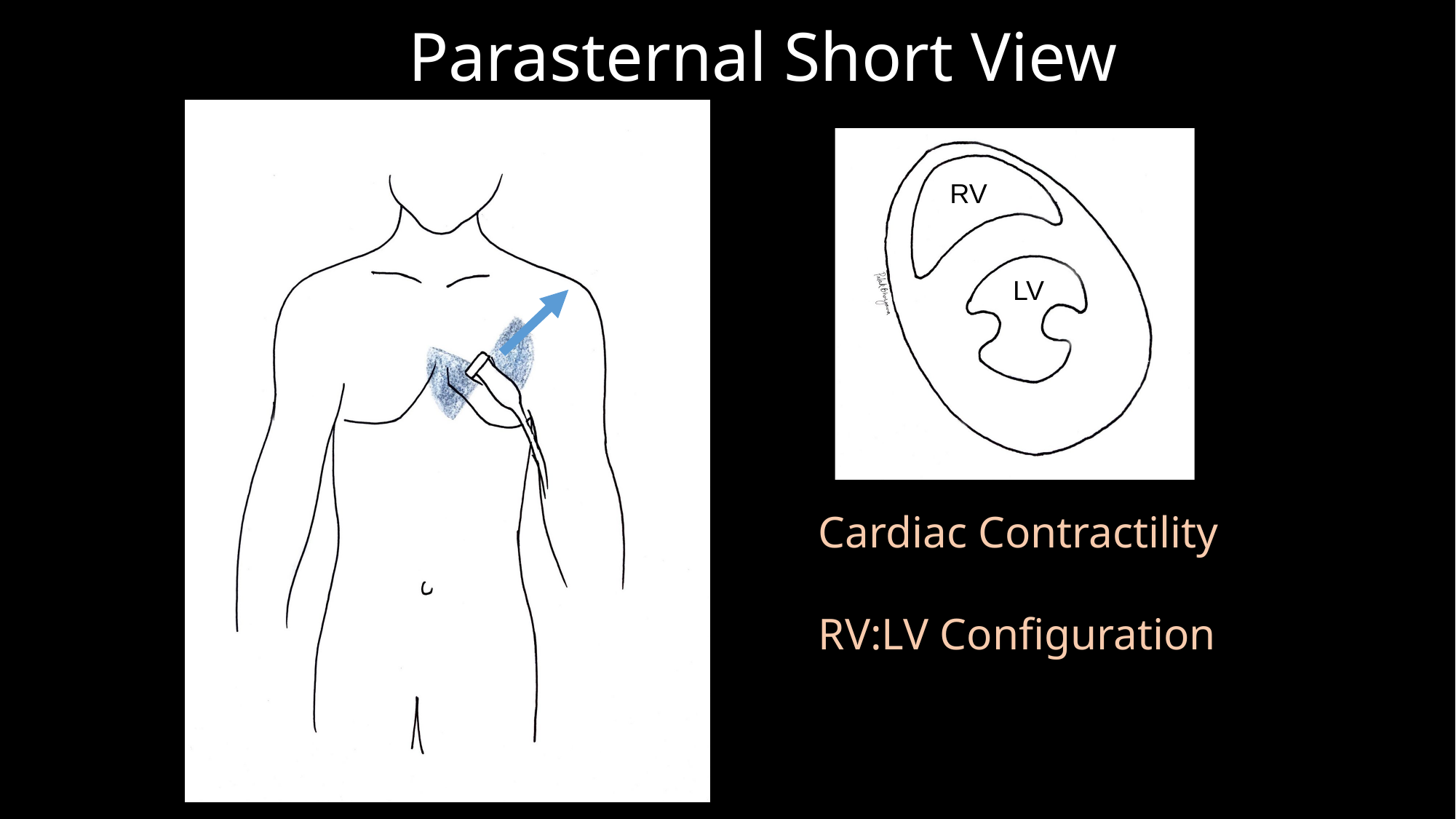

# Parasternal Short View
RV
LV
Marker
R. Hip
Cardiac Contractility
RV:LV Configuration

## Slide 10
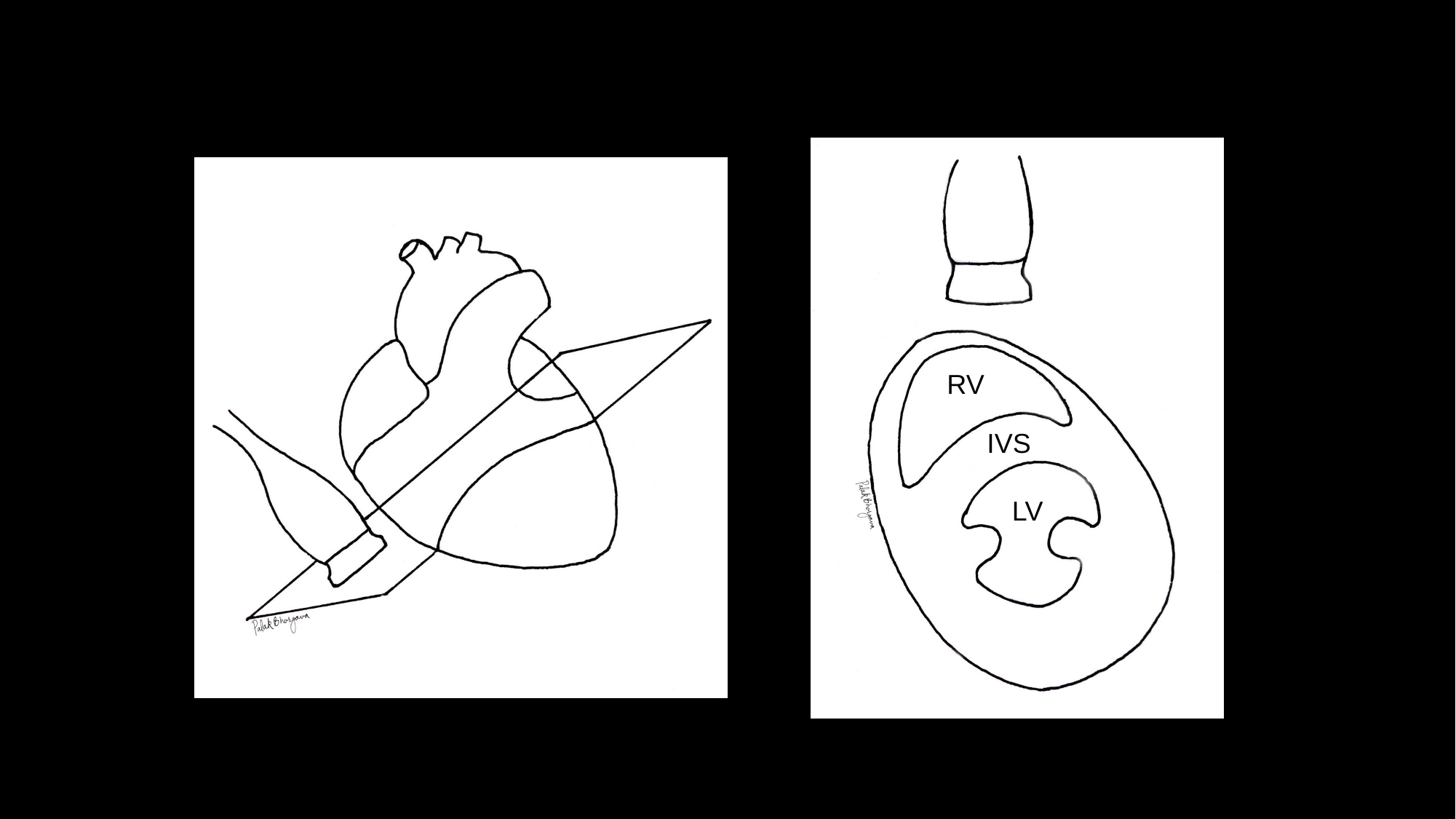

Parasternal Short Axis: Papillary Level
RV
IVS
LV

## Slide 11
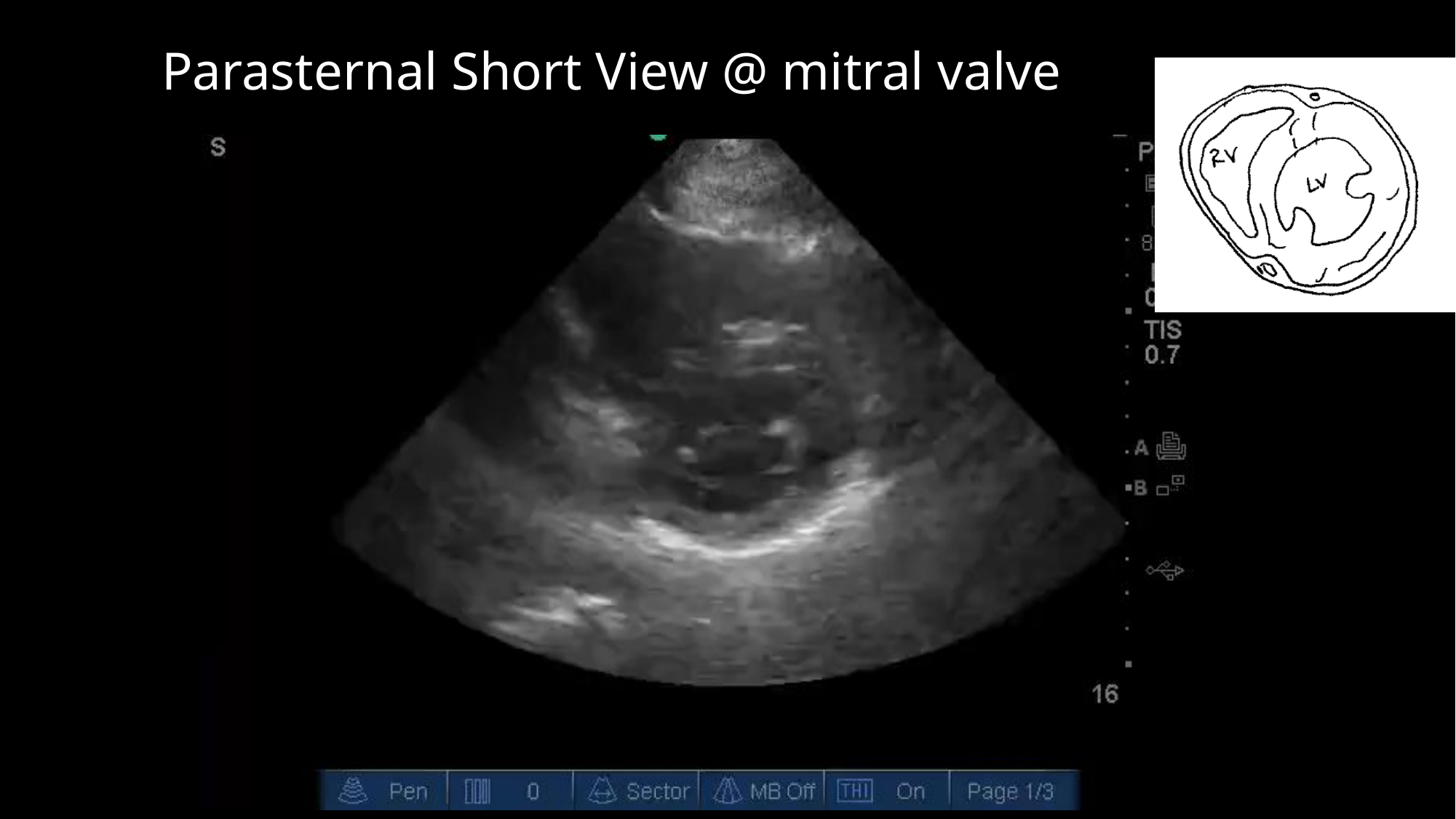

# Parasternal Short View @ mitral valve

## Slide 12
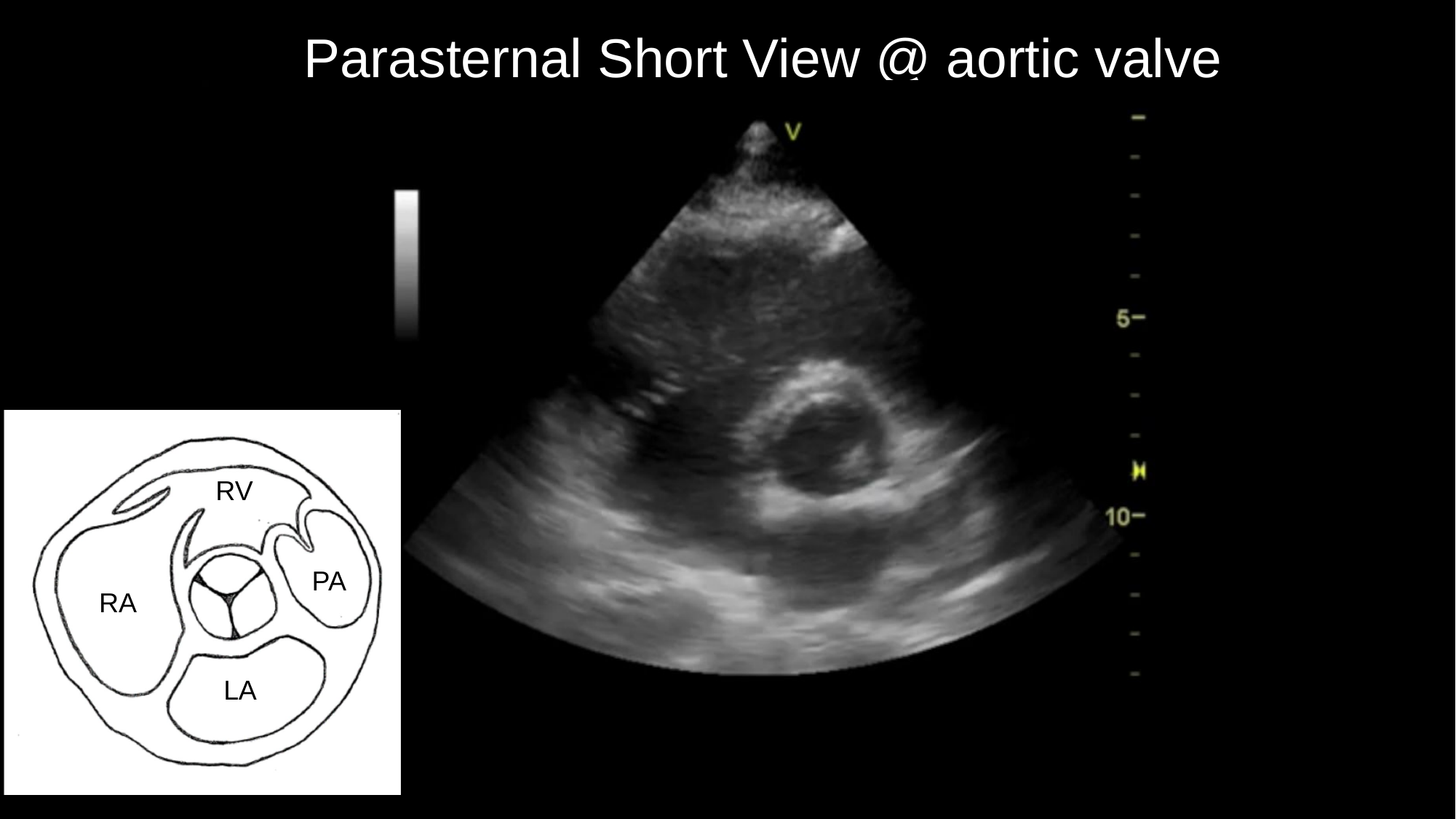

# Parasternal Short View @ aortic valve
RV
PA
RA
LA

## Slide 13
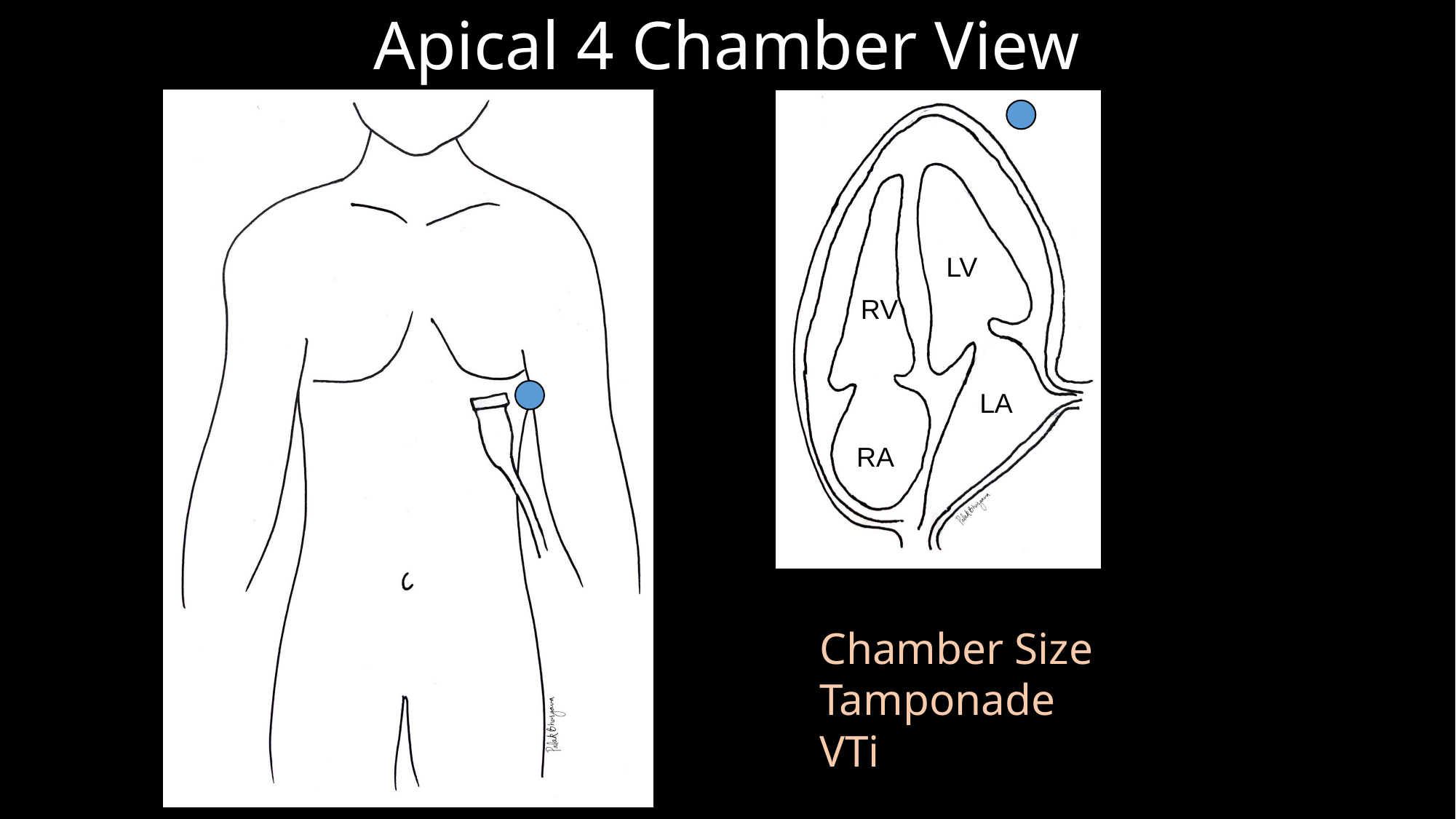

# Apical 4 Chamber View
LV
RV
LA
RA
Chamber Size
Tamponade
VTi

## Slide 14
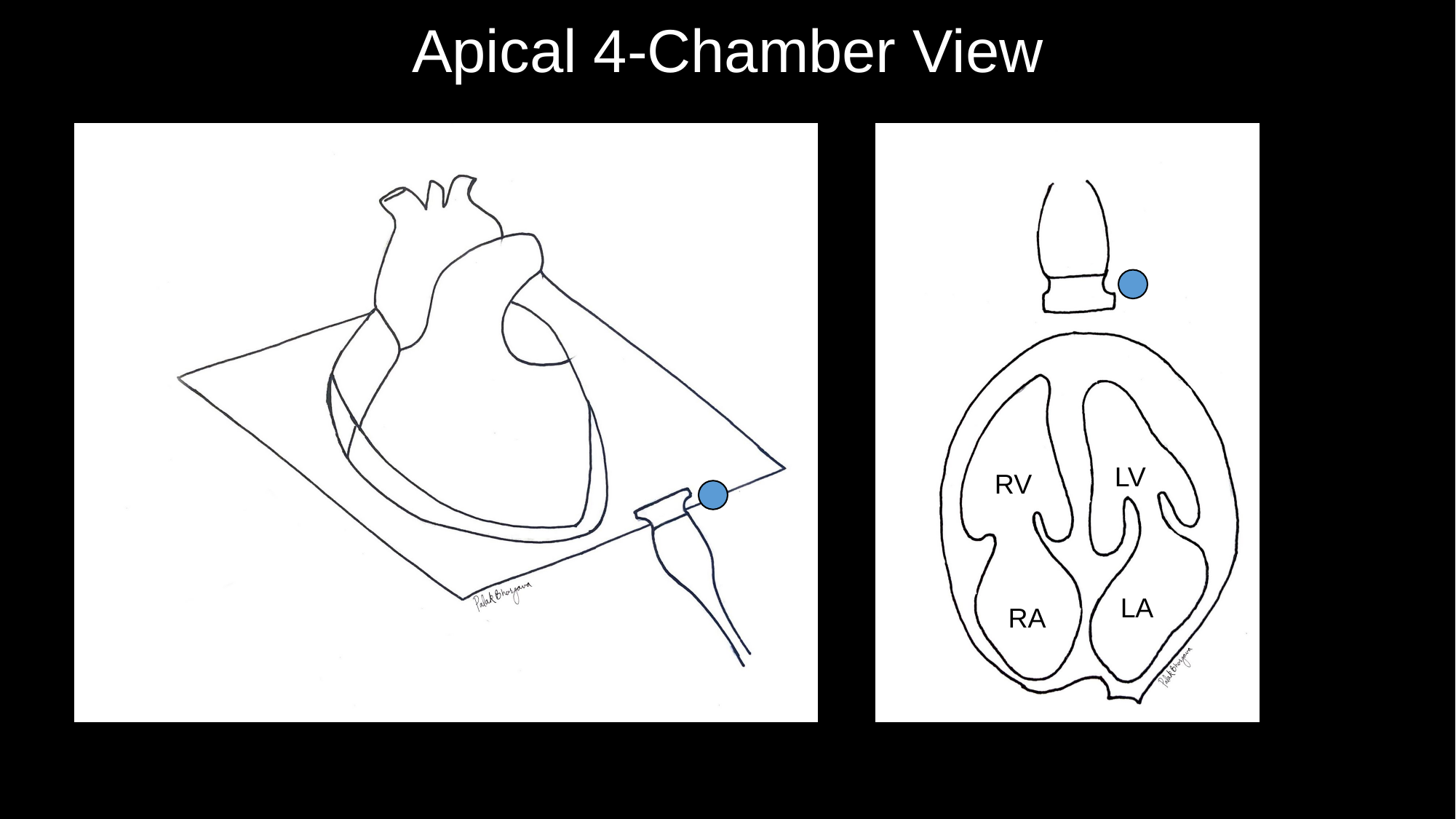

# Apical 4-Chamber View
LV
RV
LA
RA

## Slide 15
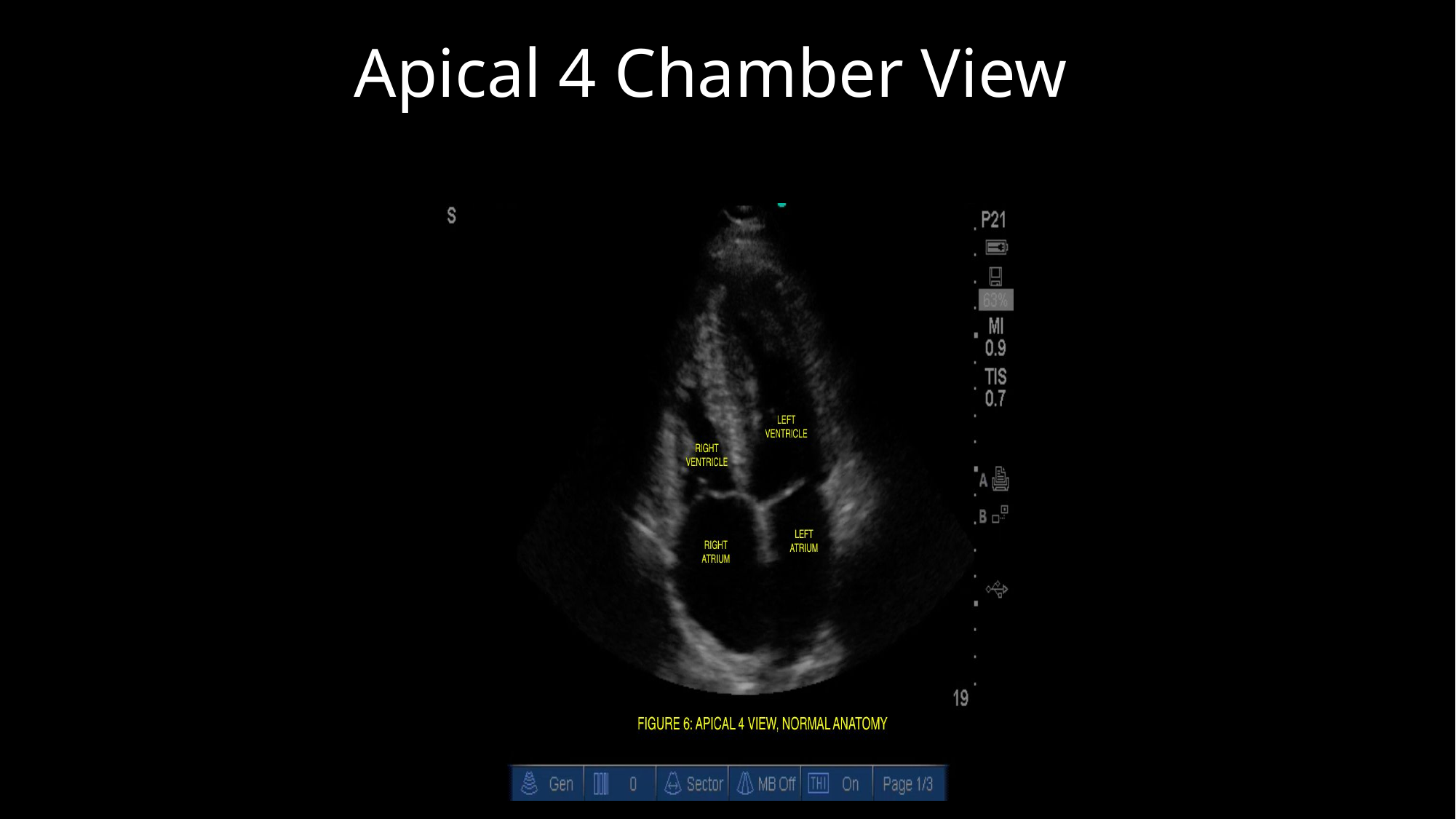

Apical 4 Chamber View

## Slide 16
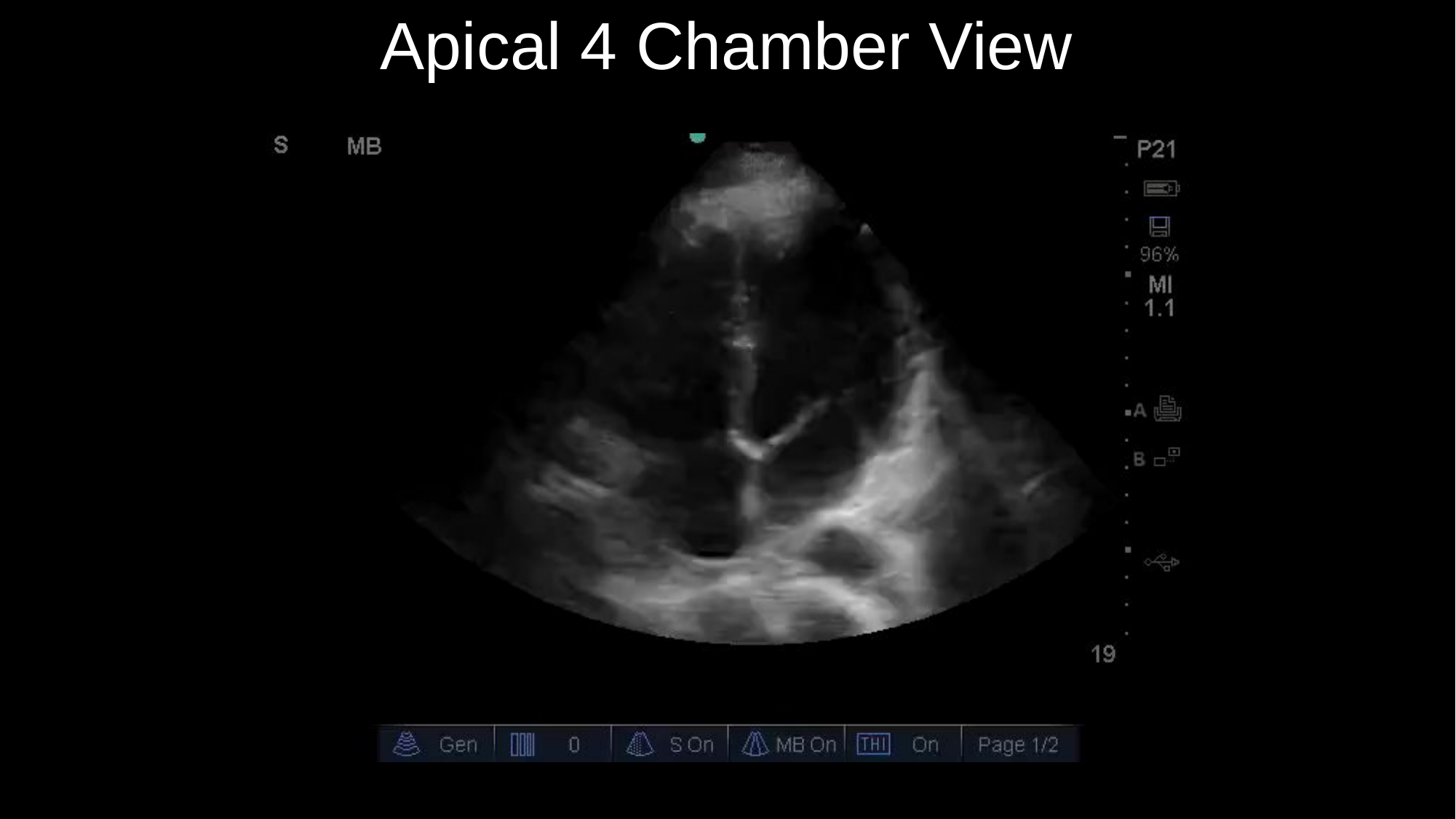

Apical 4 Chamber View

## Slide 17
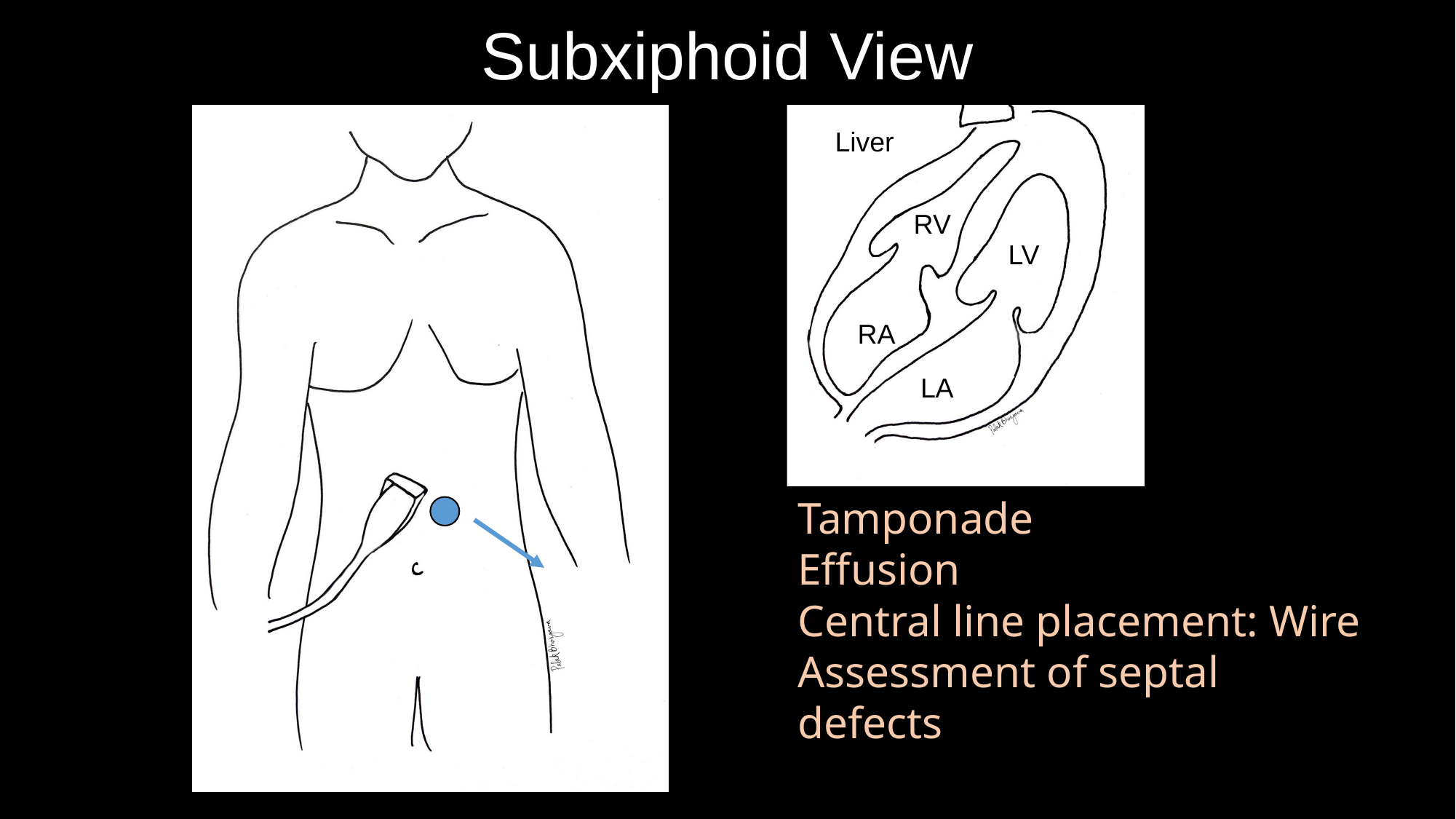

# Subxiphoid View
Liver
RV
LV
RA
LA
Tamponade
Effusion
Central line placement: Wire
Assessment of septal defects

## Slide 18
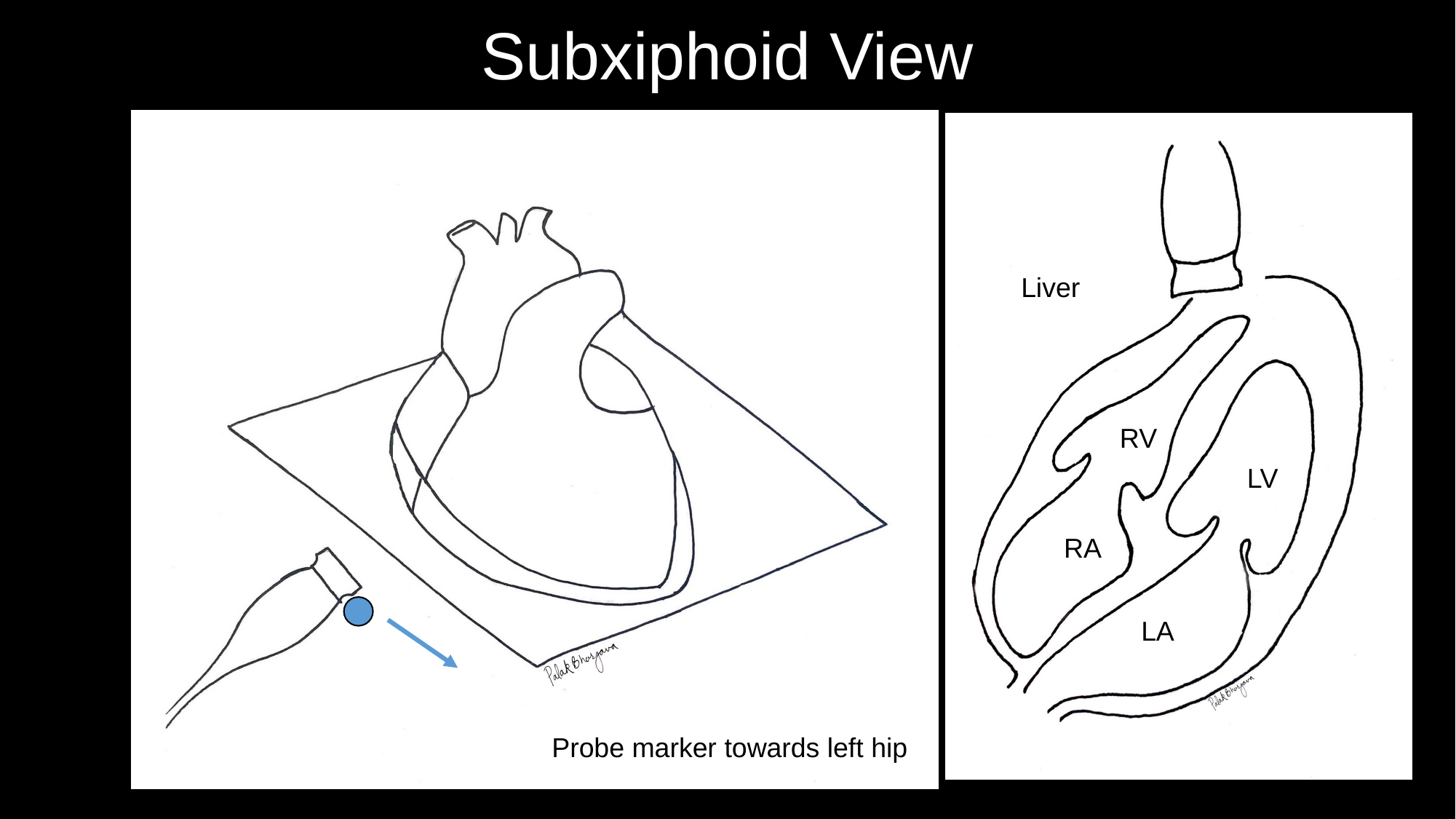

# Subxiphoid View
Liver
RV
LV
RA
LA
Probe marker towards left hip

## Slide 19
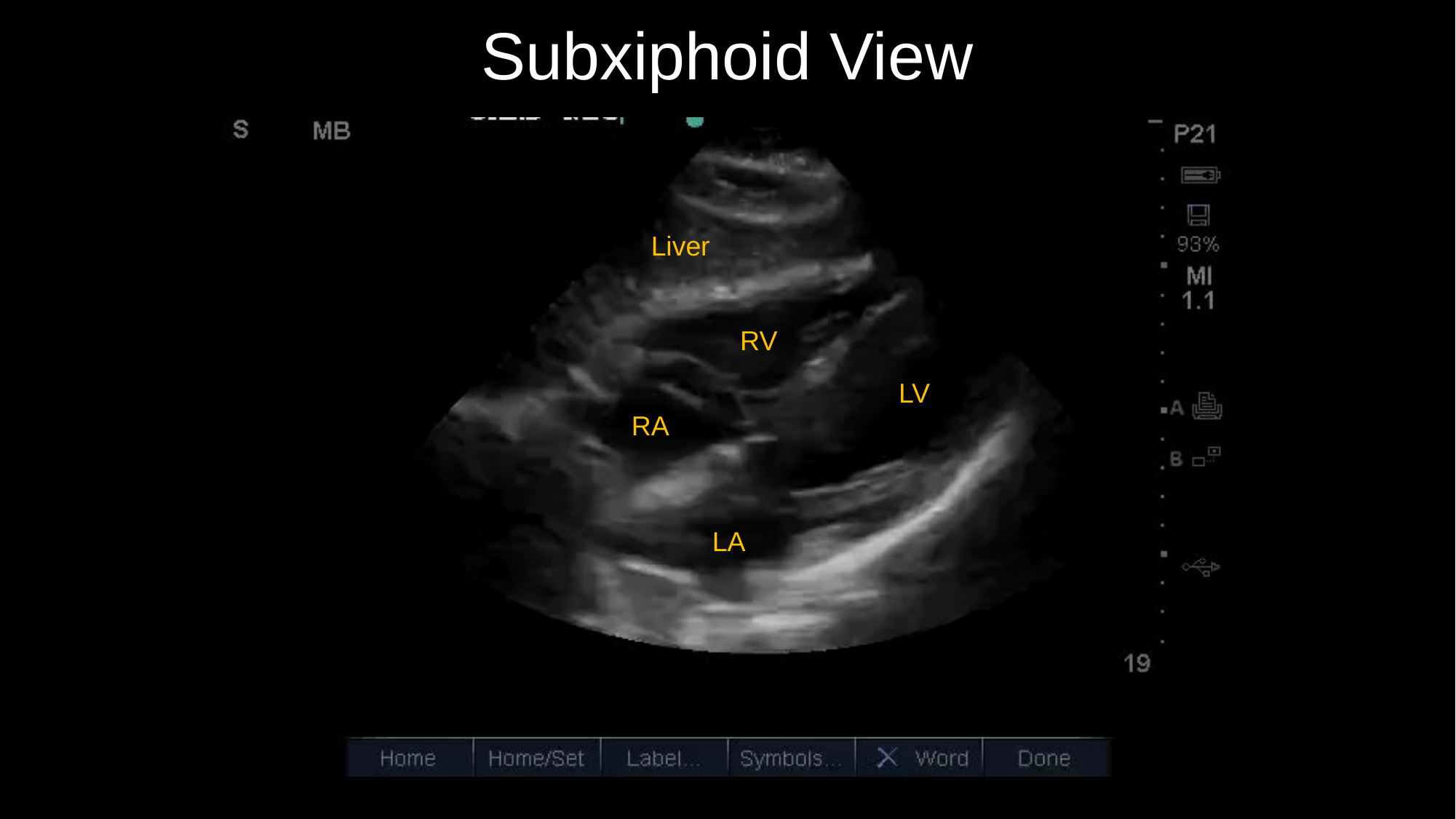

Subxiphoid View
Liver
RV
LV
RA
LA

## Slide 20
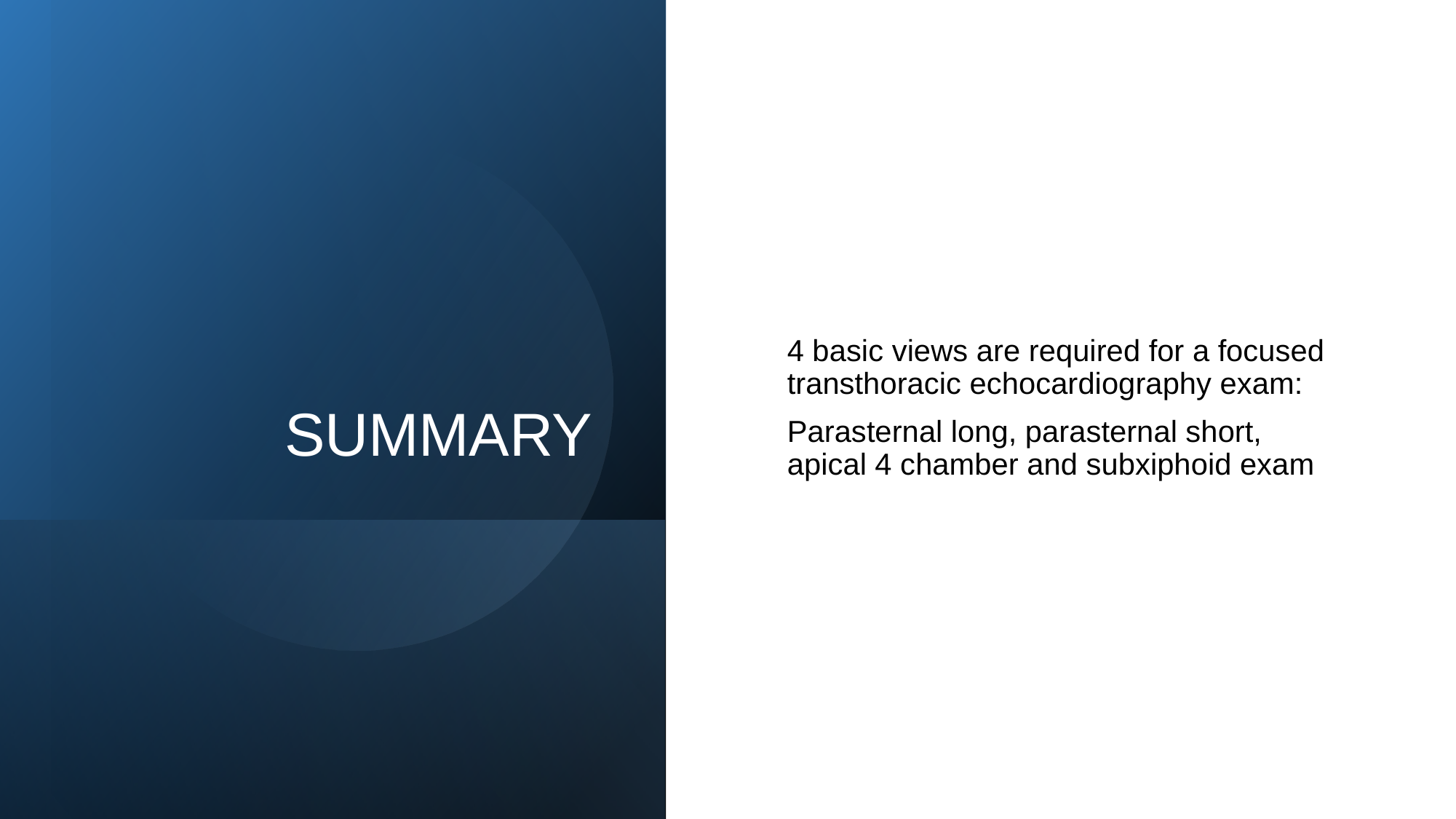

# SUMMARY
4 basic views are required for a focused transthoracic echocardiography exam:
Parasternal long, parasternal short, apical 4 chamber and subxiphoid exam

## Slide 21
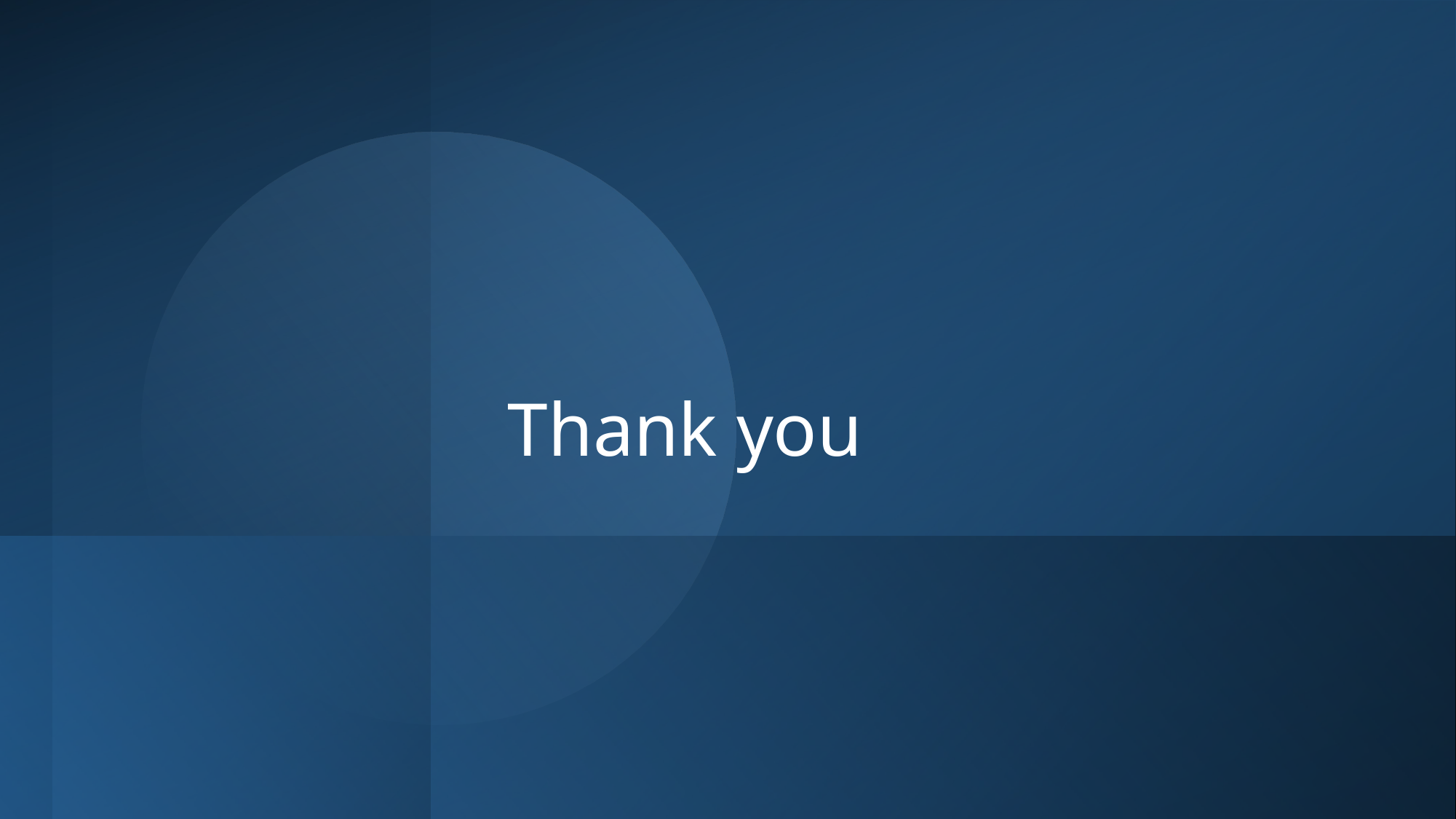

# Thank you
