# Supplementary material for: A point-of-care ultrasound education curriculum for pediatric critical care medicine
Source: Ultrasound J. 2022 Oct 31;14:44. doi: 10.1186/s13089-022-00290-6 (PMC9622960; doi:10.1186/s13089-022-00290-6)
Supplement: Supplementary file 7 — Additional file 7. Hemodynamic assessment of a critically ill patient using point-of-care ultrasound [file 13089_2022_290_MOESM7_ESM.pptx]

## Slide 1
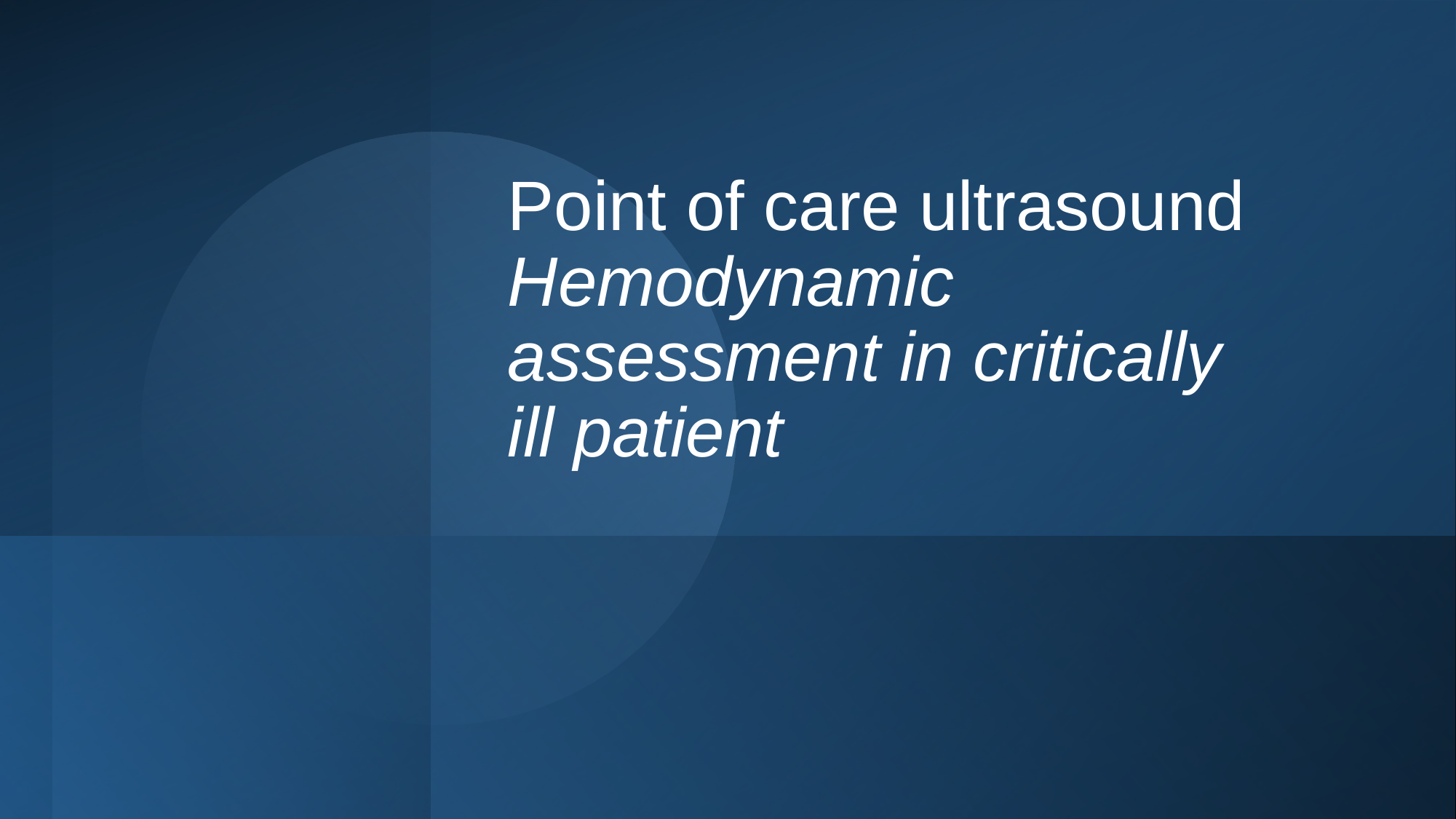

# Point of care ultrasound Hemodynamic assessment in critically ill patient

## Slide 2
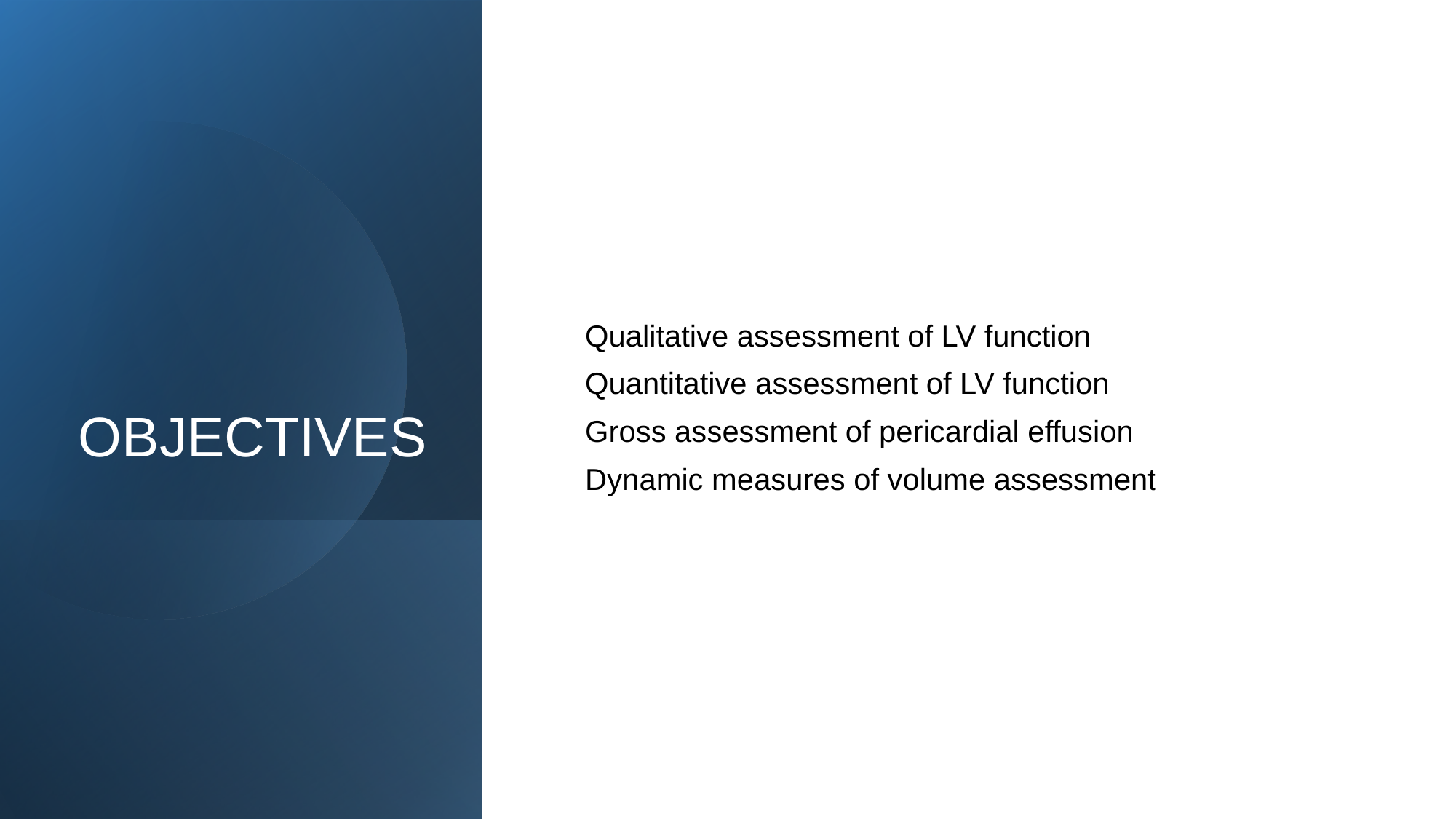

# OBJECTIVES
Qualitative assessment of LV function
Quantitative assessment of LV function
Gross assessment of pericardial effusion
Dynamic measures of volume assessment

## Slide 3
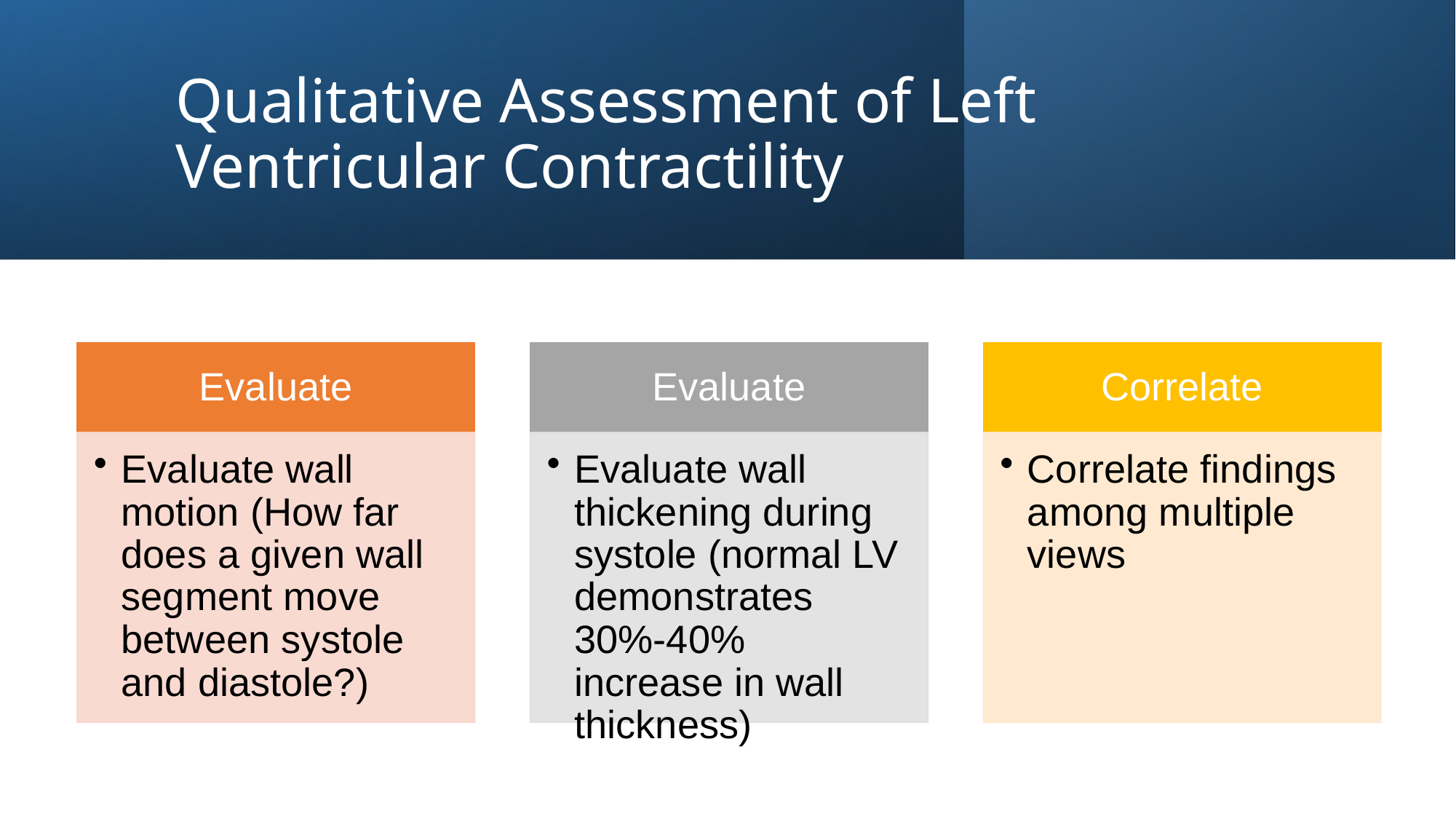

# Qualitative Assessment of Left Ventricular Contractility

## Slide 4
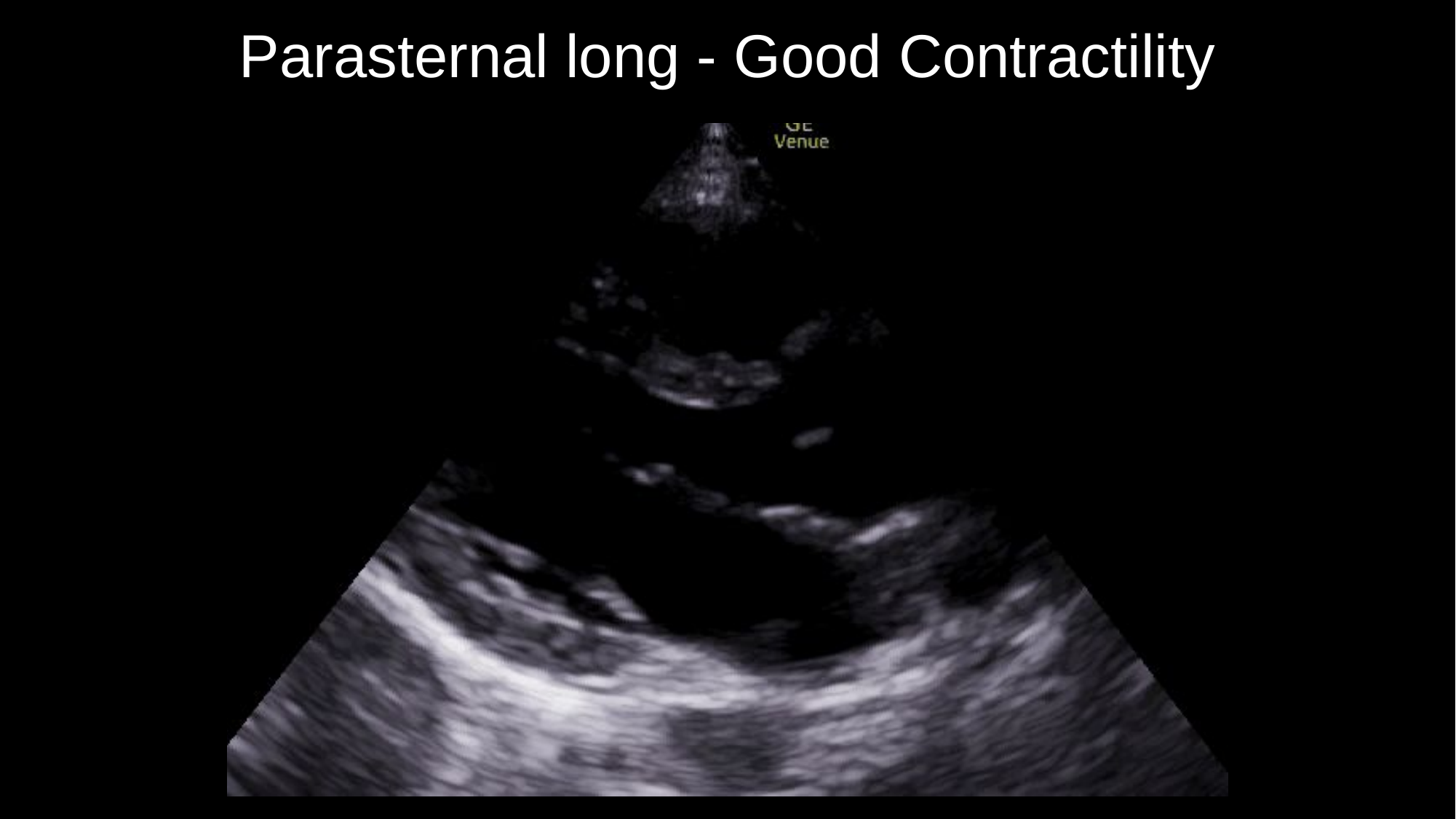

Parasternal long - Good Contractility

## Slide 5
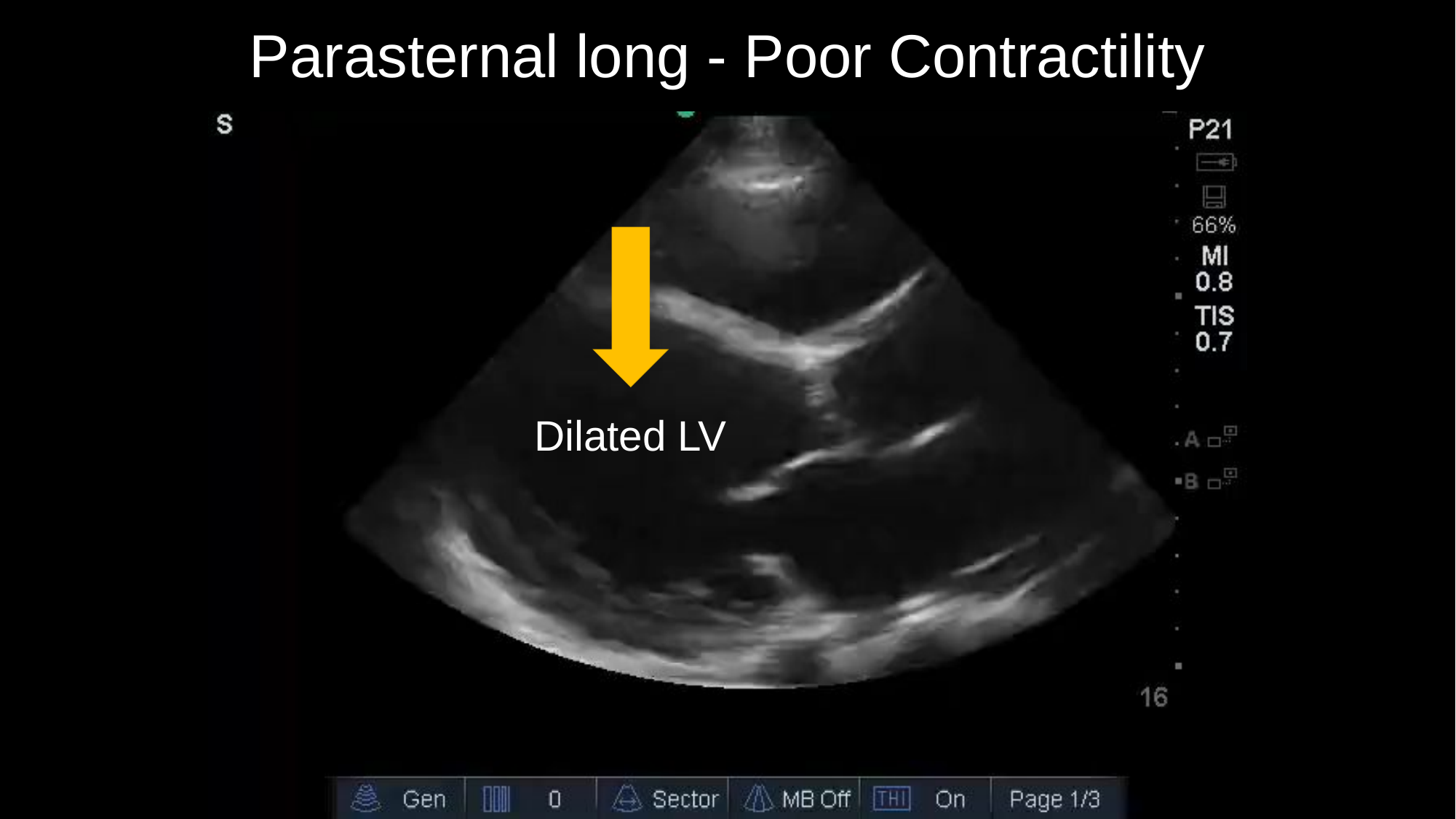

# Parasternal Long View
Parasternal long - Poor Contractility
Dilated LV

## Slide 6
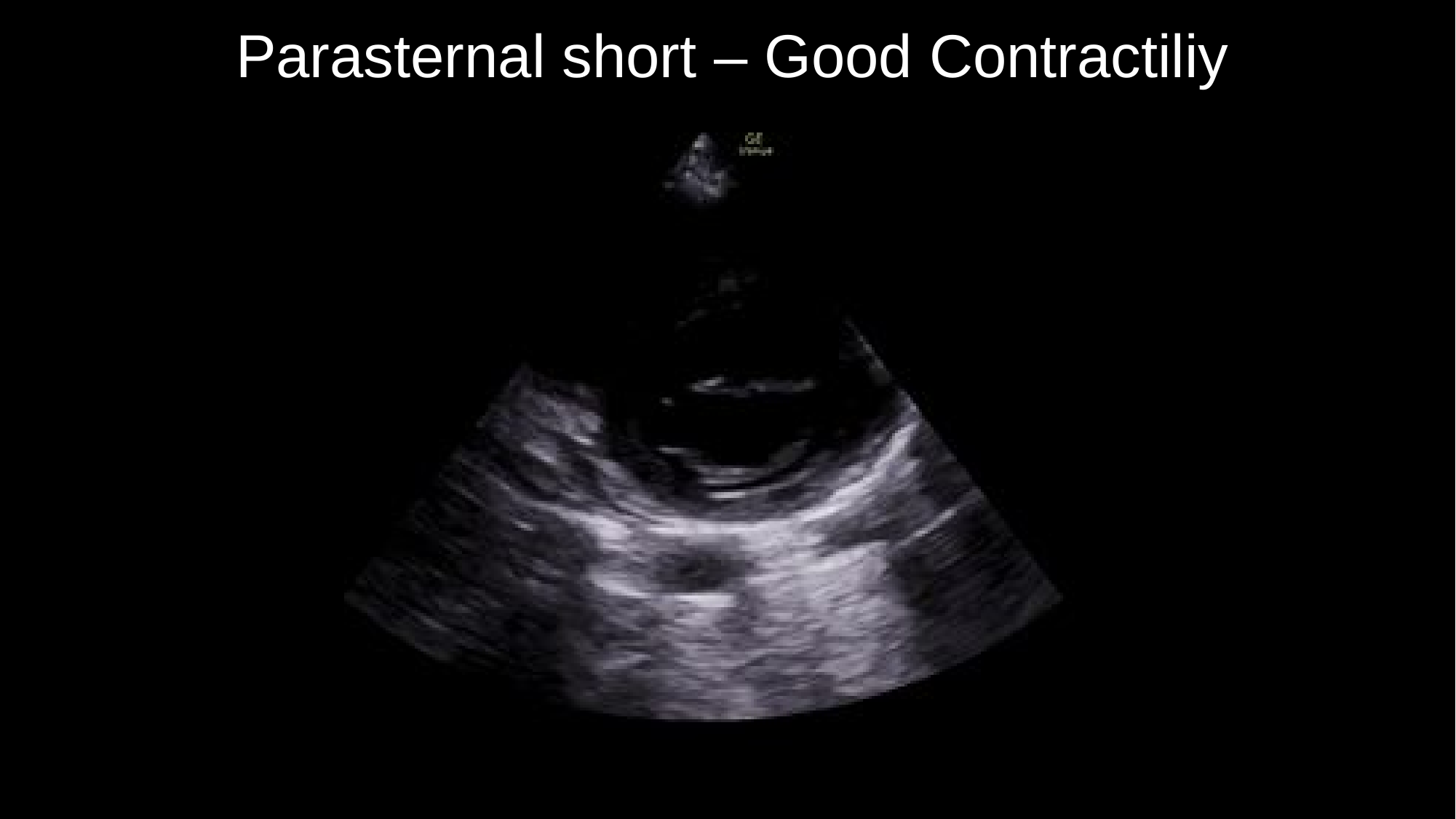

Parasternal short – Good Contractiliy

## Slide 7
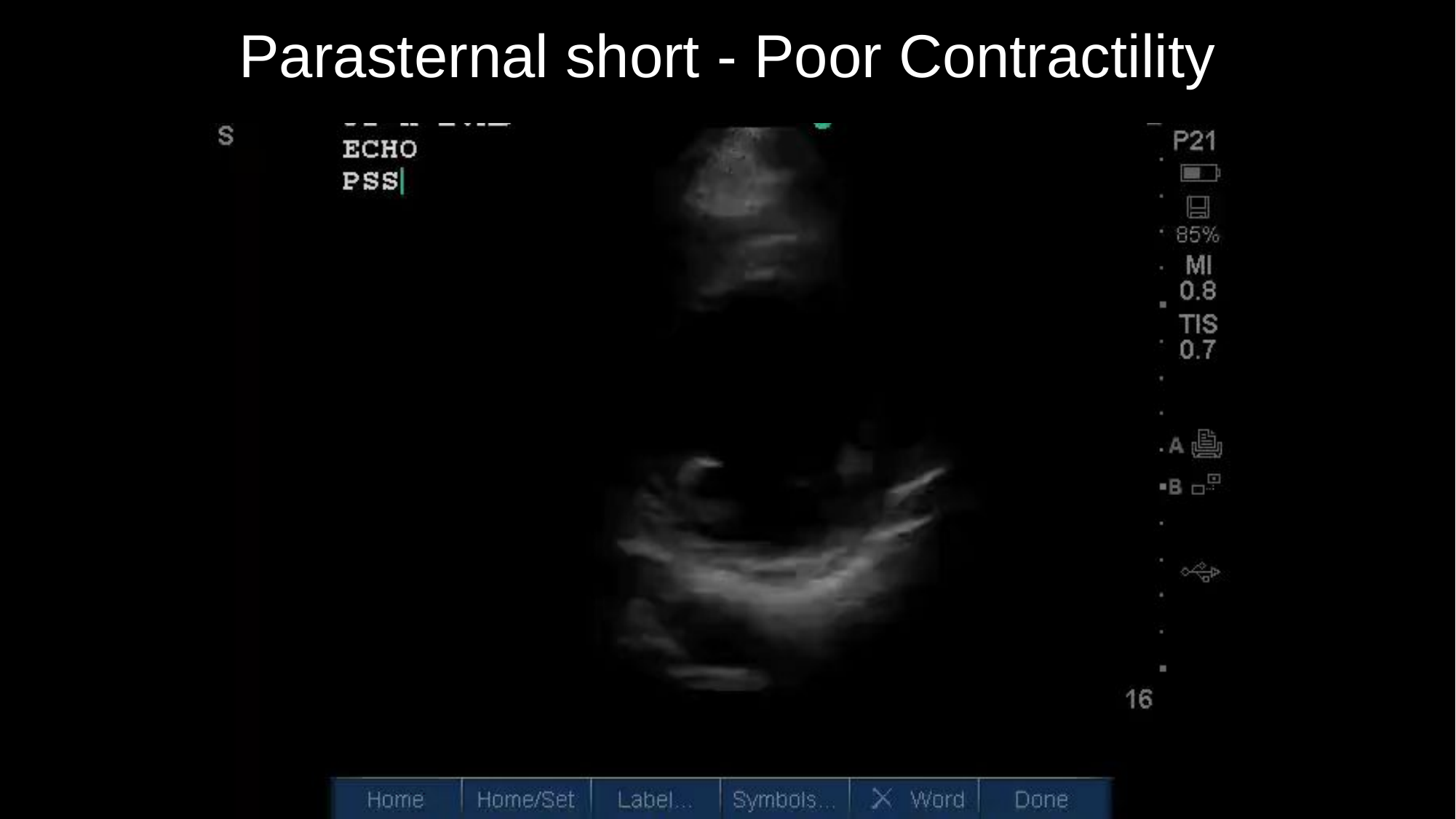

Parasternal short - Poor Contractility
# Parasternal Short View

## Slide 8
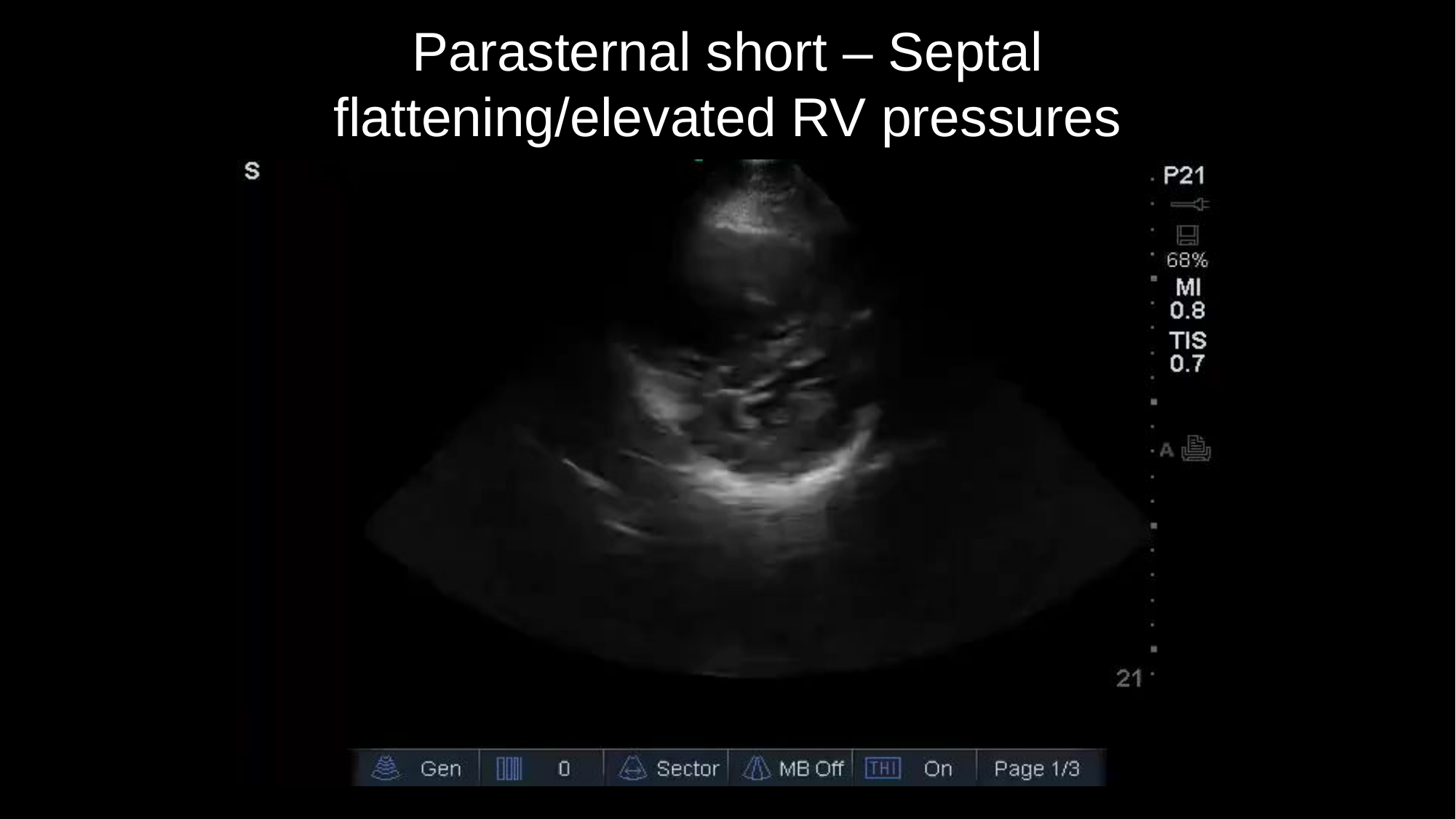

Parasternal short – Septal flattening/elevated RV pressures
# Parasternal Short View

## Slide 9
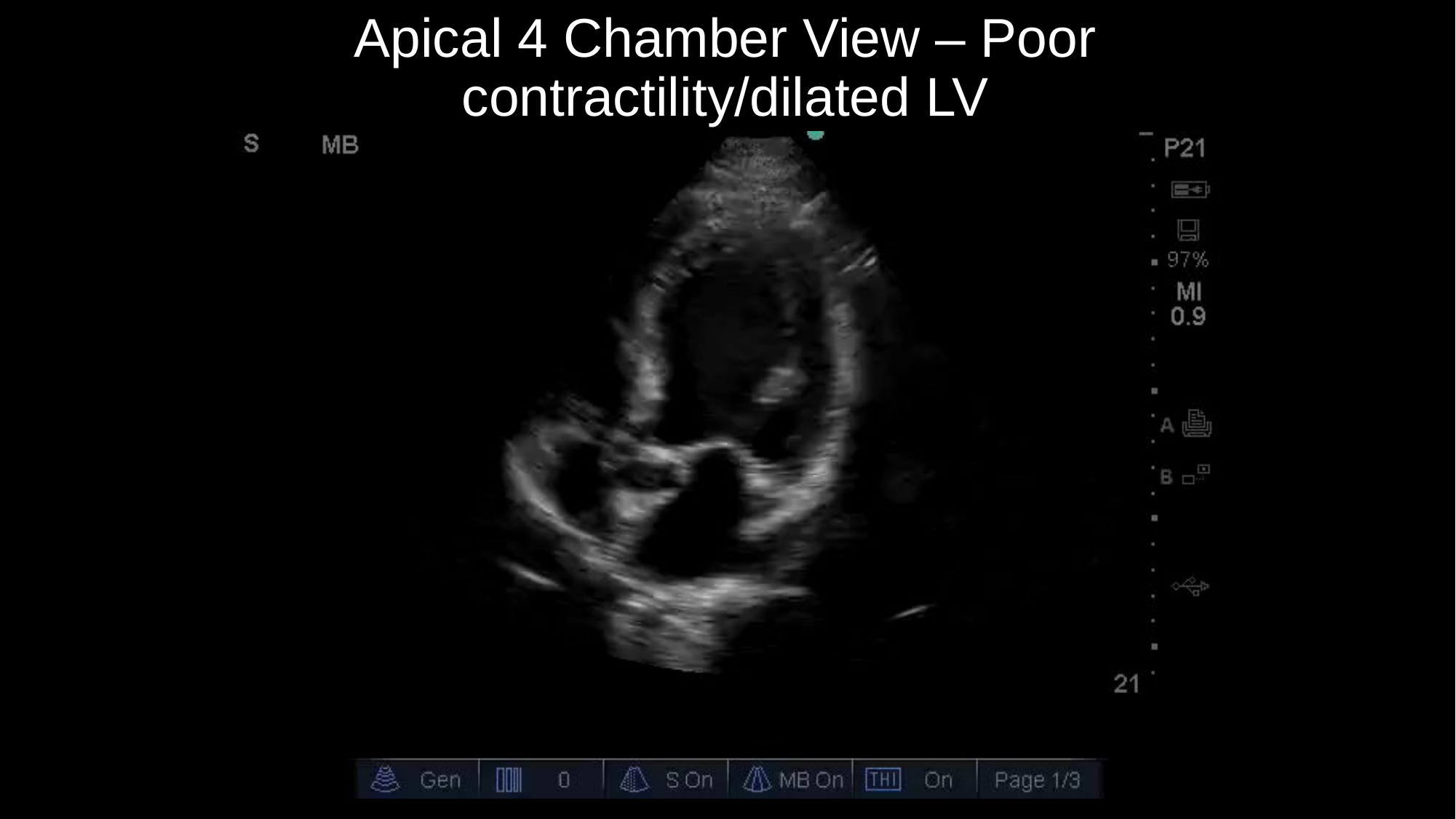

# Apical 4 Chamber View – Poor contractility/dilated LV

## Slide 10
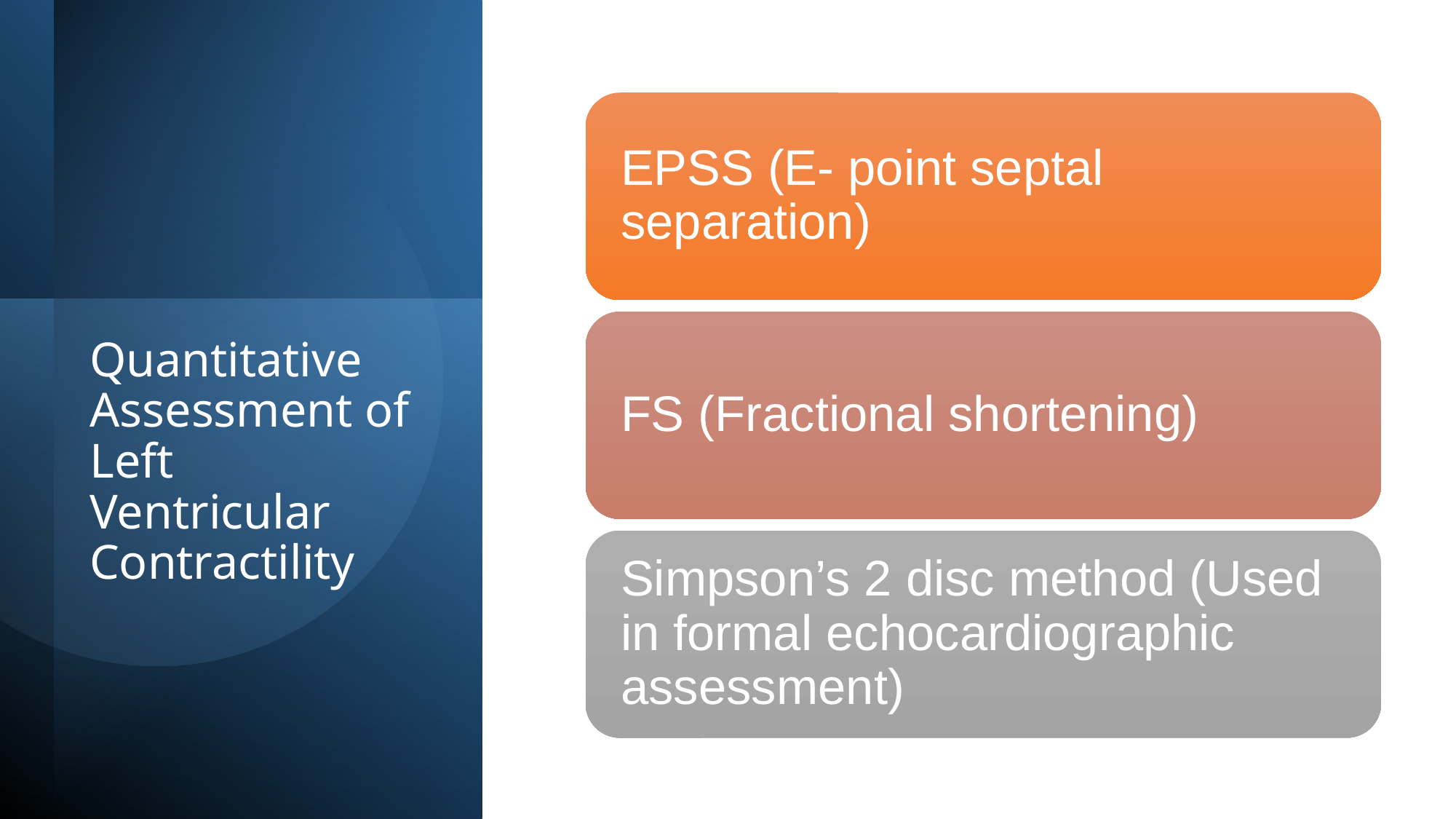

# Quantitative Assessment of Left Ventricular Contractility

## Slide 11
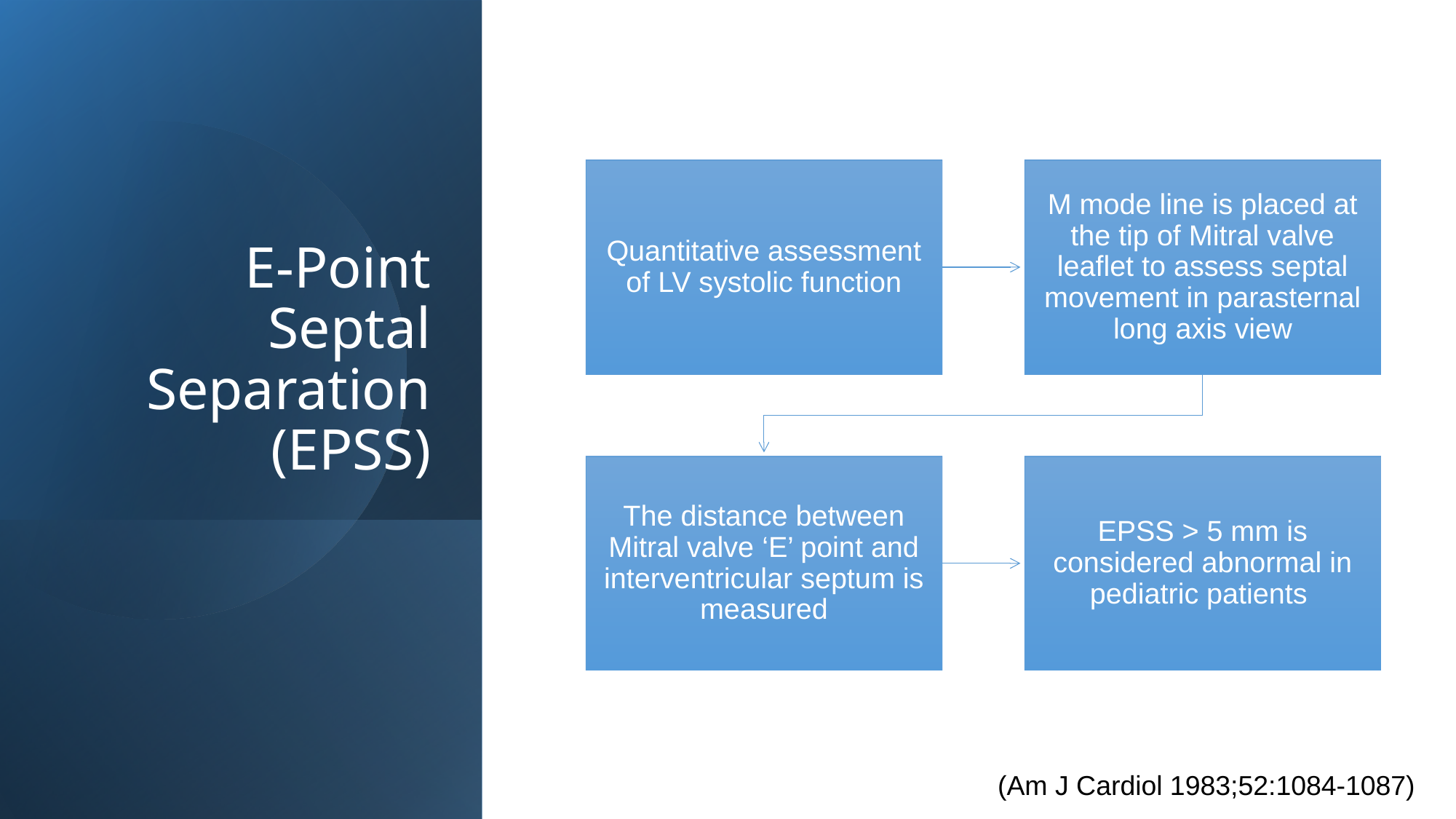

# E-Point Septal Separation (EPSS)
(Am J Cardiol 1983;52:1084-1087)

## Slide 12
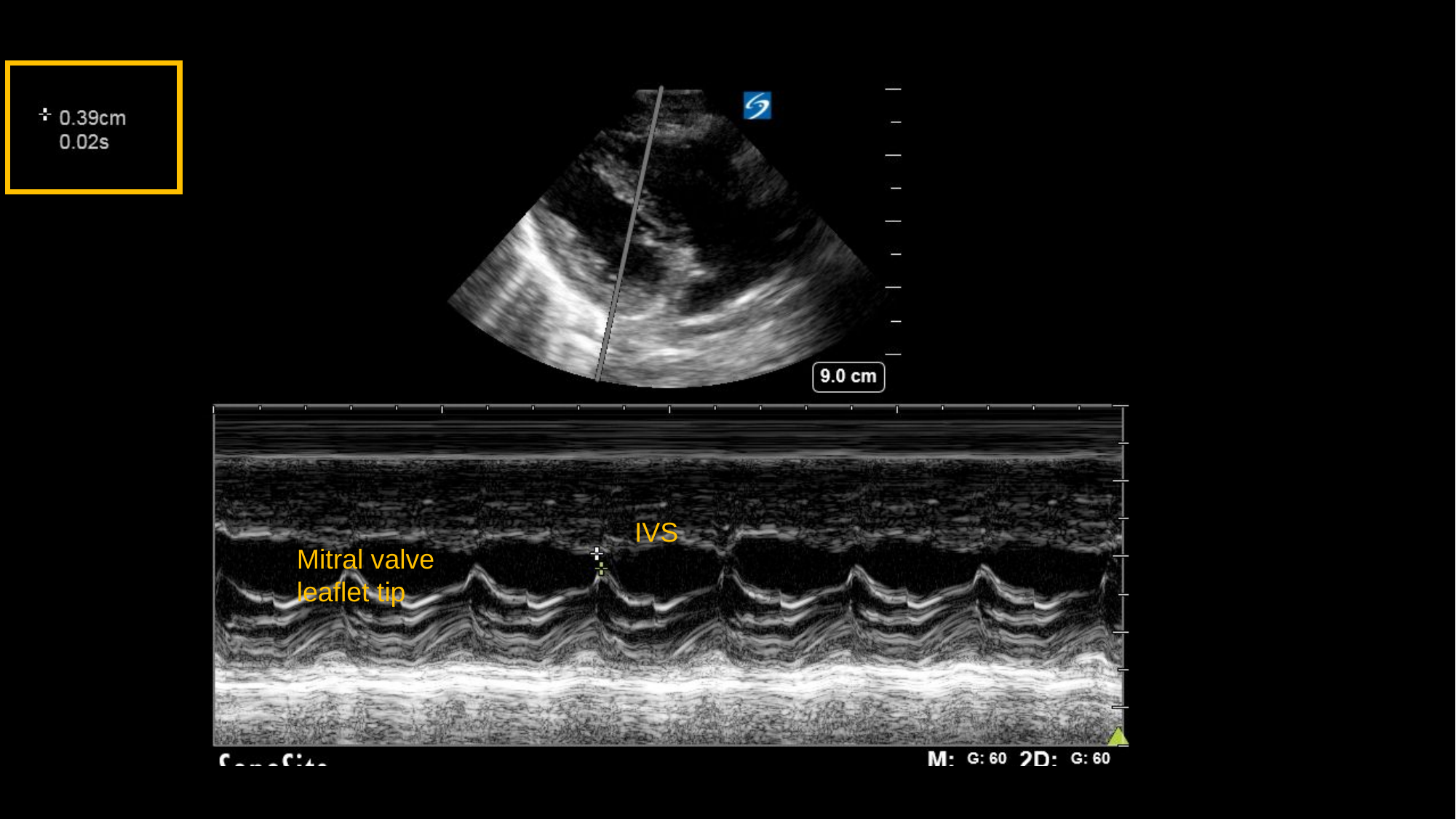

IVS
Mitral valve leaflet tip

## Slide 13
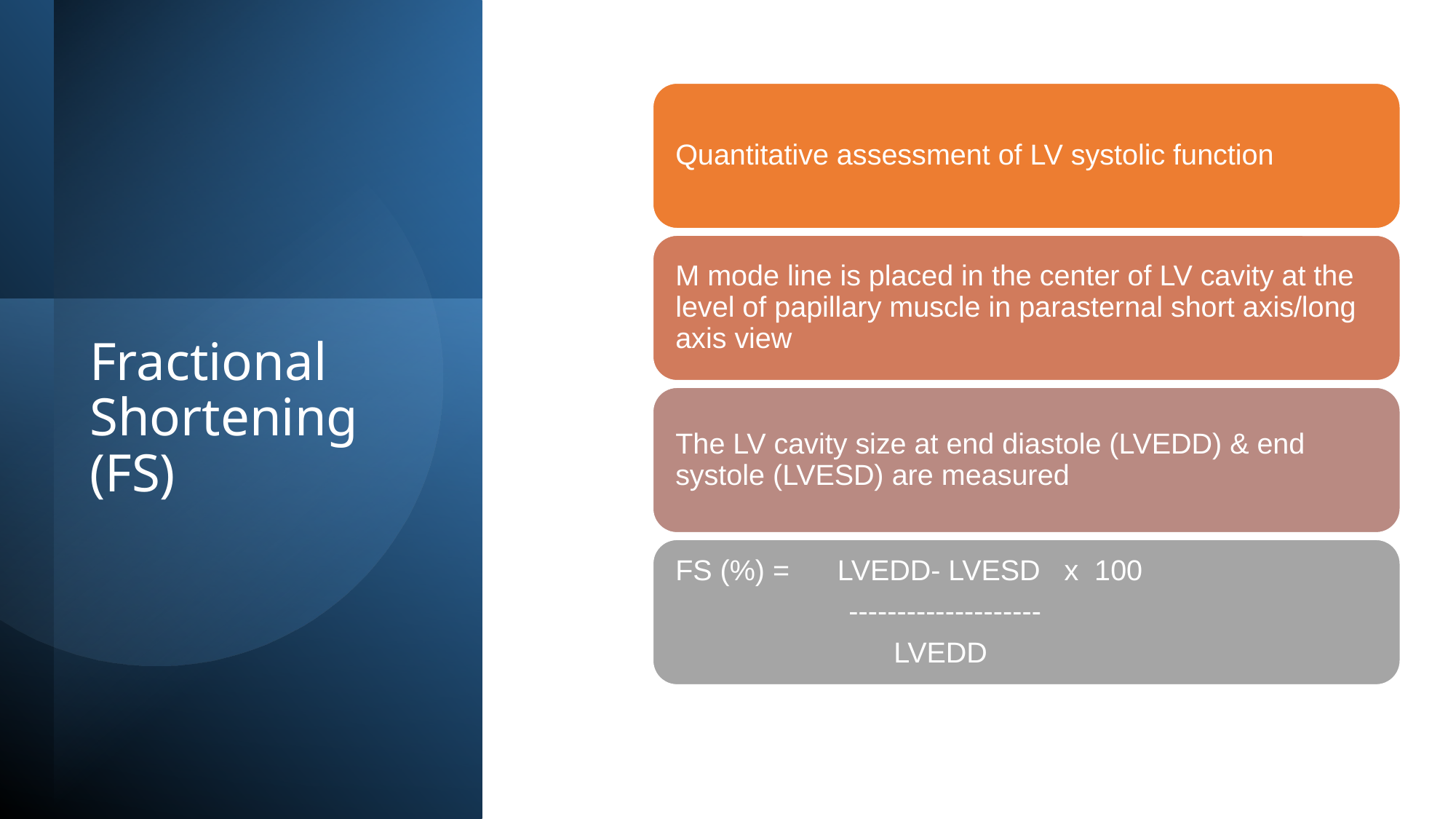

# Fractional Shortening (FS)

## Slide 14
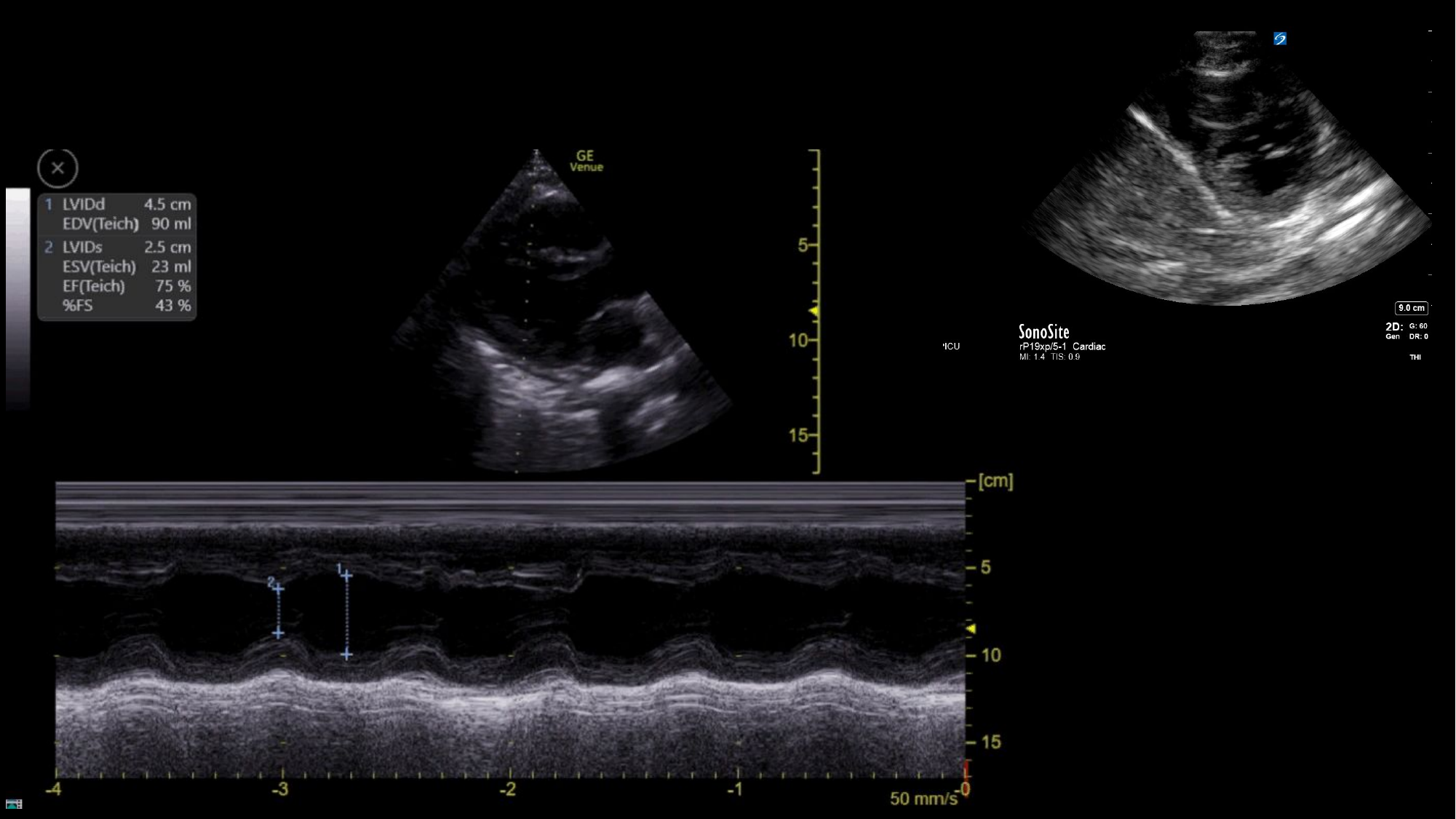

## Slide 15
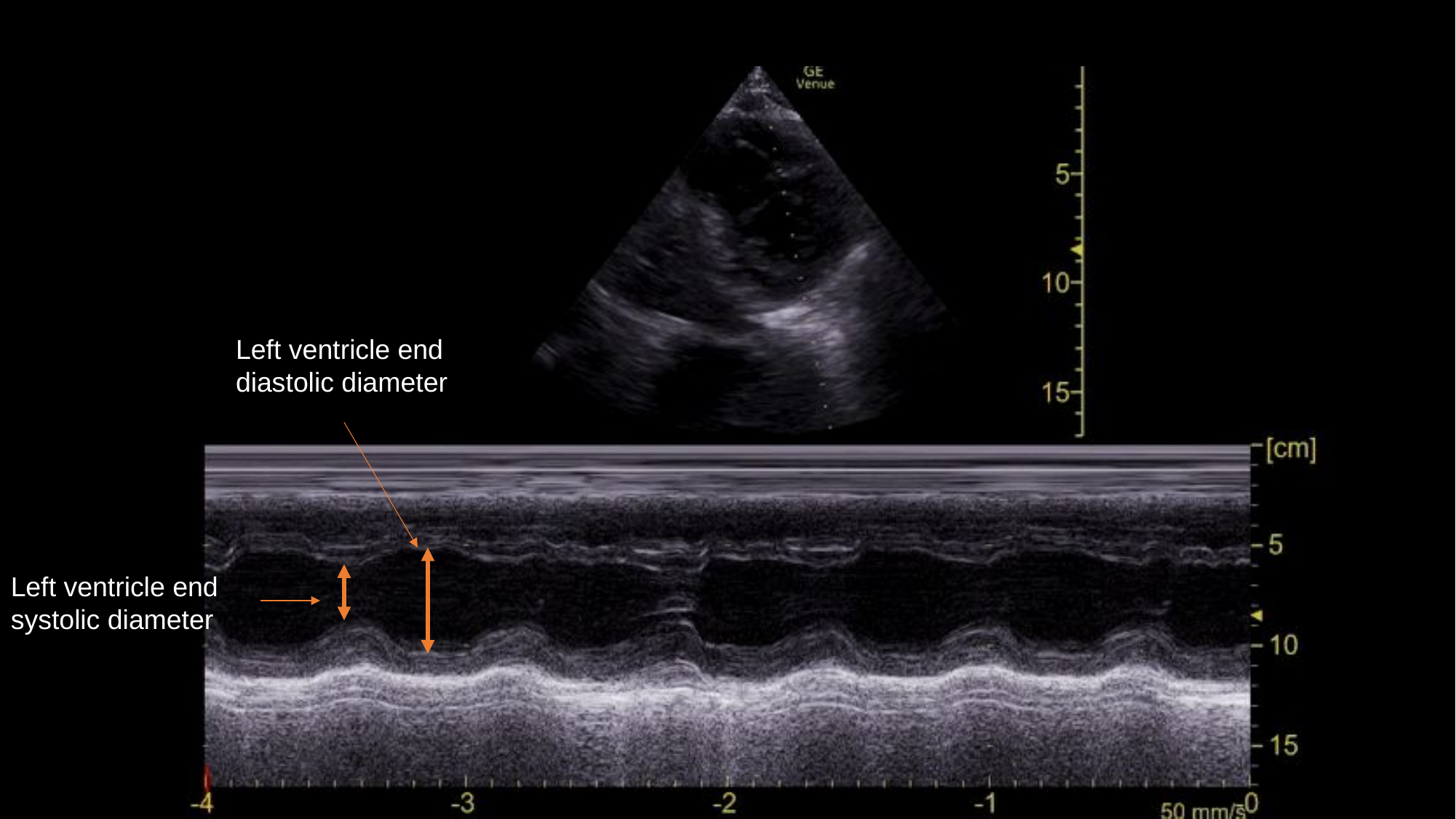

Left ventricle end diastolic diameter
Left ventricle end systolic diameter

## Slide 16
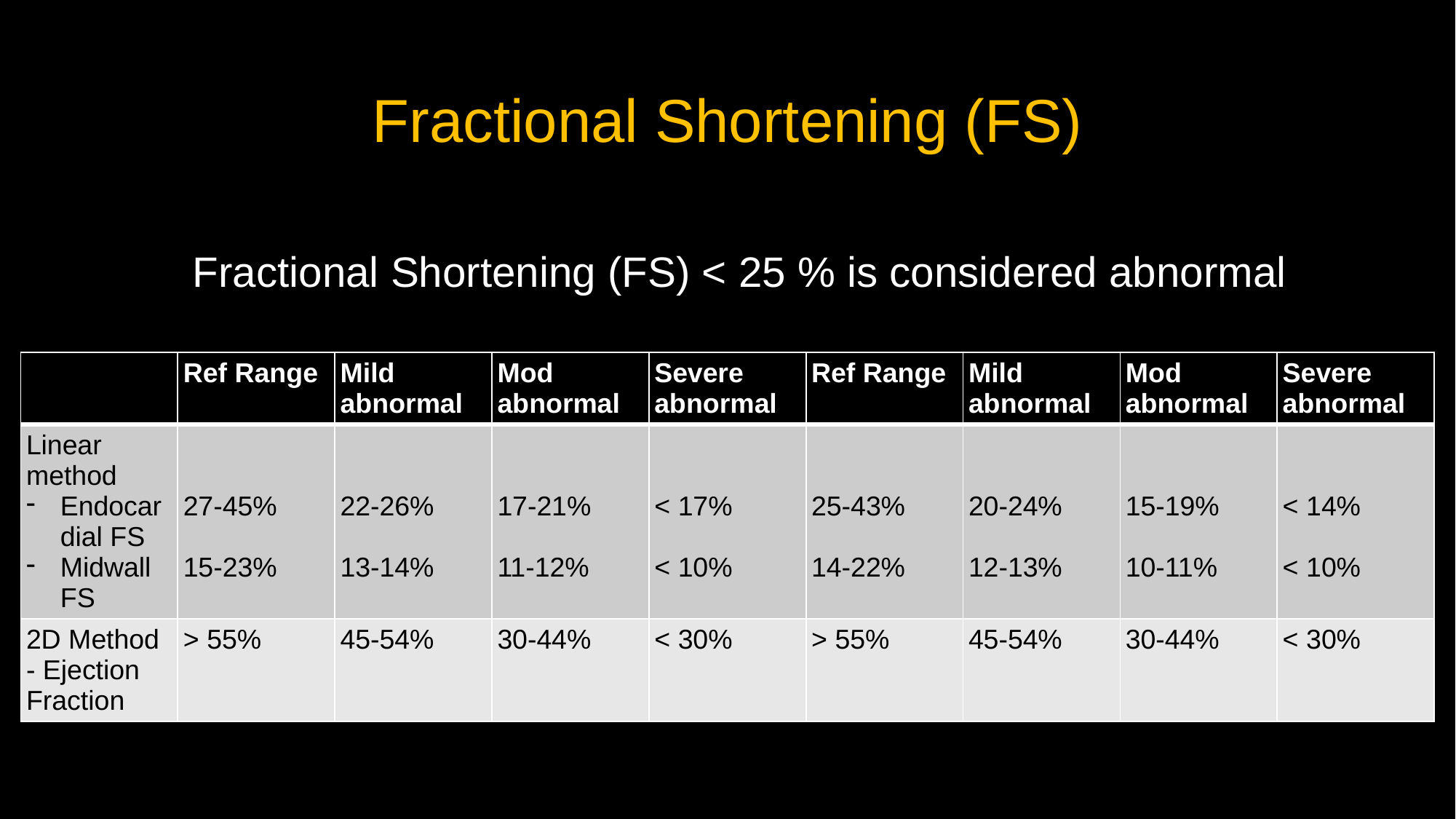

# Fractional Shortening (FS)
Fractional Shortening (FS) < 25 % is considered abnormal
| | Ref Range | Mild abnormal | Mod abnormal | Severe abnormal | Ref Range | Mild abnormal | Mod abnormal | Severe abnormal |
| --- | --- | --- | --- | --- | --- | --- | --- | --- |
| Linear method Endocardial FS Midwall FS | 27-45% 15-23% | 22-26% 13-14% | 17-21% 11-12% | < 17% < 10% | 25-43% 14-22% | 20-24% 12-13% | 15-19% 10-11% | < 14% < 10% |
| 2D Method - Ejection Fraction | > 55% | 45-54% | 30-44% | < 30% | > 55% | 45-54% | 30-44% | < 30% |

## Slide 17
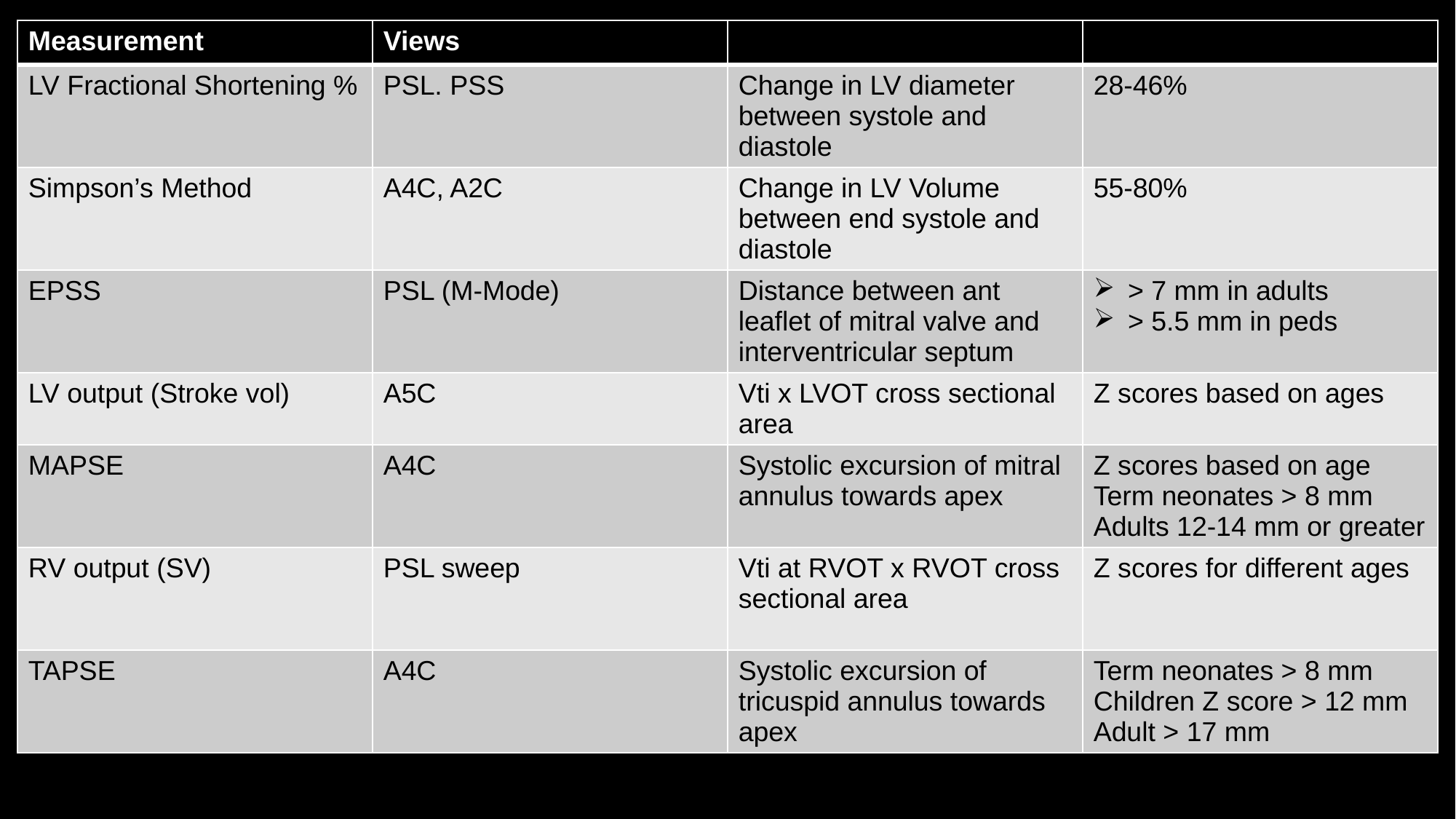

| Measurement | Views | | |
| --- | --- | --- | --- |
| LV Fractional Shortening % | PSL. PSS | Change in LV diameter between systole and diastole | 28-46% |
| Simpson’s Method | A4C, A2C | Change in LV Volume between end systole and diastole | 55-80% |
| EPSS | PSL (M-Mode) | Distance between ant leaflet of mitral valve and interventricular septum | > 7 mm in adults > 5.5 mm in peds |
| LV output (Stroke vol) | A5C | Vti x LVOT cross sectional area | Z scores based on ages |
| MAPSE | A4C | Systolic excursion of mitral annulus towards apex | Z scores based on age Term neonates > 8 mm Adults 12-14 mm or greater |
| RV output (SV) | PSL sweep | Vti at RVOT x RVOT cross sectional area | Z scores for different ages |
| TAPSE | A4C | Systolic excursion of tricuspid annulus towards apex | Term neonates > 8 mm Children Z score > 12 mm Adult > 17 mm |
# Normative Values

## Slide 18
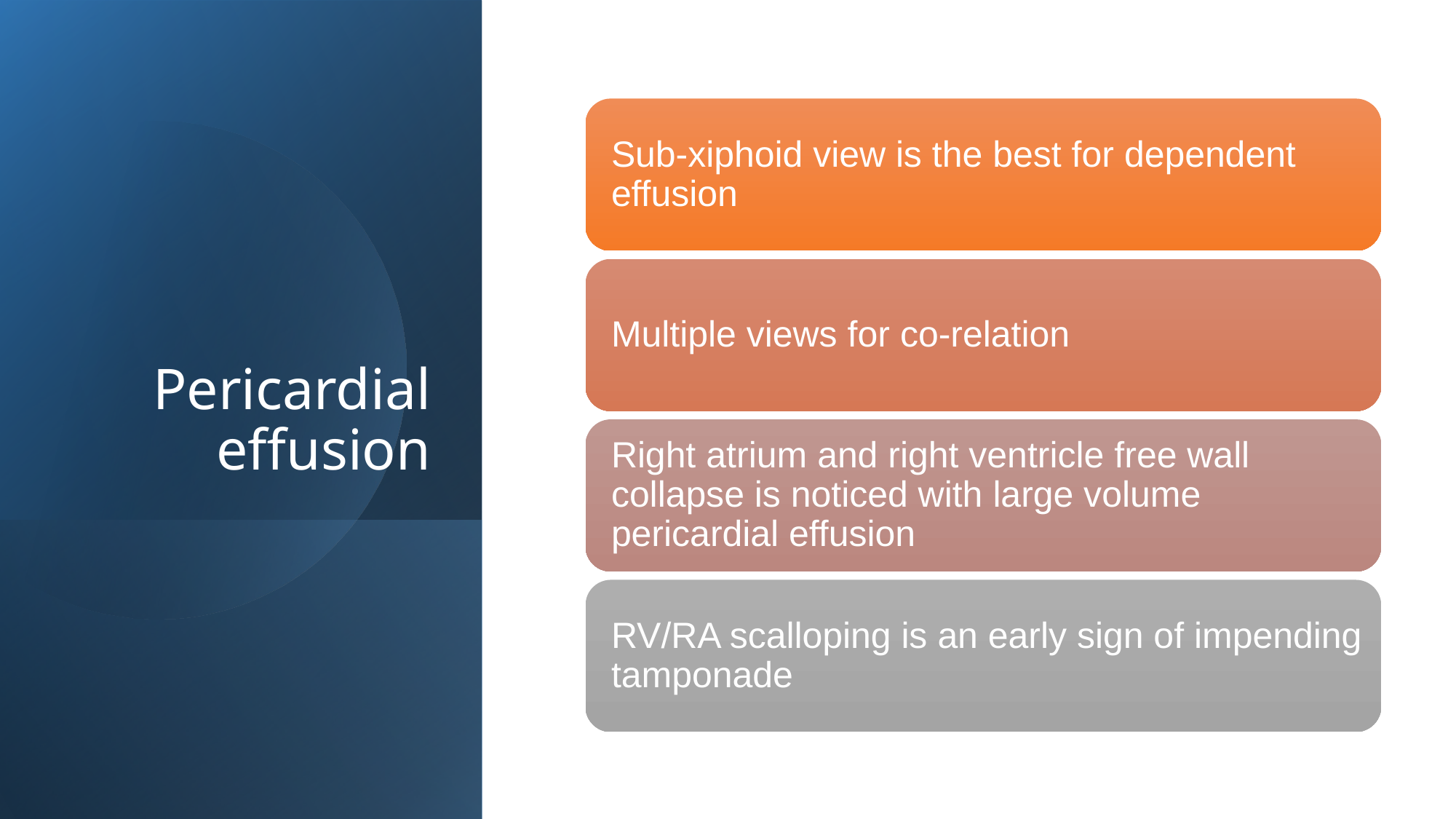

# Pericardial effusion

## Slide 19
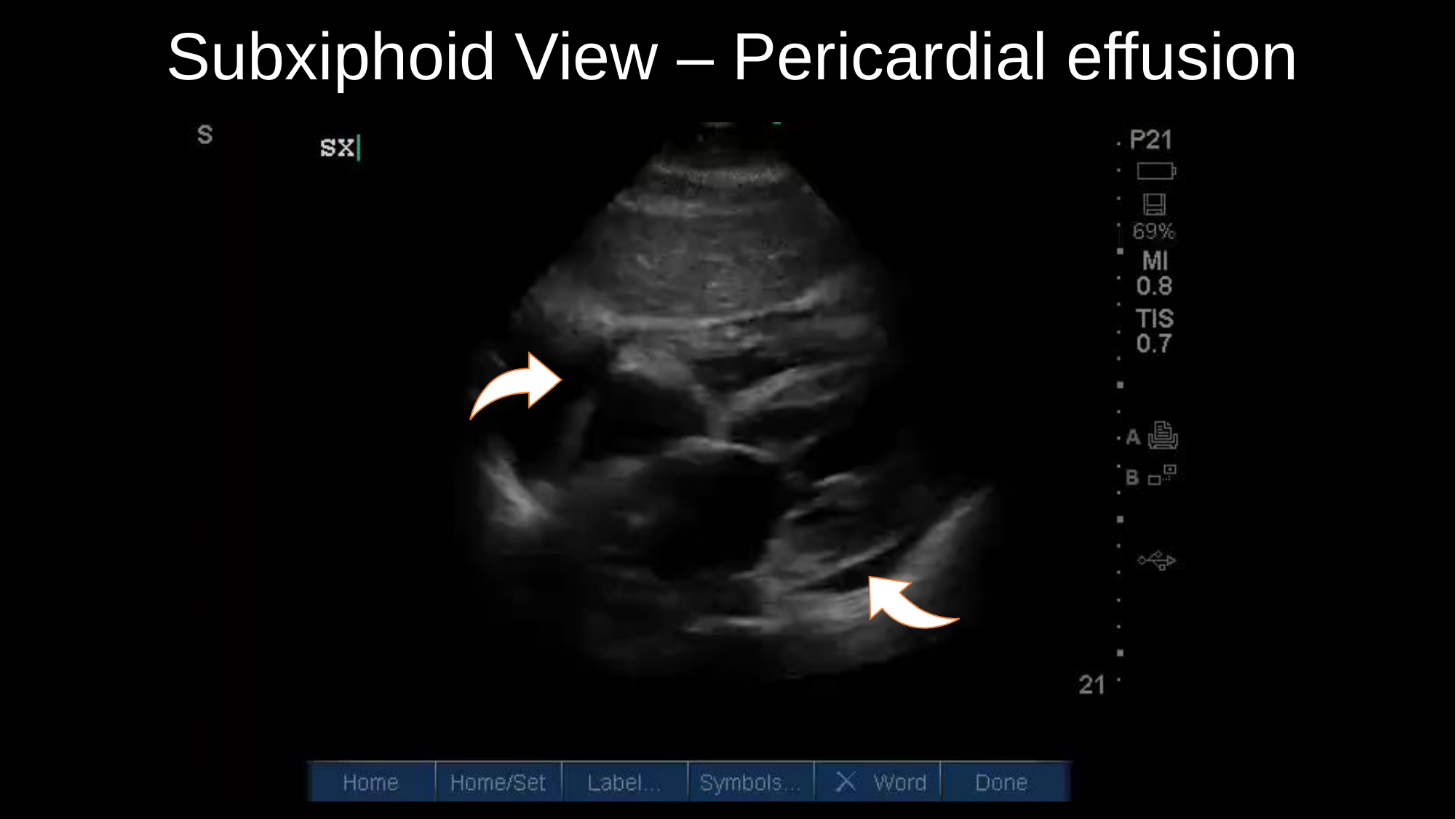

Subxiphoid View – Pericardial effusion

## Slide 20
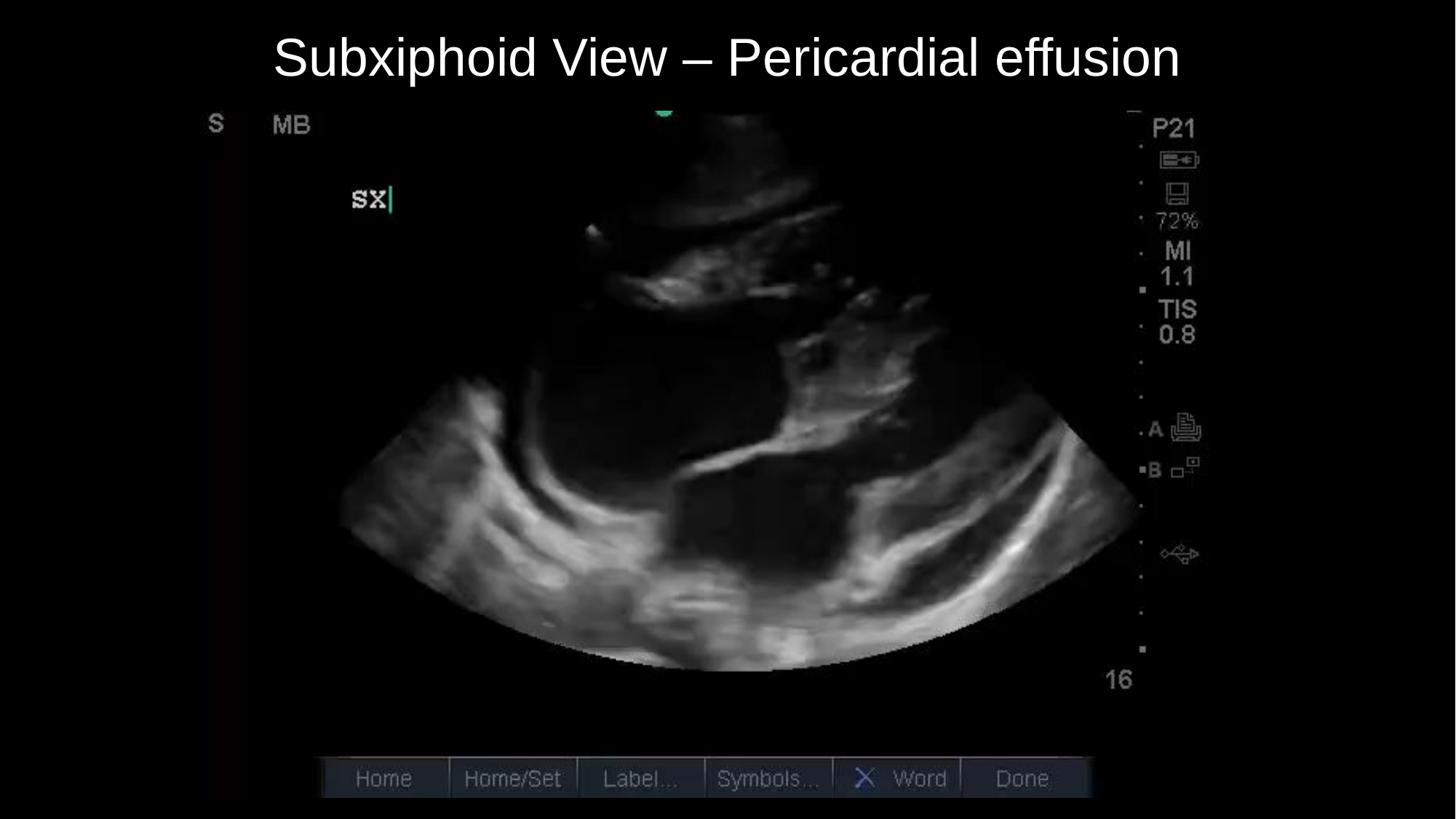

# Subxiphoid View – Pericardial effusion

## Slide 21
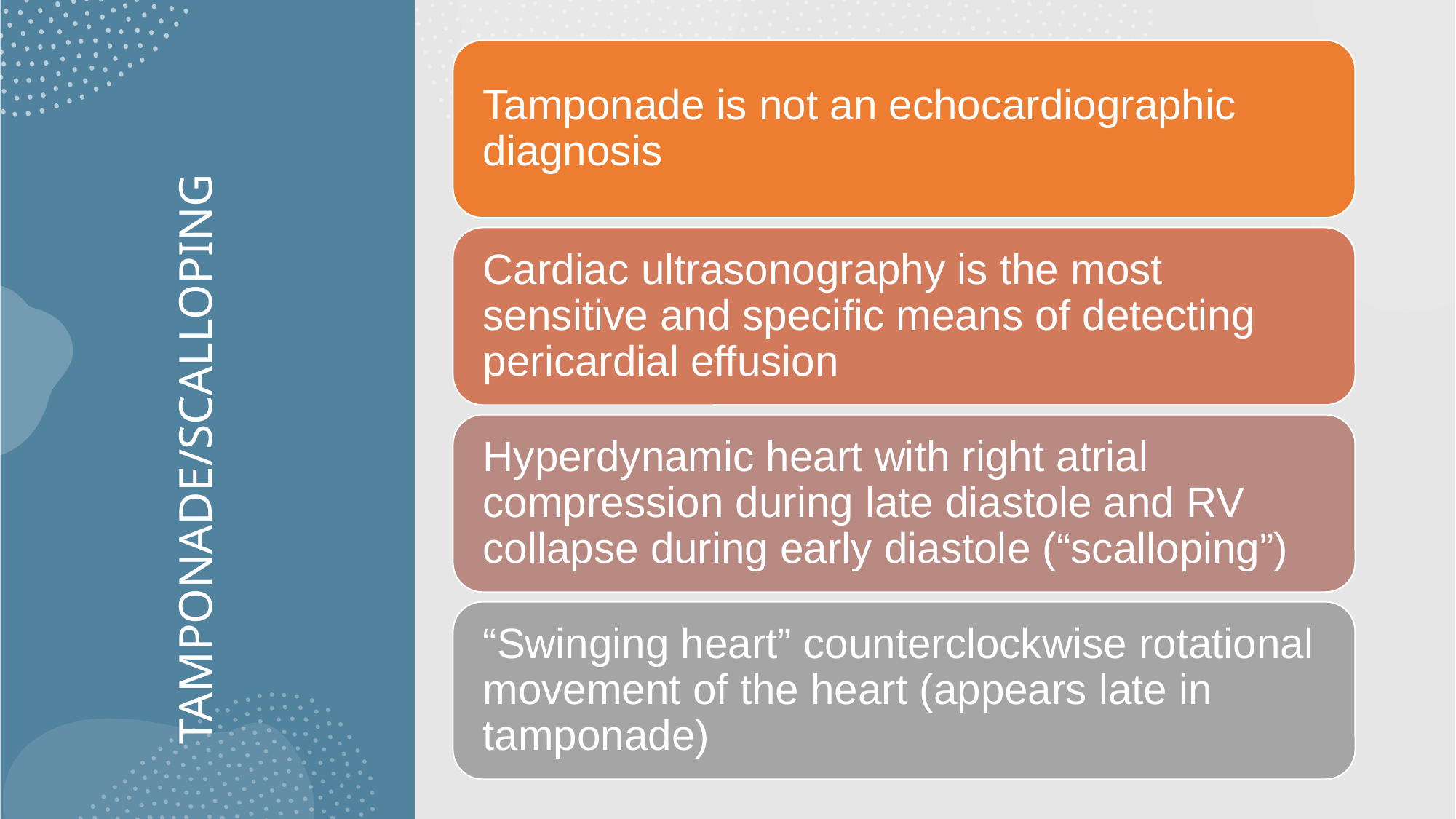

# TAMPONADE/SCALLOPING

## Slide 22
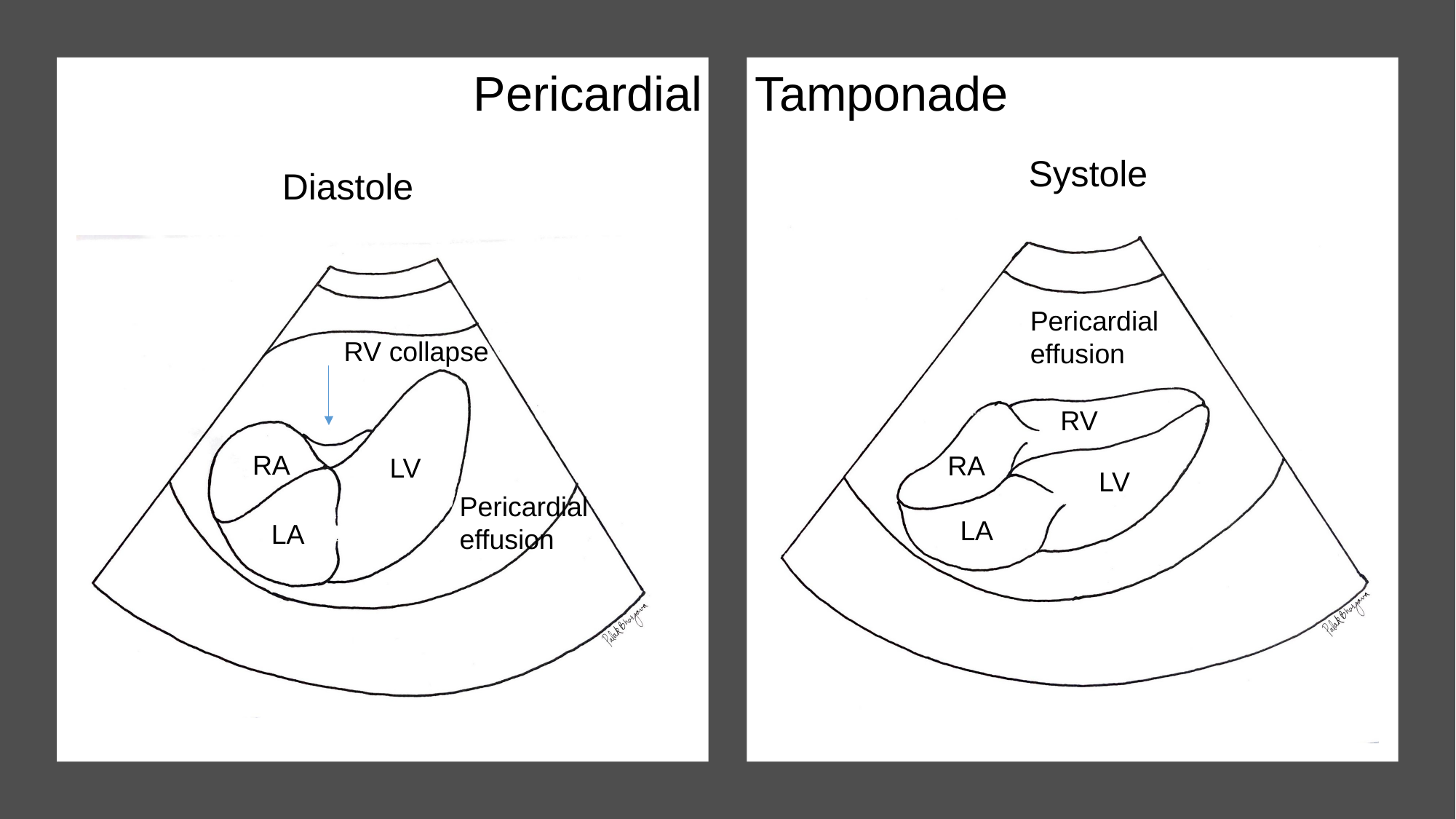

Pericardial Tamponade
Systole
Diastole
Pericardial effusion
RV collapse
RV
RA
RA
LV
LV
Pericardial effusion
LA
LA

## Slide 23
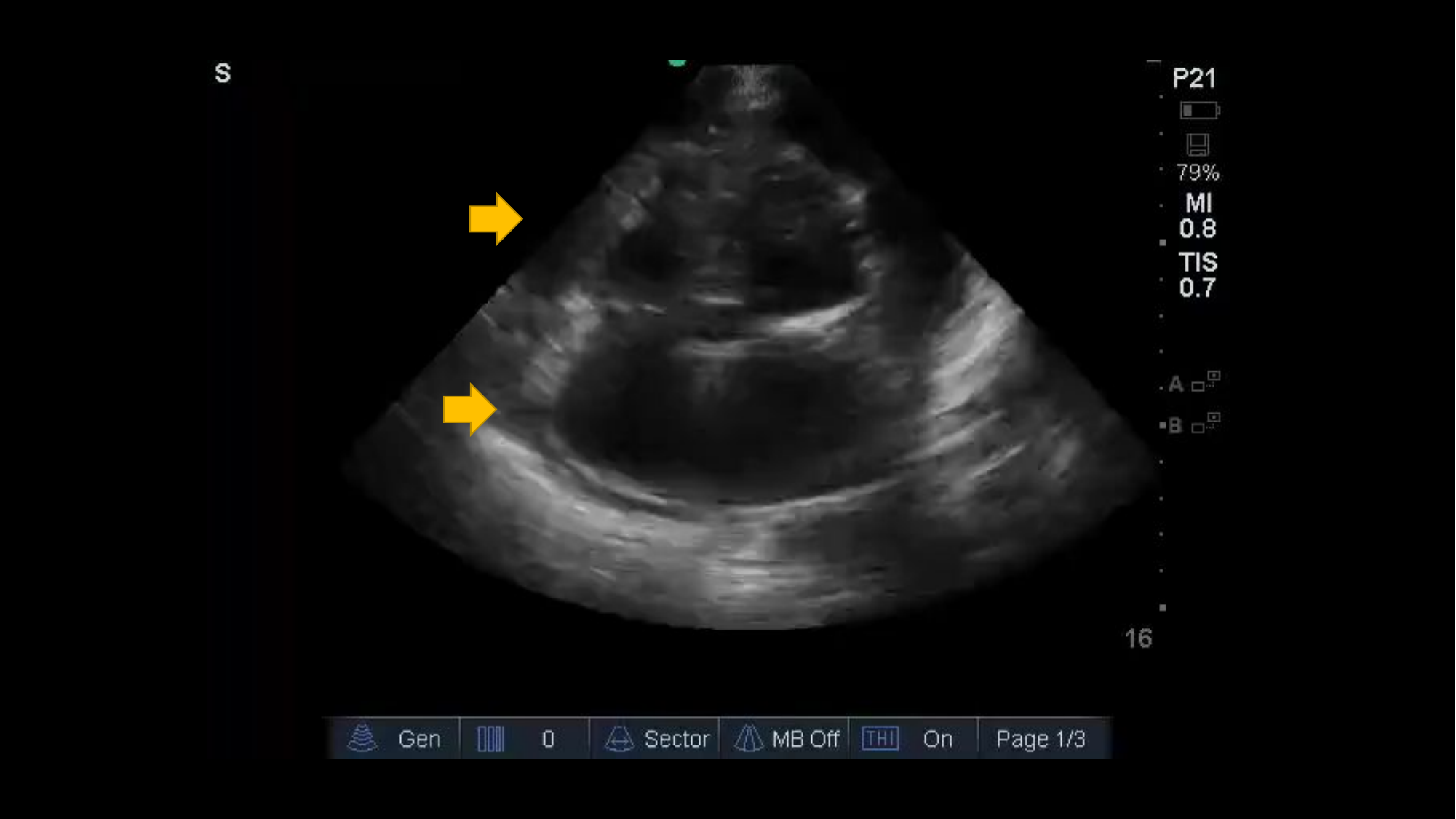

## Slide 24
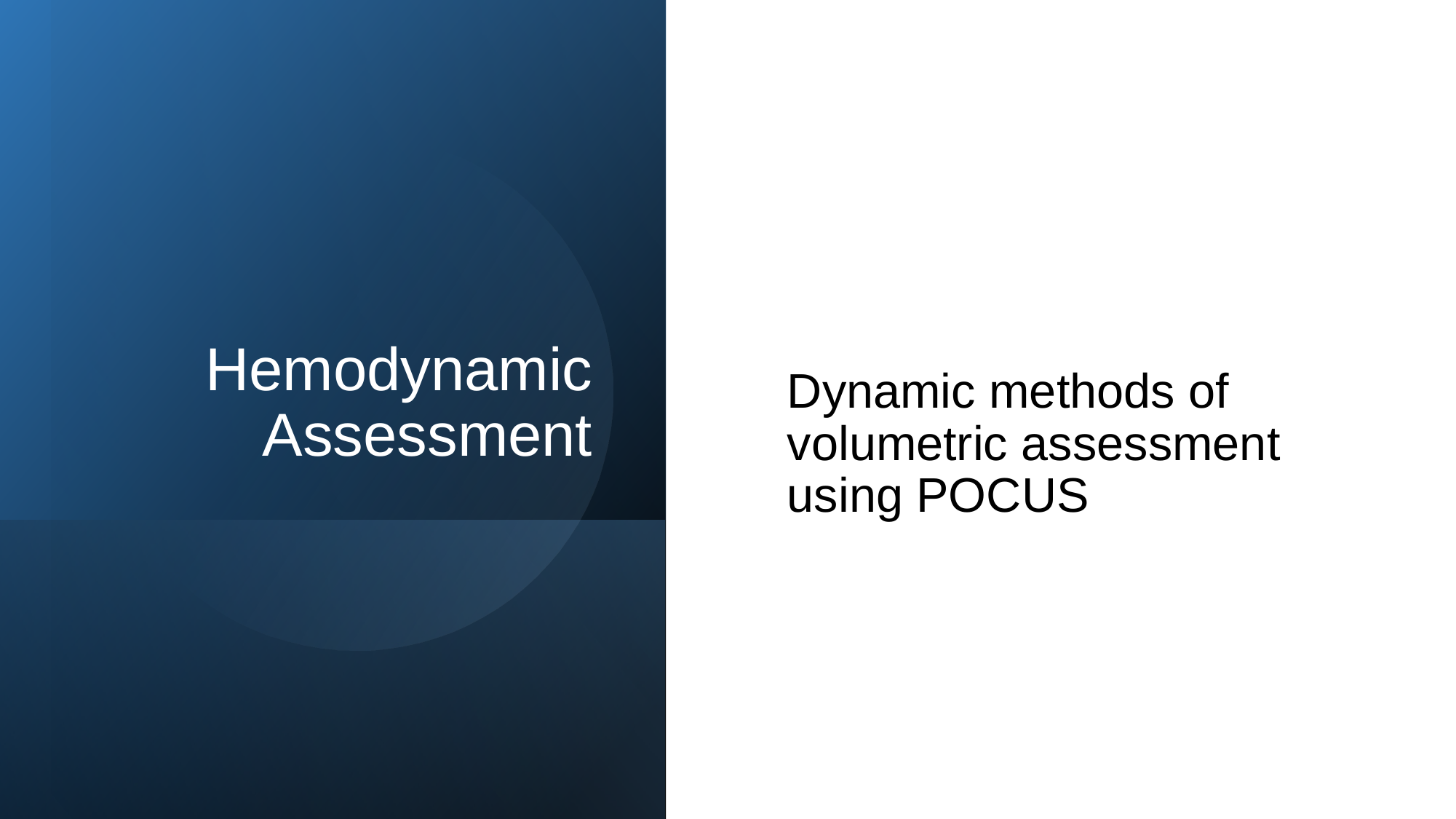

# Hemodynamic Assessment
Dynamic methods of volumetric assessment using POCUS

## Slide 25
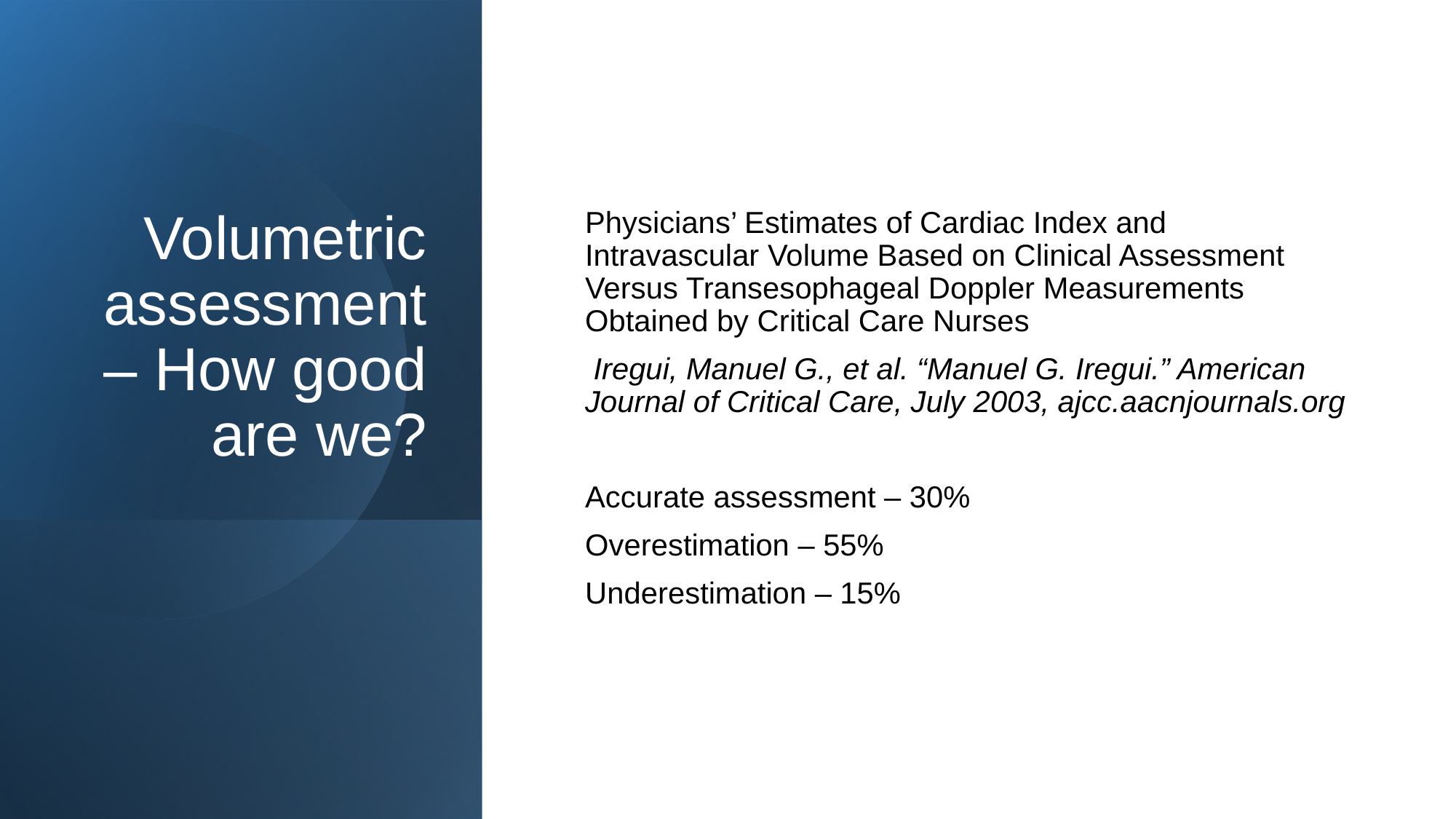

# Volumetric assessment – How good are we?
Physicians’ Estimates of Cardiac Index and Intravascular Volume Based on Clinical Assessment Versus Transesophageal Doppler Measurements Obtained by Critical Care Nurses
 Iregui, Manuel G., et al. “Manuel G. Iregui.” American Journal of Critical Care, July 2003, ajcc.aacnjournals.org
Accurate assessment – 30%
Overestimation – 55%
Underestimation – 15%

## Slide 26
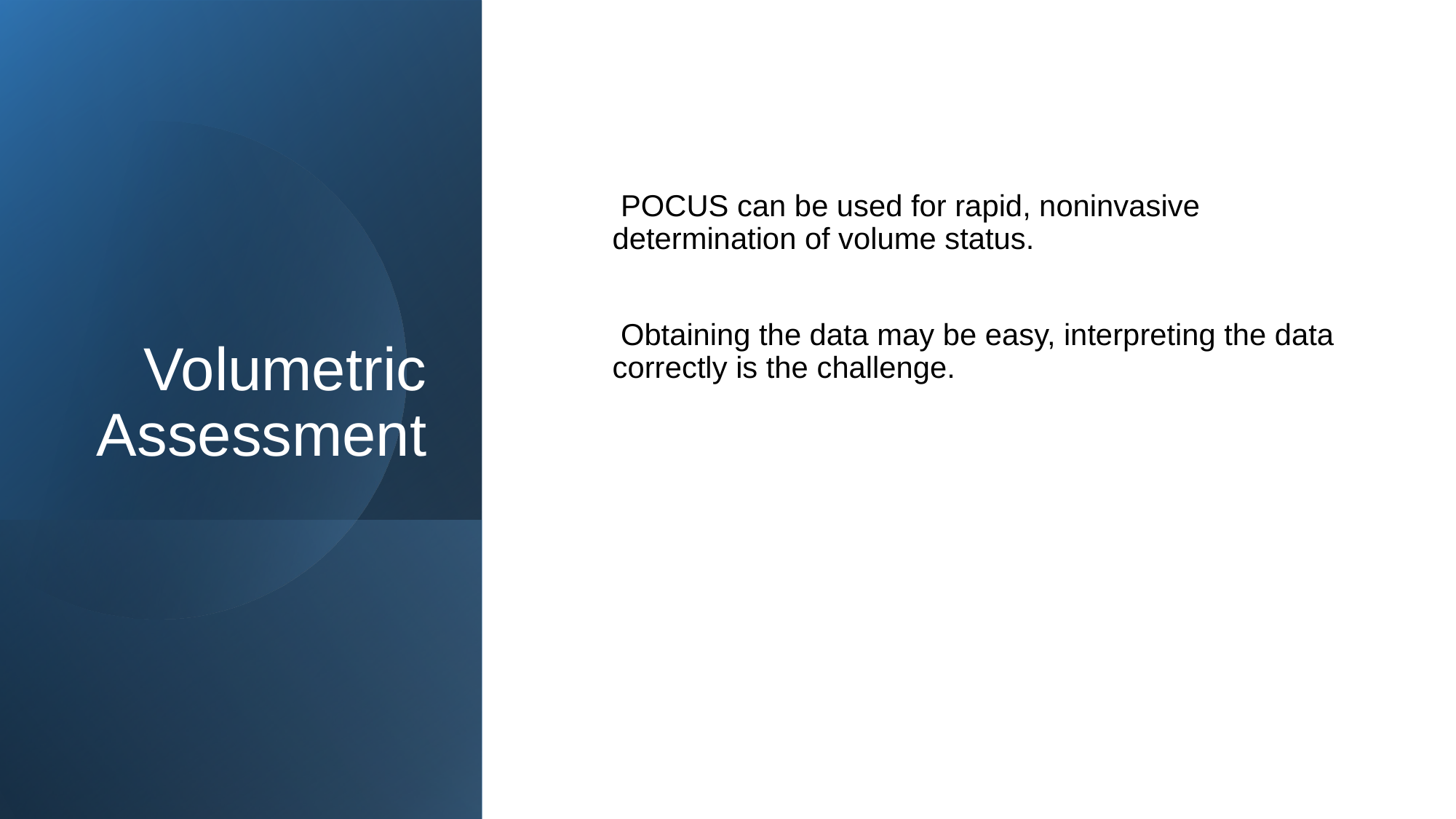

# Volumetric Assessment
 POCUS can be used for rapid, noninvasive determination of volume status.
 Obtaining the data may be easy, interpreting the data correctly is the challenge.
Pro: noninvasive, quick to obtain
Con: can be limited due to body habitus, bowel gas, patient intolerance

## Slide 27
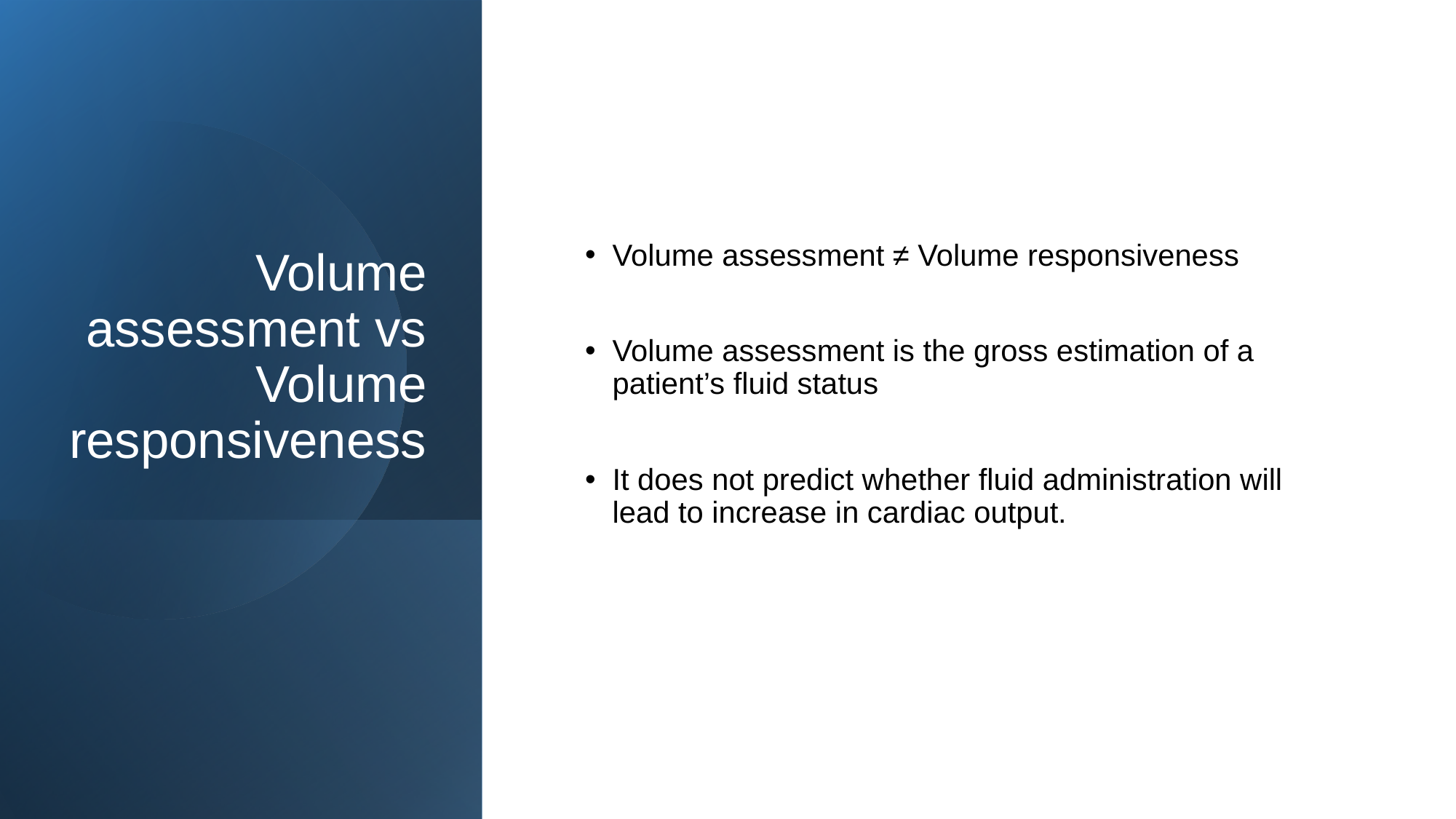

# Volume assessment vs Volume responsiveness
Volume assessment ≠ Volume responsiveness
Volume assessment is the gross estimation of a patient’s fluid status
It does not predict whether fluid administration will lead to increase in cardiac output.

## Slide 28
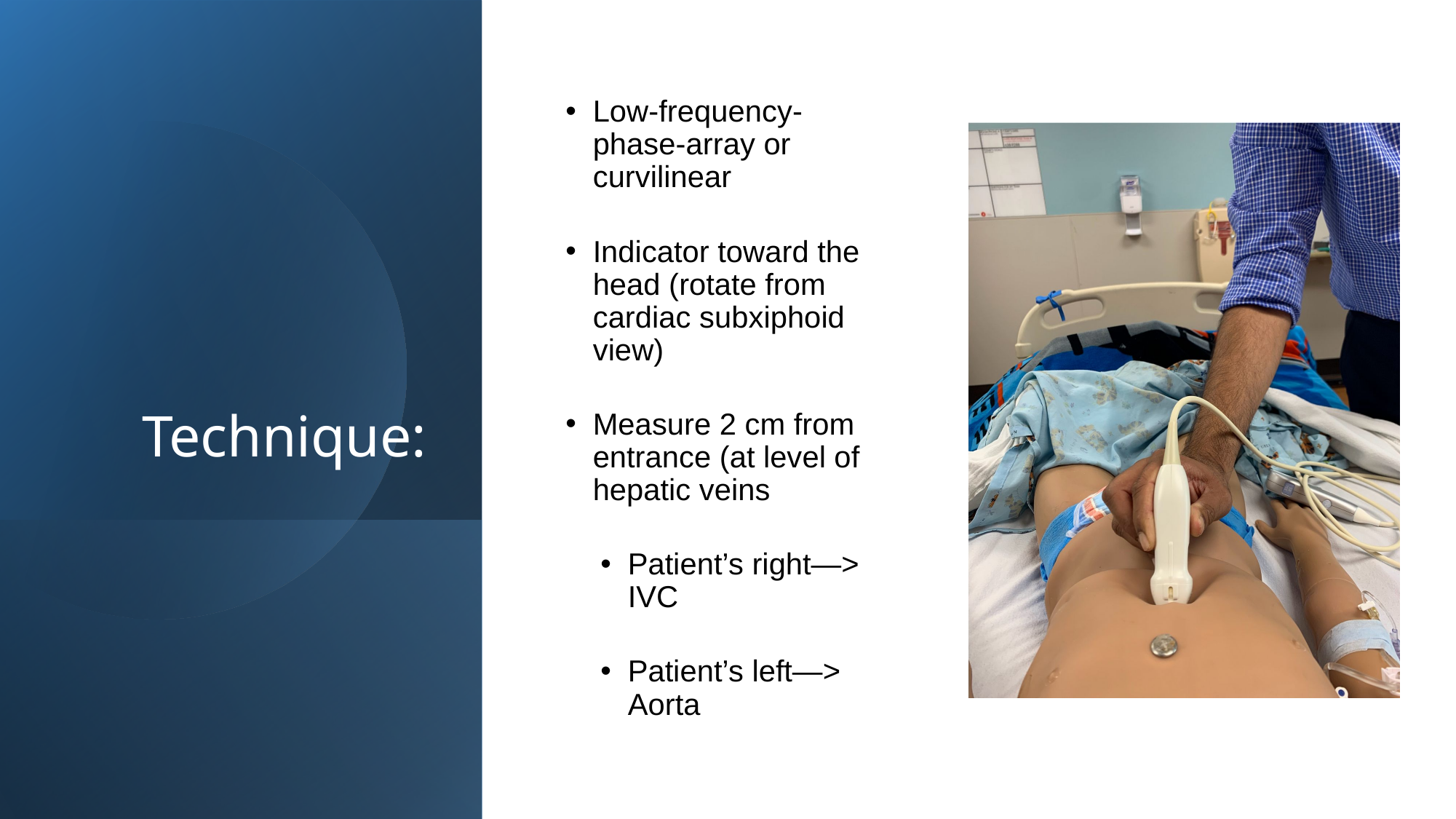

# Technique:
Low-frequency- phase-array or curvilinear
Indicator toward the head (rotate from cardiac subxiphoid view)
Measure 2 cm from entrance (at level of hepatic veins
Patient’s right—> IVC
Patient’s left—> Aorta

## Slide 29
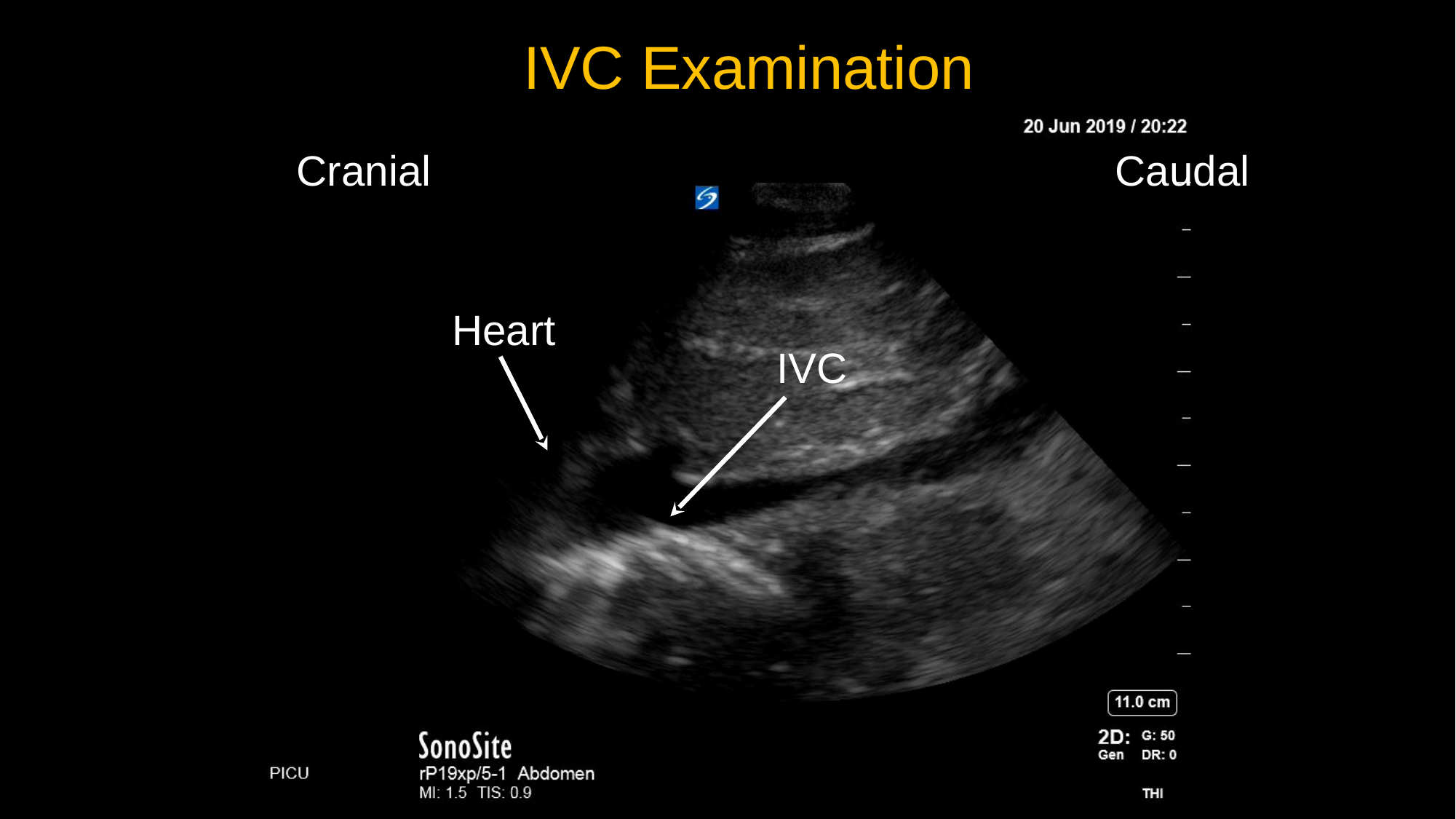

IVC Examination
Cranial
Caudal
Heart
IVC

## Slide 30
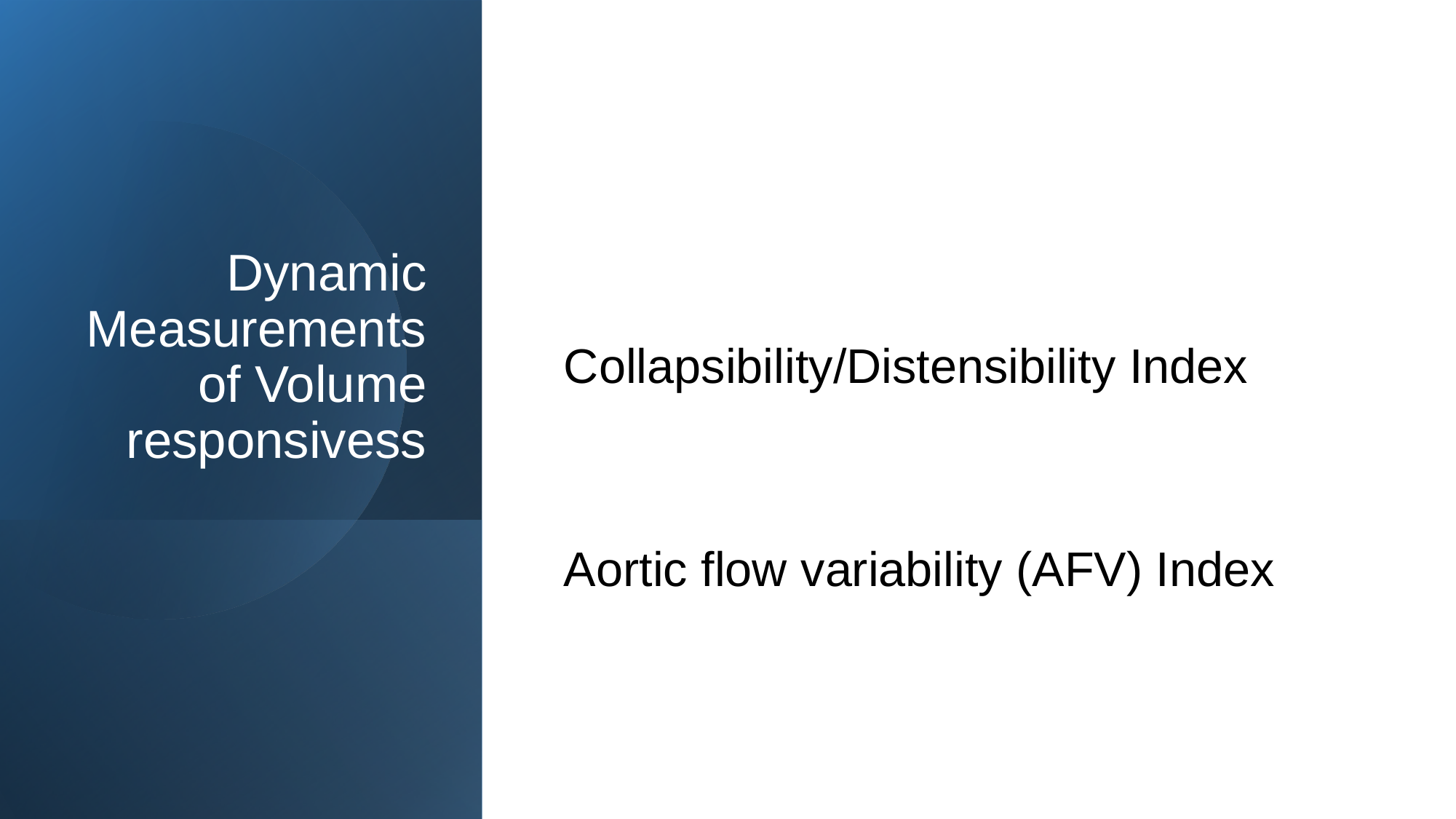

# Dynamic Measurements of Volume responsivess
Collapsibility/Distensibility Index
Aortic flow variability (AFV) Index

## Slide 31
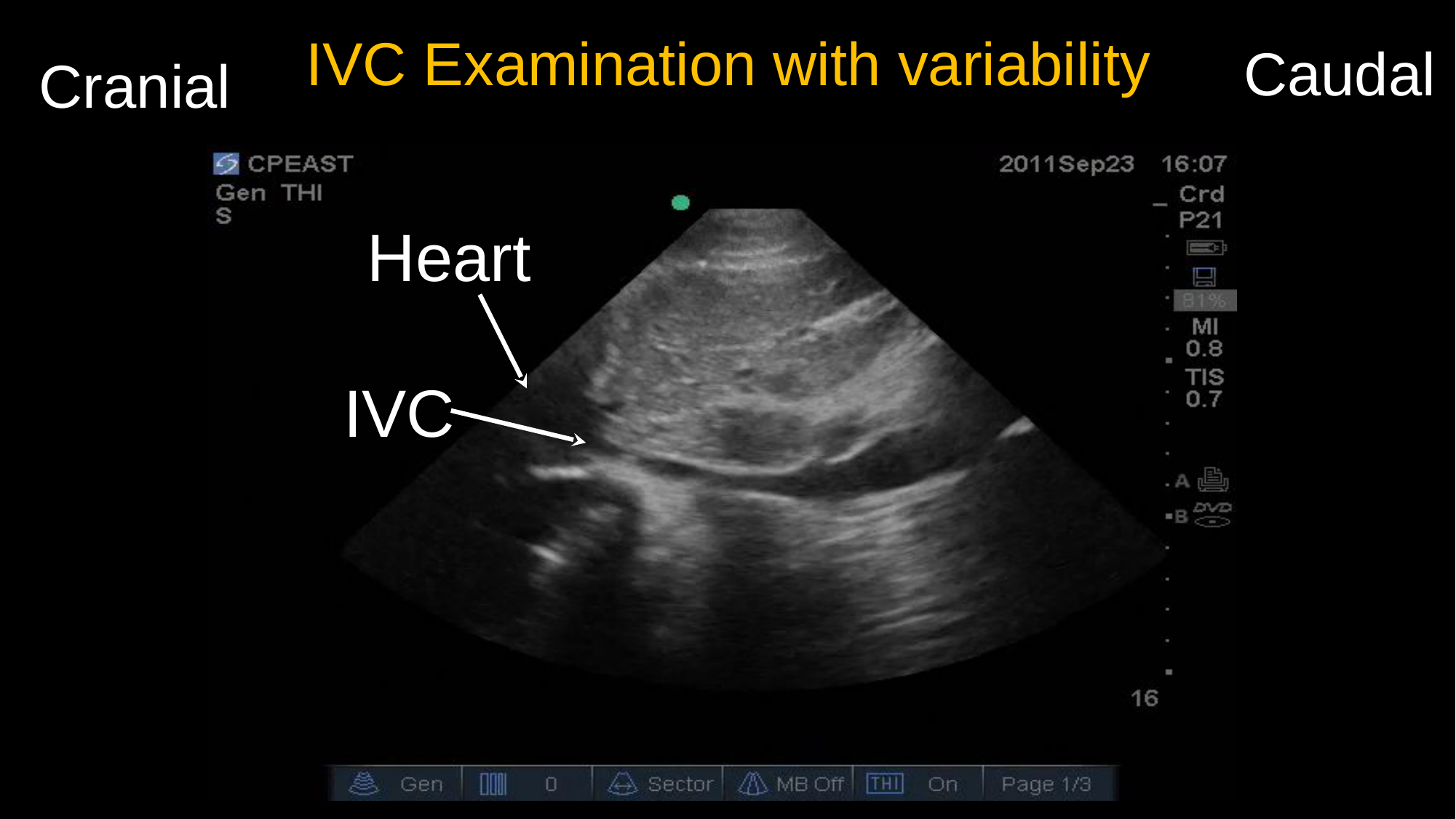

IVC Examination with variability
Caudal
Cranial
Heart
IVC
IVC-Inspiratory Collapse
IVC-No Inspiratory Collapse

## Slide 32
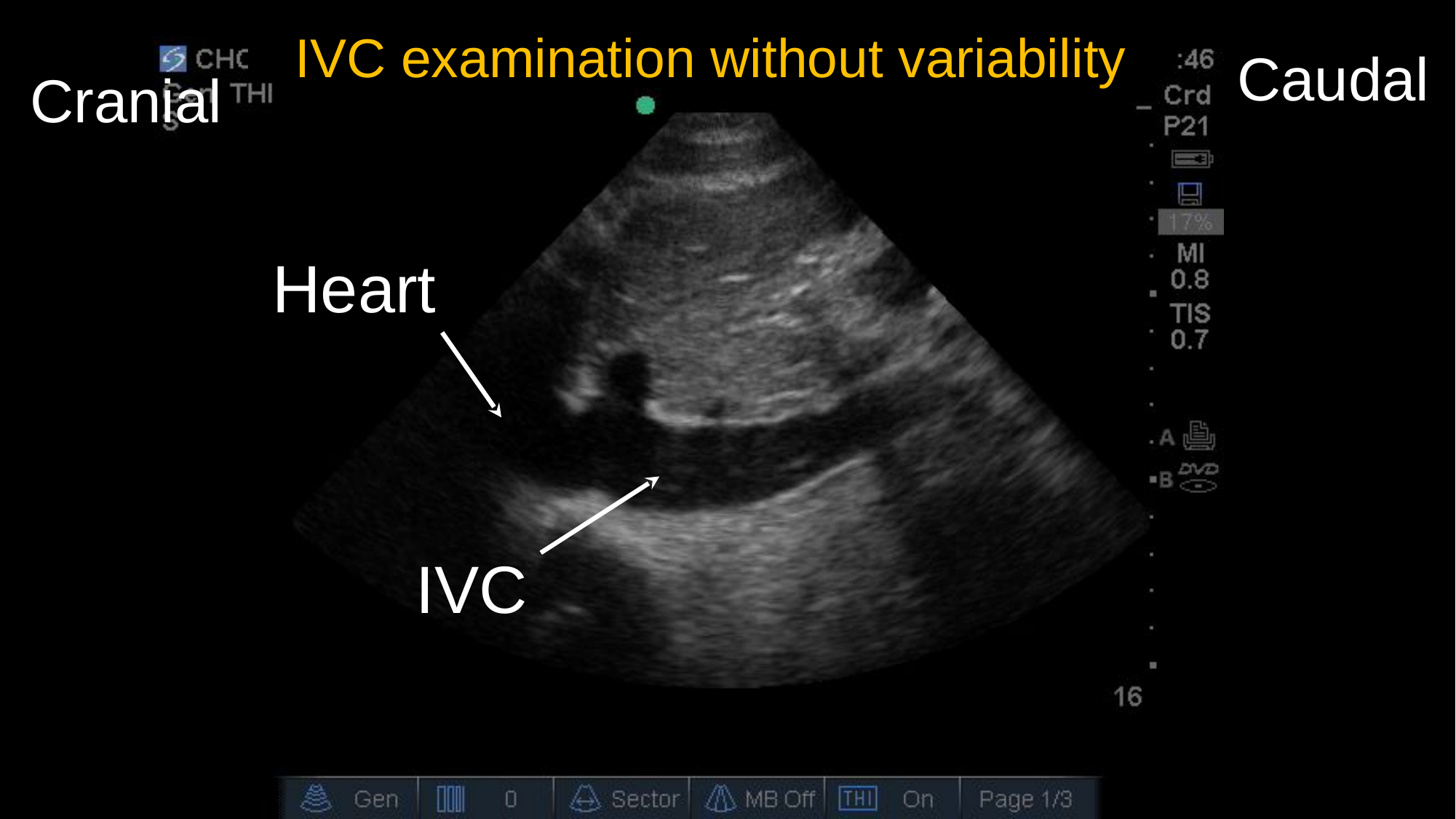

Caudal
IVC examination without variability
Cranial
Heart
IVC

## Slide 33
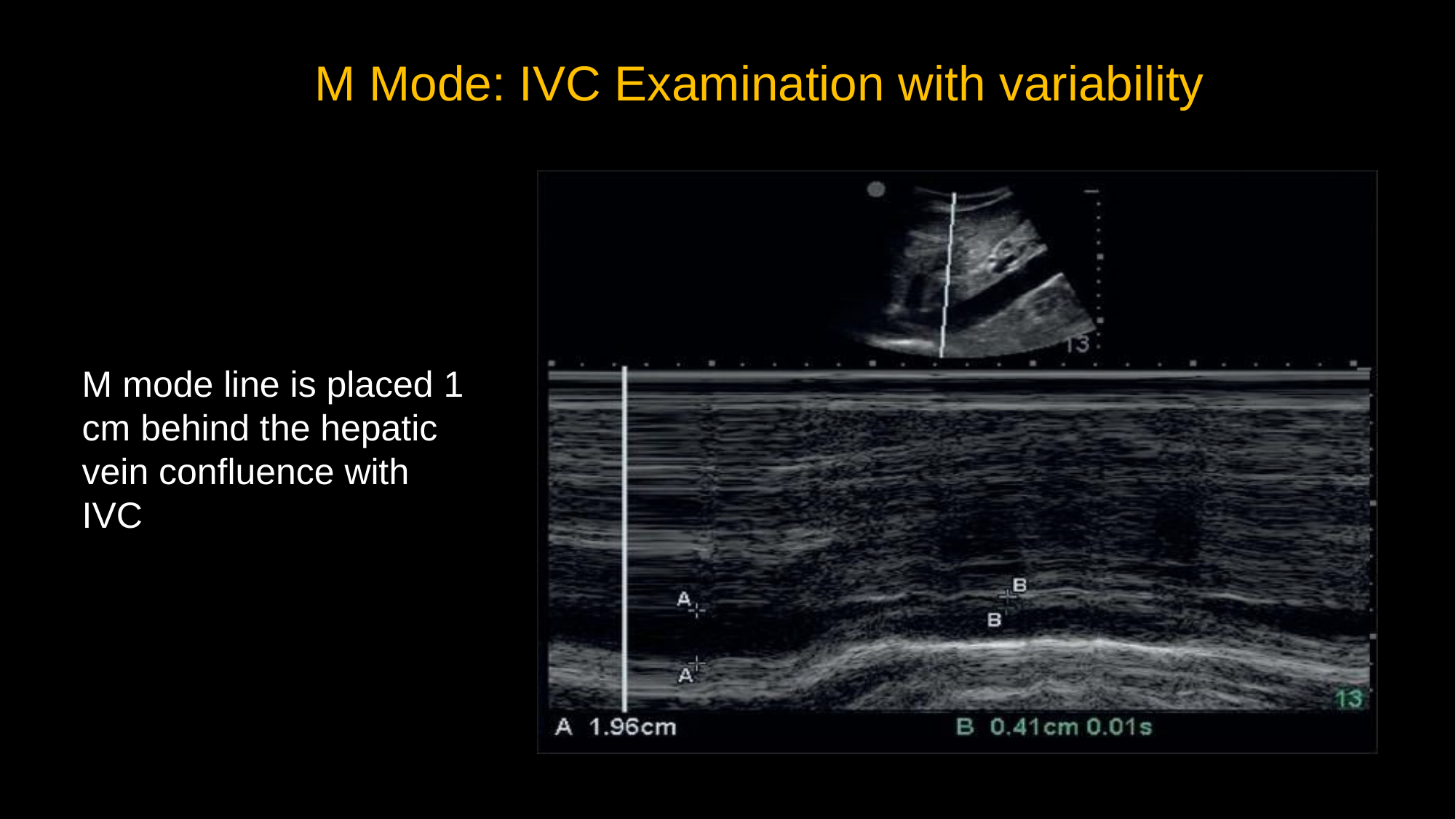

M Mode: IVC Examination with variability
M mode line is placed 1 cm behind the hepatic vein confluence with IVC

## Slide 34
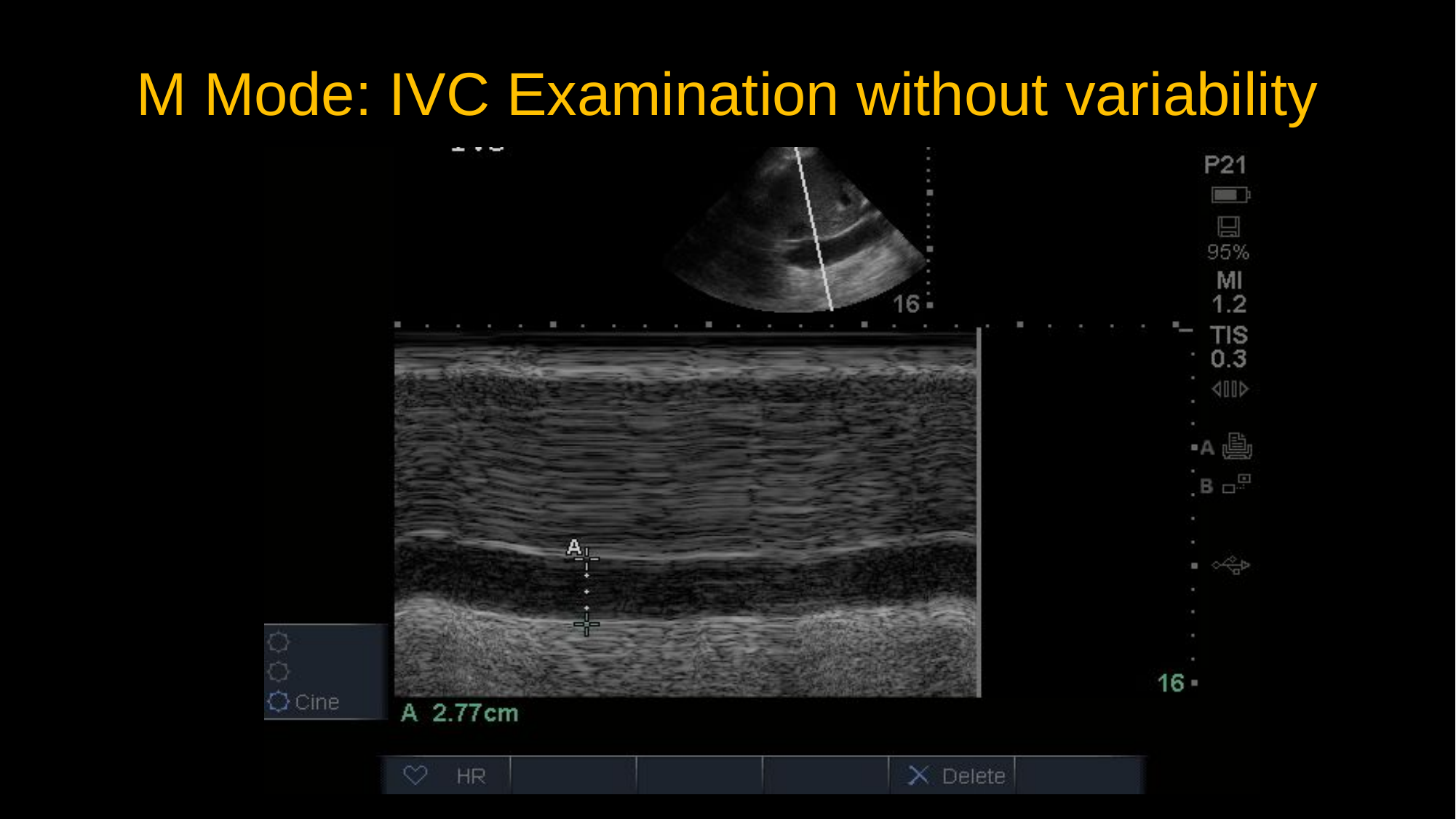

# M Mode: IVC Examination without variability

## Slide 35
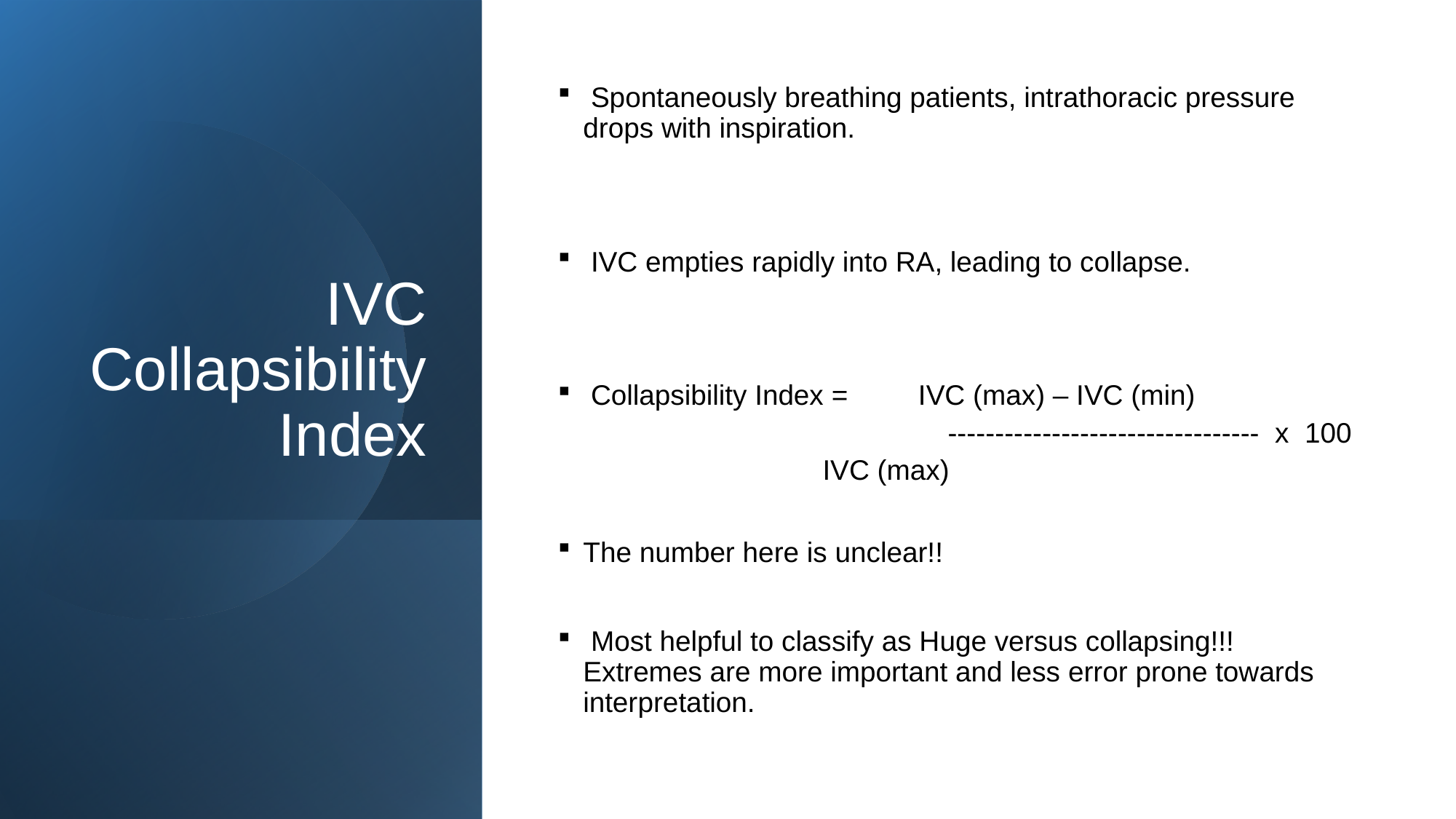

# IVC Collapsibility Index
 Spontaneously breathing patients, intrathoracic pressure drops with inspiration.
 IVC empties rapidly into RA, leading to collapse.
 Collapsibility Index = IVC (max) – IVC (min)
 	 --------------------------------- x 100
		 IVC (max)
The number here is unclear!!
 Most helpful to classify as Huge versus collapsing!!! Extremes are more important and less error prone towards interpretation.

## Slide 36
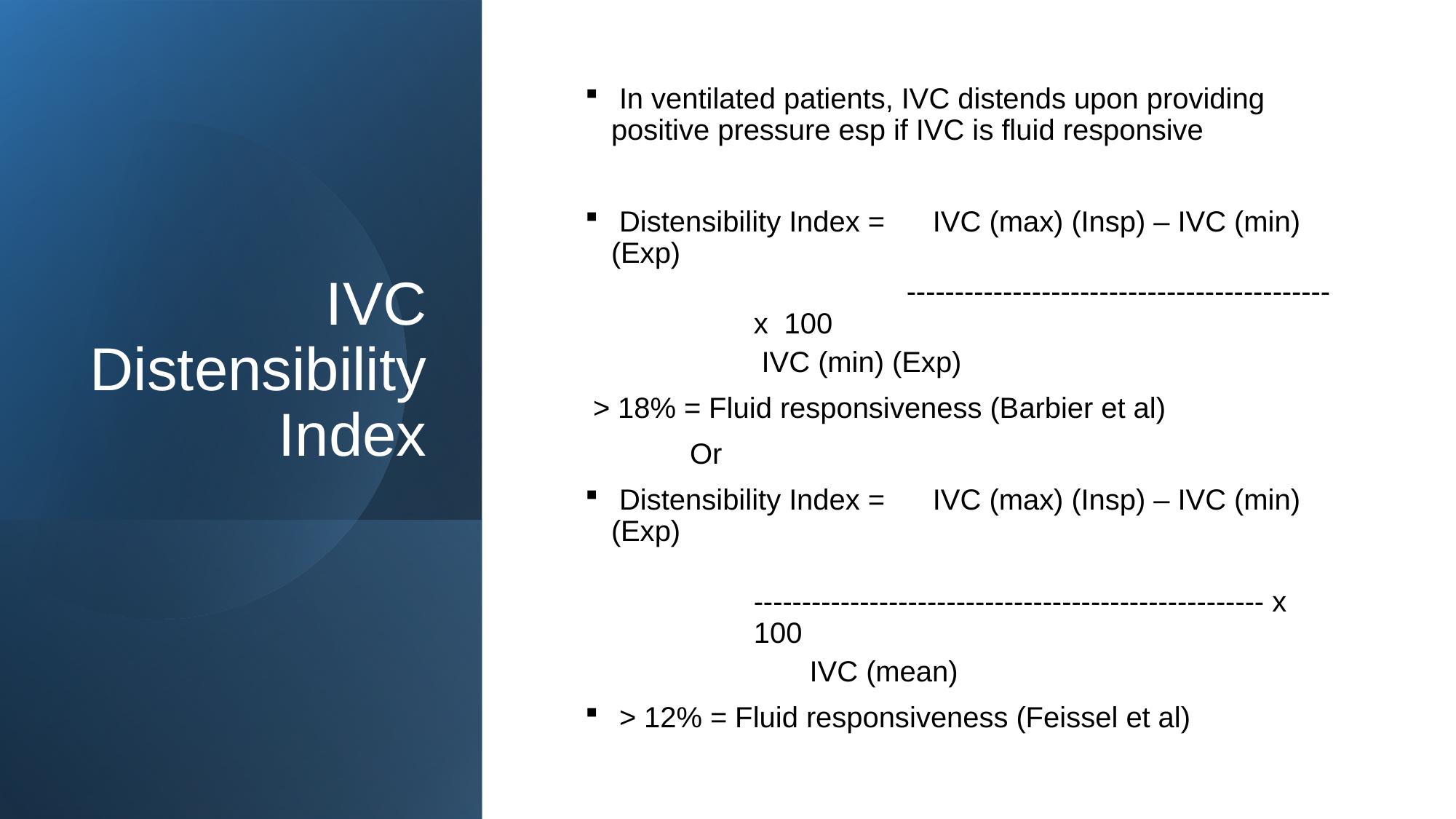

# IVC Distensibility Index
 In ventilated patients, IVC distends upon providing positive pressure esp if IVC is fluid responsive
 Distensibility Index = IVC (max) (Insp) – IVC (min) (Exp)
 	 -------------------------------------------- x 100
		 IVC (min) (Exp)
 > 18% = Fluid responsiveness (Barbier et al)
				Or
 Distensibility Index = IVC (max) (Insp) – IVC (min) (Exp)
 	 ----------------------------------------------------- x 100
		 IVC (mean)
 > 12% = Fluid responsiveness (Feissel et al)

## Slide 37
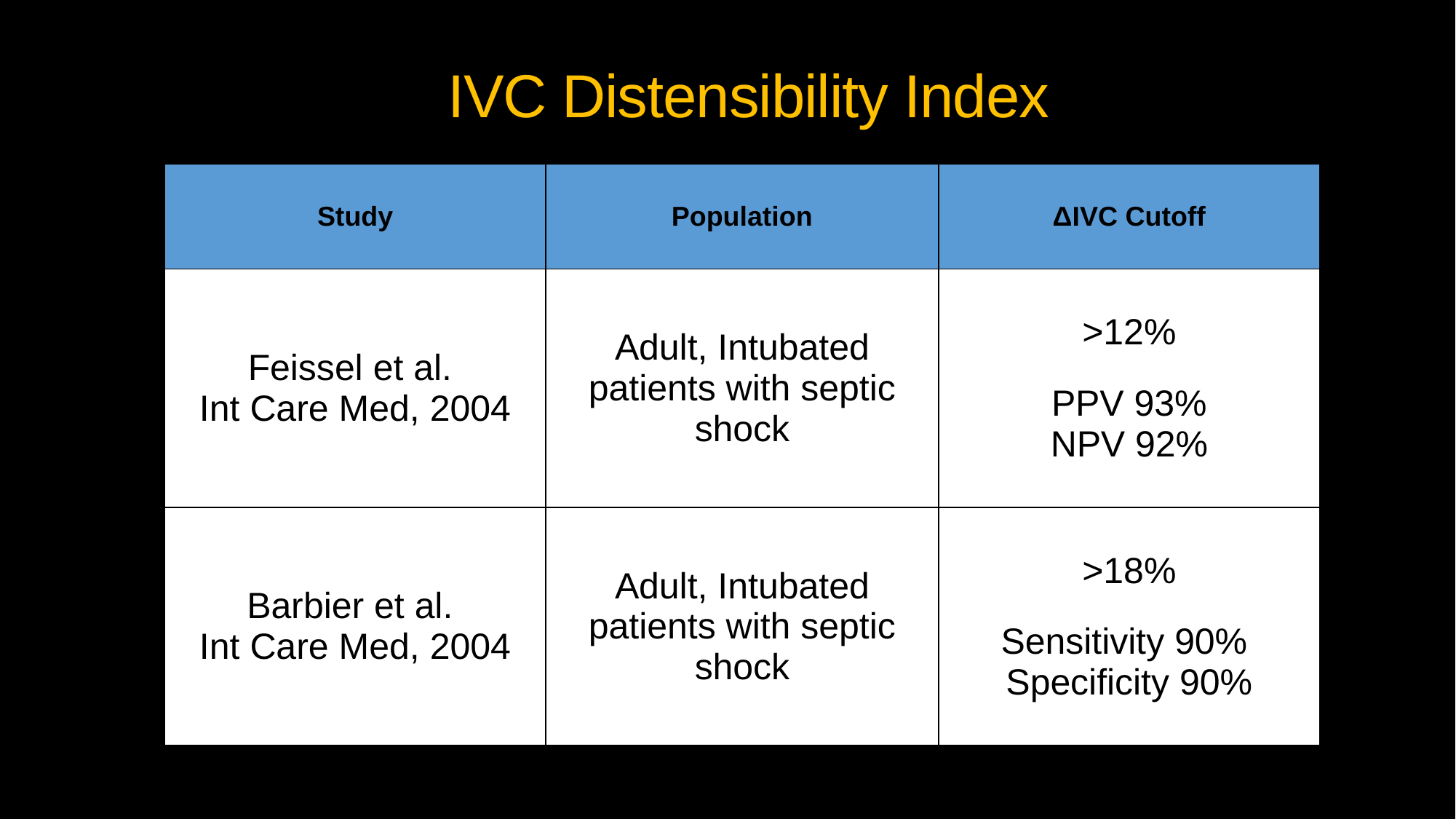

IVC Distensibility Index
| Study | Population | ΔIVC Cutoff |
| --- | --- | --- |
| Feissel et al. Int Care Med, 2004 | Adult, Intubated patients with septic shock | >12% PPV 93% NPV 92% |
| Barbier et al. Int Care Med, 2004 | Adult, Intubated patients with septic shock | >18% Sensitivity 90% Specificity 90% |

## Slide 38
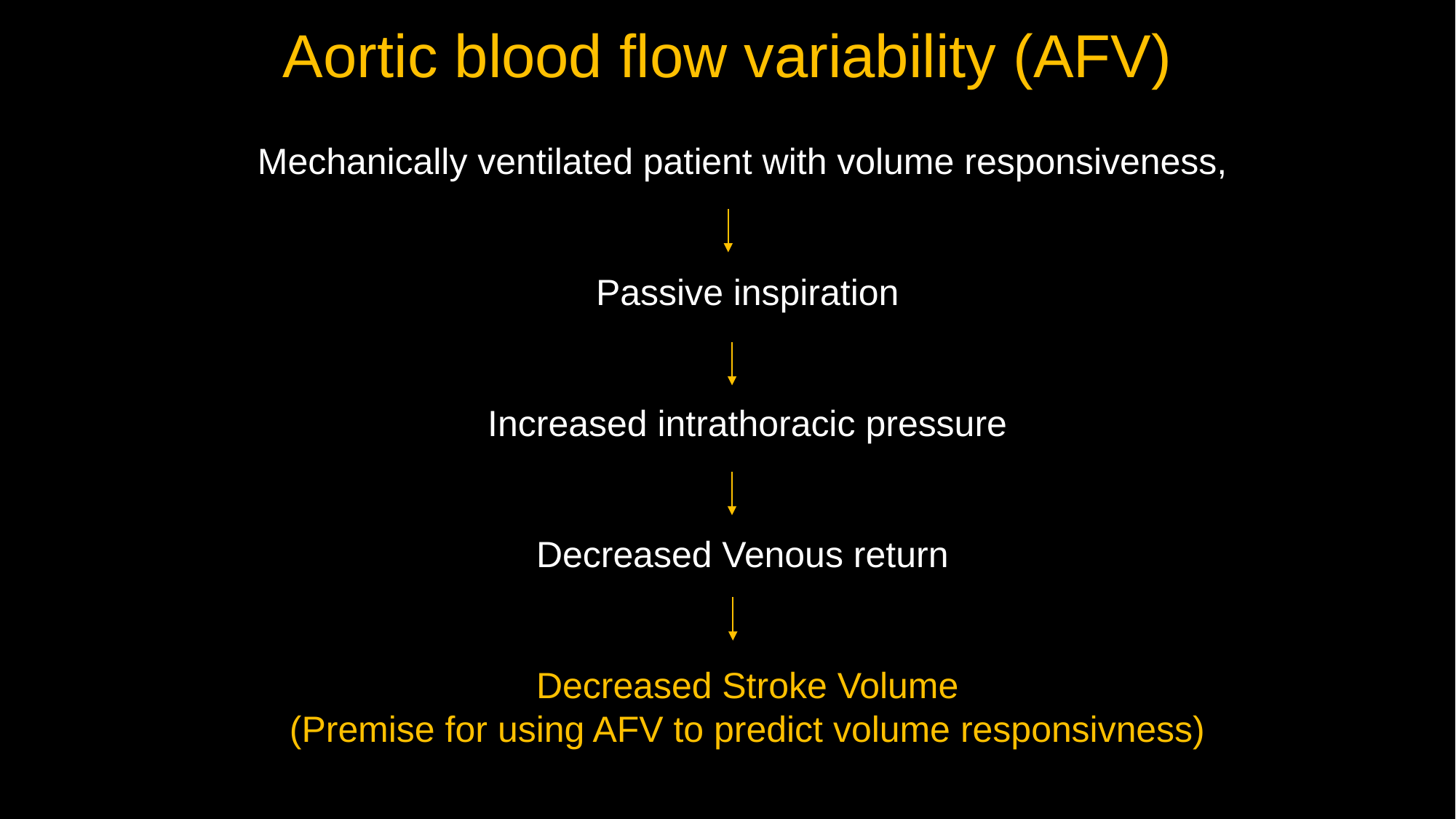

# Aortic blood flow variability (AFV)
Mechanically ventilated patient with volume responsiveness,
Passive inspiration
Increased intrathoracic pressure
Decreased Venous return
Decreased Stroke Volume
(Premise for using AFV to predict volume responsivness)

## Slide 39
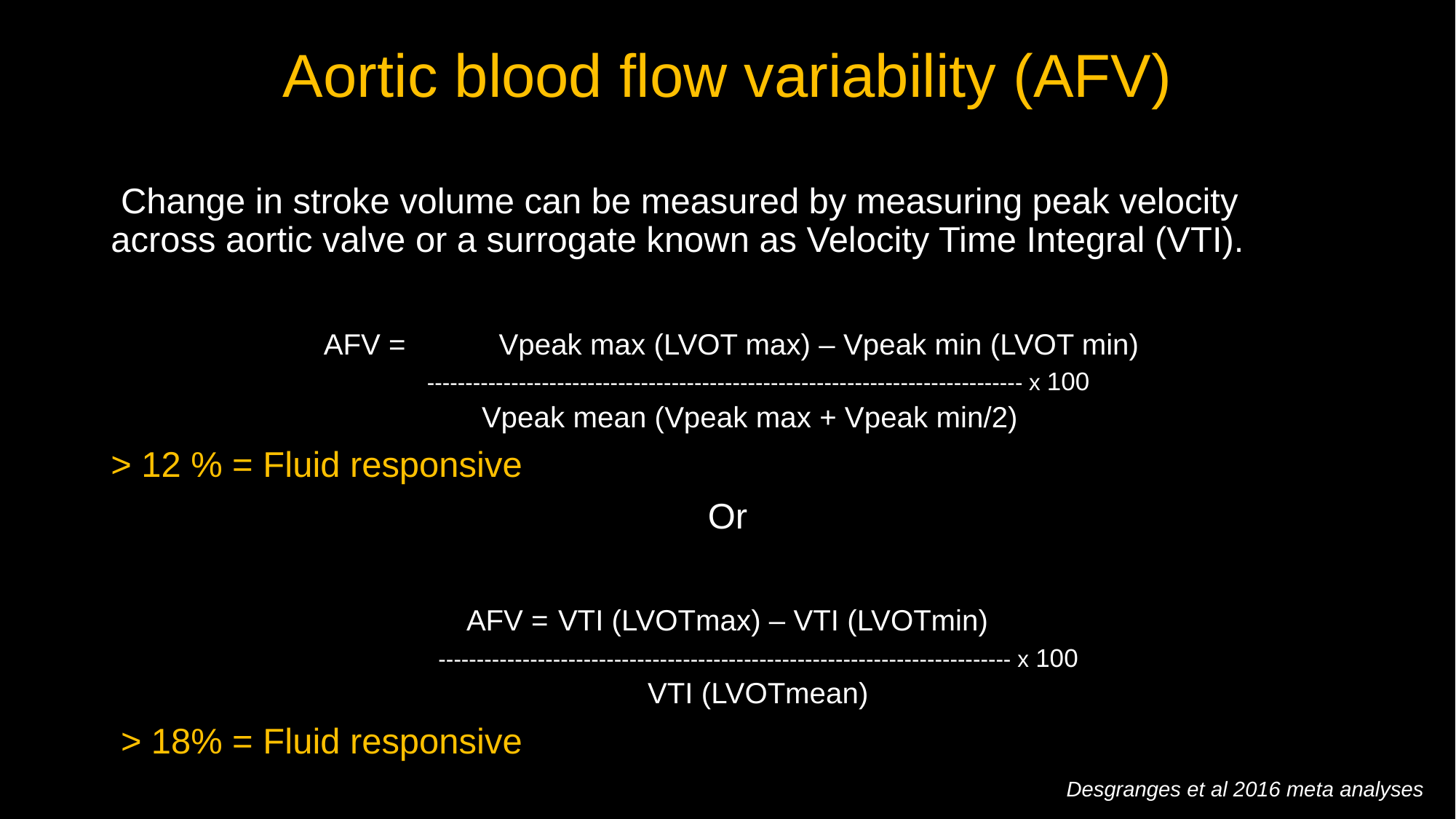

# Aortic blood flow variability (AFV)
 Change in stroke volume can be measured by measuring peak velocity across aortic valve or a surrogate known as Velocity Time Integral (VTI).
 AFV = 	Vpeak max (LVOT max) – Vpeak min (LVOT min)
	------------------------------------------------------------------------------ x 100
Vpeak mean (Vpeak max + Vpeak min/2)
> 12 % = Fluid responsive
Or
AFV = 		VTI (LVOTmax) – VTI (LVOTmin)
		--------------------------------------------------------------------------- x 100
VTI (LVOTmean)
 > 18% = Fluid responsive
Desgranges et al 2016 meta analyses

## Slide 40
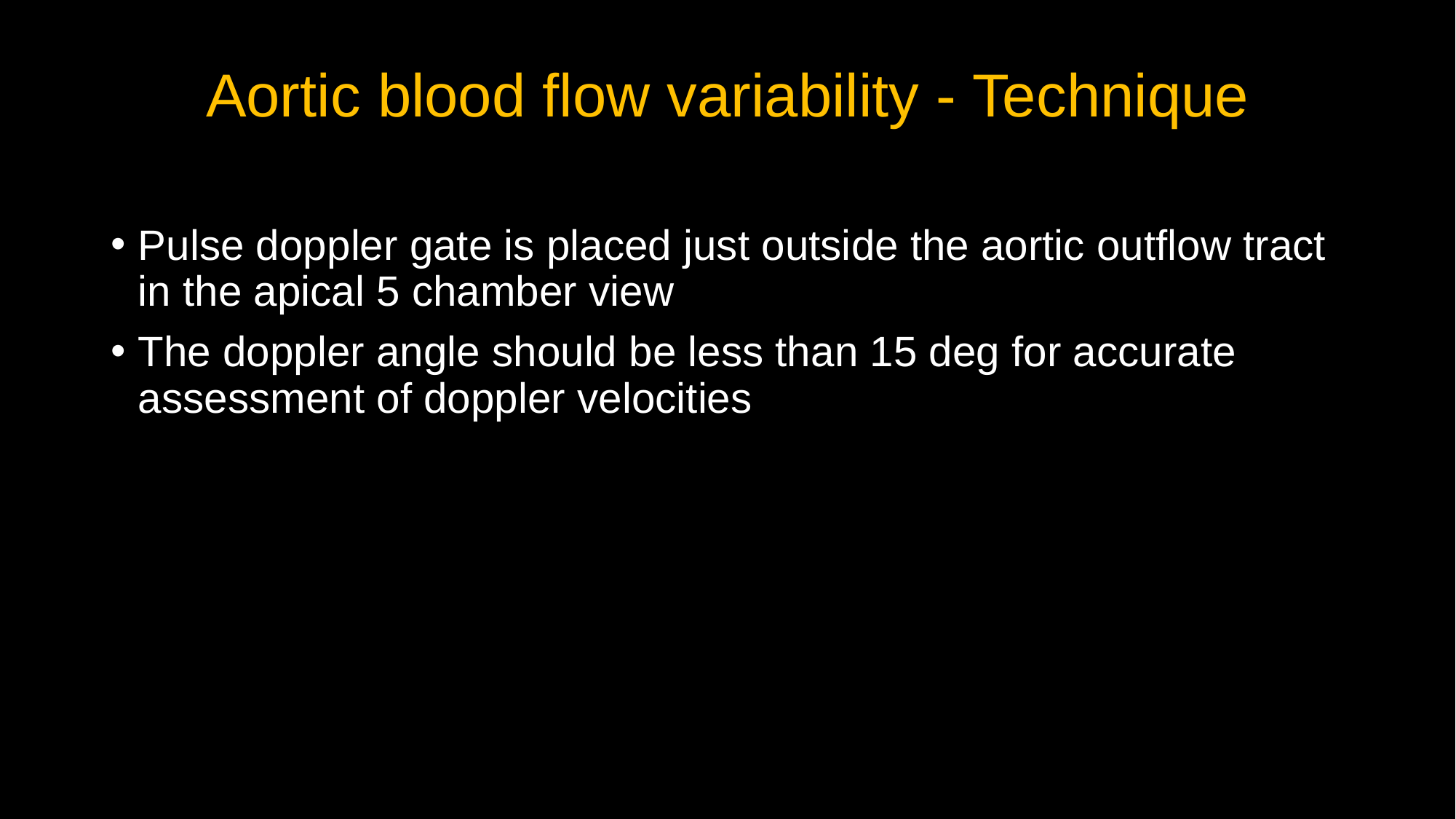

# Aortic blood flow variability - Technique
Pulse doppler gate is placed just outside the aortic outflow tract in the apical 5 chamber view
The doppler angle should be less than 15 deg for accurate assessment of doppler velocities

## Slide 41
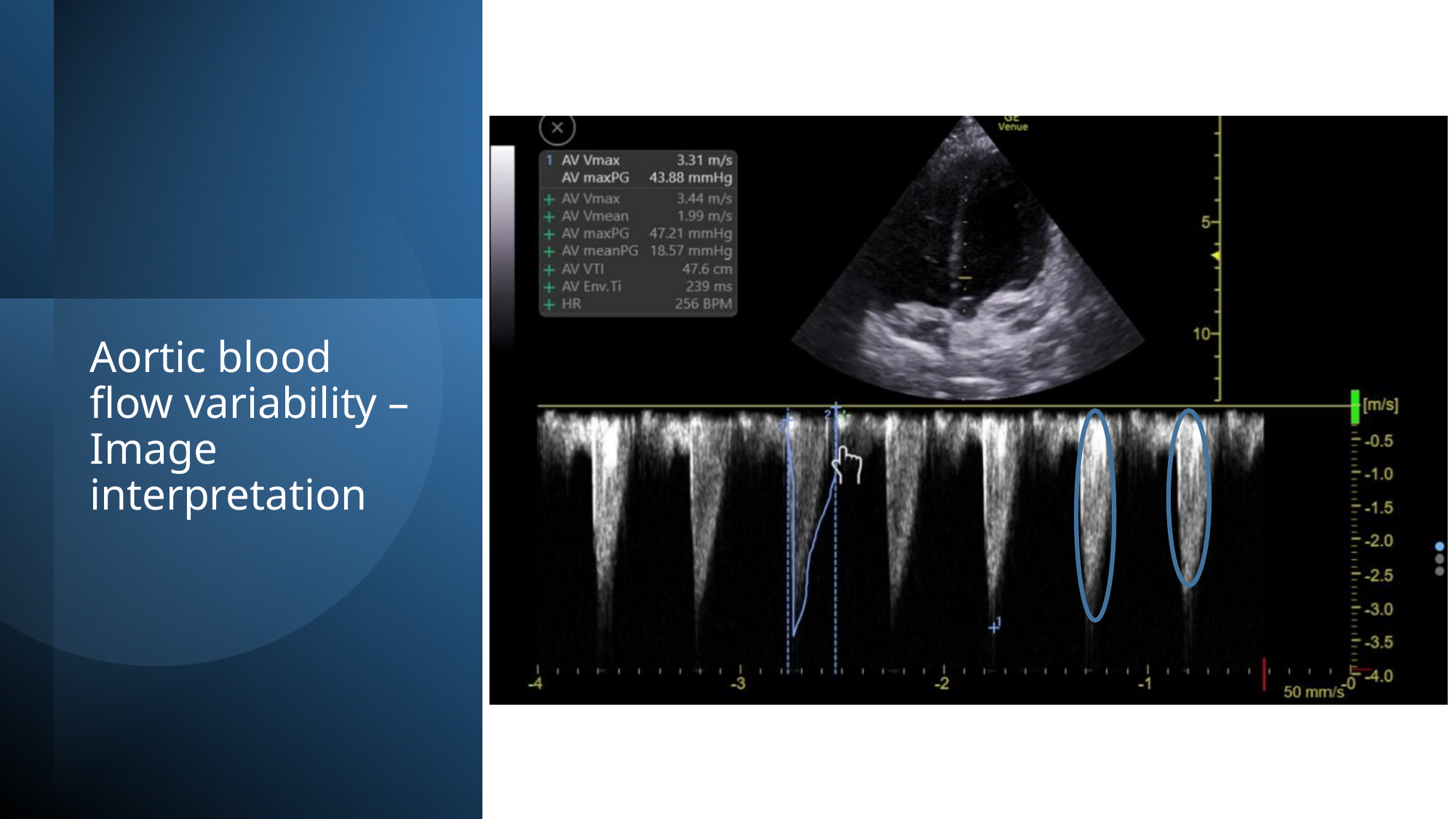

# Aortic blood flow variability – Image interpretation

## Slide 42
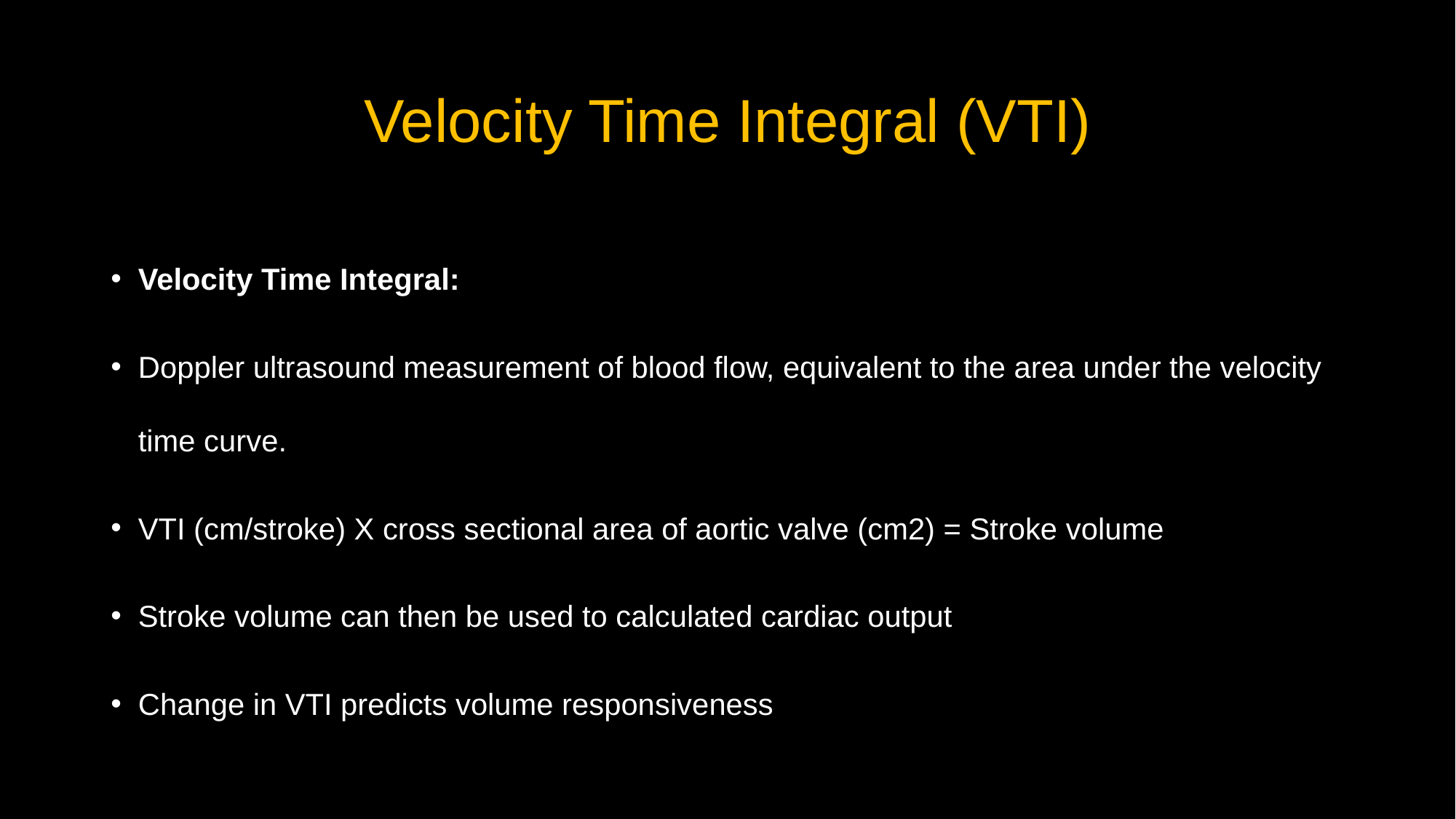

# Velocity Time Integral (VTI)
Velocity Time Integral:
Doppler ultrasound measurement of blood flow, equivalent to the area under the velocity time curve.
VTI (cm/stroke) X cross sectional area of aortic valve (cm2) = Stroke volume
Stroke volume can then be used to calculated cardiac output
Change in VTI predicts volume responsiveness

## Slide 43
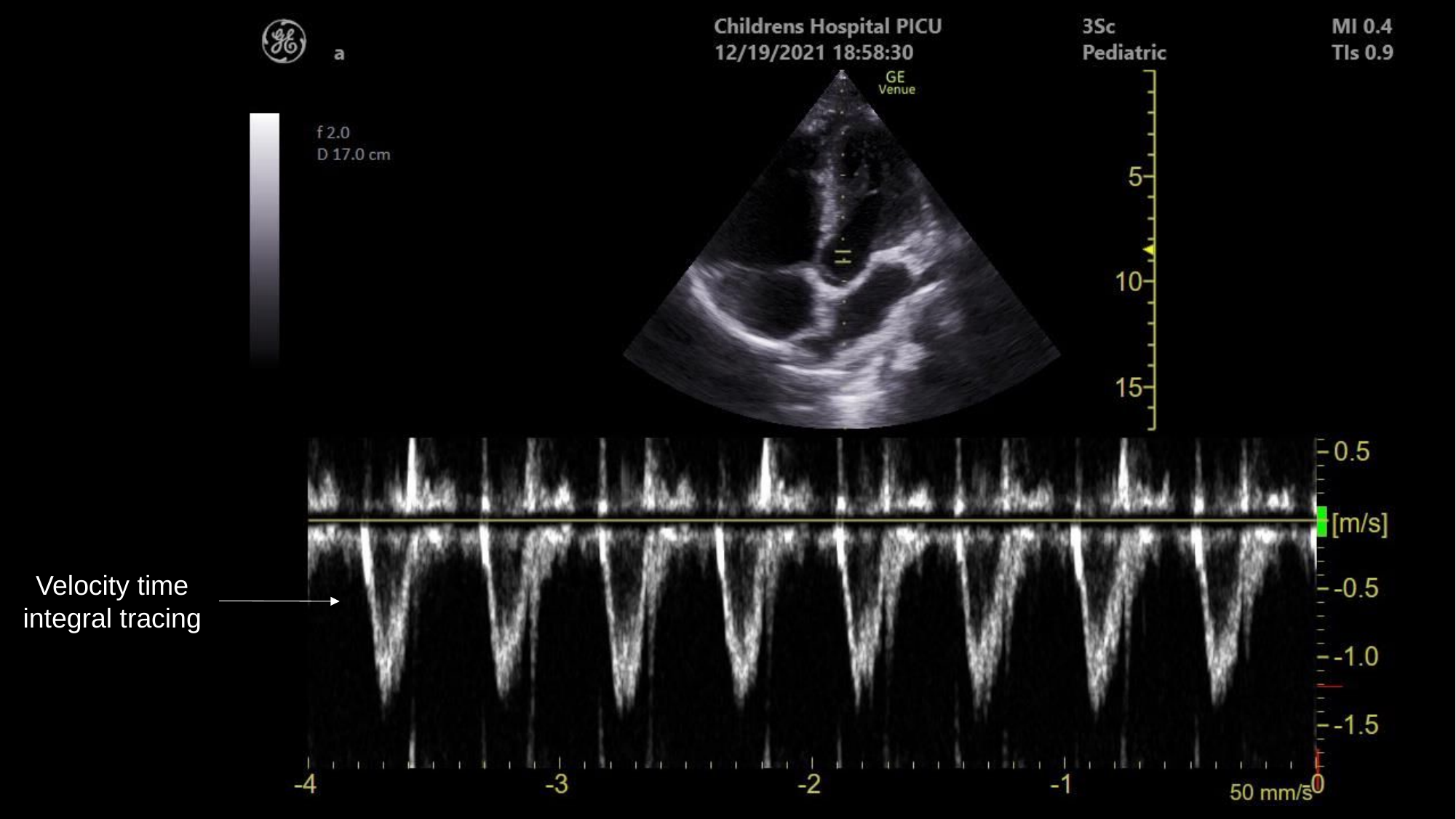

Velocity time integral tracing

## Slide 44
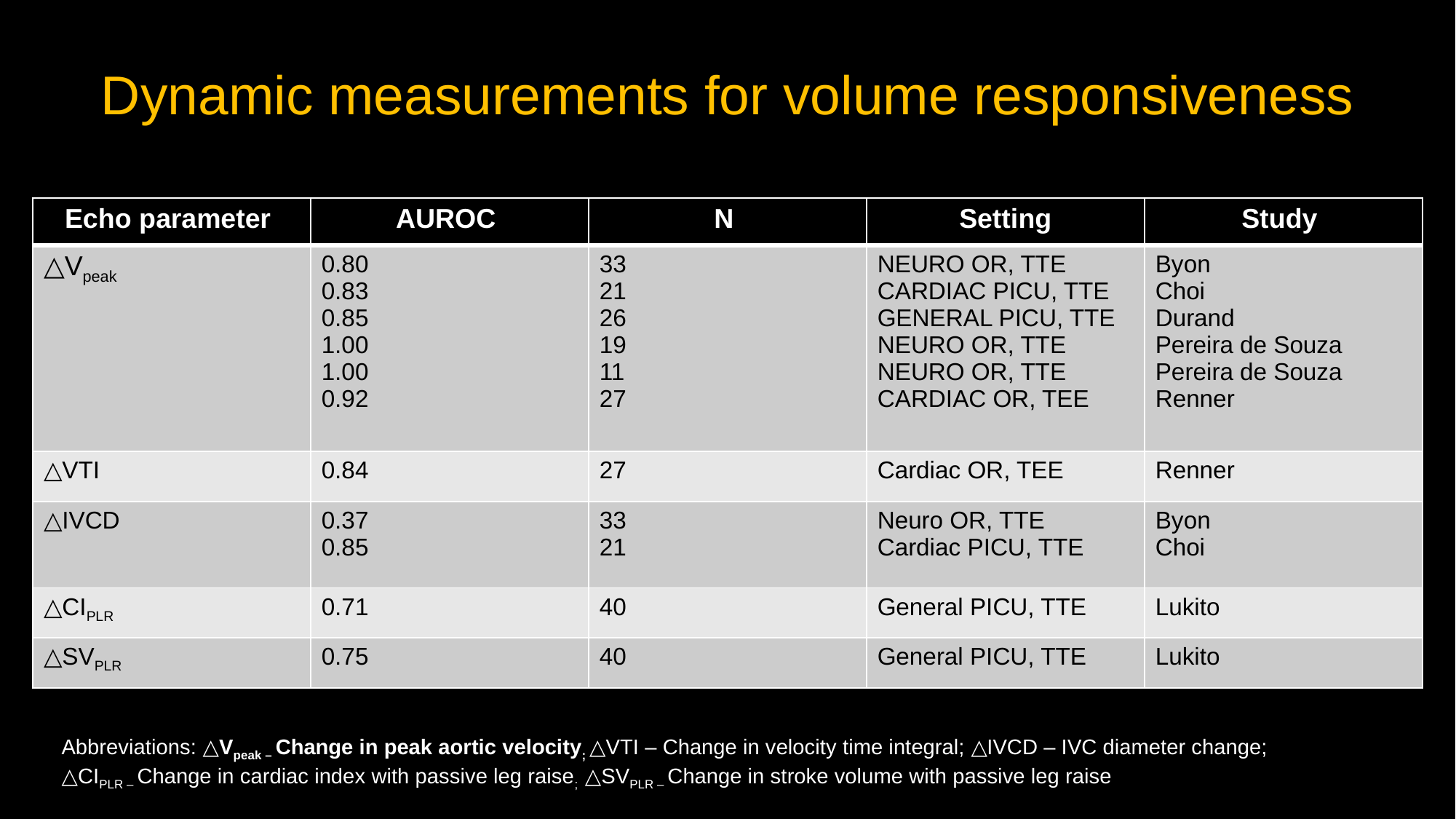

# Dynamic measurements for volume responsiveness
| Echo parameter | AUROC | N | Setting | Study |
| --- | --- | --- | --- | --- |
| △Vpeak | 0.80 0.83 0.85 1.00 1.00 0.92 | 33 21 26 19 11 27 | NEURO OR, TTE CARDIAC PICU, TTE GENERAL PICU, TTE NEURO OR, TTE NEURO OR, TTE CARDIAC OR, TEE | Byon Choi Durand Pereira de Souza Pereira de Souza Renner |
| △VTI | 0.84 | 27 | Cardiac OR, TEE | Renner |
| △IVCD | 0.37 0.85 | 33 21 | Neuro OR, TTE Cardiac PICU, TTE | Byon Choi |
| △CIPLR | 0.71 | 40 | General PICU, TTE | Lukito |
| △SVPLR | 0.75 | 40 | General PICU, TTE | Lukito |
Abbreviations: △Vpeak – Change in peak aortic velocity; △VTI – Change in velocity time integral; △IVCD – IVC diameter change; △CIPLR – Change in cardiac index with passive leg raise; △SVPLR – Change in stroke volume with passive leg raise

## Slide 45
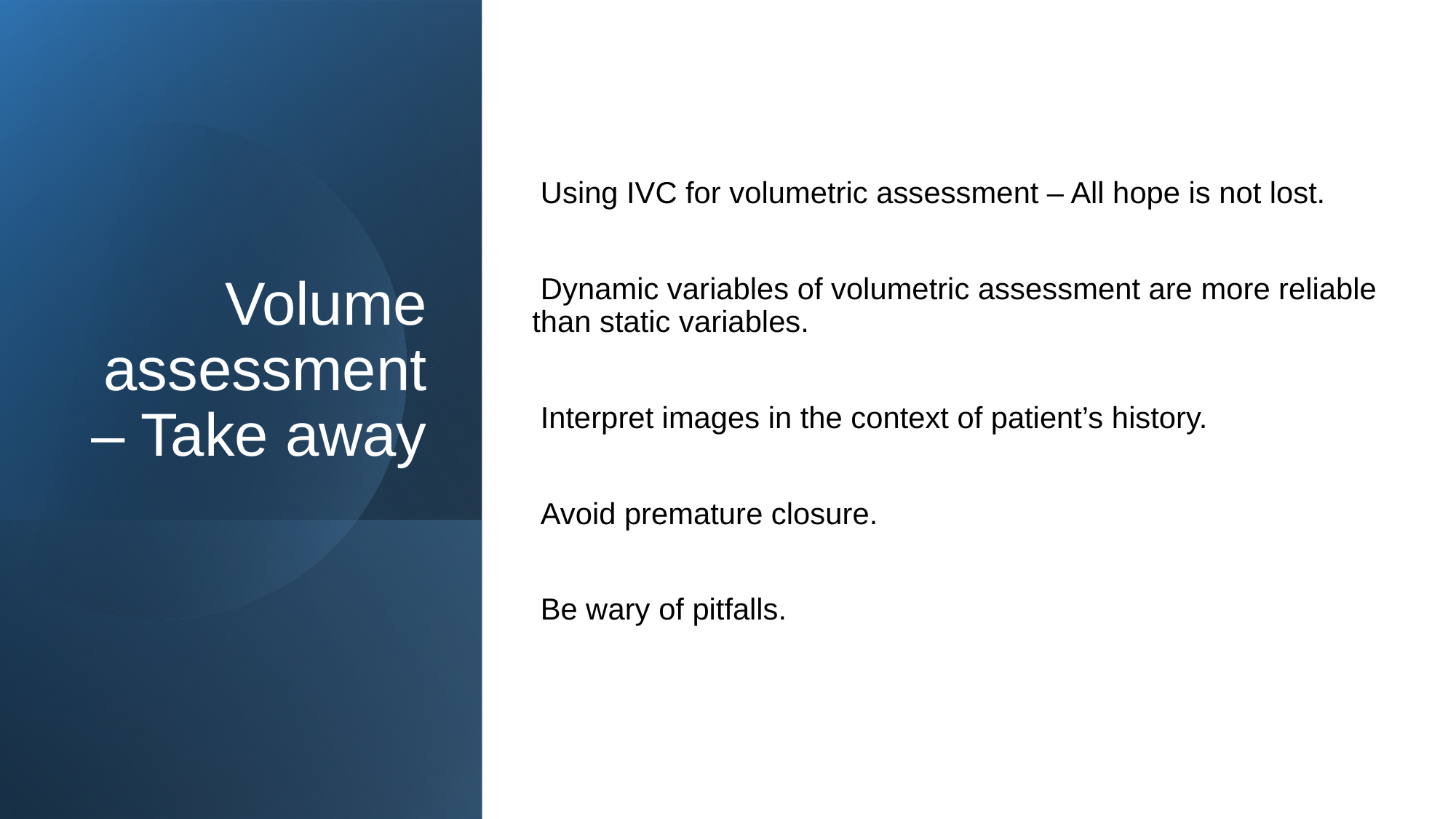

Using IVC for volumetric assessment – All hope is not lost.
 Dynamic variables of volumetric assessment are more reliable than static variables.
 Interpret images in the context of patient’s history.
 Avoid premature closure.
 Be wary of pitfalls.
# Volume assessment – Take away

## Slide 46
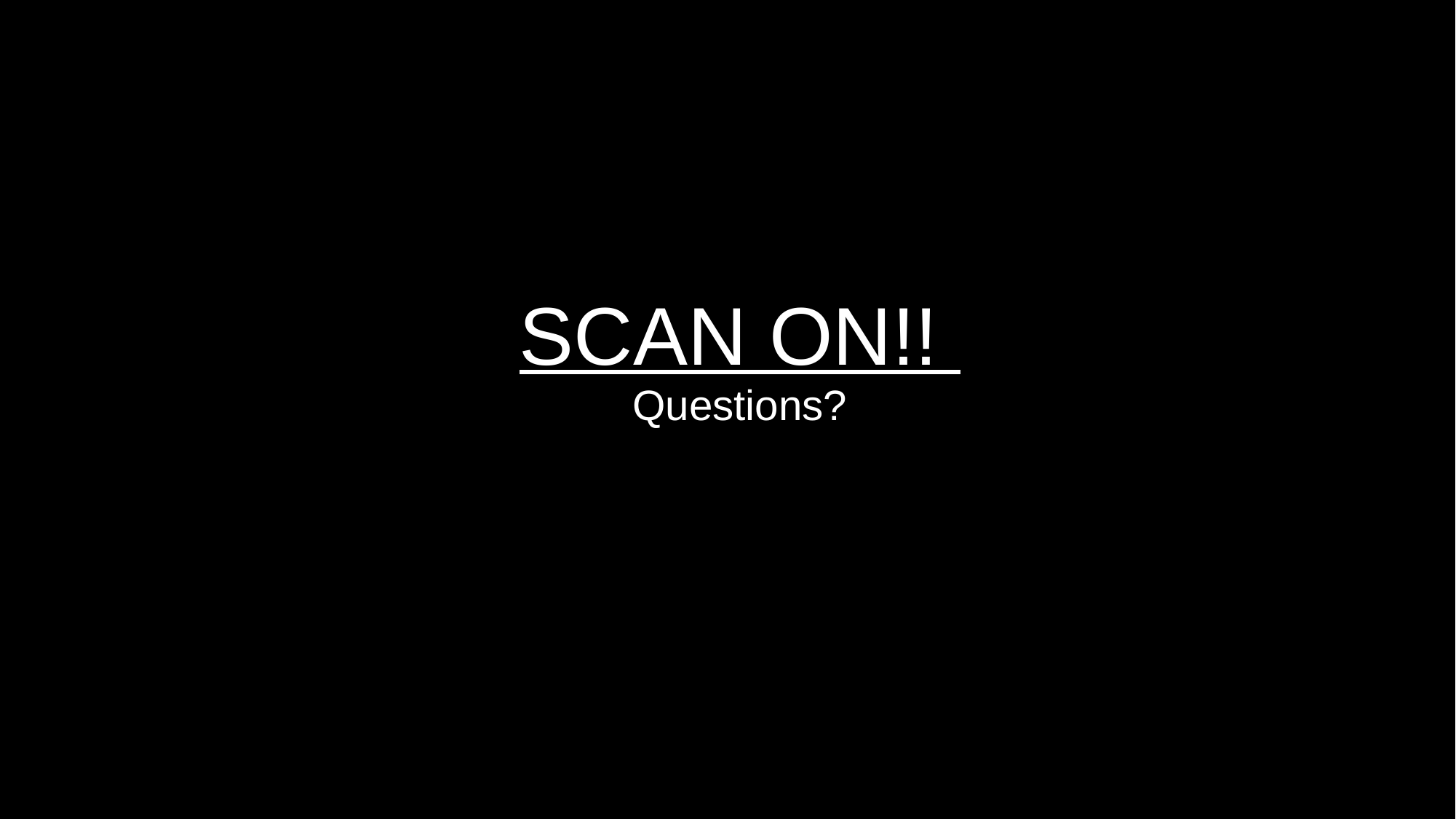

# SCAN ON!!
Questions?
