# Supplementary material for: A point-of-care ultrasound education curriculum for pediatric critical care medicine
Source: Ultrasound J. 2022 Oct 31;14:44. doi: 10.1186/s13089-022-00290-6 (PMC9622960; doi:10.1186/s13089-022-00290-6)
Supplement: Supplementary file 10 — Additional file 10. Post-course survey to assess the impact of the curriculum on the learners [file 13089_2022_290_MOESM10_ESM.docx]

Q1 Please write your initials and year of birth. (ex: JD1987 for John Doe year of birth 1987)

- ________________________________________________

Q2 How comfortable do you feel in your procedural point of care ultrasound skills?

Extremely comfortable

Moderately comfortable

Slightly comfortable

Neither comfortable nor uncomfortable

Slightly uncomfortable

Moderately uncomfortable

Extremely uncomfortable

Q3 Have you integrated diagnostic POCUS into patient assessment.

Yes

No
