# Supplementary material for: A point-of-care ultrasound education curriculum for pediatric critical care medicine
Source: Ultrasound J. 2022 Oct 31;14:44. doi: 10.1186/s13089-022-00290-6 (PMC9622960; doi:10.1186/s13089-022-00290-6)
Supplement: Supplementary file 11 — Additional file 11. High-quality, free open access resources for point-of-care ultrasound education [file 13089_2022_290_MOESM11_ESM.docx]

List of POCUS resources

Free open access online sources

[www.coreultrasound.com](http://www.coreultrasound.com)

[www.edccus.com](http://www.edccus.com)

[www.thepocusatlas.com](http://www.thepocusatlas.com)

[www.acep.org](http://www.acep.org)

[www.criticalecho.com](http://www.criticalecho.com)

[www.pocus101.com](http://www.pocus101.com)

[www.pie.med.utoronto.ca/POCUS](http://www.pie.med.utoronto.ca/POCUS)

POCUS books:

Practical guide to critical ultrasound

Introduction to bedside ultrasound Vol 1& 2
